# Supplementary material for: Comparative Effectiveness of Multi-Component, Exercise-Based Interventions for Preventing Soccer-Related Musculoskeletal Injuries: A Systematic Review and Meta-Analysis
Source: Healthcare (Basel). 2025 Mar 29;13(7):765. doi: 10.3390/healthcare13070765 (PMC11988859; doi:10.3390/healthcare13070765)
Supplement: Supplementary file 1 [file healthcare-13-00765-s001.zip › Additional material Included literature/emery2010.pdf]

Downloaded from bjsm.bmj.com on November 17, 2012 - Published by group.bmj.com

The effectiveness of a neuromuscular prevention strategy to reduce injuries in youth soccer: a cluster-randomised controlled trial

C A Emery, W H Meeuwisse

► An appendix is published online only at http://bjsm.bmj.com

Sport Medicine Centre, Roger Jackson Centre for Health and Wellness Research, Faculty of Kinesiology and Community Health Sciences and Pediatrics, Faculty of Medicine, University of Calgary, Calgary, Alberta, Canada

Correspondence to
Professor C A Emery, Faculty of Kinesiology, Sport Medicine Centre, University of Calgary, 2500 University Drive NW, Calgary, Alberta, T2N1N4, Canada; caemery@ucalgary.ca

Accepted 15 April 2010

ABSTRACT

Background Soccer is a leading sport for participation and injury in youth.

Objective To examine the effectiveness of a neuromuscular prevention strategy in reducing injury in youth soccer players.

Design Cluster-randomised controlled trial.

Setting Calgary soccer clubs (male or female, U13–U18, tier 1–2, indoor soccer).

Participants Eighty-two soccer teams were approached for recruitment. Players from 60 teams completed the study (32 training (n=380), 28 control (n=364)).

Intervention The training programme was a soccer-specific neuromuscular training programme including dynamic stretching, eccentric strength, agility, jumping and balance (including a home-based balance training programme using a wobble board). The control programme was a standardised warm-up (static and dynamic stretching and aerobic components) and a home-based stretching programme.

Main outcome measures Previously validated injury surveillance included injury assessment by a study therapist. The injury definition was soccer injury resulting in medical attention and/or removal from a session and/or time loss.

Results The injury rate in the training group was 2.08 injuries/1000 player-hours, and in the control group 3.35 injuries/1000 player-hours. Based on Poisson regression analysis, adjusted for clustering by team and covariates, the incidence rate ratios (IRR) for all injuries and acute onset injury were 0.62 (95% CI 0.39 to 0.99) and 0.57 (95% CI 0.35 to 0.91). Point estimates also suggest protection of lower extremity, ankle and knee sprain injuries (IRR=0.68 (95% CI 0.42 to 1.11), IRR=0.5 (95% CI 0.24 to 1.04) and IRR=0.38 (95% CI 0.08 to 1.75)).

Conclusions A neuromuscular training programme is protective of all injuries and acute onset injury in youth soccer players.

INTRODUCTION

Soccer is the world's most popular organised sport with an estimated 265 million players participating worldwide in 2006.[1] This is a 10% increase in participation since 2000, with the greatest increases seen in youth (32%).[1] In 2007, over 700 000 youth were registered in the Canadian Soccer Association.[2] Soccer is one of the top three sports for participation in adolescents in Calgary and region, with 20% of adolescents reporting soccer as one of their top three sports for participation.[3] With high rates of participation, there is a growing concern regarding the public health significance of soccer-related injuries.

The estimated injury rate in youth soccer in Calgary and region is 30 injuries/100 participants/year or 3–6 injuries/1000 participation hours, with no significant difference in injury rate estimated in the outdoor compared with the indoor soccer season.[3–5] Lower-extremity injuries accounted for 78% of injuries, consistent with other studies (61–81%).[5–12] Ankle injuries were the most common, followed by knee injuries accounting for 28% and 19% of all injuries, consistent with other studies.[4–12] Overall, injury rates are similar for boys and girls; however, girls have a reportedly greater risk of ankle and knee sprain injuries specifically.[4 5] Reduction of injuries in soccer is critical and has significant implications regarding future development of osteoarthritis, participation in physical activity and future morbidity and mortality related to physical activity. There is evidence that knee and ankle injuries result in an increased risk of early-onset osteoarthritis.[13–17]

Currently, there is no standard of care for evidence-based injury-prevention strategies in community youth soccer. Studies examining the effectiveness of soccer-specific injury-prevention programmes primarily evaluate multifaceted training programmes including components of balance, neuromuscular control, agility and strength.[18–29] Most studies demonstrated a protective effect of the intervention with a 32–88% reduction in injury.[12 18–22 24 26 28] The majority of these studies targeted prevention of all injury.[12 20–22 25–27] Other studies targeted specific injury types including anterior cruciate ligament (ACL) injury,[23] hamstring strains[18] and ankle sprains.[28] Five of the intervention studies have focused on female youth soccer players,[22–24 26 27] and only one study targeted male youth soccer players.[12] Only two of these are randomised controlled trials (RCT).[26 27]

The objective of this cluster-RCT was to examine the effectiveness of a neuromuscular training programme, including a wobble board balance training component, in reducing injury in male and female youth community soccer.

METHODS

Design

This was a cluster RCT.

Subjects

The sampling frame consisted of 82 youth soccer teams from 12 Calgary soccer clubs playing

Downloaded from bjsm.bmj.com on November 17, 2012 - Published by group.bmj.com

in the 2006/2007 indoor season. The 12 clubs were selected for participation based on having more than one team in tiers 1 or 2, boys and girls, U13–U18, where injury rates have previously been shown to be the highest.[5] All players were recruited at the beginning of the preseason in October 2006, as soon as teams were formed. Inclusion criteria were adolescent between the ages of 13 and 18, participating in a Calgary Minor Soccer Association Club team at the commencement of the 2006/2007 indoor soccer season (tiers 1 or 2, U13–U18, male or female). Exclusion criteria included an injury within 6 weeks, history of systemic disease (eg, cancer, arthritis, heart disease) or neurological disorder (ie, head injury, cerebral palsy) which prevented full participation at the commencement of the 2006/2007 indoor season. Consent was requested from the club president followed by the team coach, prior to recruiting players. An informed consent form, as outlined by the University of Calgary, Office of Medical Bioethics, was signed by all study participants and their parent or guardian prior to testing.

A sample size of 1008 players (504 per study group, 36 teams, 14 players) was estimated for a minimally important incidence rate ratios (IRR) of 0.675 or greater based on expected injury rate of 30 injuries/100 players in the control group, adjusting for cluster and an anticipated drop-out rate of 5% (α=0.05, β=0.2, intracluster correlation coefficient (ICC)=0.08).[5] The teams were randomised by club. All teams playing in the designated age and tier categories were invited to participate. Randomisation was revealed following recruitment of teams to ensure allocation concealment.

Procedures

Injury surveillance included a baseline medical questionnaire, baseline assessment, daily participation exposure data and injury report forms (IRFs) previously validated in adolescent community soccer.[4] Each player completed a baseline medical questionnaire and informed consent. Baseline assessment included height (m), weight (kg), leg dominance based on kicking a soccer ball and a timed dynamic eyes closed unipedal balance test on an Airex Balance Pad. This measurement was previously examined and found to be reliable in adolescents.[30]

Each team was assigned a team designate (ie, team trainer, coach or manager) to record all daily participation exposure data on a weekly exposure sheet (WES) for every game and practice. In the event of a soccer injury, the player was given an injury identification number that was also included on the IRF. Completion of 15-min warm-up was also tracked on the WES. A home journal was given to each study participant to record weekly completion of home training programme.

A study therapist (physiotherapist or athletic therapist) blinded to study group allocation was on-site at the two Calgary indoor soccer venues for all games. All injuries occurring during soccer were directed by the team designate to the study therapist for assessment immediately if a game injury occurred. Any injury occurring off site (ie, training session/practice) was assessed by the study therapist within 1 week. The study therapist completed the IRF. The injury definition included any soccer injury requiring medical attention and/or removal from a session and/or resulting in time-loss from full soccer team participation. Follow-up including the details of all medical follow-up for any injury was done by the research coordinator by telephone. A player was referred to a sport medicine physician at the University of Calgary or their family physician for any injury resulting in time-loss of more than 1 week.

The study period was 1 year. The soccer season was 20 weeks long (October 2006–March 2007). Telephone follow-up continued until October 2007 for all injuries sustained which had not previously resolved to allow full return to sport.

Intervention

A coach-delivered warm-up routine was taught to the players and coaches in both groups by a physiotherapist, following baseline assessment and treatment group allocation (online appendix 1). The programme was reviewed by the physiotherapist at 3 weeks and 6 weeks following initial teaching session. A ‘standard of practice’ 15 min warm-up routine including aerobic, static stretching and dynamic stretching components was taught to the control group. The training group was taught a 15 min warm-up including the same aerobic and dynamic stretch components to be completed in 5 min, in addition to 10 min of neuromuscular training components (ie, strength, agility, balance) and a 15-min home-based balance training programme (using a 16 inch diameter wobble board provided by Fitter First) which has been effective in preventing injuries in other youth populations.[31,32] The neuromuscular training components were selected from programmes previously used to reduce injury risk in youth soccer, basketball, handball and physical education (see figures 1 to 6).[23,24,26,31–33] The control group was given a home-programme, including only the stretching components. Teams were blinded to the details of the other study-group programmes.

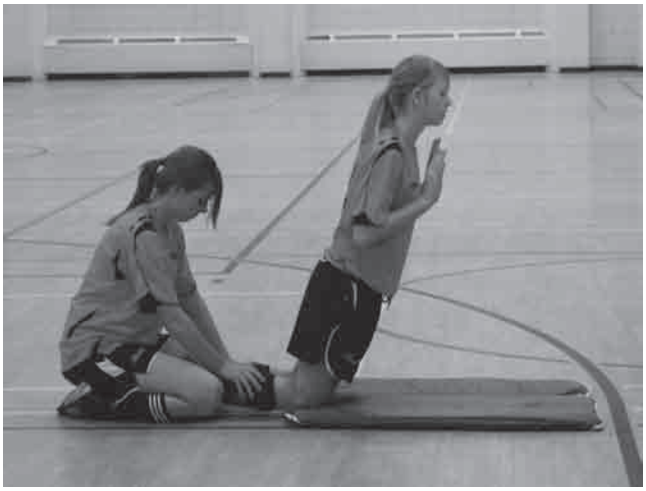

Figure 1 Eccentric hamstring exercise.

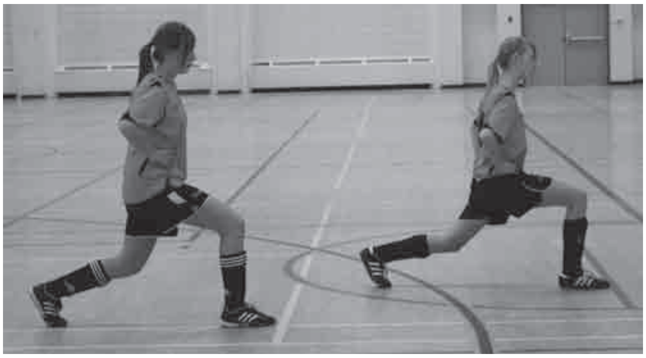

Figure 2 Walking lunges eccentric quadriceps exercise.

Br J Sports Med 2010;**44**:555–562. doi:10.1136/bjsm.2010.074377

Downloaded from bjsm.bmj.com on November 17, 2012 - Published by group.bmj.com

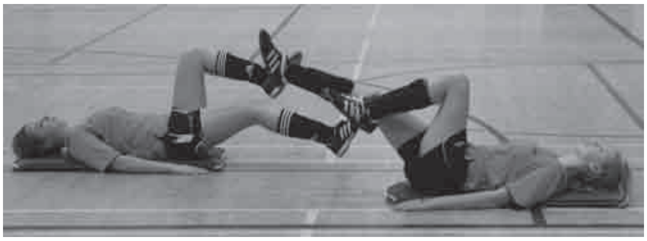

Figure 3 Core stability abdominal strength exercise.

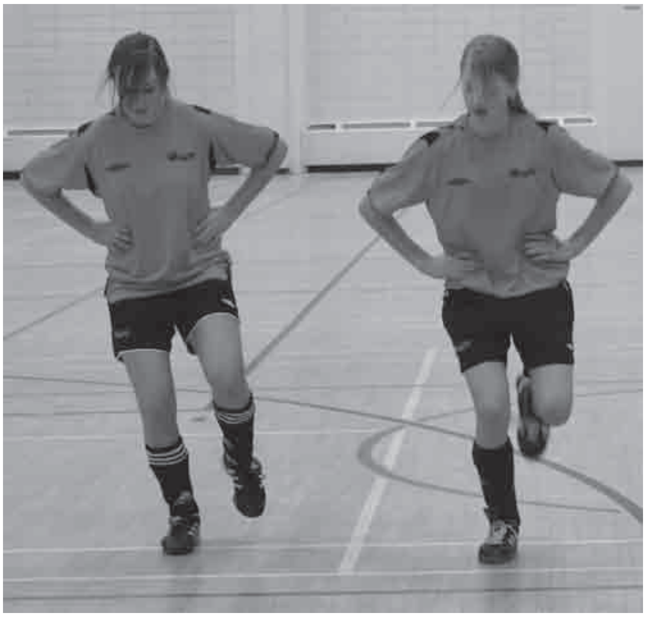

Figure 4 Single leg jump training: focus on alignment and core stability.

This soccer-specific neuromuscular training programme was pilot tested with 20 soccer players (U16) over 6 weeks in April–May 2006.[34] Excellent aderence to the programme was reported (85% completing programme three times per week). A significant improvement in dynamic balance was also demonstrated in those players reporting adherence to the programme at least three times per week (t=2.33, p=0.04).[34]

Statistical analysis

Stata (Version 9.0; Stata, College Station, Texas) was used for all statistical analyses.[35] Descriptive statistics are reported for baseline characteristics which were compared across the study groups. Injury rates (number of injuries/1000 player-hours) and 95% CI were estimated. An intent to treat multivariate Poisson regression analysis (with player hours included as an offset, adjustment for clustering by team and important covariates (ie, previous injury, age group, gender)) was used to estimate the IRRs for all injury in the training group compared with the control group. All previously described potential risk factors (ie, previous injury, age group, gender) were included as potential covariates for inclusion, but only those that were at the 5% level of significance were included in the final model. Two-way interactions were examined for evidence of intervention effect modification. IRRs associated with acute-onset injury, lower-extremity injury, ankle-sprain injury and knee-sprain injury were also estimated using the same methods.

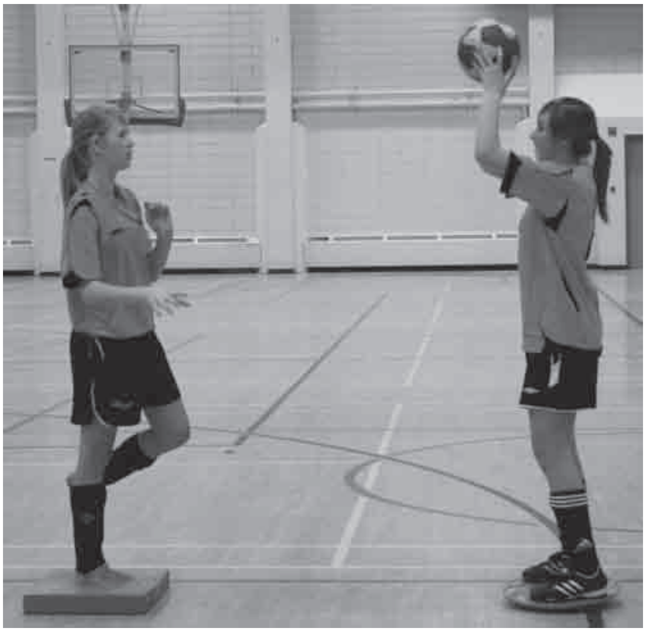

Figure 5 Team-based balance exercise.

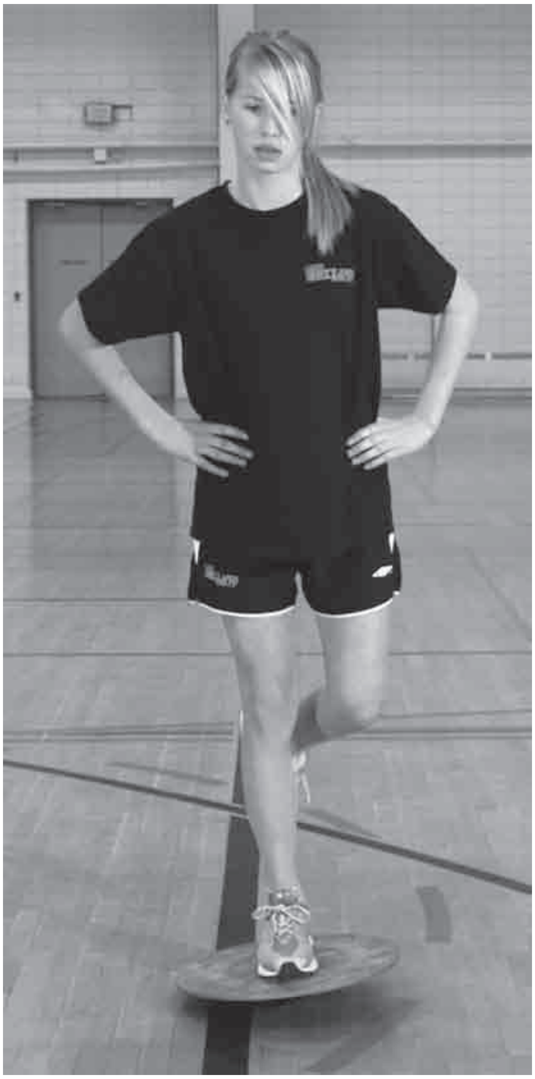

Figure 6 Balance board exercise.

Downloaded from bjsm.bmj.com on November 17, 2012 - Published by group.bmj.com

RESULTS

All 82 eligible teams were asked to participate in this study. Initially, 12 coaches declined participation in the study, and 70 teams were randomised by club to the training and control groups. Ten teams declined participation following on-site recruitment efforts with the players. Players from 60 teams completed the study (32 training group teams (n=380 players), 28 control group teams (n=364 players)). The selection and allocation of players is summarised in figure 7. Baseline characteristics by group are summarised in table 1. With the exception of gender distribution, there were no clinically relevant differences found between study groups on baseline characteristics.

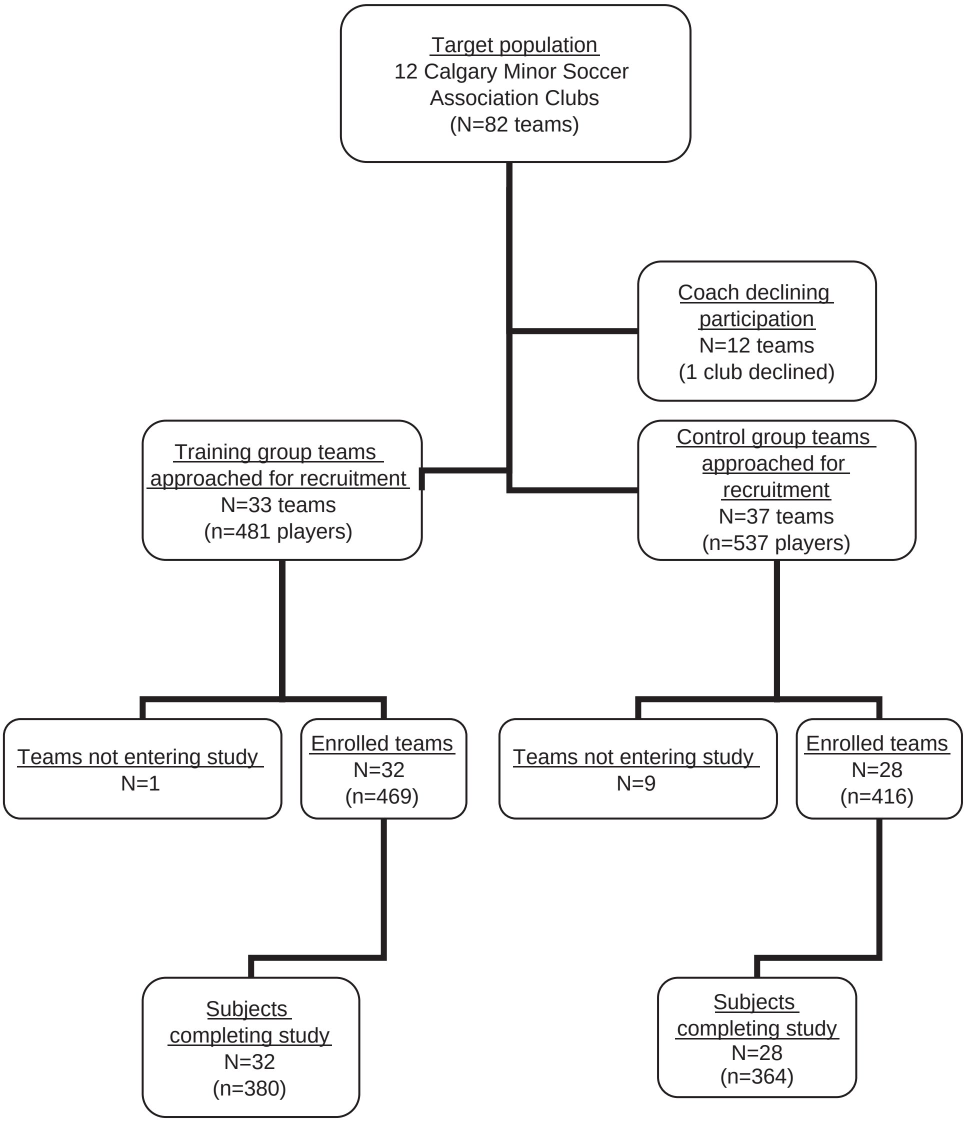

Figure 7 Flow chart of players through the study.

Table 1 Baseline characteristics

Baseline characteristic	Training group (n=380)	Control group (n=364)
Age group	U13–15=46.6% (95% CI 41.5 to 51.7)<br>U16–18=53.4% (95% CI 48.3 to 58.5)	U13–15=48.9% (95% CI 43.7 to 54.2)<br>U16–18=51.1% (95% CI 45.8 to 56.3
Gender	Female=42.4% (95% CI 37.3 to 47.5)<br>Male=57.6% (95% CI 52.5 to 62.7)	Female=69% (95% CI 63.9 to 73.7)<br>Male=31% (95% CI 26.3 to 36.1)
Previous injury 1 year	40.5% (95% CI 35.6 to 45.7)	41.8% (95% CI 36.6 to 47)
Height (m)	1.66 (95% CI 1.64 to 1.67)	1.63 (95% CI 1.61 to 1.64)
Weight (kg)	55.2 (95% CI 54.2 to 56.3)	53.8 (95% CI 52.6 to 55.0)
Body mass index (kg/m^2)	19.9 (95% CI 19.6 to 20.2)	20.4 (95% CI 19.8 to 20.9)
Left foot dominant	7.1% (95% CI 4.7 to 10.2)	6% (95% CI 3.8 to 9.0)
Dynamic balance max Left (s)	6.08 (95% CI 5.64 to 6.51)	6.51 (95% CI 5.91 to 7.11)
Dynamic balance max Right (s)	6.51 (95% CI 6.09 to 6.94)	6.44 (95% CI 5.87 to 7.02)

Downloaded from bjsm.bmj.com on November 17, 2012 - Published by group.bmj.com

There were a total of 129 injuries, 50 in the training group (n=380) and 79 in the control group (n=364). The overall injury rate in the training group was 2.08 injuries/1000 player-hours (95% CI 1.54 to 2.74) or 13.16 injuries/100 players (95% CI 9.93 to 16.98) compared with the injury rate in the control group which was 3.35 injuries/1000 player-hours (95% CI 2.65 to 4.17) or 21.7 injuries/100 players (95% CI 17.58 to 26.3) in the 20-week indoor season. In the training group, 46 players sustained one injury, and two players sustained two injuries. In the control group, 63 players sustained one injury, five players sustained two injuries, and two players sustained three injuries. Injury rates based on overall injury, acute onset injury, lower-extremity injury, ankle sprain injury and knee sprain injury are summarised in table 2.

Exposure hours for the training group were 24 051 (n=380), and for the control group 23 597 (n=364). Weekly exposure (WES) data were missing for 4/28 (14.29%) control group teams and 6/32 (18.75%) training group teams. Exposure hours were estimated for these teams based on weekly exposure data collected from other teams by age group and gender.

Comparisons of injury rates (number of injuries/1000 player hours) by injury type based on Poisson regression analysis are summarised in figure 8. IRRs associated with study group and significant covariates are summarised with 95% CI based on a cluster-adjusted Poisson regression analysis in table 3. There was a 38% reduction in all injury in the training group compared with the control group and a 43% reduction in acute-onset injury. Point estimates suggest a trend towards a reduction in lower-extremity injury, ankle sprain injuries and knee sprain injuries, but these were not statistically significant findings. All analyses included adjustment for significant covariates including previous injury and age

Table 2 Incidence rate ratios by injury type based on Poisson regression analysis adjusted for cluster and covariates (previous injury and age group)

Study group injury type	No of players	No of player hours	No of injuries	Injury rate (no of injuries/1000 player-hours) (95% CI)	Incidence rate ratio (cluster-adjusted and adjusted for covariates) (95% CI)	p Value
All injury						
Control	364	23 597	79	3.35 (2.65 to 4.17)	0.62 (0.39 to 0.99)	0.045*
Training	380	24 051	50	2.08 (1.54 to 2.74)		
Acute injury						
Control	364	23 597	72	3.05 (2.39 to 3.84)	0.57 (0.35 to 0.91)	0.018*
Training	380	24 051	42	1.75 (1.26 to 2.34)		
Lower-extremity injury						
Control	364	23 597	60	2.54 (1.94 to 3.27)	0.68 (0.42 to 1.11)	0.126
Training	380	24 051	42	1.75 (1.26 to 2.36)		
Ankle sprain						
Control	364	23 597	27	1.14 (0.75 to 1.66)	0.5 (0.24 to 1.04)	0.065
Training	380	24 051	14	0.58 (0.32 to 0.98)		
Knee sprain						
Control	364	23 597	8	0.34 (0.15 to 0.67)	0.38 (0.08 to 1.75)	0.232
Training	380	24 051	3	0.12 (0.03 to 0.36)		

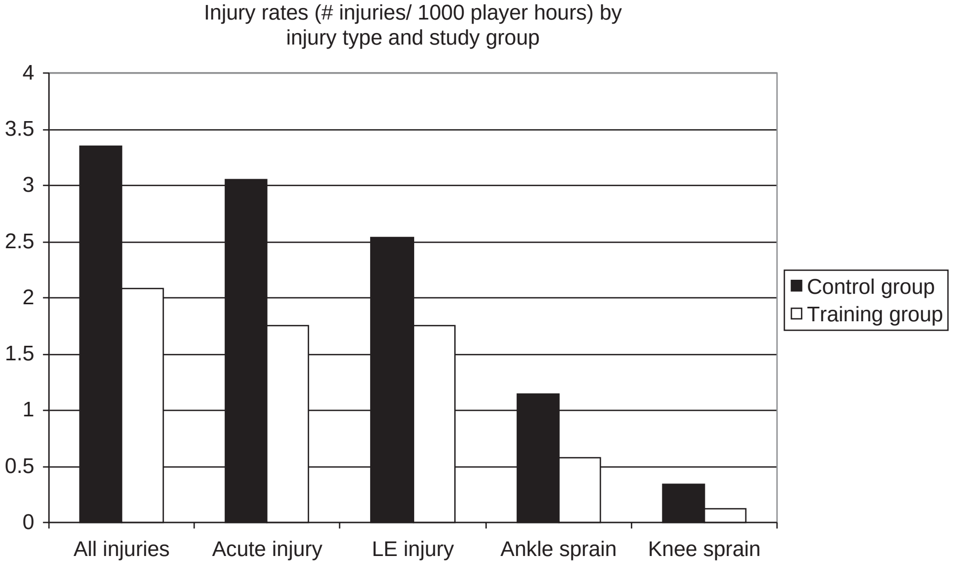

Figure 8 Injury rates by study group for all injury definitions. LE, lower extremity.

Downloaded from bjsm.bmj.com on November 17, 2012 - Published by group.bmj.com

Table 3 Analysis of risk factors for all injury types based on Poisson regression analysis adjusted for clustering by team and covariates (previous injury and age group)

	Injury type				
	All injury	Acute onset injury	Lower-extremity injury	Ankle sprain injury	Knee sprain injury
Covariates	Adjusted incidence rate ratio (95% CI)	Adjusted incidence rate ratio (95% CI)	Adjusted incidence rate ratio (95% CI)	Adjusted incidence rate ratio (95% CI)	Adjusted incidence rate ratio (95% CI)
Study group					
Training	0.62 (0.39 to 0.99)*	0.57 (0.35 to 0.91)*	0.68 (0.42 to 1.11)	0.5 (0.24 to 1.4)	0.38 (0.08 to 1.75)
Control	1	1	1	1	1
Previous injury					
Yes	2.15 (1.45 to 3.19)*	2.15 (1.41 to 3.26)*	1.88 (1.22 to 2.9)*	2.29 (1.23 to 4.28)*	3.63 (1.24 to 10.58)*
No	1	1	1	1	1
Age group					
U15–U18	1.7 (1.07 to 2.7)*	1.85 (1.18 to 2.9)*	1.54 (0.94 to 2.52)	2.62 (1.14 to 6.0)*	1.29 (0.37 to 4.4)
U13–U15	1	1	1	1	1

*Significant based on p<0.05.

group for all injury definitions. Independent of study group, there was a greater risk of injury in players reporting a previous injury within 1 year for all injury definitions and players in the U16–U18 age group compared with the U13–U15 age group for all injury, acute-onset and ankle-sprain injury. Gender was not a significant risk factor for any injury definition; however, point estimates suggest a greater risk for females for ankle sprain (IRR=1.86 (95% CI 0.72 to 4.8)) and knee sprain (IRR=2.54 (95% CI 0.25 to 28.53)) injury. There was no evidence effect modification for any injury definition.

Acute onset injuries accounted for 42/50 (84% (95% CI 70.89 to 92.83)) injuries in the training group and 72/79 (91.14% (95% CI 82.59 to 96.36)) injuries in the control group. The remaining injuries were classified as gradual-onset injuries. The most frequently occurring specific injury type was ankle sprain, accounting for 14/50 (28% (95% CI 16.23 to 42.49)) injuries in the training group and 27/79 (34.18% (95% CI 23.81 to 45.71)) injuries in the control group.

Injury severity was classified based on the consensus agreement for injury definitions as slight, minimal or mild (0–7 days' time-loss from soccer), moderate (8–28 days' time-loss from soccer), or severe (>28 days' time-loss from soccer).[36] The proportions of moderate and severe injuries in the training group were: moderate 13/50 (26% (95% CI 14.63 to 40.34)) and severe 1/50 (2% (95% CI 0.05 to 10.64)). The proportions of injuries in the training group were: moderate 13/79 (16.46% (95% CI 9.06 to 26.49)) and severe 5/79 (6.33% (95% CI 2.09 to 14.16)). The proportion of injuries by injury severity did not differ by training group.

Return of self-report adherence to home-programme was poor (<15%). For players returning home-programme journals, the median number of sessions reported was 23 (range 2–26). For all teams completing weekly exposure data, completion of warm-up was indicated for every practice and game. However, we cannot be certain that all components of prescribed warm-up were completed for each session.

DISCUSSION

The objective of this study was to examine the effectiveness of a neuromuscular training warm-up programme in reducing injury rates in community soccer. This training programme was effective in reducing the risk of all injury (IRR=0.62 (95% CI 0.39 to 0.99)) and acute-onset injuries (IRR=0.57 (95% CI 0.35 to 0.91)) in the highest 2/4 divisions of play. The protective effect of the neuromuscular training programme in reducing lower-extremity injury, ankle sprain injury and knee sprain injury is arguably clinically relevant, despite a lack of statistical significance.

The observed injury rate in the control group in our study was 3.35 injuries/1000 player-hours (95% CI 2.65 to 4.17). This is consistent with a previous cohort study in indoor soccer in Calgary where the injury rate was 4.45 injuries/1000 player-hours (95% CI 31. to 6.99).[5] Lower-extremity injuries accounted for the majority of soccer related injuries (75.9%) in the control group, consistent with other studies (61–81%).[4–12] Ankle sprain injuries were the most common type of injury accounting for 34.18% of all soccer injuries in the control group, consistent with other studies.[4–12] Independent of study group, players who report an injury in the previous year had a greater risk of injury than those who did not. These results are supported by many other studies that suggest previous injury is a major risk factor for injury in youth soccer.[5 28 37 38] Consistent also with other studies, higher rates of injury were found in the older age groups.[4 39 40] In this study, the rates of all injury, acute injury and lower-extremity injury did not differ by gender, consistent with other studies.[4 5 41 42] However, though not statistically significant, there was some suggestion of greater risk of ankle and knee sprain injuries in females. This is consistent with other studies reporting a higher risk of ligament sprain and ACL injuries in female players.[4 5 43 44]

Studies examining the effectiveness of soccer-specific injury prevention programmes primarily evaluate multifaceted training programmes including components of balance, neuromuscular control, agility and strength.[18–28] Our findings are consistent with other studies demonstrating a protective effect of the intervention (32–88% reduction in injury).[12 18–22 24 26 28] The exceptions are three studies which demonstrated no significant effect of intervention.[23 25 27] Poor adherence to the intervention may be the key factor in studies showing no effect. In the case of Hewett *et al*,[23] the outcome measure was serious knee injury. There were only 14 injuries, limiting the power to detect a significant difference, but a significant trend was observed. A RCT design was used in only two studies examining female youth soccer players.[26 27] Despite the context of a team intervention, only two studies adjusted clustering by team appropriately in the analysis.[26 27] The precision of estimates of effect in the other studies should thus be interpreted with caution.[45] In this study, we examined the

Downloaded from bjsm.bmj.com on November 17, 2012 - Published by group.bmj.com

effectiveness of a neuromuscular training programme in both male and female youth soccer players using a cluster-RCT and considering clustering by team in the design and analysis.

Limitations

There was a greater non-participation of teams in the control group compared with the training group. Teams did not participate, however, primarily based on a coach decision which was related to the inability to identify a team designate or the lack of interest in additional time commitment to warm-up activities. Non-participating teams represented teams across all clubs, age groups and genders. It is possible, however, that team coaches choosing not to participate may be less aware of injury prevention and safety in general. If this is the case, it is possible that these teams may also have higher injury rates, in which case the effect estimates estimated in this study are underestimated, as non-participating teams were primarily in the control group.

We found no difference in the effectiveness of the programme in males and females. However, an uneven distribution of male and female teams in the two study groups limited the power to examine effect modification.

Weekly exposure data were imputed for 4/28 (14.29%) control group teams and 6/32 (18.75%) training group teams. Given the relatively small proportion of weeks imputed did not differ significantly between study groups, there is minimal concern regarding the accuracy or precision of our study estimates.

Another weakness of this study was the inability to examine a dose–response effect of the home-based training programme based on the return of <15% of home-programme journals. In addition, while we know that there was reported participation in the team warm-up, completion of all components at each session remains unknown.

Strengths

This is the first single-blinded RCT to examine the effectiveness of a neuromuscular training programme in both male and female youth soccer players, using valid injury surveillance, and including an appropriate cluster-adjusted analysis. The actual ICC was 0.05 (95% CI 0.003 to 0.09). As such, the sample size (based on estimated ICC=0.08) was adequate to detect a protective effect of the programme despite underachieving the estimated sample size. Based on an intent-to-treat analysis, a significant effect was seen in reducing the risk of all injury and acute-onset injury.

CONCLUSIONS

A team-based neuromuscular training warm-up and home-based balance training programme was protective of all injury and acute-onset injuries in community youth indoor soccer players. It is recommended that adolescent soccer teams U13–U18 participate in a neuromuscular training programme including a balance training component to reduce the risk of injury in soccer. Future research will include evaluation of an individually targeted approach to preseason screening and prevention of injury in youth soccer to target the players at highest risk of injury in addition to a team-based strategy. Further examination of implementation methods and adherence is essential to maximise the prevention of injury in youth soccer. Future research should include long-term follow-up of sport injury in adolescents, examining levels of physical activity and early-onset osteoarthritis.

What is already known on this topic

- The overall injury rate in youth soccer is high and similar for male and female players.
- Females have a higher risk of ankle and knee sprain injuries in youth soccer.
- Studies evaluating the effectiveness of a multifaceted neuromuscular training programme in soccer demonstrate a reduction in injury risk between 32% and 88% in elite players and female youth players; however, few of these are randomised controlled trials, and clustering is rarely accounted for in the analysis.

What this study adds

- A soccer-specific neuromuscular training programme, including a home-based wobble board component, can reduce the risk of injury by over one-third in youth indoor soccer players.
- There is less conclusive evidence suggesting such a programme specifically reduces the risk of ankle and knee sprain injuries.

Acknowledgements The authors acknowledge the Calgary Minor Soccer Association Clubs, coaches, parents and players. Without their support, this research would not have been possible. The authors would like to thank Fitter International for their shared vision in the prevention of injury in adolescent sport. Their financial support in providing wobble boards, at a significantly reduced cost, also made this research possible. The authors also acknowledge Jamie McInnis and Jenelle McAllister (research coordinators) for their assistance with this project.

Funding Alberta Heritage Foundation for Medical Research. CAE is supported by an Alberta Heritage Foundation for Medical Research Population Health Investigator Award, a Canadian Institutes of Health Research New Investigator Award and a Professorship in Pediatric Rehabilitation funding by the Alberta Children's Hospital Foundation through the Research Institute for Child and Maternal Health, University of Calgary.

Competing interests None.

Patient consent Obtained from the parents.

Ethics approval Ethics approval was provided by the University of Calgary, Office of Medical Bioethics.

Provenance and peer review Not commissioned; externally peer reviewed.

REFERENCES

1. **FIFA**. (2007) FIFA big count 2006: 270 million people active in football. http://www.fifa.com/aboutfifa/media/newsid=529882.html (Accessed 29 March 2010).
2. **Canadian Soccer Association** (CSA) (2007): 2007 Demographics. Player registration. http://www.canadasoccer.com/documents/demographics/2007_demographics _ report.pdf (Accessed 29 March 2008).
3. **Emery CA**, Meeuwisse WH, McAllister JR. Survey of sport participation and sport injury in Calgary and area high schools. *Clin J Sport Med* 2006;**16**:20–6.
4. **Emery CA**, Meeuwisse WH, Hartmann S. Risk factors for injury in adolescent soccer. Pilot implementation and validation of an injury surveillance system. *Am J Sport Med* 2005;**1882–1891**:33.
5. **Emery CA**, Meeuwisse WH. A comparison of risk factors for injury in indoor and outdoor soccer. *Am J Sport Med* 2006;**34**:1636–42.
6. **Kibler WB**. Injuries in adolescent and preadolescent soccer players. *Med Sci Sports Exerc* 1993;**25**:1330–2.
7. **Schmidt-Olsen S**, Jørgensen U, Kaalund S, *et al.* Injuries among young soccer players. *Am J Sports Med* 1991;**19**:273–5.

Downloaded from bjsm.bmj.com on November 17, 2012 - Published by group.bmj.com

8. **Söderman K**, Adolphson J, Lorentzon R, *et al.* Injuries in adolescent female players in European football: a prospective study over one outdoor soccer season. *Scand J Med Sci Sports* 2001;**11**:299–304.
9. **Maehlum S**, Daljord O, Hansen KJ. Frequency of injuries in youth soccer. *Med Sci Sports* 1991;**31**:S401.
10. **Peterson L**, Junge A, Chomiak J, *et al.* Incidence of football injuries and complaints in different age groups and skill level groups. *Am J of Sports Med* 2000;**28**:S51–7.
11. **Schmidt-Olsen S**, Bunemann LK, Lade V, *et al*. Soccer injuries of youth. *Br J Sport Med* 1985;**19**:161–4.
12. **Junge A**, Rösch D, Peterson L, *et al.* Prevention of soccer injuries: a prospective intervention study in youth amateur players. *Am J Sports Med* 2002;**30**:652–9.
13. **Gillquist J**, Messner K. Anterior cruciate ligament reconstruction and the long-term incidence of gonarthrosis. *Sports Med* 1999;**27**:143–56.
14. **Daniel DM**, Stone ML, Dobson BE, *et al.* Fate of the ACL-injured patient. A prospective outcome study. *Am J Sports Med* 1994;**22**:632–44.
15. **Drawer S**, Fuller CW. Propensity for osteoarthritis and lower limb joint pain in retired professional soccer players. *Br J Sports Med* 2001;**35**:402–8.
16. **Roos EW**. Joint injury causes osteoarthritis in young adults. *Curr Opin Rheumatol* 2005;**17**:195–200.
17. **von Porat A**, Roos EM, Roos H. High prevalence of osteoarthritis 14 years after an anterior cruciate ligament tear in male soccer players: a study of radiographic and patient relevant outcomes. *Ann Rheum Dis* 2004;**63**:269–73.
18. **Askling C**, Karlsson J, Thorstensson A. Hamstring injury occurrence in elite soccer players after preseason strength training with eccentric overload. *Scand J Med Sci Sports* 2003;**13**:244–50.
19. **Caraffa A**, Cerulli G, Projetti M, *et al.* Prevention of anterior cruciate ligament injuries in soccer. A prospective controlled study of proprioceptive training. *Knee Surg Sports Traumatol Arthrosc* 1996;**4**:19–21.
20. **Ekstrand J**, Gillquist J, Liljedahl SO. Prevention of soccer injuries. Supervision by doctor and physiotherapist. *Am J Sports Med* 1983;**11**:116–20.
21. **Engebretsen AH**, Myklebust G, Holme I, *et al.* Prevention of injuries among male soccer players: a prospective, randomized intervention study targeting players with previous injuries or reduced function. *Am J Sports Med* 2008;**36**:1052–60.
22. **Heidt RS** Jr, Sweeterman LM, Carlonas RL, *et al.* Avoidance of soccer injuries with preseason conditioning. *Am J Sports Med* 2000;**28**:659–62.
23. **Hewett TE**, Lindenfeld TN, Riccobene JV, *et al.* The effect of neuromuscular training on the incidence of knee injury in female athletes. A prospective study. *Am J Sports Med* 1999;**27**:699–706.
24. **Mandelbaum BR**, Silvers HJ, Watanabe DS, *et al.* Effectiveness of a neuromuscular and proprioceptive training program in preventing anterior cruciate ligament injuries in female athletes: 2-year follow-up. *Am J Sports Med* 2005;**33**:1003–10.
25. **Söderman K**, Werner S, Pietilä T, *et al.* Balance board training: prevention of traumatic injuries of the lower extremities in female soccer players? A prospective randomized intervention study. *Knee Surg Sports Traumatol Arthrosc* 2000;**8**:356–63.
26. **Soligard T**, Myklebust G, Steffen K, *et al*. Comprehensive warm-up programme to prevent injuries in young female footballers; cluster randomized controlled trial. *Br J Sport Med* 2008;**337**:a2469.
27. **Steffen K**. Introduction. In: Steffen K, ed. *Injuries in female youth football. Prevention performance and risk factors. (Dissertation from the Norwegian School of Sport Sciences.)* Oslo: Oslo Sport Trauma Research Centre, 2008:1–30.
28. **Tropp H**, Askling C, Gillquist J. Prevention of ankle sprains. *Am J Sports Med* 1985;**13**:259–62.
29. **Emery CA**. Considering cluster analysis in sport medicine and injury prevention research. *Clin J Sport Med* 2007;**17**:211–14.
30. **Emery C**, Cassidy D, Klassen T, *et al*. The reliability of a static and dynamic timed balance measure in healthy adolescents. *Phys Ther* 2005;**85**:502–14.
31. **Emery CA**, Cassidy JD, Klassen TP, *et al.* Effectiveness of a home-based balance-training program in reducing sports-related injuries among healthy adolescents: a cluster randomized controlled trial. *CMAJ* 2005;**172**:749–54.
32. **Emery CA**, Rose MS, Meeuwisse WH, *et al*. The effectiveness of an injury prevention strategy in high school basketball. A cluster-randomized controlled trial. *Clin J Sport Med* 2007;**17**:17–24.
33. **Olsen OE**, Myklebust G, Engebretsen L, *et al.* Exercises to prevent lower limb injuries in youth sports: cluster randomised controlled trial. *BMJ* 2005;**330**:449.
34. **Ramage BJ**, Emery CA, Kim SH, *et al*. Does a neuromuscular training strategy, including wobble board training, change lower extremity kinematics among elite adolescent soccer players? *Clin J Sport Med* 2007;**17**:164.
35. **StataCorp**. *Stata statistical software (computer program). Release 9.0*. College Station, Texas, USA: Stata Corporation, 2005.
36. **Fuller CW**, Ekstrand J, Junge A, *et al.* Consensus statement on injury definitions and data collection procedures in studies of football (soccer) injuries. *Clin J Sport Med* 2006;**16**:97–106.
37. **Hägglund M**, Waldén M, Ekstrand J. Previous injury as a risk factor for injury in elite football: a prospective study over two consecutive seasons. *Br J Sports Med* 2006;**40**:767–72.
38. **Kucera KL**, Marshall SW, Kirkendall DT, *et al.* Injury history as a risk factor for incident injury in youth soccer. *Br J Sports Med* 2005;**39**:462.
39. **Inklaar H**, Bol E, Schmikli SL, *et al*. Injuries in male soccer players. Team risk analysis. *Int J Sport Med* 1996;**17**:229–34.
40. **Peterson L**, Junge A, Chomiak J, *et al.* Incidence of football injuries and complaints in different age groups and skill-level groups. *Am J Sports Med* 2000;**28**:S51–7.
41. **LeGall F**, Reilly T, Vandewalle H, *et al.* Incidence of injuries in elite French youth soccer players. *Am J Sport Med* 2006;**34**:928–38.
42. **Nielsen AB**, Yde J. Epidemiology and traumatology of injuries in soccer. *Am J Sports Med* 1989;**17**:803–7.
43. **Arendt E**, Dick R. Knee injury patterns among men and women in collegiate basketball and soccer. NCAA data and review of the literature. *Am J Sport Med* 1995;**23**:694–701.
44. **Mihata LCS**, Beuteur AI, Boden BP. Comparing the incidence of anterior cruciate ligament injury in collegiate lacrosse, soccer, and basketball players. *Am J Sport Med* 2006;**34**:899–904.

Downloaded from bjsm.bmj.com on November 17, 2012 - Published by group.bmj.com

The effectiveness of a neuromuscular prevention strategy to reduce injuries in youth soccer: a cluster-randomised controlled trial

C A Emery and W H Meeuwisse

Br J Sports Med 2010 44: 555-562
doi: 10.1136/bjsm.2010.074377

Updated information and services can be found at:
http://bjsm.bmj.com/content/44/8/555.full.html

These include:

Data Supplement *"Web Only Data"*
http://bjsm.bmj.com/content/suppl/2011/05/23/44.8.555.DC1.html

References This article cites 40 articles, 22 of which can be accessed free at:
http://bjsm.bmj.com/content/44/8/555.full.html#ref-list-1

Article cited in:
http://bjsm.bmj.com/content/44/8/555.full.html#related-urls

Email alerting service Receive free email alerts when new articles cite this article. Sign up in the box at the top right corner of the online article.

Topic Collections Articles on similar topics can be found in the following collections

Football (soccer) (172 articles)
Injury (659 articles)
Knee injuries (59 articles)
Trauma (603 articles)

Notes

To request permissions go to:
http://group.bmj.com/group/rights-licensing/permissions

To order reprints go to:
http://journals.bmj.com/cgi/reprintform

To subscribe to BMJ go to:
http://group.bmj.com/subscribe/
