# Supplementary material for: Comparative Effectiveness of Multi-Component, Exercise-Based Interventions for Preventing Soccer-Related Musculoskeletal Injuries: A Systematic Review and Meta-Analysis
Source: Healthcare (Basel). 2025 Mar 29;13(7):765. doi: 10.3390/healthcare13070765 (PMC11988859; doi:10.3390/healthcare13070765)
Supplement: Supplementary file 1 [file healthcare-13-00765-s001.zip › Additional material Included literature/Gilchrist2008.pdf]

A Randomized Controlled Trial to Prevent Noncontact Anterior Cruciate Ligament Injury in Female Collegiate Soccer Players

Julie Gilchrist,*[†] MD, Bert R. Mandelbaum,[‡] MD, Heidi Melancon,[§] MPH, George W. Ryan,[||] PhD, Holly J. Silvers,[‡] MPT, Letha Y. Griffin,[¶] MD, PhD, Diane S. Watanabe,[‡] MA, ATC, Randall W. Dick,[#] MS, and Jiri Dvorak,** MD

*From the [†]Division of Unintentional Injury Prevention, National Center for Injury Prevention & Control, Centers for Disease Control & Prevention, Atlanta, Georgia, [‡]Santa Monica Orthopedic & Sports Medicine Research Foundation, Santa Monica, California, the [§]National Recreation and Park Association, Ashburn, Virginia, the [||]Office of Statistics and Programming, National Center for Injury Prevention & Control, Centers for Disease Control & Prevention, Atlanta, Georgia, [¶]Peachtree Orthopedics, Atlanta, Georgia, the [#]National Collegiate Athletic Association, Indianapolis, Indiana, and the **Fédération Internationale de Football Association (FIFA), Medical Assessment and Research Center, Schulthess Clinic, Zurich, Switzerland.*

Background: Neuromuscular and proprioceptive training programs can decrease noncontact anterior cruciate ligament injuries; however, they may be difficult to implement within an entire team or the community at large.

Hypothesis: A simple on-field alternative warm-up program can reduce noncontact ACL injuries.

Study Design: Randomized controlled trial (clustered); Level of evidence, 1.

Methods: Participating National Collegiate Athletic Association Division I women's soccer teams were assigned randomly to intervention or control groups. Intervention teams were asked to perform the program 3 times per week during the fall 2002 season. All teams reported athletes' participation in games and practices and any knee injuries. Injury rates were calculated based on athlete exposures, expressed as rate per 1000 athlete exposures. A z statistic was used for rate ratio comparisons.

Results: Sixty-one teams with 1435 athletes completed the study (852 control athletes; 583 intervention). The overall anterior cruciate ligament injury rate among intervention athletes was 1.7 times less than in control athletes (0.199 vs 0.340; $P = .198$; 41% decrease). Noncontact anterior cruciate ligament injury rate among intervention athletes was 3.3 times less than in control athletes (0.057 vs 0.189; $P = .066$; 70% decrease). No anterior cruciate ligament injuries occurred among intervention athletes during practice versus 6 among control athletes ($P = .014$). Game-related noncontact anterior cruciate ligament injury rates in intervention athletes were reduced by more than half (0.233 vs 0.564; $P = .218$). Intervention athletes with a history of anterior cruciate ligament injury were significantly less likely to suffer another anterior cruciate ligament injury compared with control athletes with a similar history ($P = .046$ for noncontact injuries).

Conclusion: This program, which focuses on neuromuscular control, appears to reduce the risk of anterior cruciate ligament injuries in collegiate female soccer players, especially those with a history of anterior cruciate ligament injury.

Keywords: RCT; ACL; soccer; injuries

*Address correspondence to Julie Gilchrist, MD, CDC/NCIPC, Division of Unintentional Injury Prevention, 4770 Buford Hwy, MS F62, Atlanta, GA 30341 (e-mail: jrg7@cdc.gov).

Presented at the interim meeting of the AOSSM, San Francisco, California, March 2004.

Dr. Mandelbaum, Ms. Silvers, and Ms. Watanabe, employees of Santa Monica Orthopedic and Sports Medicine Research Foundation (SMOSMRF), were involved with the development of the PEP Program under evaluation in this study but have no financial interest in the PEP Program and did not participate in data collection or analysis. Ms. Melancon was employed by the SMOSMRF and participated in data collection and analysis.

The American Journal of Sports Medicine, Vol. 36, No. 8
DOI: 10.1177/0363546508318188
© 2008 American Orthopaedic Society for Sports Medicine

Serious knee injuries such as anterior cruciate ligament (ACL) injuries continue to be of concern to athletes in sports that require jumping and pivoting such as basketball, soccer, volleyball, football, and handball.[7] Female athletes have an increased rate of ligamentous knee injury, especially of the ACL, in comparison with their male counterparts participating in similar activities.[††] The disparity in rates is even more pronounced when noncontact ACL injuries are isolated.[1]

Anterior cruciate ligament injuries often require surgery to repair, months of rehabilitation, and result in an increased risk of degenerative arthritis and other long-term sequelae.[6,9] The high physical, mental, emotional, and economic cost of this severe knee injury has prompted research into risk factors and prevention strategies.

In an effort to guide future research, 2 conferences were held to review the identified risk factors and potential prevention strategies; results suggested that neuromuscular and proprioceptive training programs show promise toward reducing the risk of noncontact ACL injuries in female athletes.[9,10] Studies of such prevention programs in high-risk sports demonstrate a reduction in ACL injury risk (N.D. Griffis et al, unpublished data, 1989).[3,12,13,18–20,22,23] However, many of these programs require special equipment such as balance boards, mats, or ankle discs, and may be difficult for many teams to incorporate into their regular practice activities.

In an effort to design an effective program to prevent noncontact ACL injuries that could readily be used at many levels of play without significant investment in equipment or time, an expert panel was convened by the Santa Monica Orthopedic and Sports Medicine Research Foundation in 1999. This group designed the Prevent injury and Enhance Performance (PEP) Program. This program consists of warm-up, stretching, strengthening, plyometrics, and sport-specific agility exercises to address potential deficits in the strength and neuromuscular coordination of the stabilizing muscles around the knee joint. The emphasis of this program was developed around the premise of optimal biomechanical technique. It was designed as an alternative warm-up to be performed before training sessions to avoid the deleterious effects of athlete fatigue while performing such a program. When physical fatigue is an issue, biomechanical technique may falter, diminishing the proposed neuromuscular benefits of the prescribed exercises.[10] The PEP Program activities can be performed on the soccer field before practice without any additional specialized equipment. An entire soccer team can complete the 19 components in less than 30 minutes. Factors related to the ease of implementation and economical impact were carefully considered during the program's design process to improve compliance and implementation within the community.

An early nonrandomized study among highly competitive 14- to 18-year-old female club soccer players using the program demonstrated promising results.[18] In the first year of study (2000), 1012 girls on 52 intervention teams reported 2 confirmed ACL injuries, while the 1905 girls on 95 teams in the remainder of the league reported 23 confirmed ACL injuries, suggesting an 83% decrease in ACL injuries. In the second year of this nonrandomized pilot (2001), 45 teams with 844 female athletes were enrolled to perform the PEP Program and reported 4 ACL injuries. One hundred twelve age- and skill-matched control teams with 1913 athletes reported 35 ACL injuries, corresponding to a 74% reduction in ACL injuries.

In preparation for a large randomized controlled trial of the PEP Program among National Collegiate Athletic Association (NCAA) Division I soccer teams, a pilot was conducted in 2001 that documented the feasibility of the cluster randomized controlled trial and the usability and appropriateness of the materials and methods (J. Gilchrist et al, unpublished data, 2004). Fourteen teams were invited to participate representing diversity of geography, school size, and competitive success. Feasibility of implementing the program in a randomized controlled design was sufficiently demonstrated during the pilot.

This cluster randomized controlled trial examines whether the use of the alternative warm-up to enhance neuromuscular and proprioceptive control can reduce the number of ACL injuries, specifically noncontact ACL injuries, in NCAA Division I female soccer athletes.

MATERIALS AND METHODS

Study Design and Recruitment

The study was conducted during the fall 2002 NCAA soccer season. During the summer and early fall, the coaches and certified athletic trainers (ATC) of all 273 eligible NCAA Division I women's soccer teams were invited to participate (Figure 1). Division I teams were selected as the study population to ensure the availability of ATC staff with sufficient time and equipment to directly supervise each training session and complete the reporting needs of the study. Fourteen teams were ineligible due to prior participation in the pilot study conducted in the previous year. Teams whose coach, ATC, and athletic director agreed to participate in the study and which received appropriate university Institutional Review Board clearance were assigned randomly to either the intervention or the control group (Figure 1). The Institutional Review Board at the Centers for Disease Control and Prevention (CDC) and relevant universities approved the protocol.

After randomization, each team's ATC provided the athletes an overview of the study and an opportunity to consent to participate. Consenting athletes completed a preparticipation questionnaire to document demographic factors such as history of lower leg injury, age, height, and weight. Intervention teams received a videotape and instruction manual for the alternative warm-up and were asked to complete the warm-up 3 times per week during the regular season. Control teams were asked to perform their customary warm-up; they received all intervention materials after completion of all data collection at the end of the season.

Intervention

The alternative warm-up program under study was the PEP Program, which was used during the pilot[8] and in a

[††]References 1, 2, 4, 5, 8, 11, 14, 16, 17, 21

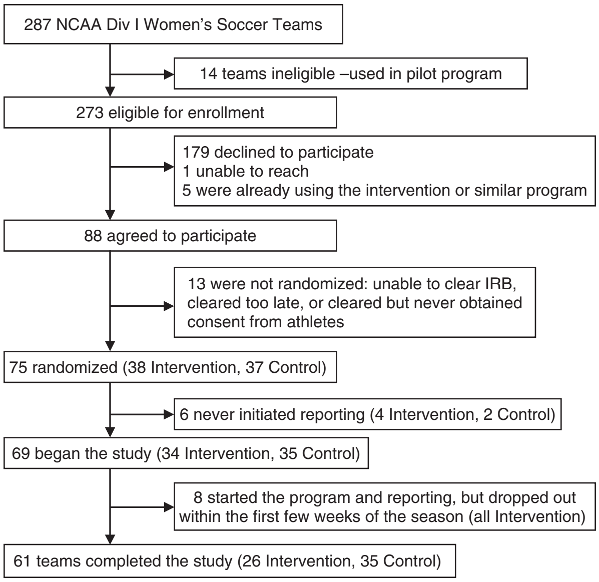

Figure 1. Flow chart of enrollment of 2002 NCAA Division I women's soccer teams.

previously published study[18] involving a 14- to 18-year-old female soccer cohort. The program includes basic components in stretching, strengthening, plyometrics, agilities, and avoidance of high-risk positions depicted on a video that was disseminated to each intervention team.[18] Additionally, teams were provided with replacement exercises to help alleviate boredom with the program (Table 1). The video and supplemental written materials stressed the importance of proper biomechanical technique in completing these exercises and visual examples of proper and improper biomechanical technique for each individual exercise. The coaches and ATCs monitoring the program were asked to emphasize technique and provide direct feedback on technique to the athletes. The program was initiated in August upon the commencement of the fall 2002 soccer season. The program was used for 12 weeks in total, the duration of the regular season schedule.

Outcome Measures

The ATCs of all teams reported each athlete's weekly participation in games and practices (athlete exposures [AE]) and any knee injuries. Participation and injury reports were submitted weekly by facsimile to study staff using codes for both teams and individual athletes for confidentiality. Certified athletic trainers for intervention teams also reported weekly compliance with the program on the participation forms.

A knee injury was defined as an injury to the area about the knee occurring in a game, practice, or conditioning activity that required medical care by ATC or physician, and caused one or more missed days of training. Injury reports collected information on the date of injury, whether it occurred in a practice or game, the timing during the practice or game (first half or second), the structure injured and diagnosis, estimated days lost from practice or play, injury circumstance (contact or noncontact), and a free text description of the incident reported by the ATC and the athlete or other witness. Final diagnoses and days lost were confirmed with the ATCs by phone or e-mail. In this study, an ACL injury was counted only if the ATC reported confirmation by magnetic resonance imaging, arthroscopy, or direct visualization at the time of repair. Classification of an ACL injury as contact or noncontact was based on the ATC's report of the circumstances of the injury using free text. A contact injury was defined as an ACL injury sustained as a result of direct contact to the knee or another body part by another player or object during the course of play. A noncontact injury was defined as an ACL injury sustained by an athlete without extrinsic contact by another player or object on the field.

To assess implementation fidelity in intervention teams and the extent to which similar drills and exercises might be incorporated into the practices of control teams, observational surveys and written surveys were conducted by study staff. Intervention and control teams were paired by proximity. Pairs were clustered geographically by region (eg, Northeast, South, Midwest, and West), and one pair from each region was selected randomly for observation. Two observers visited the 8 teams (4 intervention teams and 4 control teams—13% of teams) twice during the season (early and late in the season). Coaches were contacted regarding the dates, times, and locations of practices during a chosen week; they were aware which practice would be

TABLE 1
Basic Components of the PEP Program[a]

1. Warm-up (50 yards each):
A. Jog line to line of soccer field (cone to cone)
B. Shuttle run (side to side)
C. Backward running
2. Stretching (30 s × 2 reps each):
A. Calf stretch
B. Quadricep stretch
C. Figure 4 hamstring stretch
D. Inner thigh stretch
E. Hip flexor stretch
3. Strengthening:
A. Walking lunges (20 yards × 2 sets)
B. Russian hamstring (3 sets × 10 reps)
C. Single toe-raises (30 reps on each side)
4. Plyometrics (20 reps each):
A. Lateral hops over 2 to 6 inch cone
B. Forward/backward hops over 2 to 6 inch cone
C. Single leg hops over 2 to 6 inch cone
D. Vertical jumps with headers
E. Scissors jump
5. Agilities:
A. Shuttle run with forward/backward running (40 yards)
B. Diagonal runs (40 yards)
C. Bounding run (45–50 yards)

[a]PEP, Prevent injury and Enhance Performance Program; s, seconds; reps, repetitions; SI, sacroiliac; A/P, anterior/posterior. Adapted with permission from the Santa Monica Orthopedic and Sports Medicine Research Foundation. Additional details and supplemental replacement exercises available at www.aclprevent.com.

TABLE 2
Characteristics of Teams and Athletes by Study Group[a]

	Intervention Teams	Control Teams	*P* Value
Team/athlete characteristics			
Number of teams	26	35	—
Number of athletes	583	852	—
Average years in college[b]	2.25	2.28	.663
Average age, y[b]	19.88	19.88	.980
Average height, m[b]	1.66	1.67	.172
Average weight, kg[b]	61.90	62.1	.680
Average BMI[b]	22.36	22.28	.491
History of injuries[b] before study			
Number of athletes with history of knee injury (%)	173 (31.0)	255 (31.1)	.982
Number of athletes with history of ACL rupture (%)	76 (13.6)	100 (12.2)	.432
Exposures during study			
Total AE[c]	35 220	52 919	—
AE in games	8 168	11 843	—
AE in practices	18 370	30 105	—
Average intervention program uses per intervention team (range)	25.8 (12-37)	N/A	—

[a]BMI, body mass index; ACL, anterior cruciate ligament; AE, athlete exposure.
[b]Personal characteristics and injury history were obtained from a preparticipation survey returned by 571 intervention athletes and 834 control athletes.
[c]Total AE includes "other" activities (ie, fitness and conditioning activities performed outside of practice and games). Thus, AE in practices and AE in games will not sum to total AE.

observed but were unaware of the purpose of observation or the data to be collected.

A standardized observational instrument was used. To represent the activities of the team, 4 players were chosen randomly and observed during the entire practice. Drills or exercises performed that were the same as, or similar to, components of the alternative warm-up were noted independently by each observer. One point was given for each athlete performing the drill for the correct duration (repetitions or distance, and sets), with a second point awarded for using correct technique. An athlete performing the warm-up completely and correctly without additional relevant drills or exercises conducted in practice would receive a total of 38 points. In this report, the scores of the 2 observers were averaged and are reported in 4 categories of activities: warm-up, stretching, plyometric exercises, or agility drills. Coaches of the 8 observed teams also completed coaches' surveys regarding their knowledge, attitudes, beliefs, and behaviors regarding injury prevention in women's soccer so that general differences between coaches in intervention and control groups could be assessed.

Finally, ATCs from all participating teams (both intervention and control), with input from coaches and strength trainers, were asked to complete an end of season survey regarding training drills performed on field, in the gym, or during weight room workouts covering the use of common proprioceptive and neuromuscular training drills, including those used in the PEP Program.

Statistical Methods and Data Analysis

Data were entered into an Access database and analyzed using SAS version 8.2 (SAS Institute Inc, Cary, North Carolina). We selected an as-treated analysis (per protocol), rather than an intent-to-treat analysis, because we were primarily interested in documenting efficacy rather than effectiveness to answer the question, "if the program is used, does it decrease risk?" This intervention is a physical training program requiring repetition for effect; thus, intervention teams failing to complete the program 12 or more times were excluded from these analyses. Eight teams were excluded from the analysis because they did not use the program 12 or more times. As noted in the pilot and coaches' surveys, this amount of repetition was needed before athletes reported that the program was less demanding, suggesting a physical adaptation. Excluded teams also failed to provide substantial information on athlete participation and/or injuries, making their data unreliable. To examine the robustness of our findings, we later returned data from excluded teams to the analysis. Injury rates were calculated based on AEs and are expressed as rate per 1000 AE. Statistical tests were based on a z statistic for rate ratios computed using Kish's formula for the variance of a ratio.[15] A P value less than .05 was considered significant. No study authors who participated in the design and development of the PEP Program participated in data collection or analysis.

RESULTS

Sixty-one teams completed the study, representing a diversity of region, conference, and competitive success. Each region was well represented with 10% to 34% of teams in each region. At least one team participated from 28 of 30 (93%) conferences. Additionally, participating teams represented a range of competitive success; 8 teams had finished the previous (2001) season ranked in the top 30 Division I teams.

A total of 1435 athletes participated, with 852 athletes on 35 control teams and 583 athletes on 26 intervention teams.

TABLE 3
Comparison of Selected Injuries and Injury Rates (per 1000 AE) by Study Group and Selected Variables[a]

	Intervention		Control			
	n	Rate per 1000 AE	n	Rate per 1000 AE	z	*P* Value
Total (practice + game + other[b])						
All knee injuries	40	1.136	58	1.096	0.17	.863
ACL	7	0.199	18	0.340	–1.29	.198
Noncontact ACL	2	0.057	10	0.189	–1.84	.066
Practice						
All knee injuries	8	0.301	19	0.469	–1.11	.265
ACL	0	0.000	6	0.148	–2.45	.014
Noncontact ACL	0	0.000	3	0.074	–1.73	.083
Game						
All knee injuries	29	3.372	37	2.982	0.49	.624
ACL	7	0.814	12	0.967	–0.37	.712
Noncontact ACL	2	0.233	7	0.564	–1.23	.218
History of past ACL injury						
All knee injuries	7	0.199	16	0.302	–0.97	.331
ACL	1	0.028	7	0.132	–1.81	.071
Noncontact ACL	0	0.000	4	0.076	–2.00	.046
No history of ACL injury						
All knee injuries	33	0.937	41	0.775	0.80	.425
ACL	6	0.170	10	0.189	–0.20	.839
Noncontact ACL	2	0.057	6	0.113	–0.92	.356
Early in season (weeks 0-5)						
All knee injuries	27	1.190	38	1.165	0.08	.933
ACL	7	0.309	13	0.399	–0.56	.575
Noncontact ACL	2	0.088	7	0.215	–1.24	.216
Late in season (weeks 6-11)						
All knee injuries	13	1.037	20	0.996	0.11	.911
ACL	0	0.000	5	0.249	–2.24	.025
Noncontact ACL	0	0.000	3	0.149	–1.73	.083

[a]AE, athlete exposure; ACL, anterior cruciate ligament.
[b]"Other" includes fitness and conditioning activities performed outside of practices and games.

Comparisons of athletes and exposures in the 2 groups are presented in Table 2. No significant differences were noted with regard to age, height, weight, or history of knee injuries.

Athletes on intervention and control teams participated in a similar number of games and practices throughout the season. Intervention teams used the PEP Program an average of 25.8 times during the season with a range of 12 to 37 uses.

The most common knee injuries were medial collateral ligament injuries (either in isolation or occurring with meniscal or cartilage injury), which accounted for 35% of knee injuries in each group (rates of 0.398 and 0.383 in intervention athletes [IA] and control athletes [CA], respectively), followed by isolated meniscal or cartilage injury accounting for 25% of knee injuries in the control teams (0.274) and 35% in intervention (0.398).

The most substantial difference in injury rates was noted when comparing ACL injury rates—specifically noncontact ACL injury rates—between the 2 groups (Table 3). In the IA, 7 ACL injuries were reported compared with 18 in the CA (0.199 per 1000 AE vs 0.340; $P = .198$), a nonsignificant finding despite a 41% lower ACL injury rate than that of CA. Similarly, IA reported 2 noncontact ACL injuries compared with 10 in CA (0.057 vs 0.189, $P = .066$), a 70% decrease. In practices, no ACL injuries occurred in IA compared with 6 in CA (0.000 vs 0.148; $P = .014$). In games the difference was not significant (0.814 vs 0.967; $P = .712$); however, the IA noncontact ACL injury rate in games was less than half that of CA (0.233 vs 0.564; $P = .218$). Intervention athletes with a self-reported history of ACL injury suffered ACL injuries at a rate almost 5 times less than the rate of CA with a history of ACL injury (0.028 vs 0.132, respectively; $P = .071$). This difference reached statistical significance when limited to noncontact ACL injuries because no IA with a history of ACL injury suffered a noncontact ACL injury during this study (0.000 vs 0.076; $P = .046$). In athletes without a history of ACL injury, the noncontact ACL injury rate in IA was approximately half that of CA (0.057 vs 0.113; $P = .356$). In the first 6 weeks of the season, while total ACL injury rates were similar between the 2 groups (0.309 vs 0.399), the noncontact ACL injury rates in IA were less than half that of CA (0.088 vs 0.215; $P = .216$); in the last 6 weeks of the season, the difference in total ACL injuries reached statistical significance because IA suffered no ACL injuries in the second half of the season (0.000 vs 0.249; $P = .025$).

To assess the robustness of this as-treated analysis, all data received from all intervention teams (including those previously excluded) were analyzed. The statistically significant

TABLE 4
Frequency of Observed Athletes Performing Selected Activities[a]

Components	Control Athletes (n = 32) Average/Athlete	Intervention Athletes (n = 32) Average/Athlete	Program Score[b]
Warm-up	4.7	6.1	6
Stretching	6.2	21.5	10
Strengthening	0.2	5.6	6
Plyometrics	0.6	7.9	10
Agility drills	0.3	4.8	6
Total of all components	11.9	46.0	38
Total of strengthening, plyometric, and agility drills	1.1	18.4	22

[a]Higher scores represent more athletes performing more drills/exercises related to the intervention program during an observed practice. Scores can be higher than the sample score if additional related drills or exercises were used during practice.
[b]Intervention program alone as designed.

reductions observed in ACL injuries in practice and late in the season remained. However, the reduction in noncontact ACL injury rates in IA with a history of ACL injury did not continue to be statistically significant, although the rate was 64% less than CA with a history of ACL injury. Anterior cruciate ligament injury rates among intervention teams were potentially falsely increased in this intent-to-treat analysis because of poor reporting of AEs by excluded teams.

The observations of practices of 4 intervention and 4 control teams twice during the season demonstrated that control teams did not use strengthening, plyometric training, or agility drills routinely in their on-field practices (Table 4). Higher scores suggest more athletes spending time doing more exercises or drills in that category. In both groups, time devoted to these activities in practice generally decreased between the visits early and late in the season. Both IA and CA were observed frequently participating in warm-up, for example, light jogging, and stretching activities; however, few CA were observed completing drills designed to improve strength, balance, or coordination through strengthening, plyometric, or agility activities. The differences between intervention and control activities were highlighted by examining scores for activities related to strengthening, plyometrics, or agilities. The average score for the observed athletes in the intervention group was 18.4, while that for CA was 1.1, suggesting that control groups did not routinely choose drills and exercises related to neuromuscular and plyometric training.

These observations were supported by results from the end of season surveys completed by all teams. The ATCs of the control teams reported that the coaches did not routinely use plyometric training or agility drills on field, in the weight room, or during off-field practice time, suggesting little outside contamination of the control group. The ATCs of the intervention group similarly reported little additional plyometric or agility drills outside of the alternative warm-up used by these teams. Reported compliance with the program varied among intervention teams from 12 to 37 times, with an average of about 26 uses per team. Finally, responses to the coaches' survey (given to observed teams) questioning knowledge, attitudes, beliefs, and behaviors regarding injury prevention in women's soccer did not differ substantially between coaches of intervention and control teams.

DISCUSSION

The PEP Program was designed specifically to prevent noncontact ACL injuries but also may protect the athlete from some contact ACL injuries, potentially from benefits in strength or agility. This may partially explain the decrease in total (contact + noncontact) ACL injury rates.

The results presented here suggest that the PEP Program of neuromuscular and proprioceptive training is effective in preventing ACL injuries and can be accomplished during regular practice time without the need for additional special equipment or training; this supports earlier findings.[18] The program appears to be feasible and safe to perform; ATCs reported some athlete complaints of soreness early in the intervention. A single adverse event in the intervention group was reported. Early in the season, an athlete tripped during the program while doing a lateral hop over the ball resulting in a tibia/fibula fracture. Immediate steps were taken to ensure that all intervention teams used short cones or other flexible devices for hopping drills rather than a ball. No other injuries related to the use of the program were reported.

When using a neuromuscular intervention such as the PEP Program, it may take repeated use during several weeks for the athlete to demonstrate early changes in strength, balance, and proprioception. This might explain the differences in ACL injury rates, which were more pronounced later in the season, suggesting a cumulative benefit of the training. We hypothesize that any musculoskeletal adaptations that may have occurred as a result of performing the PEP Program would be more evident later in the season. The coaches of observed teams also reported that the athletes generally required 6 to 12 repetitions of the program before it was no longer as physically challenging to complete, depending on the athletes' baseline level of

fitness. Potentially, the differences in injury rates observed might have been greater or occurred earlier in the season if the program had been routinely initiated during the off-season or introduced during preseason practices.

These results are subject to several limitations. First, we were unable to control the drills and exercises used during practice in both groups. Any team could theoretically do the drills and exercises that make up the alternative warm-up during practices. However, both the observational survey of a subgroup (13%) of teams and the end of season survey of drills and exercises completed by ATCs of all teams suggest that control teams did not appear to routinely use any of the drills and exercises that are components of the strengthening, plyometric, and agility portion of the PEP Program. Thus, control athletes likely were not exposed to substantial or consistent neuromuscular or proprioceptive training during the season.

Second, we were unable to fully control the use of and fidelity to the program by the intervention teams. Each team's ATC reported each week regarding how many times the team completed the program, but we had no way to validate their reports. The observational survey of 4 intervention teams as they used the program twice during the season shows that they easily completed the program as intended without referencing the materials, suggesting repetition. Coaches often were observed correcting athlete's biomechanical technique during the drills to ensure maximal benefit. While observed intervention teams appeared to be routinely using the program with fidelity, we cannot assume that other intervention teams did. In which case, results may be an underestimate of the program's potential impact.

Third, it is not known whether these findings can be generalized to collegiate athletes from other divisions or to other age groups. The cohort itself may have had an element of inherent selection bias. The incidence of ACL injury typically is highest among female athletes between the ages of 14 to 18 years, therefore, the athletes who have successfully engaged in Division I collegiate soccer may be biomechanically or technically superior than their younger or age-matched counterparts.[10]

Finally, this study lacked the statistical power to sufficiently compare subgroups, for example, age and experience; despite data from 1435 athletes on 61 teams for an entire season, only 25 ACL injuries were reported. Anterior cruciate ligament injuries in general, and noncontact ACL injuries in particular, require a large number of exposures for study. An examination of power calculations before the study suggested that 100 teams might be needed to fully examine desired subgroups; however, time and funding prevented including more teams or extending the study. Several of the measures of differences in rates for noncontact ACL injuries approached significance (ie, noncontact ACL injuries in total, in practice, and late in season, and ACL injuries in those with a history of injury). Differences in ACL injury in these subgroups and others may have reached statistical significance if a larger sample size or a larger number of exposures had been possible.

National Collegiate Athletic Association Division I female soccer teams were used in this study to ensure that each team had the resources and dedicated athletic training staff to complete the study. However, the intervention also has been successfully implemented and appeared promising in a nonrandomized study among 14- to 18-year-old competitive female club soccer players without direct oversight from an ATC or a physical therapist.[18] This suggests that the program may be generalizable to other age groups and levels of play without direct oversight from a member of the medical community. Additionally, although the program is presently designed with soccer-specific drills, it may be modified to be used in other high-risk team sports such as basketball or volleyball. Further studies are currently being conducted to determine the program's effectiveness in different age cohorts, in men, and in athletes from different high-risk sports.

CONCLUSION

Implementation of an alternative warm-up program consisting of specific neuromuscular and proprioceptive training techniques (PEP Program) may reduce the risk of noncontact ACL injuries in collegiate female soccer players. While nonsignificant trends were noted in the overall ACL risk, significant reductions were found in the risk of ACL injuries in practice and the second half of the season. Additionally, the program may be of particular benefit among those athletes with a history of ACL injury. The PEP Program can be readily incorporated into practice time of collegiate soccer athletes without additional resources. It can be accomplished in a team setting and requires no additional special equipment and little training to implement effectively. Further research using an intent-to-treat analysis may test its general effectiveness under less controlled circumstances.

ACKNOWLEDGMENT

The findings and conclusions in this report are those of the authors and do not necessarily represent the views of CDC. We would like to thank the players and coaches who participated in this study, and the athletic trainers who diligently collected the information. We would also like to express our gratitude to the organizations who contributed collaboratively to support and fund this study: the International Federation of Football Associations (FIFA), the American Academy of Orthopedic Surgeons, the American Orthopedic Society for Sports Medicine, the National Collegiate Athletic Association, the Santa Monica Orthopedic and Sports Medicine Research Foundation, and the U.S. Centers for Disease Control and Prevention.

REFERENCES

1. Arendt E, Dick R. Knee injury patterns among men and women in collegiate basketball and soccer: NCAA data and review of the literature. *Am J Sports Med.* 1995;23:694-701.
2. Arendt EA, Agel J, Dick RW. Anterior cruciate ligament injury. Patterns among collegiate men and women. *J Athl Train*. 1999;34:86-92.

3. Caraffa A, Cerulli G, Projetti M, Aisa G, Rizzo A. Prevention of anterior cruciate ligament injuries in soccer. A prospective controlled study of proprioceptive training. *Knee Surg Sports Traumatol Arthrosc.* 1996;4:19-21.
4. Chandy TA, Grana WA. Secondary school athletic injury in boys and girls: a 3-year comparison. *Phys Sportsmed.* 1985;13:106-111.
5. Faude O, Junge A, Kindermann W, Dvorak J. Injuries in female soccer players: a prospective study in the German national league. *Am J Sports Med.* 2005;33:1694-1700.
6. Frank CB, Jackson DW. The science of reconstruction of the anterior cruciate ligament. *J Bone Joint Surg Am*. 1997;79:1556-1576.
7. Garrick JG, Requa RK. Anterior cruciate ligament injuries in men and women: how common are they? In: Griffin LY, ed. *Prevention of Noncontact ACL Injuries*. Rosemont, Ill: American Academy of Orthopaedic Surgeons; 2001:1-9.
8. Gray J, Taunton JE, McKenzie DC, Clement DB, McConkey JP, Davidson RG. A survey of injuries to the ACL of the knee in female basketball players. *Int J Sports Med.* 1985;6:314-316.
9. Griffin LY, Agel J, Albohm MJ, et al. Noncontact anterior cruciate ligament injuries: risk factors and prevention strategies. *J Am Acad Orthop Surg*. 2000;8:141-150.
10. Griffin LY, Albohm MJ, Arendt EA, et al. Understanding and preventing noncontact anterior cruciate ligament injuries: a review of the Hunt Valley II meeting, January 2005. *Am J Sports Med*. 2006;34: 1512-1532.
11. Gwinn DE, Wilckens JH, McDevitt ER, Ross G, Kao TC. The relative incidence of anterior cruciate ligament injury in men and women at the United States Naval Academy. *Am J Sports Med.* 2000;28(1):98-102.
12. Heidt RS, Sweeterman LM, Carlonas RL, Traub JA, Tekulve FX. Avoidance of soccer injuries with preseason conditioning. *Am J Sports Med*. 2000;28:659-662.
13. Hewett TE, Lindenfeld TN, Riccobene JV, Noyes FR. The effect of neuromuscular training on the incidence of knee injury in female athletes. *Am J Sports Med*. 1999;27:699-704.
14. Junge A, Dvorak J. Soccer injuries: a review on incidence and prevention. *Sports Med.* 2004;34:929-938.
15. Kish L. *Survey Sampling.* New York, NY: John Wiley & Sons; 1965.
16. Lindenfeld TN, Schmitt DJ, Hendy MP, Mangine RE, Noyes FR. Incidence of injury in indoor soccer. *Am J Sports Med.* 1994;22:364-371.
17. Malone TR, Hardaker WT, Garrett WE, et al. Relationship of gender to ACL injuries in intercollegiate basketball players. *J South Orthop Assoc*. 1992;2:36-39.
18. Mandelbaum BR, Silvers HJ, Watanabe DS, et al. Effectiveness of a neuromuscular and proprioceptive training program in preventing anterior cruciate ligament injuries in female athletes: 2-year follow-up. *Am J Sports Med.* 2005;33:1003-1010.
19. Myklebust G, Engebretsen L, Braekken IH, Skjølberg A, Olsen OE, Bahr R. Prevention of anterior cruciate ligament injuries in female team handball players: a prospective intervention study over 3 seasons. *Clin J Sport Med.* 2003;13:71-78.
20. Olsen OE, Myklebust G, Engebretsen L, Holme I, Bahr R. Exercises to prevent lower limb injuries in youth sports: cluster randomized controlled trial. *BMJ.* 2005;330(7489):449.
21. Strand T, Wisnes AR, Tvedte R, et al. ACL injuries in team handball. *J Nor Med Assoc*. 1990;110:45-48.
22. Thacker SB, Stroup DF, Branche CM, Gilchrist J, Goodman RA, Porter Kelling E. Prevention of knee injuries in sports. A systematic review of the literature. *J Sports Med Phys Fitness.* 2003;43(2):165-179.
23. Wedderkopp N, Kaltoft M, Lundgaard B, Rosendahl M, Froberg K. Prevention of injuries in young female players in European team handball. A prospective intervention study. *Scand J Med Sci Sports*. 1999;9:41-47.
