# Supplementary material for: Comparative Effectiveness of Multi-Component, Exercise-Based Interventions for Preventing Soccer-Related Musculoskeletal Injuries: A Systematic Review and Meta-Analysis
Source: Healthcare (Basel). 2025 Mar 29;13(7):765. doi: 10.3390/healthcare13070765 (PMC11988859; doi:10.3390/healthcare13070765)
Supplement: Supplementary file 1 [file healthcare-13-00765-s001.zip › Additional material Included literature/Hammes2015.pdf]

This article was downloaded by: [Dicle University]
On: 17 November 2014, At: 02:21
Publisher: Routledge
Informa Ltd Registered in England and Wales Registered Number: 1072954 Registered office: Mortimer House, 37-41 Mortimer Street, London W1T 3JH, UK

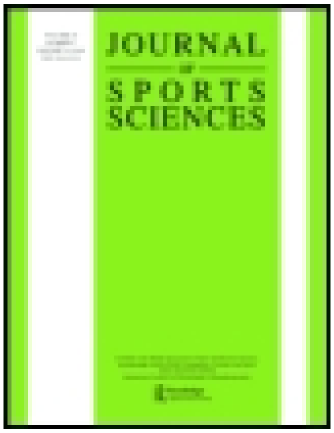

Journal of Sports Sciences
Publication details, including instructions for authors and subscription information:
http://www.tandfonline.com/loi/rjsp20

Injury prevention in male veteran football players - a randomised controlled trial using “FIFA 11+”

Daniel Hammes[a], Karen aus der Fünten[a], Stephanie Kaiser[a], Eugen Frisen[a], Mario Bizzini[b] & Tim Meyer[a]

[a] Institute of Sports and Preventive Medicine, Saarland University, Saarbrücken, Germany

[b] FIFA-Medical Assessment and Research Centre, Schulthess Clinic, Zürich, Switzerland

Published online: 05 Nov 2014.

To cite this article: Daniel Hammes, Karen aus der Fünten, Stephanie Kaiser, Eugen Frisen, Mario Bizzini & Tim Meyer (2014): Injury prevention in male veteran football players - a randomised controlled trial using “FIFA 11+”, Journal of Sports Sciences, DOI: 10.1080/02640414.2014.975736

To link to this article: http://dx.doi.org/10.1080/02640414.2014.975736

PLEASE SCROLL DOWN FOR ARTICLE

Taylor & Francis makes every effort to ensure the accuracy of all the information (the “Content”) contained in the publications on our platform. However, Taylor & Francis, our agents, and our licensors make no representations or warranties whatsoever as to the accuracy, completeness, or suitability for any purpose of the Content. Any opinions and views expressed in this publication are the opinions and views of the authors, and are not the views of or endorsed by Taylor & Francis. The accuracy of the Content should not be relied upon and should be independently verified with primary sources of information. Taylor and Francis shall not be liable for any losses, actions, claims, proceedings, demands, costs, expenses, damages, and other liabilities whatsoever or howsoever caused arising directly or indirectly in connection with, in relation to or arising out of the use of the Content.

This article may be used for research, teaching, and private study purposes. Any substantial or systematic reproduction, redistribution, reselling, loan, sub-licensing, systematic supply, or distribution in any form to anyone is expressly forbidden. Terms & Conditions of access and use can be found at http://www.tandfonline.com/page/terms-and-conditions

Journal of Sports Sciences, 2014
http://dx.doi.org/10.1080/02640414.2014.975736

Routledge
Taylor & Francis Group

Injury prevention in male veteran football players – a randomised controlled trial using "FIFA 11+"

DANIEL HAMMES[1], KAREN AUS DER FÜNTEN[1], STEPHANIE KAISER[1], EUGEN FRISEN[1], MARIO BIZZINI[2] & TIM MEYER[1]

[1]*Institute of Sports and Preventive Medicine, Saarland University, Saarbrücken, Germany and* [2]*FIFA–Medical Assessment and Research Centre, Schulthess Clinic, Zürich, Switzerland*

(*Accepted 7 October 2014*)

Downloaded by [Dicle University] at 02:21 17 November 2014

Abstract

The warm-up programme "FIFA 11+" has been shown to reduce football injuries in different populations, but so far veteran players have not been investigated. Due to differences in age, skill level and gender, a simple transfer of these results to veteran football is not recommended. The purpose of this study was to investigate the preventive effects of the "FIFA 11+" in veteran football players.

Twenty veteran football teams were recruited for a prospective 9-month (1 season) cluster-randomised trial. The intervention group (INT, n = 146; 45 ± 8 years) performed the "FIFA 11+" at the beginning of each training session, while the control group (CON, n = 119; 43 ± 6 years) followed its regular training routine. Player exposure hours and injuries were recorded according to an international consensus statement.

No significant difference was found between INT and CON in overall injury incidence (incidence rate ratio [IRR]: 0.91 [0.64–1.48]; P = 0.89). Only severe injuries reached statistical significance with higher incidence in CON (IRR: 0.46 [0.21–0.97], P = 0.04).

Regular conduction (i.e. once a week) of the "FIFA 11+" did not prevent injuries in veteran footballers under real training and competition circumstances. The lack of preventive effects is likely due to the too low overall frequency of training sessions.

Keywords: *soccer, injuries, neuromuscular training, preventive strategies, elderly*

Introduction

Football is played by more than 265 million individuals worldwide (FIFA, 2013) and, due to this popularity, receives worldwide attention in the media. Many players participate in active football as a recreational sport into older age. Organised football for older players can be found worldwide (e.g. Europe, South America, Canada, United States, Australia). About 27% (or 1.8 million) of registered players in the German Football Federation are veteran players (Woll & Dugandzic, 2007). German veteran footballers are restricted to a certain minimum age typically, 30 or 32 years dependent upon the local federation. Recent studies have shown that recreational football can improve metabolic, cardiovascular and musculoskeletal parameters and can therefore be an effective tool to protect against certain lifestyle diseases (Krustrup, Aagaard, et al., 2010; Krustrup, Christensen, et al., 2010; Randers et al., 2010). However, playing also involves the risk of injuries (Dvorak & Junge, 2000; Faude, Junge, Kindermann, & Dvorak, 2006; Junge & Dvorak, 2004; Peterson, Junge, Chomiak, Graf-Baumann, & Dvorak, 2000). To date, several lower limb injury preventive programmes have been investigated for their effectiveness in sport (Emery & Meeuwisse, 2010; Gilchrist et al., 2008; Herman, Barton, Malliaras, & Morrissey, 2012; Walden, Atroshi, Magnusson, Wagner, & Hagglund, 2012). The "Knee Injury Prevention Program," the "Prevent Injury and Enhance Performance" strategy, the "HarmoKnee" programmes and the "Anterior Knee Pain Prevention Training Programme" have been shown to significantly reduce lower limb injuries and specific injuries such as knee injuries in female football, basketball players and female military recruits (Coppack, Etherington, & Wills, 2011; Kiani et al., 2010; LaBella et al., 2011; Mandelbaum et al., 2005).

Correspondence: Daniel Hammes, Department of Sport, Exercise and Health, University of Basel, Birsstrasse 320B, 4052 Basel, Switzerland. E-mail: daniel.hammes@unibas.ch

© 2014 Taylor & Francis

Downloaded by [Dicle University] at 02:21 17 November 2014

The FIFA (Féderation Internationale de Football Association) Medical Assessment and Research Centre (F-MARC) by the football world federation FIFA developed a soccer-specific preventive programme called "The 11," which aimed to reduce common soccer injuries. Its effectiveness in injury reduction was shown in a large cohort study with Swiss amateur players (Junge et al., 2011). However, a study from Steffen, Myklebust, Olsen, Holme, and Bahr (2008) failed to verify any effect of "The 11" in a cluster-randomised controlled trial with female youth players. In this study, the authors attributed the lack of effectiveness to low compliance. Absence of progression and variation in the programme may have led to low motivation among coaches and players (Steffen et al., 2008). Furthermore, Gatterer, Ruedl, Faulhaber, Regele, and Burtscher (2012) did not find a reduction of injuries when using "The 11" in male adult amateur footballers as well. Although good compliance was reported by Van Beijsterveldt et al. (2012), only knee injuries were reduced in male amateur players.

"FIFA 11+" is an advanced version of "The 11" and contains some relevant changes. "FIFA 11+" encompasses additional exercises and provides variation and progression. FIFA recommends its use as a complete warm-up programme before each training session. Soligard et al. (2008) evaluated "FIFA 11+" in young female players over one season and found a significantly lower risk of overall injuries in the intervention group; however, the primary outcome of lower extremity injuries failed to reach significance. Steffen, Emery, et al. (2013) supported these findings in young Canadian female players. However, no such programme has been investigated for its effectiveness in veteran football players.

Veteran football players, however, vary greatly in age, training frequency, fitness level and injury history. Furthermore, gender is a known influence factor on injury characteristics, for example anterior cruciate ligament injuries (Prodromos, Han, Rogowski, Joyce, & Shi, 2007). This has led to the assumption that there might be differences in the effectiveness of a football-specific intervention among different populations. Therefore, we decided to conduct a cluster-randomised controlled trial over one season to evaluate the effectiveness of "FIFA 11+" on injury incidence in a realistic setting of veteran players.

Materials and methods

The study was undertaken in accordance with the Declaration of Helsinki and approved by the local ethics committee (approval number: 151/11, Ärztekammer of Saarland, Saarbrücken, Germany). Furthermore, it was registered at ClinicalTrials.gov (identifier: NCT01993056).

General design

Approximately 6 months prior to the start of the study, we announced our intentions during a veteran football event with an invitation to participate. Thereafter, each club with veteran football teams in the county Saarland (about one million inhabitants) was contacted by letter, email and telephone calls. This was done in cooperation with the county's football federation (Saarländischer Fußballverband). The first 20 teams, who confirmed their participation, were cluster-randomised (teams) into two groups (intervention group and control group) (Figure 1). Each individual gave written informed consent to take part in this study. During the season 2011/2012 (9 months), the intervention group performed "FIFA 11+" at the beginning of each team training session instructed by trained sport scientists, while the control group followed its regular training routine, usually without a structured warm-up programme. We decided not to conduct the 11+ before matches as we encountered several issues. The time frame between the arrival of the players and kick-off is usually very short and loaded with several other things such as planning tactics. Additionally, not all players arrive at the same time and therefore did not necessarily begin the warm-up together, with most of our participating teams also not agreeing to a warm-up without a ball.

Participants

A priori, we decided to recruit a total of 20 teams from the county Saarland. The teams performed training sessions at regular intervals and took part in league or friendly matches against other clubs. With regard to the local rules for veteran football players, the minimum age for players was set as 32 years. Due to incomplete reporting, two teams (one from the intervention group and one from the control group) were excluded from our analysis, so statistics refer to only 18 teams.

Intervention programme: FIFA 11+

"FIFA 11+" is a complete warm-up programme consisting of three parts, a total of 15 different exercises with the focus on neuromuscular effects to the lower extremities and to the core. An overview of the whole programme including manual and videos of the exercises is presented in F-MARC's website (http://f-marc.com/11plus/home). The first and third parts include running exercises, beginning "at slow speed combined with active stretching and

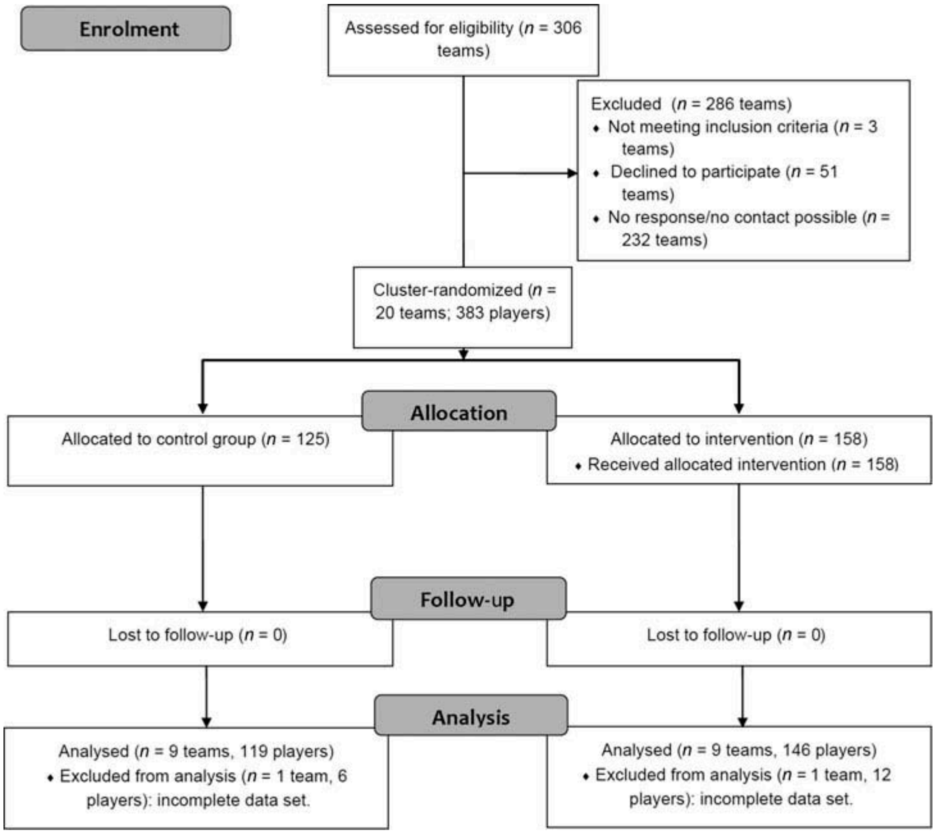

Figure 1. Flow chart of teams and players throughout the study.

Downloaded by [Dicle University] at 02:21 17 November 2014

controlled partner contacts" and finishing "at moderate/high speed combined with planting/cutting movements" (Reis, Rebelo, Krustrup, & Brito, 2013; Soligard et al., 2008). Part 2 consists of six strength, plyometrics and balance exercises with the focus on core and leg strength, proprioception and stability, with three levels of variation provided for each exercise. In the intervention group, we increased the level of part 2 with each team every 3 months (total study duration: 9 months). As mentioned before, the programme in the intervention group was instructed in every training session by trained sport scientists familiar with the correct technique of all exercises. If necessary, poor technique was corrected and demonstrated properly to ensure a high level of quality.

Data collection and definitions

Injuries were defined according to the consensus statement on injury definitions and data collection procedures in football studies (Fuller et al., 2006) (Table I). Only time-loss injuries (Table I) were considered in this study. We classified all injuries into categories according to injured body part, type of injury, recurrent injury or not, overuse or traumatic injury and training or match play injury. The injury severity was set depending on the number of days from the date of injury to full return to team training or matches according to the consensus statement (Table I).

During the study, player exposure hours (training and match) and injuries were recorded. If there was no team coach, one player per team was responsible for organisational affairs ("team advisor") and recorded exposure hours from each player individually in paper or computer (participation in team training and match in minutes). When players were absent, the reasons (injury, illness or other reasons) were noted. The team advisor transferred the data at least monthly to the study personnel. Injured players were advised to complete an injury report form with questions concerning the injured body part, type of injury, recurrence, cause and occurrence in training or match. If additional questions were required, the participants were contacted by telephone, email or personally.

The perceived exertion was measured by the "session Rating of Perceived Exertion (RPE)" by multiplying RPE and training time (Foster et al., 2001) one time per level in each team in the intervention group (last training sessions at level 1, level 2 and level 3, respectively). Immediately after the warm-up, the players completed a form containing the question "How exhausting was the warm up today?" and rated it between 0 and 10 (CR10)

Table I. Definitions (Fuller et al., 2006).

Term	Definition
Injury	Any physical complaint sustained by a player that results from a football match or football training.
Time-loss injury	Injury that results in a player being unable to take a full part in future football training or match play.
Minimal injury	Absence from training and matches for 1–3 days.
Mild injury	Absence from training and matches for 4–7 days.
Moderate injury	Absence from training and matches for 8–28 days.
Severe injury	Absence from training and matches for more than 28 days.
Match	Play between teams from different clubs.
Training	Team-based physical activities.
Overuse injury	Caused by repeated microtrauma without a single, identifiable event responsible for the injury.
Traumatic injury	Injury resulted from a specific, identifiable event.
Recurrent injury	Injury of the same type and at the same side as an index injury and which occurs after a player's full return to full participation from the index injury.
Early recurrence	Recurrent injury occurring within 2 months of a player's return to full participation.
Late recurrence	Recurrent injury occurring 2–12 months after a player's return to full participation.
Delayed recurrence	Recurrent injury occurring more than 12 months after a player's return to full participation.

Downloaded by [Dicle University] at 02:21 17 November 2014

(Foster et al., 2001). The supervising sport scientists measured the time needed for the completion of FIFA 11 +.

Statistics

The statistical analysis was performed using the package Statistica 8.0 (StatSoft Europe GmbH, Hamburg, Germany). Descriptive data are presented as means and standard deviations or incidences (injuries per 1000 h) and corresponding 95% confidence intervals (95% CI). Anthropometrics and baseline information were compared between groups by *t*-tests for independent samples and 2 × 2 chi-square tests for distributions. To test injury incidences (number of injuries/1000 player hours) for differences between the intervention group and the control group, we used rate ratios, their corresponding 95% CI and *Z*-Statistics to determine statistical significance. The numbers of lost days due to injuries are given as median and 25/75 quartiles. Groups were compared by the Mann–Whitney U-test. An α-error of $P < 0.05$ was considered significant in all calculations and all tests were two-tailed.

Results

Anthropometric data ($n = 265$ players; intervention group: $n = 146$; control group: $n = 119$) are shown in Table II, further baseline information in Table III and the flow of participants in Figure 1.

Exposure and compliance

Participants of the intervention group played overall 4172 h of football (2934 training and 1238 match hours) and those in the control group 2937 h (1864 training and 1073 match hours). The amount of match time did not differ significantly between both groups (intervention group: 8.9 ± 5.6 h; control group: 9.4 ± 4.6 h; $P = 0.44$), but time spent on

Table II. Anthropometric data.

	INT		CON		
	Mean	*s*	Mean	*s*	*P*-value
Anthropometrics $n = 265$					
Age (years)	45.2	7.7	43.1	6.5	0.02
Height (cm)	178	7	179	7	0.08
Weight (kg)	85.3	12.9	84.1	11.5	0.41
BMI ($kg \cdot m^{-2}$)	27.0	3.4	26.1	2.7	0.03
Football experience (years) $n = 235$	32.3	12.0	30.5	10.3	0.23

Note: INT: intervention group; CON: control group.

Table III. Playing position and injury history from player´s baseline information form.

	INT		CON		
	%	*n*	%	*n*	*P*-value
Playing position					
Goalkeeper	4.8	7	4.2	5	0.82
Defender	28.1	41	27.7	33	0.88
Midfielder	29.5	43	33.6	40	0.47
Attackers	12.3	18	15.1	18	0.51
Undefined	11.6	17	11.8	14	0.98
No report	13.7	20	7.6	9	0.11
Injury history					
Previous major injuries	81	118	85	101	0.39
Previous surgery	48	70	52	62	0.50
Acute complaints (without time-loss)	53	77	55	65	0.76

Note: INT: intervention group; CON: control group.

training was higher in the intervention group than in the control group (intervention group: 20.1 ± 12.3 h; control group: 15.7 ± 8.1 h; $P < 0.001$). On average, the teams performed 24 ± 7 training sessions (intervention group: 27 ± 8; control group: 21 ± 4; $P = 0.03$) and 16 ± 5 matches (intervention group: 17 ± 6; control group: 14 ± 4; $P = 0.23$) during 9 months. "FIFA 11+" was performed in 98% of all training sessions of the teams in the intervention group (e.g. due to unscheduled training sessions were conducted during weekends) with each player participating in 47% of their teams 11+ sessions. Calculated per single player, the average number of training sessions was 13 ± 7 (intervention group: 15 ± 8; control group: 12 ± 6; $P = 0.002$). The mean number of performed 11+ sessions per player in the intervention group was 14 ± 8. Numbers of performed matches per player were not different between groups: total: 8 ± 5 (intervention group: 8 ± 5; control group: 8 ± 5; $P = 0.63$).

Effect of FIFA 11+

Session RPEs for FIFA 11+ are reported in Table IV. Results of injury characteristics are summarised in Table V. Only the group comparison of severe injuries reached statistical significance with a higher incidence in the control group (incidence rate ratio: 0.46 [0.21–0.97], $P = 0.04$).

With regard to the number of performed FIFA 11+ sessions (analysed by median split), the results did not differ between players with high (>14 FIFA 11+ sessions) and low exposure (≤14 FIFA 11+ sessions) for the overall number of injuries (given as injury incidence per 1000 h of football): 12.3 (8.4–16.3) vs. 12.0 (5.7–18.3); incidence rate

Table IV. Descriptive statistics of Ratings of Perceived Exertion (RPEs), attended time of 11+ and Session RPEs (RPE [unit] × time [min]). *n* represents the number of players participating in the last training session of each 11+ level of progression.

	Level 1 (*n* = 64)			Level 2 (*n* = 53)			Level 3 (*n* = 37)		
	RPE (unit)	Time (min)	Session RPE (unit)	RPE (unit)	Time (min)	Session RPE (unit)	RPE (unit)	Time (min)	Session RPE (unit)
Mean	3.5	25.6	88.0	3.5	27.2	94.4	3.8	26.4	99.4
s	1.4	1.3	36.2	1.0	1.7	28.4	1.1	1.6	32.0
Min	2.0	23.9	47.8	2.0	24.5	50.0	2.0	24.3	50.4
Max	10.0	27.8	252.0	7.0	29.5	206.5	7.0	28.3	198.3

Table V. Injury characteristics (injuries/1000 h).

	INT		CON			
	n (%)	Incidence (95% CI)	*n* (%)	Incidence (95% CI)	Rate Ratio (95% CI)	*P*-value
Overall	51 (100)	12.2 (8.9–15.6)	37 (100)	12.6 (8.5–16.7)	**0.91 (0.64–1.48)**	0.89
Training	16 (31)	5.5 (2.8–8.1)	15 (41)	8.1 (4.0–12.1)	**0.68 (0.33–1.37)**	0.28
Match	35 (69)	28.3 (18.9–37.6)	22 (59)	20.5 (11.9–29.1)	**1.38 (0.81–2.35)**	0.24
Contact	17 (33)	4.1 (2.1–6.0)	14(38)	4.8 (2.3–7.3)	**0.85 (0.42–1.73)**	0.66
Non-contact	34 (67)	8.2 (5.4–10.9)	23 (62)	7.8 (4.6–11.0)	**1.04 (0.61–1.77)**	0.88
Trauma	30 (59)	7.2 (4.6–9.8)	22 (59)	7.5 (4.4–10.6)	**0.96 (0.55–1.66)**	0.88
Overuse	20 (39)	4.8 (2.7–6.9)	15 (41)	5.1 (2.5–7.7)	**0.94 (0.48–1.83)**	0.85
Mild	7 (14)	1.7 (0.4–2.9)	7 (19)	2.4 (0.6–4.2)	**0.70 (0.25–2.01)**	0.51
Moderate	33 (65)	7.9 (5.2–10.6)	13 (35)	4.4 (2.0–6.8)	**1.79 (0.94–3.39)**	0.08
Severe	11 (22)	2.6 (1.1–4.2)	17 (46)	5.8 (3.0–8.5)	**0.46 (0.21–0.97)**	0.04
Upper extremities	8 (16)	1.9 (0.6–3.2)	7 (19)	2.4 (0.6–4.1)	**0.80 (0.29–2.22)**	0.67
Lower extremities	43 (84)	10.3 (7.2–13.4)	30 (81)	10.2 (6.6–13.9)	**1.01 (0.63–1.61)**	0.97
Thigh	17 (33)	4.1 (2.1–6.0)	11 (30)	3.7 (1.5–6.0)	**1.09 (0.51–2.32)**	0.83
Lower leg	10 (20)	2.4 (0.9–3.9)	4 (11)	1.4 (0.0–2.7)	**1.76 (0.55–5.61)**	0.34
Knee	6 (12)	1.4 (0.3–2.6)	4 (11)	1.4 (0.0–2.7)	**1.06 (0.30–3.74)**	0.93
Other locations	10 (20)	2.4 (0.9–3.9)	11 (30)	3.7 (1.5–6.0)	**0.64 (0.27–1.51)**	0.31
Muscle/tendon	33 (65)	7.9 (5.2–10.6)	19 (51)	6.5 (3.6–9.4)	**1.22 (0.70–2.15)**	0.49
Joint/ligament	9 (18)	2.2 (0.8–3.6)	10 (27)	3.4 (1.3–5.5)	**0.63 (0.26–1.56)**	0.32
Other types of injury	9 (18)	2.2 (0.7–3.6)	8 (22)	2.7 (0.8–4.6)	**0.79 (0.31–2.05)**	0.63
Recurrence	19 (37)	4.6 (2.5–6.6)	9 (24)	3.1 (1.1–5.1)	**1.49 (0.67–3.28)**	0.33
Delayed recurrence	10 (20)	2.4 (0.9–3.9)	7 (19)	2.4 (0.6–4.1)	**1.01 (0.38–2.64)**	0.99

Note: INT: intervention group; CON: control group; CI: confidence interval.

Downloaded by [Dicle University] at 02:21 17 November 2014

ratio: 1.03 (0.56–1.90), $P = 0.93$. The same applies to training (6.0 [2.7–9.2] vs. 4.0 [−0.5 to 8.5]; incidence rate ratio: 1.50 [0.43–5.26], $P = 0.53$) and match injuries (29.2 [17.5–40.8] vs. 26.6 [10.9–42.2]; incidence rate ratio: 1.10 [0.54–2.24], $P = 0.80$). The median lost time of injuries was 17 days (lower/upper quartile: 11/31 days). A significant difference between groups was observed: intervention group: 14 (10/25) and control group: 27 (12/39); $P = 0.04$.

Discussion

This study sought to examine the effectiveness of injury prevention with "FIFA 11+" in veteran football players. Results from previous studies led to the hypothesis that "FIFA 11+" may have preventive effects on injuries in football. The main finding of the present study was that "FIFA 11+" was not able to prevent injuries in our study group under the given (externally valid) training circumstances. There was only one significant group effect: less severe injuries in the intervention group and consequently a smaller number of injury-related days of absence. Soligard et al. (2008) similarly identified a significant effect of "FIFA 11+" on severe injuries, but they found additional significant positive effects on overall and overuse injuries and also their primary outcome "lower extremity injuries" almost reached significance. Furthermore, all other (non-significant) comparisons rather indicated a lower injury risk in the intervention group, which does not apply for our data.

Since the assessment of injury severity is based on days of absence from training or match, it must be taken into account that there might be an effect in the intervention group which is not due to the physiological effects of the "FIFA 11+" exercises. The players in the intervention group might have felt obliged to take part in the study and not to miss too many "FIFA 11+" sessions. This consideration is supported by the significant higher training exposure in the intervention group than in the control group. In fact, more recurrences would be expected as the players of the intervention group returned earlier compared to the control group. However, no significant differences between recurrent injuries between groups were revealed. Time to return may have been sufficient in most cases of both groups, although mean time to return was different, which further supports our assumption. Even though we would assume that the less severe injuries in the intervention group would be due to physiological reasons, it remains unclear if it was caused by the intervention or the higher training exposure in the intervention group. The higher training exposure alone may lead to a better fitness state, which is possibly linked to a reduced injury risk (Murphy, Connolly, & Beynnon, 2003; Steffen et al., 2010). However, Arnason et al. (2004) did not report significant results examining training exposure as a risk factor in football.

An inappropriate warm-up before matches in both the intervention group and the control group could be responsible for the considerable higher injury incidence in matches compared to training. However, the higher number of match injuries is a common finding in football and supported by many studies (Dauty & Collon, 2011; Ekstrand, Hagglund, & Walden, 2011; Junge & Dvorak, 2004). Indeed, there is a need for further studies looking specifically into the prevention of match injuries.

Frequency of FIFA 11+

Each club typically arranged only one training session per week, limiting the maximum frequency of "FIFA 11+" sessions to once per week. In addition, job-related and private commitments as well as holiday times, etc. reduced the number of training sessions. Neuromuscular programmes have proven to effectively reduce injuries in adolescent and young female football players, when performed 2 or 3 times a week (Gilchrist et al., 2008; Soligard et al., 2008; Steffen et al., 2008; Steffen, Emery, et al., 2013; Walden et al., 2012). Therefore, it is possible that the training stimulus was not frequent enough to achieve long-term neuromuscular effects, which are assumed to be effective in injury prevention (Murphy et al., 2003; Steffen et al., 2010). Reis et al. (2013) demonstrated that two "FIFA 11+" sessions per week improved physical fitness in adolescent male futsal players, whereas Impellizzeri et al. (2013) found improvements in neuromuscular control measures by conducting three sessions per week in male Italian amateur players, but not in performance measures, with the exception being leg flexor strength. Prior studies showed correlations between the number of performed "FIFA 11+" sessions and a lower injury risk (Soligard et al., 2010; Steffen, Emery, et al., 2013; Steffen, Meeuwisse, et al., 2013). It is noteworthy that due to the reality-oriented setting in our study, the average number of "FIFA 11+" sessions was lower than in the "low-compliance" groups in the other studies. We were aware of this in preparation of the study but wanted to ensure the highest possible level of external validity. A practical implication from this shortcoming might be the development of an additional, slightly modified programme that can be performed at home alone or at other regularly used settings outside the football training such as a gym.

Downloaded by [Dicle University] at 02:21 17 November 2014

Downloaded by [Dicle University] at 02:21 17 November 2014

FIFA 11+ exercises/individualisation

To our knowledge, no study has examined the effect of "FIFA 11+" in veteran football players so far and other experiences with this population and "FIFA 11 +" have not been reported in the scientific literature. The collected session RPEs show moderate subjective efforts on average, but the inter-individual variability is considerable and demonstrates that the demand for individual players in some cases might have been too low or too high. From our experience, given the number of repetitions during some exercises especially "Nordic Hamstring," the demands for many players were too intense, even during level one. Due to the fact that improvements in hamstring strength have been linked to a reduction in muscle injuries (Askling, Karlsson, & Thorstensson, 2003; Arnason, Andersen, Holme, Engebretsen, & Bahr, 2008), a modified volume (number of repetitions and/or sets) rather than an exclusion seems to be an option.

We increased the level of the programme and therefore the difficulty of the exercises in the second part only every third month for the team as a whole as a common practice in team sports. The progression was surely inadequate for some players in either direction, too easy, too hard. Ideally, the progression and variation should be adapted individually. In fact, this issue is present in nearly all fields of structured team sport training.

Motivation of players

The success of a prevention training programme usually depends on both the motivation of participating players and the motivation and skills of the coaches, since training stimuli and training effects are expected to be higher if exercises are performed with proper technique and adequate effort. Especially for neuromuscular control exercises, the importance of augmented feedback has been emphasised (Hewett, Myer, & Ford, 2005; Myer et al., 2013; Myklebust et al., 2003; Steffen et al., 2008) and was realised in this study by trained instructors. However, a possible lack of supervision and instructions has to be considered should the programme be complemented with additional exercises separate from football training as suggested earlier. We recognised a decline in motivation of many players after some months. In most cases, the players explained this by a lack of variation in the warm-up and a paucity of tasks including the ball. Furthermore, we recognised that the average time spent on the programme was higher than the proposed 20 min (mean training time for the "11+" was between 25.6 and 27.2 min) which could have further reduced motivation.

One of the most important points for the development and application of a preventive programme is its practicability and attractiveness in the target population (Finch, 2006). A possible improvement of "FIFA 11+" could be to modify and individualise the programme: players could be allowed to adjust the programme to their individual fitness level. Also, ball-based exercises might be added as the lack of the use of a football within the programme was one of the main points of criticism. The programme could finally be complemented by a do-it-yourself part to increase the frequency of preventive sessions to 2–3 per week. At least for special football player populations like the veteran players, such changes may be of great merit. Applying the programme in the "real-life situation" of veteran footballers is another challenge. In this study, supervision and instructions by qualified personnel were provided. However, in the "real life," an individual or team-based education through media (videos, posters, etc.) and disseminators would be necessary.

Limitations of the study

Compared with the chosen parallel design, a cross-over design might appear more appropriately at the first glance because compared samples would be dependent ones. But the unavoidable doubling of the study duration would have led to the considerable risk that more players drop out after completing the first phase. This could be due to the perception of a too long study period or due to the denial to conduct the control phase after "FIFA 11+" when participants were convinced of a benefit from the "FIFA 11+" intervention.

Despite the randomisation, there were significant group differences in age and BMI with slightly higher values in the intervention group than in the control group, a common problem in cluster-randomised studies. However, these differences were minor (on average 2 years and 1 $kg \cdot m^{-2}$) and their practical impact seems negligible.

All participating teams had neither a coach nor medical staff. Instead, they had players (team advisors) responsible for organising the training and match schedule. Therefore, it is possible that the extra work involved in registering and reporting the data, together with the non-professional organisation in the teams, led to a lack of reporting. However, also other authors of similar football studies mentioned a lack of reporting or poor communication with the teams, even if the structure of the teams was more professional (Soligard et al., 2008, 2010; Steffen, Emery, et al., 2013). To avoid a lack of reporting, we contacted each team at least monthly, but it cannot be ruled out that overall reporting

of data (including that of injuries) was incomplete in some cases.

In terms of the lower number of players in the control group, although the number of teams was the same, we believe that the fact of an instructed “FIFA-intervention” may have attributed to this higher participation rate in the intervention group. As previously reported by Soligard et al. (2008), we also noticed the disappointment of being randomised to the control group in some teams and players.

While respecting the “real-life” conditions, the veteran football players practised “FIFA 11+” maximally once per week, which may be considered as the main cause for the lack of preventive effects found in this study. Basically all successful neuromuscular programmes, in terms of injury prevention, were performed 2 or 3 times a week (Gilchrist et al., 2008; Soligard et al., 2008; Steffen et al., 2008; Steffen, Emery, et al., 2013; Walden et al., 2012).

The study period of one season (9 months) seemed to represent an adequate and common time period in an injury prevention intervention study. However, the training setting for a prevention study differed somewhat from the ones who have been reported to be successful: Effective prevention programmes have usually included 2–3 training sessions a week for 6–8 weeks. This has to be considered in future studies with veteran footballers.

The higher age, the significant injury/surgery history and the questionable fitness level may represent additional challenges for effective injury prevention in this veteran population.

Conclusion

“FIFA 11+” programme performed at the beginning of each training session did not result in a significant preventive effect on injury incidence in veteran football players. Only severe injuries and days of absence from training or match due to injury were significantly lower in the intervention group than in the control group. The lack of preventive effects on injury incidence is likely to be attributed to the low overall number and the frequency of neuromuscular training sessions, as these two factors are critical to neuromuscular adaptations. Because our data indicate considerable injury incidences in this population, development of an effective prevention strategy is desirable for the future.

References

Arnason, A., Andersen, T. E., Holme, I., Engebretsen, L., & Bahr, R. (2008). Prevention of hamstring strains in elite soccer: An intervention study. *Scandinavian Journal of Medicine and Science in Sports*, *18*(1), 40–48.

Arnason, A., Sigurdsson, S. B., Gudmundsson, A., Holme, I., Engebretsen, L., & Bahr, R. (2004). Risk factors for injuries in football. *American Journal of Sports Medicine*, *32*(Suppl. 1), 5S–16S.

Askling, C., Karlsson, J., & Thorstensson, A. (2003). Hamstring injury occurrence in elite soccer players after preseason strength training with eccentric overload. *Scandinavian Journal of Medicine and Science in Sports*, *13*(4), 244–250.

Coppack, R. J., Etherington, J., & Wills, A. K. (2011). The effects of exercise for the prevention of overuse anterior knee pain: A randomized controlled trial. *The American Journal of Sports Medicine*, *39*(5), 940–948.

Dauty, M., & Collon, S. (2011). Incidence of injuries in French professional soccer players. *International Journal of Sports Medicine*, *32*(12), 965–969.

Dvorak, J., & Junge, A. (2000). Football injuries and physical symptoms. *American Journal of Sports Medicine*, *28*(Suppl. 5), S-3–S-9.

Ekstrand, J., Hagglund, M., & Walden, M. (2011). Injury incidence and injury patterns in professional football: The UEFA injury study. *British Journal of Sports Medicine*, *45*(7), 553–558.

Emery, C. A., & Meeuwisse, W. H. (2010). The effectiveness of a neuromuscular prevention strategy to reduce injuries in youth soccer: A cluster-randomised controlled trial. *British Journal of Sports Medicine*, *44*(8), 555–562.

Faude, O., Junge, A., Kindermann, W., & Dvorak, J. (2006). Risk factors for injuries in elite female soccer players. *British Journal of Sports Medicine*, *40*(9), 785–790.

FIFA. (2013). Big Count 2006.

Finch, C. (2006). A new framework for research leading to sports injury prevention. *Journal of Science and Medicine in Sport*, *9* (1–2), 3–9.

Foster, C., Florhaug, J. A., Franklin, J., Gottschall, L., Hrovatin, L. A., Parker, S., & Dodge, C. (2001). A new approach to monitoring exercise training. *Journal of Strength and Conditioning Research*, *15*(1), 109–115.

Fuller, C. W., Ekstrand, J., Junge, A., Andersen, T. E., Bahr, R., Dvorak, J., & Meeuwisse, W. H. (2006). Consensus statement on injury definitions and data collection procedures in studies of football (soccer) injuries. *Scandinavian Journal of Medicine and Science in Sports*, *16*(2), 83–92.

Gatterer, H., Ruedl, G., Faulhaber, M., Regele, M., & Burtscher, M. (2012). Effects of the performance level and the FIFA “11” injury prevention program on the injury rate in Italian male amateur soccer players. *Journal of Sports Medicine and Physical Fitness*, *52*(1), 80–84.

Gilchrist, J., Mandelbaum, B. R., Melancon, H., Ryan, G. W., Silvers, H. J., Griffin, L. Y., & Dvorak, J. (2008). A randomized controlled trial to prevent noncontact anterior cruciate ligament injury in female collegiate soccer players. *The American Journal of Sports Medicine*, *36*(8), 1476–1483.

Herman, K., Barton, C., Malliaras, P., & Morrissey, D. (2012). The effectiveness of neuromuscular warm-up strategies, that require no additional equipment, for preventing lower limb injuries during sports participation: A systematic review. *BMC Medicine*, *10*, 75.

Hewett, T. E., Myer, G. D., & Ford, K. R. (2005). Reducing knee and anterior cruciate ligament injuries among female athletes: A systematic review of neuromuscular training interventions. *Journal of Knee Surgery*, *18*(1), 82–88.

Impellizzeri, F. M., Bizzini, M., Dvorak, J., Pellegrini, B., Schena, F., & Junge, A. (2013). Physiological and performance responses to the FIFA 11+ (part 2): A randomised controlled trial on the training effects. *Journal of Sports Sciences*. doi:10.1080/02640414.2013.802926

Junge, A., & Dvorak, J. (2004). Soccer injuries: A review on incidence and prevention. *Sports Medicine*, *34*(13), 929–938.

Downloaded by [Dicle University] at 02:21 17 November 2014

Downloaded by [Dicle University] at 02:21 17 November 2014

Junge, A., Lamprecht, M., Stamm, H., Hasler, H., Bizzini, M., Tschopp, M., … Dvorak, J. (2011). Countrywide campaign to prevent soccer injuries in Swiss amateur players. *The American Journal of Sports Medicine*, *39*(1), 57–63.

Kiani, A., Hellquist, E., Ahlqvist, K., Gedeborg, R., Michaelsson, K., & Byberg, L. (2010). Prevention of soccer-related knee injuries in teenaged girls. *Archives of Internal Medicine*, *170*(1), 43–49.

Krustrup, P., Aagaard, P., Nybo, L., Petersen, J., Mohr, M., & Bangsbo, J. (2010). Recreational football as a health promoting activity: A topical review. *Scandinavian Journal of Medicine and Science in Sports*, *20*, 1–13.

Krustrup, P., Christensen, J. F., Randers, M. B., Pedersen, H., Sundstrup, E., Jakobsen, M. D., … Bangsbo, J. (2010). Muscle adaptations and performance enhancements of soccer training for untrained men. *European Journal of Applied Physiology*, *108* (6), 1247–1258.

LaBella, C. R., Huxford, M. R., Grissom, J., Kim, K. Y., Peng, J., & Christoffel, K. K. (2011). Effect of neuromuscular warm-up on injuries in female soccer and basketball athletes in urban public high schools: Cluster randomized controlled trial. *Archives of Pediatrics and Adolescent Medicine*, *165*(11), 1033–1040.

Mandelbaum, B. R., Silvers, H. J., Watanabe, D. S., Knarr, J. F., Thomas, S. D., Griffin, L. Y., & Garrett Jr., W. (2005). Effectiveness of a neuromuscular and proprioceptive training program in preventing anterior cruciate ligament injuries in female athletes: 2-year follow-up. *American Journal of Sports Medicine*, *33*(7), 1003–1010.

Murphy, D. F., Connolly, D. A., & Beynnon, B. D. (2003). Risk factors for lower extremity injury: A review of the literature. *British Journal of Sports Medicine*, *37*(1), 13–29.

Myer, G. D., Stroube, B. W., DiCesare, C. A., Brent, J. L., Ford, K. R., Heidt Jr., R. S., & Hewett, T. E. (2013). Augmented feedback supports skill transfer and reduces high-risk injury landing mechanics: A double-blind, randomized controlled laboratory study. *The American Journal of Sports Medicine*, *41*(3), 669–677.

Myklebust, G., Engebretsen, L., Braekken, I. H., Skjølberg, A., Olsen, O.-E., & Bahr, R. (2003). Prevention of anterior cruciate ligament injuries in female team handball players: A prospective intervention study over three seasons. *Clinical Journal of Sport Medicine*, *13*(2), 71–78.

Peterson, L., Junge, A., Chomiak, J., Graf-Baumann, T., & Dvorak, J. (2000). Incidence of football injuries and complaints in different age groups and skill-level groups. *American Journal of Sports Medicine*, *28*(Suppl. 5), 51–57.

Prodromos, C. C., Han, Y., Rogowski, J., Joyce, B., & Shi, K. (2007). A meta-analysis of the incidence of anterior cruciate ligament tears as a function of gender, sport, and a knee injury-reduction regimen. *Arthroscopy: The Journal of Arthroscopic and Related Surgery*, *23*(12), 1320–1325.e6.

Randers, M. B., Nybo, L., Petersen, J., Nielsen, J. J., Christiansen, L., Bendiksen, M., & Krustrup, P. (2010). Activity profile and physiological response to football training for untrained males and females, elderly and youngsters: Influence of the number of players. *Scandinavian Journal of Medicine and Science in Sports*, *20*, 14–23.

Reis, I., Rebelo, A., Krustrup, P., & Brito, J. (2013). Performance enhancement effects of Internationale de Football Association's "The 11+" injury prevention training program in youth futsal players. *Clinical Journal of Sport Medicine*, *23*(4), 318–320.

Soligard, T., Myklebust, G., Steffen, K., Holme, I., Silvers, H., Bizzini, M., & Andersen, T. E. (2008). Comprehensive warm-up programme to prevent injuries in young female footballers: Cluster randomised controlled trial. *British Medical Journal*, *337*, a2469.

Soligard, T., Nilstad, A., Steffen, K., Myklebust, G., Holme, I., Dvorak, J., & Andersen, T. E. (2010). Compliance with a comprehensive warm-up programme to prevent injuries in youth football. *British Journal of Sports Medicine*, *44*(11), 787–793.

Steffen, K., Andersen, T. E., Krosshaug, T., Van Mechelen, W., Myklebust, G., Verhagen, E. A., & Bahr, R. (2010). ECSS position statement 2009: Prevention of acute sports injuries. *European Journal of Sport Science*, *10*(4), 223–236.

Steffen, K., Emery, C. A., Romiti, M., Kang, J., Bizzini, M., Dvorak, J., & Meeuwisse, W. H. (2013). High adherence to a neuromuscular injury prevention programme (FIFA 11+) improves functional balance and reduces injury risk in Canadian youth female football players: A cluster randomised trial. *British Journal of Sports Medicine*, *47*(12), 794–802.

Steffen, K., Meeuwisse, W. H., Romiti, M., Kang, J., McKay, C., Bizzini, M., & Emery, C. A. (2013). Evaluation of how different implementation strategies of an injury prevention programme (FIFA 11+) impact team adherence and injury risk in Canadian female youth football players: A cluster-randomised trial. *British Journal of Sports Medicine*, *47*(8), 480–487.

Steffen, K., Myklebust, G., Olsen, O. E., Holme, I., & Bahr, R. (2008). Preventing injuries in female youth football – a cluster-randomized controlled trial. *Scandinavian Journal of Medicine and Science in Sports*, *18*(5), 605–614.

Van Beijsterveldt, A. M., Van De Port, I. G., Krist, M. R., Schmikli, S. L., Stubbe, J. H., Frederiks, J. E., & Backx, F. J. (2012). Effectiveness of an injury prevention programme for adult male amateur soccer players: A cluster-randomised controlled trial. *British Journal of Sports Medicine*, *46*(16), 1114–1118.

Walden, M., Atroshi, I., Magnusson, H., Wagner, P., & Hagglund, M. (2012). Prevention of acute knee injuries in adolescent female football players: Cluster randomised controlled trial. *BMJ*, *344*, e3042.

Woll, A., & Dugandzic, D. (2007). *Strukturanalyse des Freizeit- und Breitensports und Sports der Älteren in deutschen Fußballvereinen Bericht 2007*. Frankfurt: Deutscher Fußball Bund (DFB).
