# Supplementary material for: Comparative Effectiveness of Multi-Component, Exercise-Based Interventions for Preventing Soccer-Related Musculoskeletal Injuries: A Systematic Review and Meta-Analysis
Source: Healthcare (Basel). 2025 Mar 29;13(7):765. doi: 10.3390/healthcare13070765 (PMC11988859; doi:10.3390/healthcare13070765)
Supplement: Supplementary file 1 [file healthcare-13-00765-s001.zip › Additional material Included literature/Hasebe2020.pdf]

Published online: 05.01.2020

Effects of Nordic Hamstring Exercise on Hamstring Injuries in High School Soccer Players: A Randomized Controlled Trial

Authors
Yuki Hasebe[1, 2], Kiyokazu Akasaka[2, 3], Takahiro Otsudo[2, 3], Yomei Tachibana[4], Toby Hall[5, 6], Mitsuru Yamamoto[1]

Affiliations
1 Department of Rehabilitation, Saitama Medical University Saitama Medical Center, Kawagoe, Saitama, Japan
2 Department of Physical Therapy, Saitama Medical University Graduate School of Medicine, Moroyama, Saitama, Japan
3 School of Physical Therapy, Saitama Medical University Faculty of Health and Medical Care, Moroyama, Saitama, Japan
4 Department of Orthopaedic Surgery, Saitama Medical University Hospital, Moroyama, Saitama, Japan
5 School of Physiotherapy and Exercise Science, Curtin University -Perth City Campus, Perth, Australia
6 Manual Concept, Perth, Australia

Key words
injury prevention, time-lost-to-sport-injury rate, 50 m running time, 10000 playing hours, Nordic Hamstring Exercise, hamstring injury

accepted 11.10.2019

Bibliography
DOI https://doi.org/10.1055/a-1034-7854
Published online: 2019
Int J Sports Med
© Georg Thieme Verlag KG Stuttgart · New York
ISSN 0172-4622

Correspondence
Prof. Kiyokazu Akasaka
Physical Therapy,
Saitama Medical University Graduate School of Medicine,
981 Kawakado, Moroyama, Iruma-gun,
Saitama 350-0495, Japan
Tel : + 81 49 295 1001, Fax : + 81 49 295 5104
akasaka-smc@umin.ac.jp

ABSTRACT

We evaluated a range of physical characteristics related to hamstring injuries, as well as the Nordic Hamstring Exercise compliance rate, and whether this influenced the rate hamstring injury. Subjects comprised 259 male soccer players from seven high schools randomly clustered into two groups, a Nordic Hamstring Exercise group and a control group. Training and match time were logged, as well as details of hamstring injury, and subsequent time lost to hamstring injury recorded over a period of 27 weeks. The Nordic Hamstring Exercise compliance rate, injury rate per 10000 playing hours and time-lost-to-sport-injury rate were calculated. The relative risk and hamstring injury severity were also calculated. The hamstring injury rate was 1.04/10 000 h in the control group and 0.88/10 000 h in the intervention group. The relative risk for hamstring injury was 1.14. The time-lost to injury rate was 1116.3/10 000 h in the control group and 113.7/10 000 h in the intervention group; with relative risk 9.81. The Nordic Hamstring Exercise in high school soccer players significantly reduced hamstring injury severity compared to a control intervention. Our results indicate that the time-lost to injury rate should be taken into account when analyzing the severity of hamstring injury.

Downloaded by: University of Toronto Libraries. Copyrighted material.

Introduction

Hamstring muscle injuries are frequently reported [1, 2] and are among the most common injuries in sports such as soccer and rugby where unexpected acceleration and sprinting are required [3–5]. Hamstring injuries typically occur during the late swing phase through to the initial contact phase of gait where hip flexion and knee extension occur simultaneously. Rapid acceleration and maximum velocity are associated factors [6–10]. According to recent research, soccer has one of the highest rates of musculoskeletal injuries among soccer, track and field, and rugby, with an overall injury rate of 37 %. With respect to soccer, hamstring injuries are the most common musculoskeletal injuries, with an injury rate of 12–16 % of all musculoskeletal injuries [11, 12]. It has also been reported that male amateur soccer has a hamstring injury rate of 20.4–36.9 per 1000 match hours and 2.4–3.9 per 1000 training hours [13, 14]. Furthermore, muscle injuries were more frequent in Japanese high school soccer

players, particularly following an ankle sprain or knee joint injury [15]. Risk factors for hamstring injuries have been reported to be diverse and include age, field position, muscle weakness, lack of flexibility, strength imbalance, and race [16]. However muscle weakness, lack of flexibility, and strength imbalance have not been established to measure in the field. Once injured, it takes 8–25 days to return to the performance level prior to hamstring injury [17]. In addition, the re-injury rate is reported as 12–13 % [2, 17, 18]. Therefore, prevention of hamstring injuries in high school soccer is considered to be an important issue. In recent years, it has been reported that the hamstring injury rate has decreased by up to 70 % through implementing the Nordic Hamstring Exercise (NHE) in professional and amateur soccer players [12, 19–22]. The NHE is considered practical because it can be implemented without any equipment. Preventing hamstring injuries not only reduces the injury rate but also reduces medical costs and mental suffering of players [12]. However, it is thought that professional and amateur players and high school students differ in their access to medical staff, competition level, training intensity and time, and prevention method compliance rate [23], and it is unknown whether NHE is as effective in high school soccer players. Currently, there are few studies examining factors affecting hamstring injuries among young soccer players and the effects of introducing a hamstring injury prevention program. The purpose of this study was to investigate whether physical examination variables correlate with hamstring injuries in high school soccer players and whether the NHE and its compliance rate affect the hamstring injury rate.

Materials and Methods

Subjects

The subjects were 259 male high school soccer club level players from 7 schools. The characteristics of the subjects are shown in ▶ **Table 1**. Inclusion and exclusion criteria and consent forms were mailed in an explanatory document to all the top 16 schools in the Saitama Prefecture Football League in the previous season. Inclusion criteria were male soccer club members belonging to the top 16 schools of all 138 schools in Saitama Prefecture High School Football League. Consents from both clubs and players were necessary to be included. Exclusion criteria were players (1) who had hamstring injuries at the start of the study, and (2) who had a history of surgery in the lumbar spine or lower limbs at the time when consent was obtained. After the explanation to the subjects in November 2016, a function evaluation was carried out in December 2016 and the investigation period was January to July 2017. We confirmed that no subject had received hamstring injury prevention training prior to the start of this study.

Randomization

In order to unify the competition level of the players as much as possible, we targeted the teams at the top of the Saitama Prefecture Football League. We used cluster-randomization using teams as the unit of cluster. Normal randomization was not employed because of the risk of contamination for our allocation. Randomization was achieved using the envelope method. We assigned players to 4 intervention groups (156 players) and 3 control groups (103 players) (▶ **Fig. 1**).

Measurement variables

The measurement variables were (1) functional evaluation (finger-floor distance, isometric knee extension and flexion strength, and 50 m running time); and (2) survey recording (training and match hours per day, details of injury, rest hours due to injury, presence or absence of NHE program). Measurement of basic information questionnaires and physical function evaluation was conducted at the start of the study.

Measurement

The physical function evaluation and survey recording items were used to identify risk factors for hamstring injury and were based on previous studies [16, 19, 24]. For hamstring injuries, reduced hamstring flexibility and low hamstring/quad strength ratio (HQ ratio) are considered high risk factors for injury, so we measured finger-floor distance as well as isometric knee extension and flexion strength. In addition, we measured 50 m running time, because rapid acceleration and maximum velocity during running have been shown to be associated hamstring injury factors. Measurement of finger-floor distance was performed with the subject standing and bending forward with the knees extended, and the distance between the floor and the middle finger was measured. The HQ ratio has been reported to be a factor related to hamstring injuries [25].

▶ **Table 1** Subject characteristics. (N = 259).

		Control (N = 103)	Intervention (N = 156)
Age (years)		16.3 ± 0.6 (15–17)	16.7 ± 0.5 (15–18)
Height (cm)		171.0 ± 5.3 (160.0–183.0)	171.0 ± 5.0 (155.0–188.0)
Weight (kg)		61.5 ± 5.4 (50.0–75.0)	61.4 ± 5.7 (48.0–76.0)
Soccer experience(years)		10.1 ± 2.4 (4–14)	10.1 ± 2.2 (4–14)
Dominant foot	Right	91	128
	Left	12	28
Field position(players)	Forward	19	22
	Midfielder	30	52
	Defender	33	55
	Keeper	11	9

Mean ± SD (range).

Downloaded by: University of Toronto Libraries. Copyrighted material.

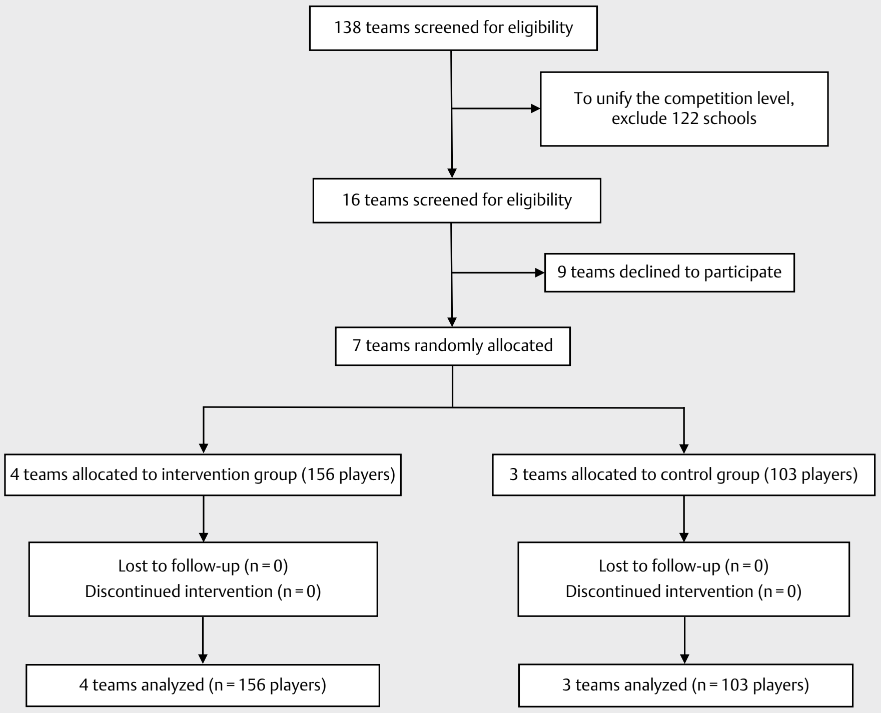

▶ **Fig. 1** Flow of participants through the trial.

Isometric knee extension and flexion muscle strength were measured using a manual muscle testing meter. Knee extension was assessed at 90 ° knee flexion in the sitting position, while knee flexion was examined at 30 ° flexion in the prone position. During strength testing, the upper limbs were placed across the chest. Measurement was carried out twice for maximum voluntary contraction for 5 seconds for each trial, and the maximum value was adopted for data analysis. The length of the lower leg was measured from the knee joint to the center of the lateral malleolus, and the knee extension and flexion muscle strength values were normalized. From these results, the HQ ratio was calculated. The 50 m run time was measured twice using a stopwatch. The fastest time was adopted for data analysis. Functional evaluation was measured at each school. Evaluation of all physical functions was carried out by the research team and the measurer was blinded to group allocation. Participants recorded the attendance and duration of training and matches, and whether they undertook the NHE program. With respect to hamstring injuries, we recorded the presence or absence of injury, time and number of days off from training or matches for more than one day due to injury, and details of the injury. For each high school, a manager was elected to check whether records were correctly taken. In addition, a member of the research team contacted each school recordkeeper at least once every two weeks and by mail every month, and conducted a school visit every two months.

NHE

The intervention group performed the NHE, with exercise compliance and hamstring injury recorded over a 27-week period. The NHE was undertaken after normal training and before cool-down. The athlete started in a kneeling position, with his torso from the knees upward held rigid and straight. A training partner stabilized the athlete's feet to keep in contact with the ground throughout the movement. The athlete then attempted to resist a forward-falling motion using his hamstring muscles to maximize loading in the eccentric phase. The participants were asked to brake the forward fall for as long as possible. The athletes were asked to use their arms and hands to buffer the fall, let the chest touch the surface, and immediately get back to the starting position by pushing with their hands to minimize loading in the concentric phase [12, 26] (▶ **Fig. 2**). Players and coaching staff were taught the correct application of the exercise by one researcher. During the intervention period, the coach and department manager oversaw the exercise. The NHE protocol was created to gradually increase from the match preparation period to the match period with reference to the previous study [12] (▶ **Table 2**). Subjects who developed hamstring injuries during the intervention period discontinued NHE until the injury had recovered.

Analysis method

The definition of a hamstring injury was a state in which pain was felt in the posterior thigh regardless of time loss or need for treatment during sports activities [27]. Only injuries reported by players were counted as hamstring injuries. To present the data, the NHE compliance rate, hamstring injury rate, time-lost-to-sport days, and time-lost-to-sport rate were calculated based on NHE and survey records as follows. Compliance rate with recordkeeping and the NHE program was calculated based on the actual record

Downloaded by: University of Toronto Libraries. Copyrighted material.

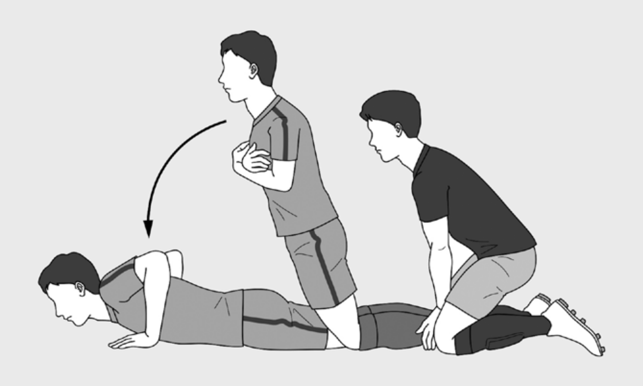

▶ **Fig. 2** The Nordic Hamstring Exercise.

▶ **Table 2** Implementation protocol of Nordic Hamstring Exercise.

Weeks	Frequency per week	No. of sets per session	Repetitions per set
1	1	5	2
2–3	2	6	2
4–6	2	6	3
7–9	2	6, 7, 8	3
10–13	2	8, 9, 10	3
14–27	2	10, 9, 8	3

and NHE implementation days, divided by scheduled records and NHE implementation days, respectively. The hamstring injury rate was calculated based on the number of hamstring injuries per 10 000 h in which one player participated in training and matches. The time-lost-to-sport hours were taken as the number of hours that the training or matches was missed due to injury. The time-lost-to-sport-injury rate was calculated based on the number of time-lost-to-sport days per 10 000 h of training and match time. The relative risk (RR) was calculated based on the number of injuries and the time-lost-to-sport hours.

Statistical analysis

For statistical analysis, SPSS Statistics Version 25.0 (IBM Corp., Armonk, NY, USA) was used. For physical function evaluation comparing the intervention group and the control group, comparison between the presence or absence of hamstring injuries and the physical function evaluation, an independent t-test was carried out after confirming normality and homoscedasticity. The level of significance was set at 5 %.

Sample size

To decide on an adequate sample size to accomplish statistical significance with 80 % power (1−β), we performed priori power analyses. Sample size was calculated by power analysis application (G * Power 3.1.9.2, http://www.gpower.hhu.de/). In order to compare the number of hamstring injuries among both groups, the effect size w was set to 0.3 ($\alpha = 0.05$, $1-\beta = 0.8$) in the χ 2 test, resulting in 143 cases. In addition, as a result of post hoc test of a previous study on hamstring injury prevention [20], the effect size was 0.26. From this result, we tested with an effect size of 0.26 ($\alpha = 0.05$, $1-\beta = 0.5$), and the sample size was 107 cases. Therefore, we tried to recruit at least 190 subjects at the beginning of this study.

Ethical considerations

This study was conducted with the approval of ethics review committee at Faculty of health and medical care of Saitama Medical University (M-71). Participation in this research was contingent on the consent of the principal of each participating high school and the head of the soccer club. In addition, for all subjects, written consent was obtained from each player and their parent. The study meets the ethical standards of the International Journal of Sports Medicine [28].

Results

The compliance rate for recordkeeping and the NHE in this study were 100 and 88 %, respectively. In the comparison of the physical function evaluation between the control group and the intervention group, the right HQ ratio was 0.73 ± 0.21 in the control group and 0.64 ± 0.17 in the intervention group, and the left HQ ratio was 0.73 ± 0.25 in the control group and 0.60 ± 0.15 in the intervention group. Mean 50 m running time was 6.92 ± 0.35 in the control group and 6.73 ± 0.28 in the intervention group. The HQ ratio and 50 m running time were significantly different between the two groups ($p < 0.01$), with no significant difference between groups for the finger-floor distance.

Hamstring injuries occurred in 7 players (3 in the control group, 4 in the intervention group) (▶ **Table 3**), and 252 players reported no injuries. All hamstring-injured players had never injured their hamstring before. When comparing the physical function variables in those with and without hamstring injuries, there was no significant difference in the left and right HQ ratio, or finger-floor distance, but there was for the 50 m running time ($p = 0.03$).

In addition to hamstring injuries, there were 84 participants reporting low back or lower limb pain during the study period (37 in the control groups, 47 in the NHE groups). In those with hamstring injuries, 4 participants reported time-lost-to-sport (2 in the control group and 2 in the NHE groups). The total time lost was 50 days (95 h) in the control group and 6 days (12 h) in the intervention group. The hamstring injury rate was 1.04 in the control group and 0.88 in the NHE group, and the relative risk was 1.14. In addition, the hamstring injury rate with time-lost-to-sport was 0.69 in the control group and 0.44 in the intervention group, and the relative risk was 1.52. The time-lost-to-sport-injury rate for hamstring injuries was 1116.3 in the control group and 113.7 in the intervention group, and the relative risk was 9.81 (▶ **Tables 4**, **5**).

Discussion and Conclusions

Compliance for recordkeeping was 100 %, whereas the compliance rate with the NHE protocol was 88 %. In this study, we designed the recording process as simple to maintain and appointed one of the participants to be a recordkeeper for a high compliance rate on a regular basis. In addition, we posted the recording sheet in the club room, so that omission of entry was recognized by other participants. Finally, we visited the school frequently to ensure continued implementation of the NHE according to the study protocol.

Downloaded by: University of Toronto Libraries. Copyrighted material.

▶ **Table 3** Low back and lower limb injuries during the investigation period (N = 259).

	n	No. of injuries	Competition hours	Injury rate/10 000 competition hours	RR* (95 %CI)	P value
Lumbar						
Control	103	6	28 910	2.08	1.01 (0.37–2.75)	0.93
Intervention	156	9	45 374	1.98		
Hip joint						
Control	103	3	28 910	1.04	4.54 (0.48–43.09)	0.17
Intervention	156	1	45 374	0.22		
Knee joint						
Control	103	5	28 910	1.73	0.76 (0.27–2.15)	0.66
Intervention	156	10	45 374	2.20		
Hamstring injuries						
Control	103	3	28 910	1.04	1.14 (0.26–4.97)	0.83
Intervention	156	4	45 374	0.88		
Ankle joint						
Control	103	20	28 910	6.92	1.32 (0.76–2.27)	0.31
Intervention	156	23	45 374	5.07		

*RR: relative risk

▶ **Table 4** Injury rate following hamstring injury (N = 259).

	n	No. of injuries	Competition hours	Injury rate/1000 competition hours	Injury rate RR* (95 %CI)	P value
All injuries						
Control	103	43	28 910	14.87	1.10 (0.81–1.50)	0.50
Intervention	156	59	45 374	13.00		
Hamstring injuries						
Control	103	3	28 910	1.04	1.14 (0.26–4.97)	0.83
Intervention	156	4	45 374	0.88		
Hamstring injuries with time-lost-to-sport						
Control	103	2	28 910	0.69	1.52 (0.22–10.58)	0.51
Intervention	156	2	45 374	0.44		

*RR: relative risk

▶ **Table 5** Time-lost-to-sport injury rate following hamstring injury (N = 259).

	n	Time-lost-to-sport days with injury	Competition hours	Time-lost-to-sport injury rate/10 000 competition hours	Time-lost-to-sport injury rate RR* (95 %CI)	P value
Hamstring injuries with time-lost-to-sport						
Control	103	95	851	1116.3	9.81 (5.42–17.8)	<0.001
Intervention	156	12	1 055	113.7		

*RR: relative risk

NHE is considered to affect the significantly improved knee flexor strength [26] and substantial increase of sprint performance [29], increased length of long head of the biceps femoris [30], and shifting the knee flexion maximum torque towards extension [31]. Despite this evidence, there was no relationship between HQ ratio and hamstring injury or between HQ ratio and finger-floor distance in our study. The following points are conceivable. Previous studies have measured the HQ ratio based on isotonic testing [25, 26, 32]. Perhaps the current study's isometric measurement method was not relevant. Also, in the assessment of the hamstring flexibility, the finger-floor distance also evaluates the flexibility factor of the erector spinae muscle, and therefore might not be specific to the hamstring muscles. Hence, the physical function measures in this study were insufficient to determine the risk factors for hamstring injury. Future studies should investigate other physical function parameters such as the straight leg raise test. On the other hand, in terms of the physical parameters evaluated, the NHE group showed a significantly reduced 50 m running time than the control group. It has been reported that the extensibility to hamstring increases and the amount of lower limb muscle activity significantly

Downloaded by: University of Toronto Libraries. Copyrighted material.

increases as the running speed increases. However, it was considered that the time-lost-to-sport rate reduced in the intervention group in those who could run at the fast speed. The possibility was that the hamstring injuries with high severity could be prevented in our study.

The hamstring injury rate in the NHE group was 1.14 times lower than in the control group, and the hamstring injury rate of time-lost-to-sport showed a reduction with 1.52 times less time lost in the NHE group compared to the control group. These data suggest a positive effect of reducing hamstring injuries associated with the NHE. However, caution is required in interpreting these results, and the hamstring injury incidence rate in this study was small compared to previous studies [12, 19, 24, 33, 34]. The hamstring injury rate in the junior generation was unclear in previous studies. Because it was difficult to predict the number of samples needed for this study, the sample size was difficult to select before the study started. Risk factors for hamstring injuries usually include physical factors such as age, hamstring flexibility, muscle strength, and imbalance of muscle strength. Environmental factors were also included such as competition level and playing field condition, which overlap physical factors [16, 19, 30]. It has been reported that the flexibility of the body decreases [35] especially with age, which suggests that the muscle injury rate increases with age. The above environment is considered different in high school soccer players compared to amateur or professional players. These factors might explain the low levels of hamstring injury and therefore low level effect of the NHE on hamstring injury rate. On the other hand, the time-lost-to-sport rate for hamstring injury was shown to be very effective, and was 9.81 times lower in the NHE group than control group. A high rate of hamstring injuries in soccer players is generally reported at all levels of sport, including in adolescent soccer players. Despite this, there is a lack of research comparing the effects of injury prevention exercise at different levels of sport. The results of this study suggest that the NHE has an effect to reduce injury severity in high school soccer players. However, because the HQ ratio between the two groups was different at the start of the study, the possibility of affecting the results cannot be ruled out and should be considered in the interpretation.

A survey of the effects of an injury prevention training program on amateur soccer players reported that injury severity was reduced after implementation of the NHE program, thereby reducing medical costs and mental suffering of athletes [36, 37]. From the present study, the time-lost-to-sport rate is an index reflecting not only the severity of injury but also the environmental influence to which the athlete is subjected. The NHE in this study also showed similar effects to previous studies, such as reducing the severity of injury even in high school soccer players who typically have long training periods. The athletes' competition level is high and players do not like to take breaks from training and matches, and will even hide minor injuries. There is also a lack of medical support staff, and management staff tends to prioritize improvement of skills over injury prevention. Furthermore, there is low self-management ability, potentially causing problems for prolonging time-lost-to-sport. As a result, many players have less than optimal care at the time of injury and often continue to play sport afterwards. This can make the injury more severe and increase time-to-return to the sport. The current results suggest that the NHE may influence reducing time lost to sport much more than the incidence of injury.

Acknowledgements

We would like to thank coaches and players of the high school soccer club in Saitama for their valuable collaboration for this study. There has been no financial assistance with this study.

Conflict of Interest

The authors declare no conflict of interest.

References

[1] Ekstrand J, Hägglund M, Waldén M. Epidemiology of muscle injuries in professional soccer (soccer). Am J Sports Med 2011; 39: 1226–1232

[2] Woods C, Hawkins RD, Maltby S et al. The Soccer Association Medical Research Programme: an audit of injuries in professional soccer–analysis of hamstring injuries. Br J Sports Med 2004; 38: 36–41

[3] Bennell KL, Crossley K. Musculoskeletal injuries in track and field: Incidence, distribution and risk factors. Aust J Sci Med Sport 1996; 28: 69–75

[4] Feeley BT, Kennelly S, Barnes RP et al. Epidemiology of National Soccer League training camp injuries from 1998 to 2007. Am J Sports Med 2008; 36: 1597–1603

[5] Orchard J, Seward H. Epidemiology of injuries in the Australian Soccer League, seasons 1997–2000. Br J Sports Med 2001; 29: 303–303

[6] Askling CM, Tengvar M, Saartok T. Thorstensson Alf. Acute first-time hamstring strains during high-speed running. Am J Sports Med 2007; 35: 197–206

[7] Montgomery WH, Pink M, Perry J. Electromyographic analysis of hip and knee musculature during running. Am J Sports Med 1994; 22: 272–278

[8] Sherry MA, Best TM. A comparison of 2 rehabilitation programs in the treatment of acute hamstring strains. J Orthop Sports Phys 2004; 34: 116–125

[9] Verrall GM, Slavotinek JP, Barnes PG et al. Clinical risk factors for hamstring muscle strain injury: a prospective study with correlation of injury by magnetic resonance imaging. Br J Sports Med 2001; 35: 435–439

[10] Wood GA. Biomechanical limitations to sprint running. In: Van Gheluwe B, Atha J (Eds). Current Research in Sports Biomechanics. Selected Topics. Medicine and Sport Science, Karger, Basel 1987; 25: 58–71

[11] Hawkins RD, Fuller CW. A prospective epidemiological study of injuries in four English professional soccer clubs. Br J Sports Med 1999; 33: 196–203

[12] Petersen J, Thorborg K, Nielsen MB et al. Preventive effect of eccentric training on acute hamstring injuries in men's soccer: A cluster-randomized controlled trial. Am J Sports Med 2011; 39: 2296–2303

[13] Kordi R, Hemmati F, Heidarian H et al. Comparison of the incidence, nature and cause of injuries sustained on dirt field and artificial turf by amateur football players. Sports Med Arthrosc Rehabil Ther Technol 2011; 3: 3

[14] Van Beijsterveldt AMC, Stubbe JH, Schmikli SL et al. Differences in injury risk and characteristics between Dutch amateur and professional soccer players. J Sci Med Sport 2015; 18: 145–149

Downloaded by: University of Toronto Libraries. Copyrighted material.

[15] Kawagoe T, Yamamoto Y, Suga Y. A study of injuries of soccer player in school students. The Hokkaido Journal of Physical Therapy 2002; 19: 102–104

[16] Freckleton G, Pizzari T. Risk factors for hamstring muscle strain injury in sport: A systematic review and meta-analysis. Br J Sports Med 2013; 47: 351–358

[17] Hägglund M, Walde n M, Ekstrand J. Injuries among male and female elite football players. Scand J Med Sci Sports 2009; 19: 819–827

[18] Heiderscheit BC, Sherry MA, Silder A et al. Hamstring strain injuries: recommendations for diagnosis, rehabilitation, and injury prevention. J Orthop Sports Phys 2010; 40: 67–81

[19] Arnason A, Andersen TE, Holme I et al. Prevention of hamstring strains in elite soccer: An intervention study. Scand J Med Sci Sports 2008; 18: 40–48

[20] Van der Horst N, Smits DW, Petersen J et al. The preventive effect of the Nordic hamstring exercise on hamstring injuries in amateur soccer players: Study protocol for a randomised controlled trial. Am J Sports Med 2015; 43: 1316–1323

[21] Al Attar WSA, Soomro N, Pappas E et al. Adding a post-training FIFA 11 + exercise program to the pre-training FIFA 11 + injury prevention program reduces injury rates among male amateur soccer players: a cluster-randomised trial. J Physiother 2017; 63: 235–242

[22] Al Attar WSA, Soomro N, Sinclair PJ et al. Effect of injury prevention programs that include the Nordic Hamstring Exercise on hamstring injury rates in soccer players: A systematic review and meta-analysis. Sports Med 2017; 47: 907–916

[23] Al Attar WSA, Soomro N, Sinclair PJ et al. Implementation of an evidence-based injury prevention program in professional and semi-professional soccer. International Journal of Sports Science and Coaching 2018; 13: 113–121

[24] Engebretsen AH, Myklevust G, Holme I et al. Intrinsic risk factors for hamstring injuries among male soccer player: A prospective cohort study. Am J Sports Med 2010; 38: 1147–1153

[25] Croisier JL, Ganteaume S, Binet J et al. Strength imbalances and prevention of hamstring injury in professional soccer players: a prospective study. Am J Sports Med 2008; 36: 1469–1475

[26] Mjølsnes R, Arnason A, Østhagen T et al. A 10-week randomized trial comparing eccentric vs. concentric hamstring strength training in well-trained soccer players. Scand J Med Sci Sports 2004; 14: 311–317

[27] Fuller CW, Ekstrand J, Junge A et al. Consensus statement on injury definitions and data collection procedures in studies of football (soccer) injuries. Br J Sports Med 2006; 40: 193–201

[28] Harriss DJ, Macsween A, Atkinson G. Standards for ethics in sport and exercise science research: 2020 update. Int J Sports Med 2019; 40: 813–817

[29] Suarez-Arrones L, Saez de Villarreal E, Núñez FJ et al. In-season eccentric-overload training in elite soccer players: Effects on body composition, strength and sprint performance. PLoS One 2018; 13: e0205332. doi:10.1371/journal.pone.0205332

[30] Bourne MN, Duhig SJ, Timmins RG et al. Impact of the Nordic hamstring and hip extension exercises on hamstring architecture and morphology: Implications for injury prevention. Br J Sports Med 2017; 51: 469–477

[31] Clark R, Bryant A, Culgan JP et al. The effects of eccentric hamstring strength training on dynamic jumping performance and isokinetic strength parameters: A pilot study on the implications for the prevention of hamstring injuries. Phys Ther Sport 2005; 6: 67–73

[32] Yeung SS, Suen AM, Yeung EW. A prospective cohort study of hamstring injuries in competitive sprinters: Preseason muscle imbalance as a possible risk factor. Br J Sports Med 2009; 43: 589–594

[33] Peterson L, Junge A, Chomiak J et al. Incidence of soccer injuries and complaints in different age groups and skill-level groups. Am J Sports Med 2000; 28: 51–57

[34] Sullivan JA, Gross RH, Grana WA et al. Evaluation of injuries in youth soccer. Am J Sports Med 1980; 8: 325–327

[35] Kinugasa T, Nagasaki H, Ito H et al. Effect of aging on motor ability in men aged 18–83 years. Jpn J Phys Fit Sports Med 1994; 43: 343–351

[36] Junge A, Lamprecht M, Stamm H et al. Countrywide campaign to prevent soccer injuries in Swiss amateur players. Am J Sports Med 2011; 39: 57–63

[37] Krist MR, Beijsterveldt AMC, Backx FJG et al. Preventive exercises reduced injury-related costs among adult male amateur soccer players: a cluster-randomised trial. J Physio 2013; 59: 15–23

Downloaded by: University of Toronto Libraries. Copyrighted material.
