# Supplementary material for: Comparative Effectiveness of Multi-Component, Exercise-Based Interventions for Preventing Soccer-Related Musculoskeletal Injuries: A Systematic Review and Meta-Analysis
Source: Healthcare (Basel). 2025 Mar 29;13(7):765. doi: 10.3390/healthcare13070765 (PMC11988859; doi:10.3390/healthcare13070765)
Supplement: Supplementary file 1 [file healthcare-13-00765-s001.zip › Additional material Included literature/Owoeye 2014.pdf]

©Journal of Sports Science and Medicine (2014) **13**, 321-328
http://www.jssm.org

Research article

Efficacy of the FIFA 11+ Warm-Up Programme in Male Youth Football: A Cluster Randomised Controlled Trial

Oluwatoyosi B. A. Owoeye [1]✉, Sunday R. A. Akinbo [1], Bosede A. Tella [1] and Olajide A. Olawale [2]
[1] Orthopaedic/Sports Physiotherapy and [2] Neuro-Physiotherapy Units, Department of Physiotherapy, Faculty of Clinical Sciences, College of Medicine, University of Lagos, Nigeria

Abstract
The FIFA 11+ is a structured warm-up programme specially designed to prevent injuries among football players from age 14 years and above. However, studies to prove its efficacy are generally few and it is yet to be tested in male youth footballers and among African players. The purpose of the study was to examine the efficacy of the FIFA 11+ programme in reducing the risk of injuries among male youth football players of the Lagos Junior League. A cluster randomised controlled trial was conducted. All the 20 teams (414 players aged 14 -19 years) in the Premier League division were block-randomised into either an intervention (INT) or a control (CON) group. The INT group performed the FIFA 11+ exercises as warm-up during training sessions and the CON group performed usual warm-up. Participating teams were prospectively followed through an entire league season of 6 months in which they were visited every week to assess injured players for time-loss injuries in both groups. The primary outcomes were any injury to the players, injuries by type of exposure and injuries specific to the lower extremities. The secondary outcomes were injuries reported by body location, aetiology, mechanism and severity. In total, 130 injuries were recorded affecting 104 (25%) of the 416 players. Team and player compliance with the INT was 60% and 74% respectively. Based on the primary outcome measures of the study, the FIFA 11+ programme significantly reduced the overall rate of injury in the INT group by 41% [RR = 0.59 (95% CI: 0.40 – 0.86; p = 0.006)] and all lower extremity injuries by 48% [RR = 0.52 (95% CI: 0.34 – 0.82; p = 0.004)]. However, the rate of injury reduction based on secondary outcomes mostly did not reach the level of statistical significance. The FIFA 11+ programme is effective in reducing the rates of injuries in male youth football players.

Key words: Injury prevention, neuromuscular training, FIFA, sports.

Introduction

Football (soccer) is the world's most popular sport. According to the Federation Internationale de Football Association (FIFA), there are 265 million male and female registered football players worldwide and of these, 46 million (17%) are from Africa; third largest after Asia (33%) and Europe (23%) (FIFA, 2006). Furthermore, 56.3% and 69.5% of all registered players are youth of 18 years and below globally and in Africa respectively (FIFA, 2006).

Africa is a continent with major developmental challenges like poverty, hunger, infectious and non-infectious diseases. Over the last decade, the use of sport as a tool to achieve development goals has gained immense support worldwide and football is the sport most frequently used. Hence, football may serve as a tool for reducing crime and poverty among young players. Furthermore, participation in football is an effective way for youths to increase their level of physical activity and fitness.

It has being emphasized that prevention of injuries should always be a priority and is even more important when treatment possibilities are restricted, as in many parts of Africa (Constantinou, 2010). Football players in Africa are faced with huge challenges. Funding is generally low and medical support appears poor especially in youth football and at the grass-roots. A recent study revealed an inadequacy in the medical coverage in a cohort of male youth football players in Lagos, Nigeria (Owoeye et al., 2013). The study further exposed that players rarely had qualified medical attendants; as masseurs predominantly attended to players during training and matches (Owoeye et al., 2013). Thus, the importance of injury prevention in youth football players in the region cannot be overemphasized. Implementation of injury prevention programmes in this extremely vulnerable population of disadvantaged youths is highly imperative.

In order to abate the high risk of injuries in football, FIFA's Medical Assessment and Research Centre (F-MARC) has developed a neuromuscular injury-prevention programme, referred to as FIFA 11+, to help reduce the incidence of injuries in football (F-MARC, 2013). The FIFA 11+ is a comprehensive warm-up programme developed by F-MARC and experts in the field of football medicine to reduce injuries among male and female football players aged 14 years and above. Sadly, implementation of this programme appears to be limited in the African region. Furthermore, there seems to be a low level of awareness of the FIFA 11+ programmes among players and coaches. Over three-quarters (79.3%) of male youth football players in Lagos, Nigeria were reported not to be aware of FIFA 11+ (Owoeye et al., 2013).

Several studies have been conducted to test the efficacy of injury prevention programmes in football (Aaltonen et al., 2007; Alentorn-Geli et al., 2009; Arnason et al., 2008; Askling et al., 2003; Emery and Meeuwisse, 2010; Grooms et al., 2013; Heidt et al., 2000; Junge and Dvorak, 2004; Peterson et al., 2011; Soligard et al., 2008; Steffen et al., 2013; Walden et al., 2012). However, to our knowledge, none of these programmes have been tested in an African population and most studies were conducted on female players. Specific injury prevention programmes

Received: 17 October 2013 / Accepted: 22 December 2013 / Available (online): 01 May 2014

such as the FIFA 11+, Prevent Injuries and Enhance Performance (PEP) programme and the Harmoknee Programme have been reviewed to be effective neuromuscular warm-up programmes requiring no additional equipment for preventing lower extremity (LE) injuries in football (Herman et al., 2012). These injury prevention programmes reduced the rate of LE injuries by 32 – 95% in female football players (Herman et al., 2012).

Other injury prevention programmes focusing on intrinsic risk factors have also been shown to reduce the risk of injuries in heterogeneous populations of football players (Aaltonen et al., 2007; Arnason et al., 2008; Askling et al., 2003; Emery and Meeuwisse, 2010; Junge and Dvorak, 2004; Peterson et al., 2011). In addition, a neuromuscular warm-up programme targeting core stability, balance, and proper knee alignment effectively reduced non-contact anterior cruciate ligament injuries by 64% in Swedish female football players (Walden et al., 2012). In another cohort study, the FIFA 11+ reduced the overall risk of LE injury in collegiate male football players in the United States of America by 72% (Groom et al., 2013).

The FIFA 11+ is a comprehensive warm-up programme aimed at improving muscular strength, body kinesthetic awareness, and neuromuscular control during static and dynamic movements. The FIFA 11+ is one of the few structured exercise based injury prevention programmes presently available in football. However, its efficacy is yet to be fully documented in literature as it has only been tested in randomised controlled trials (RCTs) in young female footballers in Norway (F-MARC, 2013; Soligard et al., 2008; Steffen et al., 2013). Soligard et al. (2008) reported an overall significant reduction of 32% in injury rate in teams that performed FIFA 11+ while Steffen et al. (2013) reported an overall significant injury reduction of 72% based on secondary data analysis. Randomised controlled trials on the efficacy of the FIFA 11+ injury prevention programme in reducing injury rate among young male football players are presently lacking and its potency in minimising the incidence of injuries in African players is unknown. We therefore conducted a cluster RCT to evaluate the effects of the FIFA 11+ programme on male youth football league players in Lagos, Nigeria.

Methods

Study design, participants and randomisation

A cluster randomised controlled trial was conducted. A cluster design was adopted so as to administer the INT programme to teams and not directly to individual players thereby minimising contamination between the INT and CON groups. Data collection procedures and definitions used in the present study were in accordance with international consensus guidelines for injury surveillance in football (Fuller et al., 2006).

The sample frame for this study consisted of 20 teams (416 players) of the Premier League division of the Lagos Junior League (LJL) registered for the 2012/2013 season. Blocked randomisation technique was used to equally allocate the 20 teams into 2 groups and teams were prospectively followed for a period of 6 months (September, 2012 to February, 2013) through the 2013/2013 league season.

The randomisation procedure for this study was based on team clusters with regards to the official list of premier league teams registered for the season. A randomisation block comprising 4 unlabelled teams was chosen based on the intended number of groups (two) (Kang et al., 2008). A total of 6 possible blocks were derived from a probability/balanced combination of teams from a list of 20 teams. Five blocks were randomly chosen and a random sequence was then used to assign prelisted teams into equal numbers per group. Ten teams were randomly allocated to an intervention (INT) group (212 players) and 10 teams to the control (CON) group (204 players) (Figure 1). All the 20 teams completed the study and all players with available data were involved in the final analysis based on the intention to treat principle (Mohar et al., 2010).

Intervention

The 11+" is a structured, exercise-based warm-up programme aimed at reducing injuries among male and female football players aged 14 years and above (Soligard et al., 2008). This neuromuscular training programme consists of three parts. The initial part is running exercises at slow speed combined with active stretching and controlled contacts with a partner. The running course includes six to ten pairs of cones (depending on the number of players) about five to six metres apart (length and width). The second part consists of six different sets of exercises; these include strength, balance, and jumping exercises, each with three levels of increasing difficulty. The final part is speed running combined with football specific movements with bounding and plant-cut movements.

The purpose and procedures of the study was explained to all the players and coaches of participating teams at a pre-participation screening programme. Furthermore, a pre-season workshop and practical session was organised for the head coaches and assistant coaches of the 10 teams in the INT group prior to the commencement of the 2012/2013 league season. Coaches of teams randomised into the INT group were instructed on the FIFA 11+ injury prevention warm-up exercise programme and to facilitate understanding, compliance, ongoing safety and correct execution, materials such as 11+ posters, booklet manuals and DVDs were also given to them as reference guide for implementing the injury prevention programme with their teams. The information material given to coaches in the INT group explains the proper form for each exercise, as well as common biomechanical mistakes. It also describes the principles of progression in the exercise prescription. The coaches in INT group were also instructed to follow F-MARC's recommendations for the 11+ which is to use the complete exercise programme as the warm up for at least two training sessions every week throughout the 2012/2013 league season. The CON group players on the other hand were instructed to continue with their usual non-structured warm-up during training periods with no additional training programme.

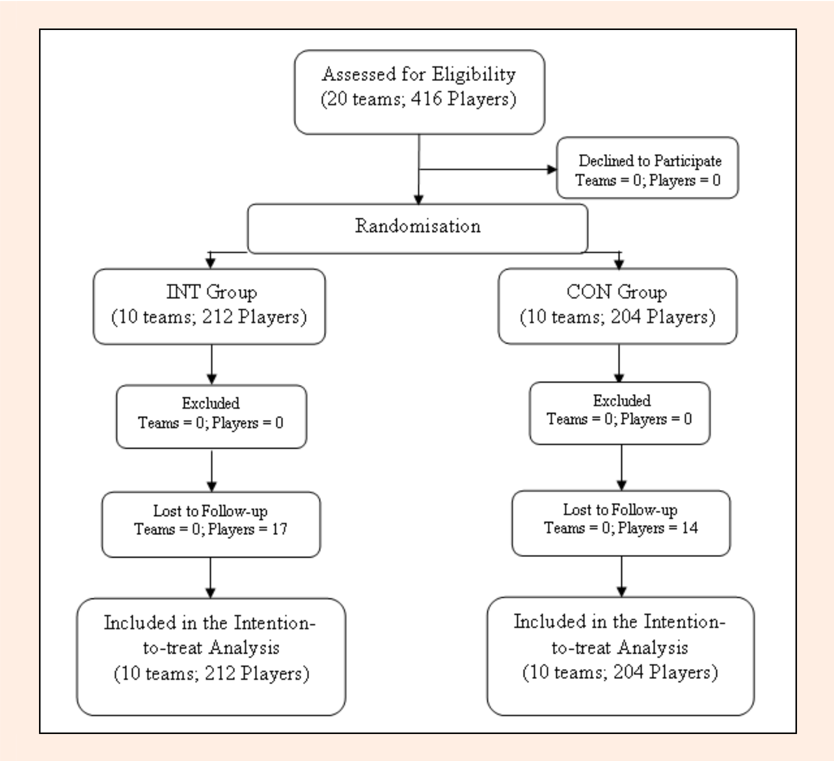

Figure 1. Randomisation and flow diagram of the study teams/players.

Data collection procedures

Baseline characteristics of players: Baseline characteristics of all the players were recorded at a pre-season fitness assessment programme. All 416 registered players in the INT and CON groups were invited to this mandatory programme prior to the commencement of the 2012/2013 league season. A pre-designed questionnaire targeted at documenting players' descriptive characteristics and selected physical fitness parameters was administered on all players.

Information on players' age, height, weight, body mass index and body fat were recorded. Information concerning players' injury history over the past 1-year period was also recorded. In addition, selected physical performance parameters were assessed.

A pre-assessment education and demonstration session was held for each participating team in order for players to understand the assessment protocols. Height was measured (to the nearest 0.5 cm) using a non-elastic measuring tape, fastened to a vertical wall, with the player standing on bare feet. Players' weight and body-fat percentage were measured using the Omron Body Composition Monitor. In assessing players' performance fitness, each player was asked to perform 3 different fitness tests - standing stork balance test on dominant leg, prone hold core stability test and dynamic leg strength according to standardized protocols (Kilding *et al*, 2008). Three trials of each assessment, except the prone hold (single trial), were performed and the mean of the trials was determined and used for analysis.

Injury, exposure and compliance reporting: The participating teams were visited on a weekly basis by 3 physiotherapists (1 of the authors and 2 research assistants) with experience in sports injury surveillance and with prior training on data collection procedures for the study. The physiotherapists visited the various teams at their training centres through the entire league season to assess players who met the inclusion for reportable injuries. Reportable injuries for this study were time-loss injuries defined as injuries that resulted in players being unable to fully participate in subsequent football training sessions or matches (Fuller et al., 2006). Players with time-loss injuries were assessed about a week after injuries were sustained and diagnosed based on study outcomes.

The physiotherapists compiled information about injuries vis-a-vis exposure, location, aetiology, mechanism and severity on injury report forms. Also, the physiotherapists interviewed the coaches to obtain details of team exposures in the previous week and players' participation in each training session and match on exposure forms. Number of matches played, training sessions observed and total number of players present (from team register) was documented for each team. Furthermore, weekly information on the extent of team and player compliance with the FIFA 11+ programme was obtained from the coaches in the INT group on separate compliance forms. Teams in the INT group were constantly reminded and encouraged through their coaches to implement the exercise programme through regular phone contacts by one of the authors and during weekly data collection by study physiotherapists at training venues. Data were collected on validated forms in accordance with consensus guidelines outlined for football injury research (Fuller et al., 2006).

Outcome measures

All injuries were reported in relation to primary and

Table 1. Baseline characteristics of players in both groups.

Variable	INT Group (n = 212)	CON Group (n = 204)	t – value/ χ^2	P – value
**Age (year)**	17.80 (.94)	17.49 (1.10)	.54	.593
**Height (m)**	1.72 (.57)	1.71 (.64)	1.16	.249
**Weight (kg)**	63.84 (6.07)	62.40 (6.68)	1.47	.145
**Body Mass Index (kg/m^2)**	21.82 (1.65)	21.22 (1.46)	1.92	.058
**Body Fat (%)**	14.09 (3.54)	15.59 (12.03)	-.82	.413
**History of Previous Injury (%) (1Year)**	37.2	33.3	.66	.579
**Dynamic Leg Strength (cm)**	30.44 (16.17)	27.01 (13.66)	1.15	.254
**Balance on Dominant Leg (s)**	2.55 (1.43)	3.22 (2.20)	-1.80	.075
**Core Stability (s)**	38.23 (17.25)	40.55 (20.93)	-.60	.547

INT – Intervention. CON – Control
Data are means (±SD).

secondary outcomes as described below. The primary outcomes were any time-loss injury to the players, injuries by type of exposure (match/training) and all injuries to the LEs. The secondary outcomes were injuries by body location, injuries by onset (acute or overuse), injuries by aetiology, mechanism and severity.

Statistical analyses

Statistical analyses for all data were performed using the Statistical Package for Social Sciences (SPSS) Version 15.0 (SPSS Inc., Chicago, Illinois, USA). Baseline characteristics, measured as continuous variables, were expressed as mean and standard deviation (SD) and categorical variables such as injury history were expressed as percentages. Baseline differences between the 2 groups were evaluated using independent t-test and chi-square test for continuous and categorical variables respectively.

Injury rates (number of injuries/1000 player hours) and 95% CI were estimated vis-a-vis the primary and secondary outcome measures for this study. The overall incidence of injuries and those of other primary and secondary outcome measures were analysed and compared between the 2 groups (as injury rate ratios – RRs) using Poisson regression analysis. Comparison of the rates of injury in the 2 groups was done according to the intention to treat principles. Statistical significance was set at $p < 0.05$.

Ethical considerations

Ethical approval for the study was obtained from the Health Research and Ethics Committee of the Lagos University Teaching Hospital, Idi-Araba, Lagos (ADM/DCST/HREC/487) prior to its commencement. Approval to carry out the study was also obtained from the LJL for official access to the various football teams. Informed consent was sought and obtained from players on team basis (each team was represented by the coach) after the purpose and procedure of study had been carefully explained to them at a pre-season screening programme prior to the commencement of the 2012/2013 league season.

Results

Descriptive characteristics of players

The baseline characteristics of the 2 groups are presented in Table 1. Players in both groups were generally well matched at baseline as no significant differences were noted in all baseline variables ($p > 0.05$).

Exposure time of players and injuries in both groups

Players' exposures to football in both groups are presented in Table 2. Overall, those in the INT group played 51,017 hours of football (involving a total of 205 matches and 431 training sessions) while those in the CON group played 61, 045 hours of football (involving a total of 218 matches and 458 training sessions). During the 6-month league season, 31 (7.5%) players were lost to follow-up, mainly because some ended their football career or they changed teams to other local or national clubs (Figure 1). However, based on the intention-to-treat standard, all available data for the players during their participation period were included in the final analysis. Drop-out rate was similar in both groups (INT - 17 (8.0%); CON - 14 (6.9%)).

Overall, 104 (25%) of the 416 players included in the study sustained a total of 130 injuries; 36 in the INT group and 94 in the CON group throughout the league season.

Compliance with the FIFA 11+ intervention programme

The 10 teams (212 players) in the INT group performed the injury prevention programme in 60% of all training sessions; corresponding to 30 ± 12 (range, 5 – 22) sessions per team. Furthermore, this is equivalent to performing the FIFA 11+ programme an average of 1.6 times per week. Regarding players' compliance, 74% of all the players in the INT group; corresponding to an average of 14 players per team participated in the programme. None of the teams in the CON regularly performed a structured preventive programme comparable to the INT programme.

Table 2. Details of players' exposure to football.

Exposure Variables	INT Group	CON Group	P-value
**Match Exposure (hours)**	3,383 (338.3 ± 42.1)	3,597 (359.7 ± 92.3)	.512
**Training Exposure (hours)**	47,634 (4,763.4 ± 1,502.3)	57,448 (5,744.8 ± 2,932.3)	.359
**Overall Exposure Time (hours)**	51,017 (5,101.7 ± 1473.7)	61,045 (6,104.6± 2,976.5)	.352

INT – Intervention; CON – Control. Data presented as sum (mean ± standard deviation) and compared using t-test analysis

Table 3. Effects of FIFA 11+ intervention on injury risk in players based on primary outcomes.

	INT Group		CON Group				
	n	Incidence (x1000 hrs)	n	Incidence (x1000 hrs)	z-test	Rate Ratio (95% CI)	P-value
**All injuries**	36	0.8	94	1.5	- 2.72	.59 (.40 – .86)	.006 *
**Match injuries**	23	7.5	73	20.3	- 4.50	.35 (.23 – .55)	<.001 *
**Training injuries**	12	0.3	22	0.4	- 0.20	.93 (.47 – 1.86)	.842
**All lower extremity injuries**	26	0.6	76	1.2	- 2.87	.52 (.34 – .82)	.004 *

* Significant at p < 0.05. CI – Confidence Interval; INT – Intervention; CON – Control.

Effects of FIFA 11+ intervention programme

The incidence of injuries and comparison of injury rates for the primary outcome measures in both groups are presented in Tables 3. Significantly lower injury rates were found in the INT group based on all the primary outcome measures ($p < 0.05$) except training injuries ($p = 0.842$). The prevention programme significantly reduced the overall rate of injury in the INT group by 41% and lower extremity injury by 48% (Table 3).

Table 4 shows the effects of the intervention programme based on the secondary outcomes. The rate of injury reduction based on secondary outcomes (injury by body location, mechanism and severity) mostly did not reach the level of significance however, there were significant differences in the relative risk of injury in group the INT group compared to the CON group ($P < 0.05$) for overuse injuries (RR = 0.26; 95% CI: 0.07 – 0.98), acute injuries (RR = 0.65; 95% CI: 0.44 – 0.97) and mild injuries (RR = 0.42; 95% CI: 0.19 – 0.95). Furthermore, there was a strong trend for a reduction in thigh ($p = 0.052$), ankle ($p = 0.08$) and non-contact injuries ($p = 0.056$).

Discussion

The principal finding in this cluster randomised controlled trial is that the FIFA 11+ neuromuscular warm-up programme significantly reduced the overall rate of injuries in male youth football by 41% and LE injuries by 48%. To our knowledge, only 2 RCTs (Soligard et al., 2008; Steffen et al., 2013) have so far evaluated the efficacy of the FIFA 11+ on football players and these studies were conducted on female youth football players. Soligard et al. (2008) reported overall significant reduction in injury rate in 11+ intervention group by 32% while Steffen et al. (2013) reported a significant reduction of 72% and 68% in all injuries and LE injuries respectively.

An overall injury reduction rate of 41% reported in the present study is comparable with that of Soligard et al. (2008) on female youth footballers in which the FIFA 11+ was reported to reduce injury risk by 32%. It is conceivable that exercise programmes have greater physical effects in younger players, since they have not yet established their basic movement patterns (Soligard et al., 2008). This may explain why an effect was found in both male and female youth football players studied so far. Although the compliance rate reported among teams (60%) in the INT group of the present study is lower than that reported by Soligard et al. (2008) for female youth football (77%), the overall injury risk reduction is higher. In contrast, player compliance (74%) in this study is higher than player compliance in that of the female youth footballers (59.4%) (Soligard et al., 2008). This might have compensated for lower team compliance and may explain why the FIFA 11+ was still effective in reducing injuries in main outcomes. Therefore, these findings suggests that male youth football teams may not need as much compliance as would be required of female players during training sessions to have the preventive effect of the FIFA 11+.

The protective effect of FIFA 11+ in reducing specific LE injuries such as thigh and ankle injuries is arguably clinically relevant, despite a lack of statistical

Table 4. Effects of FIFA 11+ intervention on injury risk in players based on secondary outcomes.

	INT Group		CON Group				
	n	Incidence (x1000 hrs)	n	Incidence (x1000 hrs)	z-test	Rate Ratio (95% CI)	P-value
**Injury by Body Location**							
Upper Extremity Injuries	10	.2	18	.3	- .38	.86 (.41 – 1.84)	.706
Thigh Injuries	1	.0	14	.2	- 1.95	.19 (.04 – 1.01)	.052
Knee Injuries	12	.3	21	.3	- 1.92	.93 (.47 – 1.88)	.848
Ankle Injuries	10	.2	30	.5	- 1.75	.53 (.27 – 1.08)	.080
Lower Leg Injuries	2	.0	3	.0	-	-	-
**Injury by Aetiology**							
Overuse Injuries	2	.0	14	.2	- 1.96	.26 (.07 – 0.98)	.047*
Acute Injuries	34	.9	80	1.3	- 2.09	.65 (.44 – 0.97)	.037*
**Injury by Mechanism**							
Contact Injuries	27	.6	66	1.1	- .98	.65 (.27 – 1.54)	.328
Non-contact Injuries	7	.2	16	.3	- 1.91	.65 (.42 – 1.01)	.056
**Injury by Severity**							
Minimal Injuries	7	.2	23	.4	- 1.50	.52 (.23 – 1.21)	.133
Mild Injuries	7	.2	27	.4	- 2.08	.42 (.19 – 0.95)	.037 *
Moderate Injuries	14	.3	33	.5	- 1.40	.64 (.35 – 1.19)	.162
Severe Injuries	7	.2	9	.2	.170	1.09 (.42 – 2.83)	.865

* Significant at p < 0.05. CI – Confidence Interval, INT – Intervention, CON – Control

significance reported in the secondary outcomes of this study ($p = 0.052$ and $p = 0.080$ respectively). Furthermore, a trend towards a reduction in non-contact injuries was also observed, but not to a level of statistical significance ($p = 0.056$). However, overuse and acute injuries were significantly reduced in the INT group compared to the CON group ($p < 0.05$).

Strengths and limitations of study

To our knowledge, this is the first RCT to investigate the effects of the FIFA 11+ on male youth football players and it is also the first injury prevention trial conducted on any population of football players in Africa. An important strength of this trial is the comprehensive baseline data that included physical characteristics, injury history of players and selected physical performance parameters. This was necessary to completely rule out any potential risk factor for injuries among players in both groups prior to the commencement of the trial. Comparative analysis of baseline data revealed that the groups were well matched.

Another strength of this trial is cluster randomisation of teams to avoid contamination between the INT and CON groups. Also, to improve the reliability and validity of the results of this study, an intention-to-treat analysis as advocated for RCTs in the 2010 consort statement was applied (Mohar et al., 2010). This was imperative to fully preserve the benefit of randomisation without bias. Hence, teams with both high and low compliance with the programme were included in the final analysis. Furthermore, we adapted the consensus statement on injury definitions and data collection in football (Fuller et al., 2006) so that our study can be compared with other studies relating to football injury prevention.

This study however has some limitations. Firstly, the researchers and assistants could not be blinded to the INT programme because they had to monitor compliance and evaluate injured players in both groups. Secondly, all injuries regardless of severity were diagnosed based on subjective assessment and physical examination of players due to limited funds and resources for the study. In order to minimise the effect of the aforesaid, injuries were not described with reference to type, but by location, aetiology, mechanism and severity. Thirdly, administration of 11+ exercises was done by coaches with occasional supervision by study physiotherapists. The extent to which the INT teams used the programme with fidelity and progressed through the programme is uncertain. However, this approach was preferred so as to resemble a real world situation where no external professional resources would be unavailable to youth football teams as mostly applicable in Africa. Fourthly, information on players' compliance with the 11+ programme was got from the coaches; hence it is possible that compliance with INT programme might have been over- or under-reported for the teams and players.

Finally, the CON group did not perform a standardized programme of warm-up. Each team performed its usual warm-up programme; mostly involving a combination of stretches, jogging and strength exercises a in a non-standardized fashion and we had no control over whether any of the teams in the CON group performed exercises similar to those of the INT group during the study period. However, any possible bias introduced by such contamination of the CON group would only lead to underestimation of the preventive effect found in our study.

Implications

Although the pattern of injuries in African players have been noted to be mostly consistent with reports from other parts of the world, a peculiar record of higher injury rates has been documented (Akodu et al., 2013; Owoeye et al., 2012; Zerguini et al., 2010). This may be connected with the more physical pattern of play and a general problem of inadequate funding by government and stakeholders in most parts of the continent. This implies poor pitches and playing surfaces, lack of training facilities and inadequate personnel among others. Factors such as poor medical support and poor knowledge and practice of injury prevention strategies especially at the youth football level have been highlighted and may also be contributory (Owoeye et al., 2013). Successful preventive measures in football are therefore crucial in Africa. This could have a substantial effect on injury burden and also dramatically diminish the costs associated with treatment. The findings from our study supports an advocacy for the FIFA 11+ injury prevention programme as a viable low cost injury prevention option for youth football players in the region.

Conclusion

This study has established that the FIFA 11+ programme is effective in reducing the incidence of injury in male youth football players. A country-wide campaign and implementation of the FIFA 11+ injury prevention programme among male youth football players may be pursued by youth football administrators and federations across Nigeria and regions of Africa respectively in order to help minimise the risk of injury among players. The world governing body for football (FIFA) should consider supporting federations and local administrators in achieving this.

Acknowledgements

We are grateful to F-MARC for providing the FIFA 11+ materials used for this research project. We also thank the study physiotherapists: Yewande Apatira, BPT and Wole Fasuba, BPT, the Lagos Junior League administrators; particularly Mr. Tunde Disu (Technical Director) and Mr. Taofik Aromire (Executive Secretary), coaches and players who participated in this project.

References

Aaltonen, S., Karjalainen, H., Heinonen, A., Parkkari, J. and Kujala, U. (2007) Prevention of sports injuries: systematic review of randomized controlled trials. *Achieves of Internal Medicine* **167,** 1585-1592.

Akodu, A.K., Owoeye, O.B.A., Ajenifuja M., Akinbo, S.R.A., Olatona, F. and Ogunkunle, O. (2012) Incidence and characteristics of injuries during the 2011 West Africa Football Union (WAFU) Nations' Cup. *African Journal of Medicine and Medical Sciences* **41,** 423-428.

Alentorn-Geli, E., Myer, G.D., Silvers, H.J., Samitier, G., Romero, D., Lazaro-Haro, C. and Cugat, R. (2009) Prevention of non-contact anterior cruciate ligament injuries in soccer players. Part 2: a re-

view of prevention programmes aimed to modify risk factors and to reduce injury rates. *Knee Surgery, Sports Traumatology, Arthroscopy* **17,** 859-879.

Arnason, A., Andersen, T.E., Holme, I., Engebretsen, L. and Bahr, R. (2008) Prevention of hamstring strains in elite soccer: an intervention study. *Scandinavian Journal of Medicine and Science in Sports* **18,** 40-48.

Askling, C., Karlsson, J. and Thorstensson, A. (2003) Hamstring injury occurrence in elite soccer players after preseason strength training with eccentric overload. *Scandinavian Journal of Medicine and Science in Sports* **13,** 244-250.

Constantinou, D. (2010) Football injuries – surveillance, incidence and prevention. *Continuing Medical Education* **28(5),** 220-225.

Emery, C.A. and Meeuwisse, W.H. (2010) The effectiveness of a neuromuscular prevention strategy to reduce injuries in youth soccer: a cluster-randomised controlled trial. *British Journal of Sports Medicine* **44,** 555-562.

FIFA. (2006) FIFA Big Count. FIFA communications division, information services. Available from URL: http://www.fifa.com/mm/document/fifafacts/ bcoffsurv/bigcount.statspackage_7024.pdf

F-MARC. (2013) Fifa 11+ - a complete warm-up programme. Available from URL: http://f-marc.com.

Fuller, C.W., Ekstrand, J., Junge, A., Anderson, T.E., Bahr, R., Dvorak, J., Hugglund, M., McCrory, P. and Meeuwisse, W.H. (2006) Consensus statement on injury definitions and data collection procedures in studies of football (soccer) injuries. *British Journal of Sports Medicine* **40,** 193-201.

Grooms, D.R., Palmer, T., Onate, J.A., Myer, G.D., Grindstaff, T. (2013) Soccer-Specific Warm-Up and Lower Extremity Injury Rates in Collegiate Male Soccer Players. *Journal of Athletic Training* **48(6):** 782–789.

Hawkins, R.D. and Fuller, C.W. (1999) A prospective epidemiological study of injuries in four English professional football clubs. *British Journal of Sports Medicine* **33,** 196-203.

Heidt, R.S. Jr, Sweeterman, L.M., Carlonas, R.L., Traub, J.A. and Tekulve, F.X. (2000) Avoidance of soccer injuries with preseason conditioning. *American Journal of Sports Medicine* 28, 659-962.

Herman, K., Barton C, Malliaras, P. and Morrissey, D. (2012) The effectiveness of neuromuscular warm-up strategies, that require no additional equipment, for preventing lower limb injuries during sports participation: a systematic review. *BMC Medicine* **10,** 75

Kang, M., Ragan, B.G. and Park, J. (2008) Issues in Outcomes Research: An Overview of Randomization Techniques for Clinical Trials. *Journal of Athletic Training* **43(2),** 215-221.

Kilding, A.E., Tunstall, H. and Kuzmic, D. (2008) Suitability of FIFA's "The 11" training programme for young football players - impact on physical performance. *Journal of Sports Science and Medicine* **7,** 320-326.

Kirkendal, D.T., Junge, A. and Dvorak J. (2010) Prevention of football injuries. *Asian Journal of Sports Med*icine **1(2),** 81-92.

Moher, D., Hopewell, S., Schulz, K.F.. Montori, V., Gøtzsche, P.C., Devereaux, P.J, Elbourne, D, Egger, M, Altman, D.G. (2010) CONSORT 2010 Explanation and Elaboration: updated guidelines for reporting parallel group randomised trials. *British Medical Journal* **23,** 340.

Owoeye, O.B.A., Nwachukwu, A.L. and Akinbo, S.R.A. (2012) Injuries in Nigeria National female footballers at the 2008 Beijing Olympic Games, China: A prospective case study. *African Journal of Physiotherapy and Rehabilitation Sciences* **4(1&2),** 57-61.

Owoeye, O.B.A., Akinbo, S.R.A., Olawale, O.A., Tella, B.A. and Ibeabuchi, N.M. (2013) Injury prevention in football: Knowledge and behaviour of players and availability of medical care in a Nigerian youth football league. *South African Journal of Sports Medicine* **25(3),** 77-80.

Petersen, J., Thorborg, K., Nielsen, M.B., Budtz-Jorgensen, E. and Holmich, P. (2011) Preventive effect of eccentric training on acute hamstring injuries in men's soccer: a cluster-randomized controlled trial. *American Journal of Sports Medicine* **39,** 2296-303.

Soligard, T., Myklebust, G., Steffen, K., Holme, I., Silvers, H., Bizzini, M., Junge, A., Dvorak, J., Bahr, R. and Andersen, T.E. (2008) Comprehensive warm-up programme to prevent injuries in young female footballers: cluster randomised controlled trial. *British Medical Journal* **337,** 2469.

Steffen, K., Emery, C.A., Romiti, M., Kang, J., Bizzini, M., Dvorak, J., Finch, C.F. and Meeuwisse, W.H. (2013) High adherence to a neuromuscular injury prevention programme (FIFA 11+) improves functional balance and reduces injury risk in Canadian youth female football players: a cluster randomised trial. *British Journal of Sports Medicine* **47(12),** 794-802.

Zerguini, Y., Bungu-kakula, J.P., Junge, A. and Dvorak, J. (2010) Examination of injuries in an African national football league. The 2006 – 2007 EPFKIN championship in Kinshasha (Congo DR). *Journal of Sports Traumatology* **27,** 171-176.

Key points

- The FIFA 11+ has only been tested in randomised controlled trials conducted on female youth football players; this study reports its efficacy in male youth football for the first time
- The FIFA 11+ programme significantly reduced the overall rate of injuries and lower extremity injuries in male youth football players
- Youth football administrators in Africa and other parts of the world should pursue the implementation of the FIFA 11+ in order to minimize the incidence of injuries among players

AUTHORS BIOGRAPHY

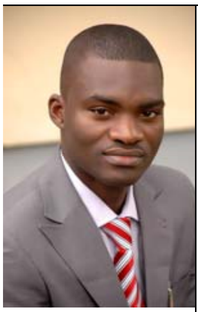

Oluwatoyosi B. A. OWOEYE
Employment
Lecturer at the Orthopaedic and Sports Physiotherapy Units, Department of Physiotherapy, Faculty of Clinical Sciences, College of Medicine, University of Lagos
Degree
MSc, PhD Scholar
Research interests
Injury prevention in sports, football medicine, youth sports, sports and exercise medicine.
E-mail: obowoeye@unilag.edu.ng

Sunday R. A. AKINBO
Employement
Professor at the Orthopaedic and Sports Physiotherapy Units, Department of Physiotherapy, Faculty of Clinical Sciences, College of Medicine, University of Lagos
Degree
PhD
Research interests
Orthopaedics and rheumatology, sports medicine.
E-mail: sraakinbo@cmul.edu.ng

Bidemi A. TELLA
Employment
Senior Lecturer at the Orthopaedic and Sports Physiotherapy Units, Department of Physiotherapy, Faculty of Clinical Sciences, College of Medicine, University of Lagos
Degree
PhD
Research interests
Orthopaedics and rheumatology, women health
E-mail: adedemi@yahoo.com

Olajide A. OLAWALE
Employment
Associate professor at the Neuro-Physiotherapy Unit, Department of Physiotherapy, Faculty of Clinical Sciences,

College of Medicine, University of Lagos
Degree
PhD
Research interests
Neuro-rehabilitation, sports medicine
E-mail: olajideolawale@yahoo.com

✉ **Oluwatoyosi B.A. Owoeye**
Lecturer and PhD Scholar, Department of Physiotherapy, Faculty of Clinical Sciences, College of Medicine, University of Lagos, PMB 12003, Lagos, Nigeria
