# Supplementary material for: Comparative Effectiveness of Multi-Component, Exercise-Based Interventions for Preventing Soccer-Related Musculoskeletal Injuries: A Systematic Review and Meta-Analysis
Source: Healthcare (Basel). 2025 Mar 29;13(7):765. doi: 10.3390/healthcare13070765 (PMC11988859; doi:10.3390/healthcare13070765)
Supplement: Supplementary file 1 [file healthcare-13-00765-s001.zip › Additional material Included literature/Silvers-Granelli2017.pdf]

Clin Orthop Relat Res
DOI 10.1007/s11999-017-5342-5

Clinical Orthopaedics and Related Research®
A Publication of The Association of Bone and Joint Surgeons®

SYMPOSIUM: IMPROVING CARE FOR PATIENTS WITH ACL INJURIES: A TEAM APPROACH

Does the FIFA 11+ Injury Prevention Program Reduce the Incidence of ACL Injury in Male Soccer Players?

Holly J. Silvers-Granelli MPT, Mario Bizzini PhD, MSC, PT, Amelia Arundale DPT, Bert R. Mandelbaum MD, Lynn Snyder-Mackler PT, ScD

© The Association of Bone and Joint Surgeons® 2017

Abstract

Background The FIFA 11+ injury prevention program has been shown to decrease the risk of soccer injuries in men and women. The program has also been shown to decrease time loss resulting from injury. However, previous studies have not specifically investigated how the program might impact the rate of anterior cruciate ligament (ACL) injury in male soccer players.

Questions/purposes The purpose of this study was to examine if the FIFA 11+ injury prevention program can (1) reduce the overall number of ACL injuries in men who play competitive college soccer and whether any potential reduction in rate of ACL injuries differed based on (2) game versus practice setting; (3) player position; (4) level of play (Division I or II); or (5) field type.

Methods This study was a prospective cluster randomized controlled trial, which was conducted in 61 Division I and Division II National Collegiate Athletic Association men's soccer teams over the course of one competitive soccer season. The FIFA 11+ is a 15- to 20-minute on-the-field dynamic

One of the authors (HJS-G) received a PhD Research Grant from Simbex, Inc (Lebanon, NH, USA; less than USD 10,000) and is a research consultant to Major League Soccer's M-MARC program (New York, NY, USA; less than USD 10,000) unrelated to this work. One of the authors (AA) received a grant from the National Institutes of Arthritis and Musculoskeletal and Skin Diseases (Bethesda, MD, USA; R01-AR048212; less than USD 10,000) and the Foundation for Physical Therapy–Promotion of Doctoral Studies I Scholarship (Alexandria, VA, USA; less than USD 10,000). One of the authors (BRM) received funding from FIFA (Zurich, Switzerland) to support the completion of this study (see subsequently) and is a consultant to Arthrex (City of Industry, CA, USA; USD 10,000 to USD 100,000), RTI (Alachua, FL, USA; USD 10,000 to USD 100,000), Exactech Inc (Gainesville, FL, USA; USD 10,000 to USD 100,000), and DePuy Mitek (Raynham, MA, USA; USD 10,000 to USD 100,000). The institution (Santa Monica Orthopaedic Group) of one or more of the authors (HJS-G, BRM) has received funding from FIFA's F-MARC program (Zurich, Switzerland; 2012) for the initial publication (USD 10,000 to USD 100,000).
All ICMJE Conflict of Interest Forms for authors and *Clinical Orthopaedics and Related Research®* editors and board members are on file with the publication and can be viewed on request.
Clinical Orthopaedics and Related Research® neither advocates nor endorses the use of any treatment, drug, or device. Readers are encouraged to always seek additional information, including FDA-approval status, of any drug or device prior to clinical use.
Each author certifies that his or her institution approved the human protocol for this investigation, that all investigations were conducted in conformity with ethical principles of research, and that informed consent for participation in the study was obtained.
This work was performed at the University of Delaware, Newark, DE, USA.

H. J. Silvers-Granelli, A. Arundale
Biomechanics and Movement Science Program, University of Delaware, Newark, DE, USA

M. Bizzini
Fédération Internationale de Football Association (FIFA), Medical Assessment and Research Centre (F-MARC), Schulthess Clinic, Zurich, Switzerland

B. R. Mandelbaum
Santa Monica Orthopaedic Group, Santa Monica, CA, USA

L. Snyder-Mackler
Department of Physical Therapy and Biomechanics and Movement Science Program, University of Delaware, Newark, DE, USA

H. J. Silvers-Granelli (✉)
Velocity Physical Therapy, 11611 San Vicente Boulevard, GF-1, Los Angeles, CA 90049, USA
e-mail: hollysilverspt@gmail.com

Published online: 07 April 2017

warm-up program used before training and games and was utilized as the intervention throughout the entire competitive season. Sixty-five teams were randomized: 34 to the control group (850 players) and 31 to the intervention group (675 players). Four intervention teams did not complete the study and did not submit their data, noting insufficient time to complete the program, reducing the number for per-protocol analysis to 61. Compliance to the FIFA 11+ program, athletic exposures, specific injuries, ACL injuries, and time loss resulting from injury were collected and recorded using a secure Internet-based system. At the end of the season, the data in the injury surveillance system were crosshatched with each individual institution's internal database. At that time, the certified athletic trainer signed off on the injury collection data to confirm their accuracy and completeness.

Results A lower proportion of athletes in the intervention group experienced knee injuries (25% [34 of 136]) compared with the control group (75% [102 of 136]; relative risk [RR], 0.42; 95% confidence interval [CI], 0.29-0.61; $p < 0.001$). When the data were stratified for ACL injury, fewer ACL injuries were reported in the intervention group (16% [three of 19]) compared with the control group (84% [16 of 19]), accounting for a 4.25-fold reduction in the likelihood of incurring ACL injury (RR, 0.236; 95% CI, 0.193–0.93; number needed to treat = 70; $p < 0.001$). With the numbers available, there was no difference between the ACL injury rate within the FIFA 11+ group and the control group with respect to game and practice sessions (games—intervention: 1.055% [three of 15] versus control: 1.80% [12 of 15]; RR, 0.31; 95% CI, 0.09–1.11; $p = 0.073$ and practices—intervention: 0% [zero of four] versus control: 0.60% [four of four]; RR, 0.14; 95% CI, 0.01–2.59; $p = 0.186$). With the data that were available, there were no differences in incidence rate (IR) or injury by player position for forwards (IR control = 0.339 versus IR intervention = 0), midfielders (IR control = 0.54 versus IR intervention = 0.227), defenders (IR control = 0.339 versus IR intervention = 0.085), and goalkeepers (IR control = 0.0 versus IR intervention = 0.0) ($p = 0.327$). There were no differences in the number of ACL injuries for the Division I intervention group (0.70% [two of nine]) compared with the control group (1.05% [seven of nine]; RR, 0.30; CI, 0.06–1.45; $p = 0.136$). However, there were fewer ACL injuries incurred in the Division II intervention group (0.35% [one of 10]) compared with the control group (1.35% [nine of 10]; RR, 0.12; CI, 0.02–0.93; $p = 0.042$). There was no difference between the number of ACL injuries in the control group versus in the intervention group that occurred on grass versus turf (Wald chi square [1] = 0.473, b = 0.147, SE = 0.21, $p = 0.492$). However, there were more ACL injuries that occurred on artificial turf identified in the control group (1.35% [nine of 10]) versus the intervention group (0.35% [one of 10]; RR, 0.14; 95% CI, 0.02–1.10; $p = 0.049$).

Conclusions This program, if implemented correctly, has the potential to decrease the rate of ACL injury in competitive soccer players. In addition, this may also enhance the development and dissemination of injury prevention protocols and may mitigate risk to athletes who utilize the program consistently. Further studies are necessary to analyze the cost-effectiveness of the program implementation and to analyze the efficacy of the FIFA 11+ in the female collegiate soccer cohort.

Level of Evidence Level I, therapeutic study.

Introduction

Soccer-related injuries are a relatively common occurrence across sex, age, and level of competition. The high prevalence of soccer-related injury has been well documented [5, 6, 13, 19, 20, 22, 23, 26, 31, 35]. Injuries incurred during soccer most commonly involve the lower extremity and most commonly occur in a game situation [9, 10, 18, 21]. The National Collegiate Athletic Association (NCAA) has reported that the game-related injury rate in men's and women's soccer games ranked third and fourth for all NCAA sports, respectively [1, 2]. Anterior cruciate ligament (ACL) injuries continue to consistently negatively impact recreational, competitive, and professional athletes globally. There are approximately 200,000 ACL injuries that occur in the United States annually making it the most commonly injured ligament in the knee [3, 28]. The NCAA's Injury Surveillance System (ISS and DATALYS) reported that the overall ACL injury rates were 1.45 per 10,000 athletic exposures for female athletes and 0.60 per 10,000 athletic exposures for male athletes [48]. Gilchrist et al. [25] noted that 31% of Division I soccer athletes polled had a history of knee injury and 14% had a history of ACL injury. The documented increase in incidence and the increased risk associated with prior knee injury initiate an obvious concern for the health and integrity of the articular cartilage of the knee in this young athletic cohort longitudinally [12, 17, 36, 38, 52].

For the last three decades, there has been a variety of effective ACL injury prevention programs developed, namely for high-risk sports [11, 14, 25, 32, 39–41, 46, 47]. Many of these programs have focused specifically on female athletes [25, 33, 39, 40, 47, 50] and have included a variety of strengthening, plyometric, and agility-based drills that addressed the major deficits most commonly associated with ACL injury [24, 27, 29]. Several programs have been designed as dynamic warm-up programs to increase program utilization and compliance and to capitalize on the biomechanical advantages associated with improved joint position sense [39, 45, 47]. Despite the development and the evolution of the aforementioned

programs, there is a continued and implicit need to address soccer-related injury in totality. The FIFA 11+ injury prevention program was designed to address all soccer-related injuries not only specific to the knee or to the ACL [47]. It is a dynamic on-the-field warm-up that is time-efficient and requires no additional equipment. The efficacy of the program has been documented and decreases in overall injury rate have been shown in both male and female soccer players [30, 44, 46, 47]. However, prior studies did not specifically analyze the ability of the FIFA 11+ prevention program to reduce the number of ACL injuries in male soccer players.

The purpose of this study was to examine if the FIFA 11+ injury prevention program can (1) reduce the overall number of ACL injuries in men who play competitive college soccer and whether any potential reduction in rate of ACL injuries differed based on (2) game versus practice setting; (3) player position; (4) level of play (Division I or II); or (5) field type.

Patients and Methods

As previously reported in an earlier publication, a prospective cluster randomized controlled trial was conducted in Division I and Division II NCAA men's soccer teams in the Fall 2012 season [46]. Every NCAA member institution with a men's Division I or Division II soccer program (N = 396) was contacted through a formal letter, email, and a direct phone call. The correspondence included a hyperlink for a video that featured former and current prominent US soccer players and a coach who discussed the nature and importance of prevention in the sport of soccer (http://vimeo.com/25708967 and http://vimeo.com/25708960). Of the 396 eligible teams, 299 met the inclusion criteria. Sixty-five institutions consented to participate with the male participants from each institution ranging in age from 18 to 25 years. The additional institutions opted out of the study noting time restrictions, no current issues with injuries in their team, not enough coaching staff to implement the program, not wanting to implement the program in the competitive fall season, or lack of interest. Human ethics internal review board approval was obtained through the Quorum institutional review board (Seattle, WA, USA).

The inclusion criteria stipulated that each subject was a male college soccer player between the ages of 18 and 25 years in good academic standing and was medically cleared to participate in the 2012 season. The teams confirmed that they had not participated in an injury prevention program in the past 4 academic years to avoid subject contamination. Before simple computer-generated team randomization, individual player informed consent was obtained and a documentation of coaching understanding was signed by each institution to ensure robust comprehension of the expectations of study participation.

On computer-generated randomization of the enrolled institutions, the intervention group received an instructional FIFA 11+ DVD, prevention manual, and explanatory placards describing the FIFA 11+ intervention (www.f-marc.com/11plus). The FIFA 11+ is a 15- to 20-minute on-the-field dynamic warm-up program used before games and training performed two to three times a week throughout the entire season. It includes strength, agility, proprioceptive, and plyometric exercises and was designed to reduce injuries most commonly identified in soccer players.

A secure Internet-based injury surveillance system was utilized (HealtheAthlete; Cerner Corporation, Overland Park, KS, USA) by every enrolled institution (control group and intervention group). Every athletic exposure, injury incurred (including ACL injury), mechanism of injury, and date of return to play were entered weekly by the team's certified athletic trainer. The environmental conditions of the ACL injury were also considered with respect to field type: grass versus artificial turf. Sixty-five institutions were randomized using a simple computer-generated randomization and 61 completed the study during the Fall 2012 season (August to December): 34 control institutions (N = 850 athletes; 17 Division I teams [425 players] and 17 Division II teams [425 players]) and 27 intervention institutions (N = 675 athletes; 16 Division I teams [400 players] and 11 Division II teams [275 players]) (Fig. 1). Demographic information including age, position played, and leg dominance was also collected. During the course of the season, the research staff monitored the data entry for each institution. In the event that no logon to the injury surveillance system was detected and no data were uploaded into the system for 14 days, a computer-generated email was dispersed and a research staff member followed up immediately. On the completion of the season, the data entry was confirmed by each certified athletic trainer and the accuracy and completeness with their individual institution's internal data collection system were established. As a result of the loss of four intervention teams to followup, a per-protocol analysis of the data was completed.

Statistical Analysis

All statistical analyses were conducted utilizing IBM SPSS Statistics Editor for MAC Version 24 (IBM Corporation, Armonk, NY, USA). Descriptive and inferential tests were used to compare the control group and intervention group, including frequency counts, t-tests, chi-square tests,

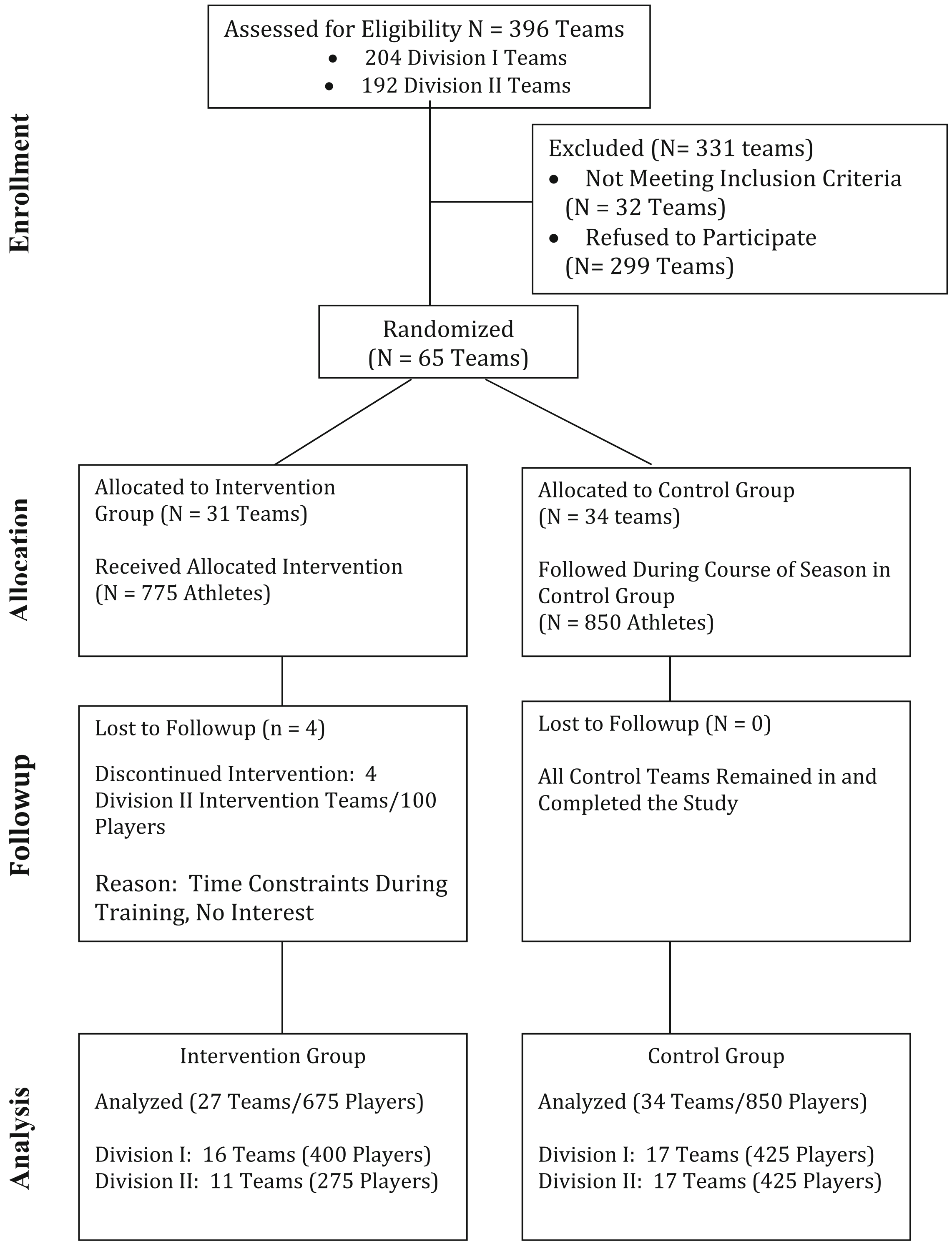

Fig. 1 Description of the NCAA team randomization and study flow is presented.

factorial analysis of variance, and logistic regression tests (Biostatistics Core Facility University of Delaware, Newark, DE, USA). Injury rates were calculated based on athletic exposures and are expressed as a rate per 1000 athletic exposures.

Results

There were 1305 overall team exposures to the FIFA 11+ in the intervention group (405 games and 900 training sessions) over the course of the season with an average of

2.19 FIFA 11+ utilizations over the course of the season per week. The control group consisted of 850 athletes (34 teams [56%]) who had 44,212 athletic exposures (games: 13,624 and practices: 30,588). The intervention group consisted of 675 athletes (27 teams; 44%) who had 35,226 athletic exposures (games: 10,935 and practices: 24,291) (Fig. 1) [46]. There was no difference between the ages of the athletes at the time of ACL injury (control group: 20.68 ± 1.46 years versus intervention group: 20.40 ± 1.66 years, range, 20.24–21.81, $p = 0.914$) (Table 1). The risk of ACL injury was lower in the teams that used FIFA 11+ than in those that did not (1.1% [three of 19] versus 2.4% [16 of 19]; relative risk [RR], 0.24; 95% confidence interval [CI], 0.07–0.81; $p = 0.021$). When identifying the mechanism of ACL injury, there was a higher injury rate in the control group compared with the intervention group for both contact and noncontact mechanisms. For contact ACL injuries, there were fewer injuries in the athletes who used the FIFA 11+ compared with those who did not (0.35% [one of seven] versus 0.90% [six of seven]; RR, 0.21; 95% CI, 0.03–1.74; $p = 0.148$). For noncontact mechanisms, there were fewer ACL injuries in the athletes who utilized the FIFA 11+ compared with those who did not (0.70% [two of 12] versus 1.5% [10 of 12]; RR, 0.25; 95% CI, 0.06-1.15; $p = 0.049$), representing a 75% decrease in noncontact ACL injury (Table 2).

With the numbers available, there was no difference between the ACL injury rate within the FIFA 11+ and control groups with respect to game and practice sessions (games—intervention: 1.055% [three of 15] versus control: 1.80% [12 of 15]; RR, 0.31; 95% CI, 0.09–1.11; $p = 0.073$ and practices—intervention: 0% [zero of four] versus control: 0.60% [four of four]; RR, 0.14; 95% CI, 0.01–2.59; $p = 0.186$) (Table 2).

Table 1. Control versus intervention group demographic values

Team/athlete characteristics	Control<br>Teams	Intervention<br>Teams	Range	p value
Players (number)	850/34 teams<br>Division I: 425 (17 teams)<br>Division II: 425 (17 teams)	675/27 teams<br>Division I: 400 (16 teams)<br>Division II: 275 (11 teams*)	–	–
Age (years)				
Age (years; mean ± SD)	21 ± 1	20 ± 2	20–22	0.914
Athletic exposures				
Number of athletic exposures	Total: 44,212<br>Games: 13,624<br>Practices: 30,588	Total: 35,226<br>Games: 10,935<br>Practices: 24,291	–	–

* Four teams lost to followup.

With the numbers available, there were no differences associated with player position in either group; the incidence rates for midfielders, defenders, forwards, and goalkeepers in the intervention group were 0.227, 0.085, 0, and 0, whereas in the control group they were 0.54, 0.339, 0.339, and 0, respectively ($p = 0.207$) (Table 2).

We observed no differences with the numbers available between the ACL injury rates for Division I between groups (intervention: 0.70% [two of nine] versus control: 1.05% [seven of nine]; RR, 0.30; 95% CI, 0.06–1.45; $p = 0.136$). However, the risk of injury was lower in the intervention group than the control group in Division II athletes (intervention: 0.35% [one of 10] versus control: 1.35% [nine of 10]; RR, 0.12; 95% CI, 0.02–0.93; $p = 0.042$) (Table 2).

A two-way analysis of variance was conducted to compare the main effects of field type between the intervention group and control group on contact versus noncontact ACL injury. A logistic linear regression (Poisson) analysis was used to compare the number of ACL injuries between groups, intervention versus control, and for field types, grass versus turf, because number of ACL injuries is a count variable and normality was violated for both groups. There was no difference between the number of ACL injuries in the control group versus the intervention group that occurred on grass versus turf (Wald chi square [1] = 0.473, b = 0.147, SE = 0.21, $p = 0.492$). There were no differences in the number of ACL injuries that occurred on grass between the teams that used the FIFA 11+ versus those that did not (control group: 1.05% [seven of nine] versus intervention group: 0.7% [two of nine]; RR, 0.36; 95% CI, 0.08–1.73; $p = 0.201$). However, there were more ACL injuries that occurred on artificial turf identified in the control group (1.35% [nine of 10]) versus the intervention group (0.35% [one of 10]; RR, 0.14; 95% CI, 0.02–1.10; $p = 0.049$; Table 3).

Discussion

The FIFA 11+ was designed as an injury prevention program to address the most common soccer-related injuries. Unlike other injury prevention programs, the FIFA 11+ was not solely designed to decrease ACL injury [32, 39–41]. To our knowledge, the degree to which the program may effectively reduce the rate of ACL injury has not been examined [30, 37, 44, 47, 50]. This current study demonstrated that the FIFA 11+ program decreased the overall incidence rate of ACL injury by 77% in competitive collegiate male soccer players. There was no difference in ACL injury rates based on grass, games versus practices, in Division I athletes, or between player positions. However, there were fewer ACL injuries incurred in the Division II

Table 2. Control versus intervention group comparison chart, injury frequency, percent of total injury, injury rates, RRs with 95% confidence intervals, and p values

Injury characteristics	Control			Intervention			RR (95% CI)	p value
		Number/percent	IR		Number/percent	IR		
Total injuries	Total	665/100	15.04	Total	285/100	8.09	0.54 (0.49–0.59)	< 0.001*
	Game	392/58.9	28.77	Game	185/64.9	16.92	0.59 (0.52–0.68)	< 0.001*
	Practice	273/41.1	8.93	Practice	100/35.1	4.01	0.46 (0.38–0.57)	< 0.001*
Knee injuries	Total	102/15.3	2.307	Total	34/11.9	0.965	0.42 (0.29–0.61)	< 0.001*
Mechanism of ACL	Total	16 /2.41	0.362	Total	3/1.05	0.085	0.24 (0.07–0.81)	0.021*
	Contact	6/0.90	0.135	Contact	1/0.35	0.028	0.21 (0.03–1.74)	0.148
	Non-contact	10/1.50	0.226	Noncontact	2/0.70	0.057	0.25 (0.06–1.15)	0.049*
ACLs game versus practice	Game	12/1.80	0.881	Game	3/1.05	0.283	0.31 (0.09–1.11)	0.073
	Practice	4/0.60	0.131	Practice	0	0.0	0.14 (0.01–2.59)	0.186
ACLs incurred (number/%) by position	Defender	5/0.75	0.339	Defender	1/0.35	0.085	0.25 (0.03–2.15)	0.207
	Forward	5/0.75	0.339	Forward	0	0	0.11 (0.01–2.07)	0.142
	Midfielder	6/0.90	0.54	Midfielder	2/0.70	0.227	0.42 (0.06–2.07)	0.142
	Goalkeeper	0	0	Goalkeeper	0	0	1.26 (0.03–63.36)	0.908
ACLs by division	Division I	7/1.05	0.317	Division I	2/0.70	0.114	0.30 (0.06–1.45)	0.136
	Division II	9/1.35	0.407	Division II	1/0.35	0.057	0.12 (0.02–0.93)	0.042*

* Statistical significance with $p < 0.05$; RR = rate ratio; IR = injury rate; CI = confidence interval; ACL = anterior cruciate ligament.

Table 3. Anterior cruciate ligament injuries by field type

Environmental condition	Control			Intervention			RR (95% CI)	p value
	ACL type	Number/percent	IR		Number/percent	IR		
Grass	Total	7/1.05	0.158	Total	2/0.70	0.057	0.36 (0.08–1.73)	0.201
	Noncontact	4/0.60	0.090	Noncontact	2/0.70	0.057	0.63 (0.12–3.48)	0.535
	Contact	3/0.45	0.067	Contact	0	0.0	0.18 (0.01–3.58)	0.256
Turf	Turf Total	9/1.35	0.204	Total	1/0.35	0.035	0.14 (0.02–1.10)	0.049*
	Noncontact	6/0.90	0.135	Noncontact	0	0.0	0.10 (0.01–1.72)	0.111
	Contact	3/0.45	0.678	Contact	1/0.35	0.35	0.18 (0.01–3.48)	0.256
	Grass versus turf within CG: p = 0.719			Grass versus turf within IG: p = 0.645				

* Statistical significance with $p < 0.05$; chart describes ACL injuries within the CG and IG by field type; the main effect for field type = $F(1,18) = 1.885$, $p = 0.190$ and the main effect for group = $F(1,18) = 0.131$, $p = 0.723$; the interaction effect was not significant $F(1,18) = 2.762$, $p = 0.117$; however, there was a significant difference between the CG (N = 9 [1.35%], IR = 0.407) and the IG (N = 1 [0.35%], IR = 0.057) for all ACL injuries that occurred on artificial turf (RR = 0.14, 95% CI, 0.02-1.10, p = 0.049); ACL = anterior cruciate ligament; IR = injury rate; RR = rate ratio; CI = confidence interval; CG = control group; IG = intervention group.

teams that utilized the FIFA 11+ compared with the control group (p = 0.042). In addition, there were more ACL injuries that occurred on artificial turf identified in the control group compared with the intervention group (p = 0.049).

The study's limitations include that four intervention teams were lost to followup and, therefore, an intent-to-treat analysis was not feasible. A per-protocol analysis was completed, which might inflate the reported benefit to the intervention group. This study only involved male soccer players. The rate of ACL injury in the male collegiate cohort is typically lower than the female injury rate [1, 2, 4]. However, the initial study of the FIFA 11+ was conducted using female soccer players, hence the decision to study the male population in this specific study [47]. In addition, the study has been lacking the statistical power to sufficiently compare ACL injury rates in the various subgroups despite the fact that the study encompassed 1525 athletes participating on 61 collegiate soccer teams. The occurrence of an ACL injury is a relatively rare event, and as a result of the prospective nature of the study design, we were limited in our analysis attributable to the low

incidence rate of ACL injury during the data collection period. The analysis comparing ACL injury rates in games and practices, for Division I athletes and for grass injuries, showed no difference compared with the overall ACL injury rate and the overall injury rate reported and analyzed in an earlier publication [46]. Although steps were taken to mitigate team and player contamination to injury prevention program exposure, we were unable to fully account for program exposure that may have occurred in the high school and club soccer setting or in the event that the athlete transferred from another institution.

This study demonstrated a decreased overall risk of ACL injury and noncontact ACL injury in men in the intervention group. The study did not reflect a decrease in contact ACL injury despite the fact that there was only one contact ACL injury reported in the intervention group compared with six in the control group. This may be explained by the fact that ACL injuries, despite their deleterious nature, are a relatively rare event in the sport of soccer, which is evident when analyzing the injury rate.

There was no difference in the male ACL injury rate between groups with respect to player position. This is inconsistent with prior research that has demonstrated that defenders are at a higher risk for ACL injury than other player positions [10, 53]. A recent study highlighted the fact that, on video analysis of ACL injuries occurring in the sport of soccer, 73% of the injuries occurred while defending [10]. An additional study corroborated these findings suggesting that the most common playing situations preceding an ACL injury were defensive in nature 77% of the time: pressing followed by kicking and heading [53]. Ascertaining meaningful knowledge about the incidence of ACL injury based on the specific demands of player positioning may allow researchers to improve existing injury prevention and reduction methods [8, 10, 53].

We did not observe a difference in ACL injury rates between the FIFA 11+ and control teams in Division I soccer, but we did observe fewer ACL injuries among Division II teams that trained using FIFA 11+. Historically, game and practice injury rates have been shown to be lower in Division II and III compared with Division I [34]. This might be attributed to differences in the intensity of play and overall skill level across divisions. This may also represent an important finding on program delivery and overall program efficacy. The FIFA 11+ program was designed to be administered by coaches, parents, or athletes who may or may not have any medical expertise, clinical background, or a biomechanical knowledge base. Division II athletes traditionally are not privy to as many resources as Division I athletes and may not have direct oversight during program delivery by a certified athletic trainer or strength and conditioning coach for every game and training session. Therefore, the data suggest that this program can be effectively implemented without demanding the presence of a licensed medical professional. This has important implications from a public health perspective with respect to cost-effectiveness and the ease of program implementation [7, 49].

Although the overall risk of injury was not greater on turf than on grass, the risk of injury on turf was lower in the group that used FIFA 11+ than the group that did not. Field type has been discussed in prior work and has often been found to be associated with an increased risk of ACL injury in other NCAA sports [15, 16]. Researchers and clinicians should consider the role that field surface may play in addition to friction coefficient from the shoe-surface interface and peak torque measures between the shoe and playing surface [42, 43, 51]. Further clinical investigation is warranted to enhance the understanding of how these variables may affect the rate of ACL injury.

The results of this study demonstrated the ability of the FIFA 11+ to decrease the incidence of ACL injuries in competitive collegiate male soccer players by 77%. This information may have an important impact on the development and advancement of injury prevention protocols and may mitigate risk to soccer athletes who utilize the program. This knowledge can provide critical insight to help reduce the rate of ACL injury in male soccer players, improve the efficacy of existing ACL injury prevention protocols, and improve secondary prevention strategies. Future studies should investigate the efficacy of the FIFA 11+ program with respect to ACL injury prevention in female collegiate players. In addition, the cost-effectiveness of utilizing this prevention program in the collegiate cohort should be analyzed to determine if the cost associated with program implementation is justified.

Acknowledgments We thank all of the Division I and Division II NCAA-certified athletic trainers, coaching staffs, and players that participated in this study. We are appreciative of their time, dedication, and compliance to the important body of research.

References

1. Agel J, Arendt EA, Bershadsky B. Anterior cruciate ligament injury in national collegiate athletic association basketball and soccer: a 13-year review. *Am J Sports Med.* 2005;33:524–530.
2. Agel J, Evans TA, Dick R, Putukian M, Marshall SW. Descriptive epidemiology of collegiate men's soccer injuries: National Collegiate Athletic Association Injury Surveillance System. *J Athl Train.* 2007;42:270–277.
3. Albright JC, Carptner JE, Graf BK. *Knee and Leg: Soft Tissue Trauma.* Rosemont, IL, USA: American Academy of Orthopaedic Surgeons; 1999.
4. Arendt EA, Agel J, Dick R. Anterior cruciate ligament injury patterns among collegiate men and women. *J Athl Train.* 1999;34:86–92.

5. Arnason A, Andersen TE, Holme I, Engebretsen L, Bahr R. Prevention of hamstring strains in elite soccer: an intervention study. *Scand J Med Sci Sports.* 2008;18:40–48.
6. Arnason A, Gudmundsson A, Dahl HA, Johannsson E. Soccer injuries in Iceland. *Scand J Med Sci Sports.* 1996;6:40–45.
7. Bizzini M, Junge A, Dvorak J. Implementation of the FIFA 11+ football warm up program: how to approach and convince the football associations to invest in prevention. *Br J Sports Med.* 2013;47:803–806.
8. Boden BP, Torg JS, Knowles SB, Hewett TE. Video analysis of anterior cruciate ligament injury: abnormalities in hip and ankle kinematics. *Am J Sports Med.* 2009;37:252–259.
9. Brophy RH, Backus S, Kraszewski AP, Steele BC, Ma Y, Osei D, Williams RJ. Differences between sexes in lower extremity alignment and muscle activation during soccer kick. *J Bone Joint Surg Am.* 2010;92:2050–2058.
10. Brophy RH, Stepan JG, Silvers HJ, Mandelbaum BR. Defending puts the anterior cruciate ligament at risk during soccer: a gender-based analysis. *Sports Health.* 2015;7:244–249.
11. Cerulli G, Benoit DL, Caraffa A, Ponteggia F. Proprioceptive training and prevention of anterior cruciate ligament injuries in soccer. *J Orthop Sports Phys Ther.* 2001;31:655–660; discussion 661.
12. Chhadia AM, Inacio MC, Maletis GB, Csintalan RP, Davis BR, Funahashi TT. Are meniscus and cartilage injuries related to time to anterior cruciate ligament reconstruction? *Am J Sports Med.* 2011;39:1894–1899.
13. Croisier J. Factors associated with recurrent hamstring injuries. *Sports Med.* 2004;34:681–695.
14. Daneshjoo A, Mokhtar A, Rahnama N, Yusof A. The effects of injury prevention warm-up programmes on knee strength in male soccer players. *Biol Sport.* 2013;30:281–288.
15. Dragoo JL, Braun HJ, Durham JL, Chen MR, Harris AH. Incidence and risk factors for injuries to the anterior cruciate ligament in National Collegiate Athletic Association football: data from the 2004-2005 through 2008-2009 National Collegiate Athletic Association Injury Surveillance System. *Am J Sports Med.* 2012;40:990–995.
16. Dragoo JL, Braun HJ, Harris AH. The effect of playing surface on the incidence of ACL injuries in National Collegiate Athletic Association American Football. *Knee.* 2013;20:191–195.
17. Dumont GD, Hogue GD, Padalecki JR, Okoro N, Wilson PL. Meniscal and chondral injuries associated with pediatric anterior cruciate ligament tears: relationship of treatment time and patient-specific factors. *Am J Sports Med.* 2012;40:2128–2133.
18. Ekstrand J, Gillquist J. Soccer injuries and their mechanisms: a prospective study. *Med Sci Sports Exerc.* 1983;15:267–270.
19. Ekstrand J, Gillquist J, Moller M, Oberg B, Liljedahl SO. Incidence of soccer injuries and their relation to training and team success. *Am J Sports Med.* 1983;11:63–67.
20. Ekstrand J, Hagglund M, Walden M. Epidemiology of muscle injuries in professional football (soccer). *Am J Sports Med.* 2011;39:1226–1232.
21. Ekstrand J, Hagglund M, Walden M. Injury incidence and injury patterns in professional football: the UEFA injury study. *Br J Sports Med.* 2011;45:553–558.
22. Engebretsen AH, Myklebust G, Holme I, Engebretsen L, Bahr R. Intrinsic risk factors for groin injuries among male soccer players: a prospective cohort study. *Am J Sports Med.* 2010;38:2051–2057.
23. Engebretsen AH, Myklebust G, Holme I, Engebretsen L, Bahr R. Intrinsic risk factors for hamstring injuries among male soccer players: a prospective cohort study. *Am J Sports Med.* 2010;38:1147–1153.
24. Faude O, Junge A, Kindermann W, Dvorak J. Risk factors for injuries in elite female soccer players. *Br J Sports Med.* 2006;40:785–790.
25. Gilchrist J, Mandelbaum BR, Melancon H, Ryan GW, Silvers HJ, Griffin LY, Watanabe DS, Dick RW, Dvorak J. A randomized controlled trial to prevent noncontact anterior cruciate ligament injury in female collegiate soccer players. *Am J Sports Med.* 2008;36:1476–1483.
26. Giza E, Mithöfer K, Farrell L, Zarins B, Gill T. Injuries in women's professional soccer. *Br J Sports Med.* 2005;39:212–216.
27. Giza E, Silvers HJ, Mandelbaum BR. Anterior cruciate ligament tear prevention in the female athlete. *Curr Sports Med Rep.* 2005;4:109–111.
28. Gordon MD SM. *Anterior Cruciate Ligament Injuries.* Rosemont, IL, USA: American Academy of Orthopaedic Surgeons; 2004.
29. Griffin LY, Albohm MJ, Arendt EA, Bahr R, Beynnon BD, Demaio M, Dick RW, Engebretsen L, Garrett WE Jr, Hannafin JA, Hewett TE, Huston LJ, Ireland ML, Johnson RJ, Lephart S, Mandelbaum BR, Mann BJ, Marks PH, Marshall SW, Myklebust G, Noyes FR, Powers C, Shields C Jr, Shultz SJ, Silvers H, Slauterbeck J, Taylor DC, Teitz CC, Wojtys EM, Yu B. Understanding and preventing noncontact anterior cruciate ligament injuries: a review of the Hunt Valley II meeting, January 2005. *Am J Sports Med.* 2006;34:1512–1532.
30. Grooms DR, Palmer T, Onate JA, Myer GD, Grindstaff T. Soccer-specific warm-up and lower extremity injury rates in collegiate male soccer players. *J Athl Train.* 2013;48:782–789.
31. Hagglund M, Walden M, Bahr R, Ekstrand J. Methods for epidemiological study of injuries to professional football players: developing the UEFA model. *Br J Sports Med.* 2005;39:340–346.
32. Hewett TE, Lindenfeld TN, Riccobene JV, Noyes FR. The effect of neuromuscular training on the incidence of knee injury in female athletes. A prospective study. *Am J Sports Med.* 1999;27:699–706.
33. Hewett TE, Myer GD, Ford KR. Reducing knee and anterior cruciate ligament injuries among female athletes: a systematic review of neuromuscular training interventions. *J Knee Surg.* 2005;18:82–88.
34. Hootman JM, Dick R, Agel J. Epidemiology of collegiate injuries for 15 sports: summary and recommendations for injury prevention initiatives. *J Athl Train.* 2007;42:311–319.
35. Kristenson K, Bjorneboe J, Walden M, Andersen TE, Ekstrand J, Hagglund M. The Nordic Football Injury Audit: higher injury rates for professional football clubs with third-generation artificial turf at their home venue. *Br J Sports Med.* 2013;47:775–781.
36. Lohmander LS, Englund PM, Dahl LL, Roos EM. The long-term consequence of anterior cruciate ligament and meniscus injuries: osteoarthritis. *Am J Sports Med.* 2007;35:1756–1769.
37. Longo UG, Loppini M, Berton A, Marinozzi A, Maffulli N, Denaro V. The FIFA 11+ program is effective in preventing injuries in elite male basketball players: a cluster randomized controlled trial. *Am J Sports Med.* 2012;40:996–1005.
38. Magnussen RA, Pedroza AD, Donaldson CT, Flanigan DC, Kaeding CC. Time from ACL injury to reconstruction and the prevalence of additional intra-articular pathology: is patient age an important factor? *Knee Surg Sports Traumatol Arthrosc.* 2013;21:2029–2034.
39. Mandelbaum BR, Silvers HJ, Watanabe DS, Knarr JF, Thomas SD, Griffin LY, Kirkendall DT, Garrett W Jr. Effectiveness of a neuromuscular and proprioceptive training program in preventing anterior cruciate ligament injuries in female athletes: 2-year follow-up. *Am J Sports Med.* 2005;33:1003–1010.
40. Myklebust G, Engebretsen L, Braekken IH, Skjolberg A, Olsen OE, Bahr R. Prevention of anterior cruciate ligament injuries in female team handball players: a prospective intervention study over three seasons. *Clin J Sports Med.* 2003;13:71–78.
41. Myklebust G, Engebretsen L, Braekken IH, Skjolberg A, Olsen OE, Bahr R. Prevention of noncontact anterior cruciate ligament

injuries in elite and adolescent female team handball athletes. *Instr Course Lect.* 2007;56:407–418.
42. Nigg BM, Yeadon MR. Biomechanical aspects of playing surfaces. *J Sports Sci.* 1987;5:117–145.
43. Olsen OE, Myklebust G, Engebretsen L, Holme I, Bahr R. Relationship between floor type and risk of ACL injury in team handball. *Scand J Med Sci Sports.* 2003;13:299–304.
44. Owoeye OB, Akinbo SR, Tella BA, Olawale OA. Efficacy of the FIFA 11+ warm-up programme in male youth football: a cluster randomised controlled trial. *J Sports Sci Med.* 2014;13:321–328.
45. Salgado E, Ribeiro F, Oliveira J. Joint-position sense is altered by football pre-participation warm-up exercise and match induced fatigue. *Knee.* 2015;22:243–248.
46. Silvers-Granelli H, Mandelbaum B, Adeniji O, Insler S, Bizzini M, Pohlig R, Junge A, Snyder-Mackler L, Dvorak J. Efficacy of the FIFA 11+ injury prevention program in the collegiate male soccer player. *Am J Sports Med.* 2015;43:2628–2637.
47. Soligard T, Myklebust G, Steffen K, Holme I, Silvers H, Bizzini M, Junge A, Dvorak J, Bahr R, Andersen TE. Comprehensive warm-up programme to prevent injuries in young female footballers: cluster randomised controlled trial. *BMJ.* 2008;337:a2469.
48. Stanley LE, Kerr ZY, Dompier TP, Padua DA. Sex differences in the incidence of anterior cruciate ligament, medial collateral ligament, and meniscal injuries in collegiate and high school sports: 2009-2010 through 2013-2014. *Am J Sports Med.* 2016;44:1565–1572.
49. Steffen K, Meeuwisse WH, Romiti M, Kang J, McKay C, Bizzini M, Dvorak J, Finch C, Myklebust G, Emery CA. Evaluation of how different implementation strategies of an injury prevention programme (FIFA 11+) impact team adherence and injury risk in Canadian female youth football players: a cluster-randomised trial. *Br J Sports Med.* 2013;47:480–487.
50. Steffen K, Myklebust G, Olsen OE, Holme I, Bahr R. Preventing injuries in female youth football—a cluster-randomized controlled trial. *Scand J Med Sci Sports.* 2008;18:605–614.
51. Torg JS, Quedenfeld TC, Landau S. The shoe-surface interface and its relationship to football knee injuries. *J Sports Med.* 1974;2:261–269.
52. Walden M, Hagglund M, Ekstrand J. High risk of new knee injury in elite footballers with previous anterior cruciate ligament injury. *Br J Sports Med.* 2006;40:158–162; discussion 158–162.
53. Walden M, Krosshaug T, Bjorneboe J, Andersen TE, Faul O, Hagglund M. Three distinct mechanisms predominate in non-contact anterior cruciate ligament injuries in male professional football players: a systematic video analysis of 39 cases. *Br J Sports Med.* 2015;49:1452–1460.
