# Supplementary material for: Comparative Effectiveness of Multi-Component, Exercise-Based Interventions for Preventing Soccer-Related Musculoskeletal Injuries: A Systematic Review and Meta-Analysis
Source: Healthcare (Basel). 2025 Mar 29;13(7):765. doi: 10.3390/healthcare13070765 (PMC11988859; doi:10.3390/healthcare13070765)
Supplement: Supplementary file 1 [file healthcare-13-00765-s001.zip › Additional material Included literature/soligard2008.pdf]

BMJ

RESEARCH

Comprehensive warm-up programme to prevent injuries in young female footballers: cluster randomised controlled trial

Torbjørn Soligard, PhD student,[1] Grethe Myklebust, associate professor,[1] Kathrin Steffen, research fellow,[1] Ingar Holme, professor,[1] Holly Silvers, physical therapist,[2] Mario Bizzini, physical therapist,[3] Astrid Junge, associate professor,[3] Jiri Dvorak, professor,[3] Roald Bahr, professor,[1] Thor Einar Andersen, associate professor[1]

[1]Oslo Sports Trauma Research Centre, Norwegian School of Sport Sciences, PO Box 4014 Ullevaal Stadion, 0806 Oslo, Norway

[2]Santa Monica Orthopaedic and Sports Medicine Research Foundation, 1919 Santa Monica Blvd, Suite 350, Santa Monica, CA 90404 USA

[3]FIFA Medical Assessment and Research Centre, Schulthess Clinic, Lengghalde 2, CH-8008 Zurich, Switzerland

Correspondence to: T Soligard torbjorn.soligard@nih.no

Cite this as: *BMJ* 2008;337:a2469
doi:10.1136/bmj.a2469

ABSTRACT

Objective To examine the effect of a comprehensive warm-up programme designed to reduce the risk of injuries in female youth football.

Design Cluster randomised controlled trial with clubs as the unit of randomisation.

Setting 125 football clubs from the south, east, and middle of Norway (65 clusters in the intervention group; 60 in the control group) followed for one league season (eight months).

Participants 1892 female players aged 13-17 (1055 players in the intervention group; 837 players in the control group).

Intervention A comprehensive warm-up programme to improve strength, awareness, and neuromuscular control during static and dynamic movements.

Main outcome measure Injuries to the lower extremity (foot, ankle, lower leg, knee, thigh, groin, and hip).

Results During one season, 264 players had relevant injuries: 121 players in the intervention group and 143 in the control group (rate ratio 0.71, 95% confidence interval 0.49 to 1.03). In the intervention group there was a significantly lower risk of injuries overall (0.68, 0.48 to 0.98), overuse injuries (0.47, 0.26 to 0.85), and severe injuries (0.55, 0.36 to 0.83).

Conclusion Though the primary outcome of reduction in lower extremity injury did not reach significance, the risk of severe injuries, overuse injuries, and injuries overall was reduced. This indicates that a structured warm-up programme can prevent injuries in young female football players.

Trial registration ISRCTN10306290.

INTRODUCTION

Football (soccer) is the most popular team sport in the world. There are already more than 265 million registered players, and the number of participants is continuing to grow.[1] In particular, the number of women players is increasing rapidly.[1] Playing football, however, entails a substantial risk of injury, and studies on elite and non-elite female footballers have reported rates of injury similar to those in men,[2-11] the most common being injuries to the knee and ankle ligament and thigh muscle strains.[2-9 11 12] Women might even be at greater risk of serious injury than men; the rate of anterior cruciate ligament injuries is three to five times higher for girls than for boys.[13 14]

The high injury rate among football players in general and female players in particular constitutes a considerable problem for the player, the club, and—given the popularity of the sport—for society at large. Health consequences are seen not just in the short term but also in the dramatic increase in the risk of early osteoarthritis.[15-17] Despite the urgent need to develop programmes to prevent knee and ankle injuries in footballers, there exist only a few small or non-randomised studies on prevention of injury in female football players.[18-20]

In a recent randomised controlled trial, we examined the effect of a structured training programme ("The 11")[21] over one season among 2000 female players aged 13-17.[22] The intervention consisted of exercises focusing on core stability, balance, dynamic stabilisation, and eccentric hamstring strength. We found no difference in the injury risk between the intervention group and control group, though the study was limited by low compliance among the intervention teams.

This led us to develop an exercise programme to improve both the preventive effect of the programme and the compliance of coaches and players. The revised programme ("The 11+") included key exercises and additional exercises to provide variation and progression. It also included a new set of structured running exercises that made it better suited as a comprehensive warm-up programme for training and matches.

We conducted a randomised controlled trial to examine the effect of the revised programme on rates of lower extremity injury in young female footballers. To minimise contamination bias within clubs, we used a cluster randomised design.

METHODS

We randomised 125 clubs who agreed to participate in the study to the intervention or control group. All teams from one club were randomised to the same treatment arm. Five clubs included two teams each. The statistician (IH) who conducted the randomisation did not take part in the intervention. Box 1 provides details of the procedure used to recruit clubs.

We informed clubs allocated to the intervention group that they would receive a programme of warm-up exercises used to prevent injuries and enhance performance. We asked the clubs in the control group to warm up as usual during the season and informed them that, if the intervention programme prevented injuries, they would receive the same programme as the intervention group in the subsequent season.

Box 1 Recruitment of clubs to the study

- All of the 181 clubs in the 15-16 year divisions from the south, east, and middle of Norway, organised by the regional districts of the Norwegian Football Association, received an invitation to participate in the study during one eight month season (March to October 2007)
- To be included in the study, clubs had to carry out at least two training sessions a week in addition to match play. The clubs practised two to five times a week and played between 15 and 30 matches during the season
- All clubs were recruited in January and February 2007. Club enrolment registries for the 2007 league system were obtained from the regional districts of the Norwegian Football Association, and coaches were informed by telephone about the purpose and the design of the study. After oral consent, a letter containing a more thorough description of the study and a study enrolment return form was sent out to the coaches, who also informed the players
- Player participation was voluntary

Intervention

An expert group convened by the international football federation (FIFA), with representatives from the Oslo Sports Trauma Research Center, the Santa Monica Orthopaedic and Sports Medicine Research Foundation, and the FIFA Medical Assessment and Research Centre, developed the warm-up programme. Before the start of the study one club tested the programme. It consisted of three parts (table 1). The initial part was running exercises at slow speed combined with active stretching and controlled contacts with a partner. The running course included six to ten pairs of cones (depending on the number of players) about five to six metres apart (length and width). The second part consisted of six different sets of exercises; these included strength (fig 1), balance, and jumping exercises, each with three levels of increasing difficulty. The final part was speed running combined with football specific movements with sudden changes in direction.

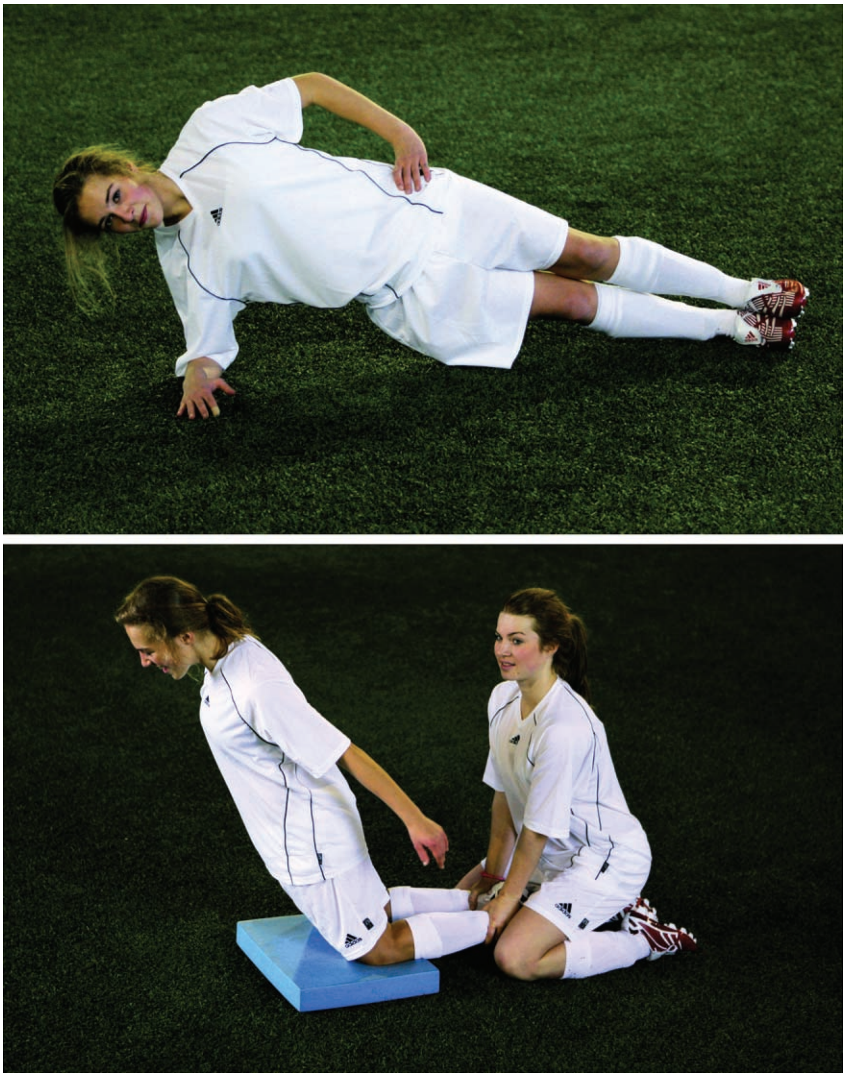

Fig 1 | Two examples of strength exercises. Top: side plank exercise. Bottom: the "Nordic hamstring lower"

At the start of the pre-season, February to mid-April 2007, we invited the coaches and team captains from all clubs in the intervention group to a three hour instructional course in which we introduced the warm-up programme. Instructors from the Oslo Sports Trauma Research Centre arranged courses at different locations in each of the eight regional districts. The instructors had been familiarised with the programme during a seminar, where they received theoretical and practical training in the programme and instruction in how to teach the exercises to the coaches and team captains.

The coaches received an instructional DVD showing all of the exercises in the programme, a loose leaf exercise book, and small exercise cards attached to a neck strap. In addition, the coaches and every player received a poster explaining every exercise. The information material detailed each exercise and explained the proper form for each, as well as common biomechanical mistakes. It also described the principles of progression in the exercise prescription. We asked the coaches to use the complete exercise

programme as the warm up for every training session throughout the season and to use the running exercises in the programme as part of their warm up for every match.

When introducing the programme to the clubs, our main focus was to improve awareness and neuromuscular control during standing, running, planting, cutting, jumping, and landing. We encouraged the players to concentrate on the quality of their movements and put emphasis on core stability, hip control, and proper knee alignment to avoid excessive knee valgus during both static and dynamic movements (fig 2). We asked the coaches and the players to watch each other closely and give feedback during training. Once players were familiar with the exercises the programme took about 20 minutes to complete.

Throughout the season, researchers contacted the coaches regularly by email and telephone; this allowed the coaches to ask questions and provide feedback with respect to the warm-up programme and injury and exposure registration. It also helped us to identify clubs that were not complying with the protocol or where additional motivational measures, such as site visits, were needed to increase compliance. Clubs in both groups were offered an incentive in the form of high quality footballs, provided they completed data registration throughout the study period. Despite these measures, 13 clubs in the intervention group did not start the warm-up programme nor did they deliver any data on injury or exposure (fig 3). Nineteen clubs in the control group did not provide any data.

Table 1 | Revised warm-up exercise programme used to prevent injury in young female footballers

Exercise	Repetitions
**I. Running exercises, 8 minutes (opening warm up, in pairs; course consists of 6-10 pairs of parallel cones):**	
Running, straight ahead	2
Running, hip out	2
Running, hip in	2
Running, circling	2
Running and jumping	2
Running, quick run	2
**II. Strength, plyometrics, balance, 10 minutes (one of three exercise progression levels each training session):**	
The plank:	
Level 1: both legs	3×20-30 seconds
Level 2: alternate legs	3×20-30 seconds
Level 3: one leg lift	3×20-30 seconds
Side plank:	
Level 1: static	3×20-30 seconds (each side)
Level 2: dynamic	3×20-30 seconds (each side)
Level 3: with leg lift	3×20-30 seconds (each side)
Nordic hamstring lower:	
Level 1	3-5
Level 2	7-10
Level 3	12-15
Single leg balance:	
Level 1: holding ball	2×30 seconds (each leg)
Level 2: throwing ball with partner	2×30 seconds (each leg)
Level 3: testing partner	2×30 seconds (each leg)
Squats:	
Level 1: with heels raised	2×30 seconds
Level 2: walking lunges	2×30 seconds
Level 3: one leg squats	2×10 (each leg)
Jumping:	
Level 1: vertical jumps	2×30 seconds
Level 2: lateral jumps	2×30 seconds
Level 3: box jumps	2×30 seconds
**III. Running exercises, 2 minutes (final warm up)**	
Running over pitch	2
Bounding run	2
Running and cutting	2

Outcome measures

We defined the primary outcome as an injury to the lower extremity (foot, ankle, lower leg, knee, thigh, groin, and hip) and secondary outcomes as any injury, or an injury to the ankle, knee, or other body parts. We included all injuries reported after an intervention club had completed the first prevention training session (matched with the same date in a control club) to compare the risk of injury between the groups.

Exposure and injury registration

The coaches reported injuries and details of an individual player's participation for each training session and match, as well as to what extent the warm-up programme was carried out each session (intervention clubs) on weekly registration forms throughout the study period. These were submitted by email, mail, or fax to the research centre. Data on players who dropped out during the study period were included for the entire period of their participation.

At the research centre one physical therapist and one medical student, who were blinded to group allocation, recorded injuries. They were given specific training on the protocols for injury classification and injury definitions (box 2) before the start of the injury registration period. Every injured player was contacted to assess aspects of the injury based on a standardised injury questionnaire.[23] In most cases, players were contacted within four weeks (range one day to five months) after the injury. Box 2 shows the definitions we used to register injuries. These are in accordance with the consensus statement on injury definitions and data collection procedures.[24]

Sample size

We calculated our sample size on the basis of data on incidence of injury in young female footballers in Norway during the 2005 season.[22] We estimated that 16% would incur an injury to the lower extremities and about 10-12% would injure a knee or ankle during one season. Given an estimated inflation factor for cluster effects because of randomisation by clubs of 1.8,[22] 900 players in each group would provide an acceptable power of 0.86 at the 5% significance level to detect a 40% reduction in the number of players with a lower extremity injury. Our model was based on 18 players

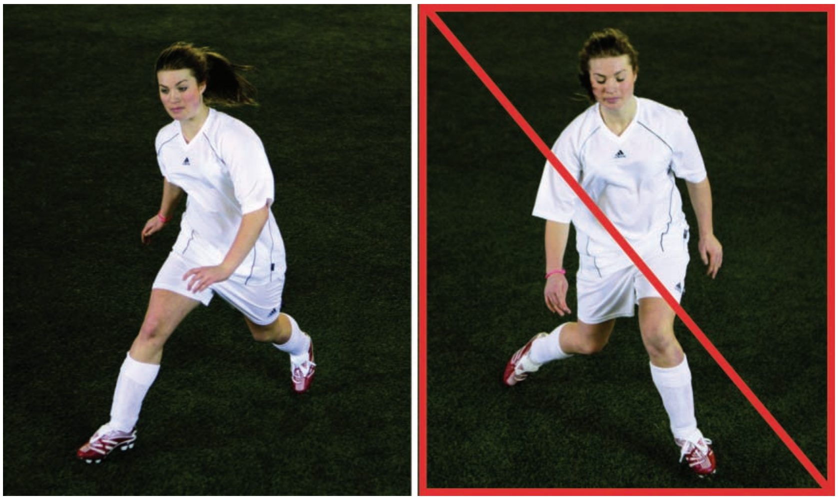

Fig 2 | Example of running exercise illustrating key objectives of all running, jumping, cutting, and landing exercises: core stability and correct lower extremity alignment. Left: correct technique; right: incorrect technique with pelvic tilt and knee valgus alignment to right

per club and a dropout rate of 15%, which means that we needed to include about 120 clubs with 2150 players.

Statistical methods

We conducted all statistical analyses according to a prespecified plan using Stata, version 10.0 (StataCorp, College Station, TX). We used the rate ratio of the risk of injury according to the intention to treat principle to compare the risk of an injury in the two groups. We used Cox regression for the primary and secondary outcomes and the robust calculation method of the variance-covariance matrix,[25] taking the cluster randomisation by clubs into account. Rate ratios were tested with the Wald test. One way analysis of variance was used to estimate the intracluster correlation coefficient to obtain estimates of the inflation factor for comparison with planned sample size. To calculate the number needed to prevent one injury we used the inverse of the difference between percentages of injured players in the two groups. We used one minus survival plots based on the Cox regression to evaluate possible delays of the injury prevention effects of the programme in the intervention group compared with the control group. We used a Poisson model, taking cluster effects into account, as a per protocol analysis to compare the rate ratios of risk of injury between players in the intervention group stratified into thirds of compliance according to the number of prevention sessions completed: low, intermediate, and high. The summary measure of injury incidence (i) was calculated according to the formula $i=n/e$, where n is the number of injuries during the study period and e the sum of exposure time expressed in player hours of match, training, or in total. We calculated confidence intervals of the rate ratio of the number of injuries in the intervention and control groups by a simple Poisson model, assuming constant hazard per group. Injury incidences are presented as means with standard errors. Rate ratios are presented with 95% confidence intervals. We regarded two tailed P values ≤0.05 as significant.

RESULTS

The final sample consisted of 52 clubs (1055 players) in the intervention group and 41 clubs (837 players) in the control group (fig 3). The players in the two groups were similar in age (15.4 (SD 0.7) years in both groups) and age distribution. The dropout rate was similar between the groups (23 (2.1%) *v* 24 (2.9%)).

Exposure and injury characteristics

Those in the intervention group played 49 899 hours of football (16 057 hours of matches and 33 842 hours of practice). The figure for the control group was 45 428 hours (14 342 and 31 086). During the eight month season, 301 (16%) of the 1892 players included in the study sustained a total of 376 injuries; 161 in the intervention group, 215 in the control group. There were 299 (80%) acute injuries and 77 (20%) overuse injuries. The overall incidence of injuries was 3.9 (SD 0.2) per 1000 player hours (8.1 (SD 0.5) in matches and 1.9 (SD 0.2) in training).

Effect of revised injury prevention programme

The rate ratio for players with a lower extremity injury between the intervention and the control group was 0.71 (0.49 to 1.03, P=0.072). There was a significantly lower risk of injuries overall, overuse injuries, and severe injuries in the intervention group (table 2). The reduction in the risk of match injuries, training injuries, knee injuries, and acute injuries (from 26% to 38%) did not reach significance. The degree of clustering at the club level (intracluster correlation coefficient) ranged from 0.028 to 0.096. The estimated inflation factor varied from 1.54 to 2.86. The number needed to treat to prevent one injury varied from 15 to 63 players. Figure 4 shows survival curves for lower extremity injuries and severe injuries in the two groups.

The mean age of injured players was 15.4 (SD 0.6) in the intervention group and 15.5 (SD 0.7) in the control group. Table 3 shows the most commonly injured

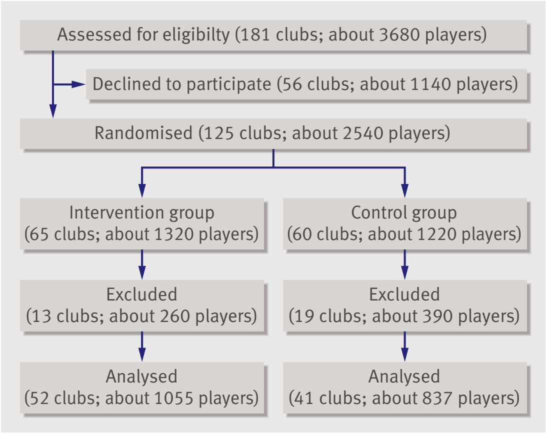

Fig 3 | Flow of club clusters and players through study

body parts and the type of acute and overuse injuries for both groups.

Compared with the control group, significantly fewer players in the intervention group had two or more injuries (rate ratio 0.51, 95% confidence interval 0.29 to 0.87), while a reduction in the risk of re-injuries did not reach significance (0.46, 0.20 to 1.01). Table 4 shows the severity distribution for different types of injury. The overall rate of injuries, as well as the rate of match injuries, training injuries, overuse injuries, and acute injuries, differed significantly. The rate of severe injuries, severe overuse injuries, and severe acute injuries was significantly lower in the intervention group.

Compliance with programme

The 52 clubs in the intervention group performed the injury prevention programme for 44 (SD 22, range 11-104) sessions (77%) throughout the season. The average player attendance for matches and training sessions was 11.7 (57.9% of all the players on the roster), which was similar to the average number who participated in the warm-up programme (12.0 (59.4%) of all the players on the roster). The average attendance in the control group was 12.2 (59.8% of all the players on the roster). None of the clubs in the control group reported performing structured warm-up exercises comparable with the intervention. The risk of injury was 35% lower in intervention players in the third with the highest compliance (2.6 (20. to 3.2) injuries/1000 player hours, mean (range 33-95) 49.2 sessions) compared with players in the intermediate third (4.0 (3.0 to 5.0) injuries/1000 player hours, mean 23.4 (15-32) sessions) (rate ratio 0.65, 0.44 to 0.94, P=0.02). The 32% reduction in risk of injury compared with the third with the lowest compliance (3.7 (2.2 to 5.3) injuries/1000 player hours, mean 7.7 (0-14) sessions) did not reach significance (rate ratio 0.68, 0.41 to 1.12, P=0.13).

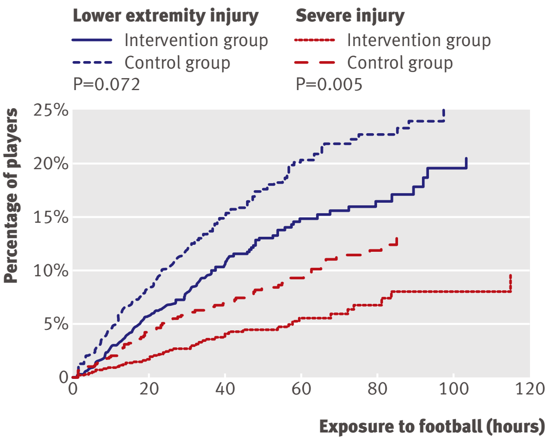

Fig 4 | Survival curves based on Cox regression for players with lower extremity injury and severe injury

Box 2 Operational definitions used in registration of injury

Reportable injury

- An injury occurred during a scheduled match or training session, causing the player to be unable to fully take part in the next match or training session.

Player

- A player was entered into the study if she was registered by the coach on the club roster to take part in the club's team competing in the 15 or 16 year divisions.

Return to participation

- The player was defined as injured until she was fully fit to take part in all types of training and matches.

Type of injury

- Acute: injury with sudden onset associated with known trauma
- Overuse: injury with gradual onset without known trauma
- Re-injury: injury of the same type and location sustained previously.

Severity

- Minimal injuries: absence from match and training for 1-3 days
- Mild injuries: absence from match and training for 4-7 days
- Moderate injuries: absence from match and training for 8-28 days
- Severe injuries: absence from match and training for more than 28 days.

Exposure

- Match exposure: hours of matches.
- Training exposure: hours of training.

In almost all cases, a doctor examined players with moderate or severe injuries. If there was any doubt about the diagnosis the player was referred to a sports medicine centre for follow-up, which often included imaging studies or arthroscopic examination. In cases of minimal or mild injuries, players were examined by a local physiotherapist or the coach, or not at all. None of the injured players was examined or treated by any of the authors or injury recorders involved in the study, and we had no influence on the time it took a player to return to club activities.

DISCUSSION

This randomised controlled trial of a structured warm-up programme in young female footballers showed that the risk of injury can be reduced by about one third and severe injuries by as much as one half. Although the rate ratios for the different outcome variables indicated a consistent effect on risk of injury across most types of injury, the primary outcome—lower extremity injury—did not reach significance when we adjusted for the cluster sampling. There was, however, a significant reduction in several secondary outcome variables, including the rate of severe injuries, overuse injuries, and injuries overall.

The effect of various intervention programmes designed to reduce the risk of injury to the lower extremities in young female footballers has been studied previously.[18-20 22] These studies, however, were either non-randomised, had small sample sizes, had low compliance, or had other important limitations.

Methodological considerations

The trial took place in the 15 and 16 year divisions from the south, east, and middle of Norway and recruited 69% of all clubs and players organised by the Norwegian Football Association in these areas. Of the 181 clubs assessed for eligibility, 56 declined to participate and 125 were randomised. During the recruitment of clubs, the most common barrier to participation that coaches reported was the additional work of registering and reporting data weekly. Other less common reasons for non-participation included a

Table 2 | Intention to treat analysis of warm-up exercise programme (intervention) in young female footballers. Values are numbers (percentages) of injured players

	Intervention group (n=1055)	Control group (n=837)	Intracluster correlation coefficient*	Inflation factor*	NNT	Rate ratio (95% CI)†	P value
All injuries	135 (13.0)	166 (19.8)	0.096	2.86	15	0.68 (0.48 to 0.98)	0.041
Match injuries	96 (9.1)	114 (13.6)	0.045	1.87	22	0.72 (0.52 to 1.00)	0.051
Training injuries	50 (4.7)	63 (7.5)	0.044	1.86	36	0.68 (0.41 to 1.11)	0.120
Lower extremity injuries	121 (11.5)	143 (17.1)	0.088	2.70	18	0.71 (0.49 to 1.03)	0.072
Knee injuries	33 (3.1)	47 (5.6)	0.028	1.54	40	0.62 (0.36 to 1.05)	0.079
Ankle injuries	45 (4.3)	49 (5.9)	0.026	1.50	63	0.81 (0.50 to 1.30)	0.378
Acute injuries	112 (10.6)	130 (15.5)	0.070	2.35	20	0.74 (0.51 to 1.08)	0.110
Overuse injuries	27 (2.6)	48 (5.7)	0.040	1.76	32	0.47 (0.26 to 0.85)	0.012
Severe injuries	45 (4.3)	72 (8.6)	0.028	1.54	23	0.55 (0.36 to 0.83)	0.005

NNT=number needed to treat.
*Generalised estimating equation model with clubs as cluster unit.
†Cox model calculated according to method of Lin and Wei,[25] which takes cluster randomisation into account.

reluctance to use the same warm-up programme for every training session and match and low priority for injury prevention. Thus, although we recruited a high proportion of eligible teams, the final sample probably included teams with more dedicated coaches. After inclusion, we had to exclude 13 intervention clubs and 19 control clubs because they did not deliver any data on injury or exposure. In most cases the coaches were volunteers, such as parents, and the most common reason for not reporting any data was the additional work of registering and reporting data weekly. Despite the fact that they were informed about the study both orally and in writing before signing up for participation, after randomisation many of the coaches in the excluded clubs decided that the extra work would be too time consuming. Additionally, the disappointment of being randomised to the control group and hence not receiving the warm-up programme might explain the somewhat higher number of clubs we had to exclude from the control group. We think it is unlikely that the excluded clubs had coaches who were less "safety conscious" than the coaches in the clubs that completed the study. Our experience with this and several other studies is that, at the outset, few coaches consider injuries as a factor they can influence.

With respect to the internal validity, we found no differences between the two groups in their training or match exposure during the study. The coaches in both groups reported injuries and individual training and match participation prospectively on weekly registration forms according to pre-specified protocols and accepted injury definitions.[24] Because we recorded individual exposure we could adjust for playing time, which can vary greatly among players. This adjustment might be important as the best players play more games than substitutes and they might also train more. Individual exposure also takes censorship into account, such as abbreviated lengths of follow-up for reasons other than injury (such as illness, moving, quitting the sport).[26] Another advantage of this approach is that it provides accurate data about each player's exposure to the intervention, in this case the injury prevention programme.

Injury recorders, who were blinded to group allocation, interviewed the injured players using a standardised injury questionnaire as soon as possible after the weekly registration form was received. Even so, some coaches might have overlooked injuries, although this is less likely for more severe injuries such as knee and ankle sprains. Our definition of reportable injury embraced any injury that occurred during a scheduled match or training session, causing the player to be unable to fully take part in the next match or training session.[24] Given the individual activity logs kept by the coaches, it is therefore unlikely that injuries would go unreported, and we see no reason to expect a reporting bias between the groups. Our method should ensure good reliability and validity of the injury and exposure data.

The intention to treat analysis showed that the inflation factor for cluster effects was higher than our power calculation (2.7 *v* 1.8). We estimated the inflation factor on the incidence of lower extremity injuries in our previous study on a similar sample.[22] Yet our results indicate that we might have underestimated the number of players we needed to establish possible intervention effects. This is also supported by the larger confidence intervals of the rate ratios calculated from the Cox regression analysis (taking cluster randomisation into account) than the simpler Poisson regression model (assuming constant hazard per group). In addition, our power calculation was based on a dropout rate of 15% when the actual dropout rate was 25.6%.

Compliance

In our previous intervention study we tested the effect of a training programme,[21] in a similar cohort of young female footballers.[22] We were encouraged that compliance in the current trial was higher than with the previous programme (77% *v* 52%). One key objective for the revision was to improve the compliance among coaches and players, and, with this in mind, the revised programme was expanded with more exercises to provide variation and progression. It also included a new set of structured running exercises to make it better suited as a stand alone warm-up programme for

training and matches. In addition, the first part of the programme included exercises with a partner, which seemed to appeal to the players.

The resources used to promote the programme among the intervention teams were moderate so it should be possible to replicate implementation in large scale nationwide programmes. The coaches and team captains were introduced to the programme during one three hour training session. To boost compliance we also developed new information material for coaches and players: a DVD showing all the exercises, a poster, a loose leaf exercise book, and small exercise cards attached to a handy neck strap that the coaches could bring to the training field. It was up to the coaches and team captains, however, to teach the programme to the players on the roster. Furthermore, the clubs received no follow-up visits throughout the season to refresh coaching skills or give players feedback on their performance. Despite the moderate efforts to promote the programme, compliance was good and we saw effects on the risk of injury. It should be possible to implement the programme at the community level by including injury prevention as part of basic coaching education and making educational material such as that developed for the current study available to teams, coaches, players, and parents.

The technical nature of many of the exercises in the programme required players to focus during training to gain the intended benefit. Site visits indicated that not all of the players seemed to concentrate fully on the performance of the exercises, which might be expected for this age group. Furthermore, the compliance logs documented that not all clubs completed the requested minimum of two training sessions a week. We included all clubs and players in the intention to treat analysis, which means that the preventive effect of the programme might be higher than reported. This is supported by subgroup analyses within the intervention group, indicating a trend towards a lower risk of injury among the most compliant players.

Structured programme of warm-up exercises to prevent injuries

The programme was developed on the basis of "The 11" programme[21] and the prevent injury and enhance performance (PEP) programme,[20] combined with running activities at the start and the end.[27] The running exercises were chosen not just to make the programme more suitable as a warm up but also to teach proper knee control and core stability during cutting and landing. Furthermore, the revised exercises include both variety and progression of difficulty. These elements were absent from the previously tested training programme[22] but exist in other successful prevention programmes.[27-30]

The focus on core stability, balance, and neuromuscular control as well as hip control and knee alignment that avoids excessive knee valgus during both static and dynamic movements is a feature of earlier intervention studies.[18 20 27-29 31] This rationale is justified by data from studies on the mechanisms of anterior cruciate ligament injuries.[32-36] These studies indicate that players could benefit from not allowing the knee to sag medially during football specific movements, when suddenly changing speed, or when being tackled by opponents. The programme therefore focused on proper biomechanical technique and improved awareness and control during standing, running, planting, cutting, jumping, and landing.

The programme included a set of balance exercises, and during single leg balance training the players were also purposely pushed off balance; this provided an

Table 3 | Most commonly injured body parts and most common type of acute and overuse injuries in young female footballers according to use of warm-up exercise programme (intervention). Values are numbers (percentages) of injuries unless otherwise indicated. Incidence is reported as number of injuries per 1000 player hours, with standard errors

	Intervention group (n=1055)		Control group (n=837)		Rate ratio (95% CI)*	P value (z test)
	Injuries	Incidence	Injuries	Incidence		
Body category:						
Knee	35 (21.7)	0.7 (0.1)	58 (27.0)	1.3 (0.2)	0.55 (0.36 to 0.84)	0.005
Ankle	51 (31.7)	1.0 (0.1)	52 (24.2)	1.1 (0.2)	0.89 (0.61 to 1.31)	0.562
Leg	14 (8.7)	0.3 (0.1)	22 (10.2)	0.5 (0.1)	0.58 (0.30 to 1.13)	0.111
Anterior thigh	9 (5.6)	0.2 (0.1)	9 (4.2)	0.2 (0.1)	0.91 (0.36 to 2.29)	0.842
Posterior thigh (hamstring)	5 (3.1)	0.1 (0.0)	8 (3.7)	0.2 (0.1)	0.57 (0.18 to 1.74)	0.322
Hip/groin	10 (6.2)	0.2 (0.1)	9 (4.2)	0.2 (0.1)	1.01 (0.41 to 2.49)	0.984
Acute injuries:						
Sprains	65 (47.8)	1.3 (0.2)	76 (46.6)	1.7 (0.2)	0.78 (0.56 to 1.08)	0.139
Strains	25 (18.4)	0.5 (0.1)	28 (17.2)	0.6 (0.1)	0.81 (0.47 to 1.39)	0.453
Contusions	16 (11.8)	0.3 (0.1)	33 (20.2)	0.7 (0.1)	0.44 (0.24 to 0.80)	0.007
Fractures	14 (10.3)	0.3 (0.1)	7 (4.3)	0.2 (0.1)	1.82 (0.74 to 4.51)	0.194
Overuse injuries:						
Lower extremity tendon pain	11 (44.0)	0.2 (0.1)	21 (40.4)	0.5 (0.1)	0.48 (0.23 to 0.99)	0.047
Low back pain	1 (3.4)	0.0 (0.0)	8 (14.3)	0.2 (0.0)	0.11 (0.01 to 0.91)	0.040
Anterior lower leg pain (periostitis)	9 (36.0)	0.2 (0.1)	12 (23.1)	0.3 (0.1)	0.68 (0.29 to 1.62)	0.384

*Rate ratio obtained from Poisson model.

additional challenge to the ability to maintain a stable core and proper alignment. Previous studies in footballers have shown that the rate of anterior cruciate ligament injuries can be reduced by improving dynamic and static balance, neuromuscular control, and proprioception.[20 28] Our programme also included strength exercises, such as the "Nordic hamstring lower," which has been shown to increase eccentric hamstring muscle strength[37] and decrease the rate of hamstring strain injuries.[38] The hamstrings can act as agonists to the anterior cruciate ligament during stop and jump tasks,[39-41] at least at knee flexion angles above 30°.[42-44] Stronger hamstring muscles might therefore prevent injuries to the ligament, but this theory has never been tested directly. Based on data from volleyball[39 45] and team handball,[27 29] we also encouraged players to reduce the impact of landings with increased hip and knee flexion and to land on two legs rather than one.

Our prevention programme is multifaceted and addresses many factors that could be related to the risk of injury (jogging and active stretching for general warm up, strength, balance, awareness of vulnerable hip and knee positions, technique of planting, cutting, landing, and running), and it is not possible to determine exactly which exercises or factors might have been responsible for the observed effects. Further studies are needed to determine what the key components are so that future programmes might require less time and effort.

Except for a few reports from coaches on muscular soreness in the beginning of the intervention period and one report about a minor hamstring strain, we observed no negative effects of the programme.

WHAT IS ALREADY KNOWN ON THIS TOPIC

The injury rate among female footballers, regardless of age and level of performance, approaches that of men

The risk of severe knee injuries, such as anterior cruciate ligament injuries, is three to five times higher for female than male football players

Studies from other sports indicate that it might be possible to reduce the rate of lower extremity injuries, but no programmes have been validated for female footballers

WHAT THIS STUDY ADDS

A comprehensive warm-up programme designed to improve strength, awareness, and neuromuscular control can prevent injuries in young female footballers

The risk of injury can be reduced by about one third and the risk of severe injuries by as much as a half

Table 4 | Numbers and severity of injuries in young female footballers according to use of warm-up exercise programme (intervention)

	Intervention (n=1055)	Control (n=837)	Rate ratio (95% CI)*	P value (z test)
All injuries:				
Total	161	215	0.68 (0.56 to 0.84)	0.0003
Match	109	138	0.71 (0.55 to 0.91)	0.007
Training	51	74	0.63 (0.44 to 0.90)	0.012
Minimal injuries (1-3 days)	27	32	0.77 (0.46 to 1.28)	0.313
Mild injuries (4-7 days)	24	34	0.64 (0.38 to 1.08)	0.097
Moderate injuries (8-28 days)	63	70	0.82 (0.58 to 1.15)	0.250
Severe injuries (>28 days)	47	79	0.54 (0.38 to 0.78)	0.0009
Overuse injuries:				
Total	25	52	0.44 (0.27 to 0.71)	0.0007
Minimal injuries	5	10	0.46 (0.16 to 1.33)	0.142
Mild injuries	3	7	0.39 (0.10 to 1.51)	0.174
Moderate injuries	9	11	0.75 (0.31 to 1.80)	0.509
Severe injuries	8	24	0.30 (0.14 to 0.68)	0.004
Acute injuries:				
Total	136	163	0.76 (0.61 to 0.95)	0.017
Minimal injuries	22	22	0.91 (0.50 to 1.64)	0.757
Mild injuries	21	27	0.71 (0.40 to 1.25)	0.234
Moderate injuries	54	59	0.83 (0.58 to 1.21)	0.332
Severe injuries	39	55	0.65 (0.43 to 0.97)	0.037
Contact	53	76	0.64 (0.45 to 0.90)	0.011
Non-contact	55	58	0.86 (0.60 to 1.25)	0.435
Acute knee injuries	27	37	0.66 (0.41 to 1.09)	0.105
Acute ankle injuries	51	52	0.89 (0.61 to 1.31)	0.562

*Rate ratio obtained from Poisson model.

Implications

We used young female footballers (aged 13-17) as a model, and we do not know if the results can be generalised to both sexes, other age groups, or other youth sports. Similar preventive programmes, however, were effective in senior elite football,[28 38] young male footballers,[46] and in both sexes in other sports.[30 31] Furthermore, in youth team handball Olsen et al[27] also documented a substantial decrease in the rate of injuries as a result of a similar structured warm-up programme. Football differs from many other team sports, however, in that there is a much higher potential for direct contact to the lower extremities. Nevertheless, the mechanisms for serious knee injuries seem to be comparable across many sports (mostly non-contact, resulting from pivoting and landing movements). It therefore seems reasonable to assume that the programme we used could be modified for use in other similar sports, at least for some types of injury.

One of the goals in sports injury prevention should be to develop less vulnerable movement patterns. Thus, it might be easier to work with even younger players who have not yet established their basic motion patterns. We therefore suggest that programmes to improve strength, awareness, and neuromuscular control of static and dynamic movements should be implemented as soon as children start playing organised football.

We thank the project assistants (Birgitte Lauersen, Agnethe Nilstad, Ellen Blom, Olav Kristianslund, Tone Wigemyr, Monika Bayer, Heidi M Pedersen, Vegar Vallestad, and John Bjørneboe), the coaches, and the players who participated in this study. A poster illustrating various exercise components and progressions of programme is available at www.ostrc.no/en/Project/144—The-11-plus/. Also, videos displaying every exercise in the programme (with Norwegian text and narrator) are available at www.klokavskade.no/no/Skadefri/Fotball/SPILLEKLAR/.

Contributors: TS, GM, KS, HS, MB, AJ, JD, RB, and TEA contributed to study conception, design, and development of the intervention. TS coordinated the study and managed all aspects of the trial, including data collection. IH conducted and initialised the data analyses, which were planned and checked with TS, RB, and TEA. TS, RB, and TEA wrote the first draft of the paper, and all authors contributed to the final manuscript. TS and TEA are guarantors.
Funding: This study was supported by grants from the FIFA Medical Assessment and Research Centre. The Oslo Sports Trauma Research Center has been established at the Norwegian School of Sport Sciences through grants from the Royal Norwegian Ministry of Culture and Church Affairs, the South-Eastern Norway Regional Health Authority, the Norwegian Olympic Committee and Confederation of Sport, and Norsk Tipping AS. No author or related institution has received any financial benefit from research in this study.
Competing interests: None declared.
Ethical approval: The study was approved by the regional committee for medical research ethics, South-Eastern Norway Regional Health Authority, Norway. Players and parents gave individual written informed consent.
Provenance and peer review: Not commissioned; externally peer reviewed.

1 FIFA. *FIFA big count 2006: 270 million people active in football*. www.fifa.com/aboutfifa/media/newsid=529882.html
2 Engström B, Johansson C, Törnkvist H. Soccer injuries among elite female players. *Am J Sports Med* 1991;19:372-5.
3 Östenberg A, Roos H. Injury risk factors in female European football. A prospective study of 123 players during one season. *Scand J Med Sci Sports* 2000;10:279-85.
4 Söderman K, Adolphson J, Lorentzon R, Alfredson H. Injuries in adolescent female players in European football: a prospective study over one outdoor soccer season. *Scand J Med Sci Sports* 2001;11:299-304.
5 Faude O, Junge A, Kindermann W, Dvorak J. Injuries in female soccer players: a prospective study in the German national league. *Am J Sports Med* 2005;33:1694-700.
6 Giza E, Mithofer K, Farrell L, Zarins B, Gill T. Injuries in women's professional soccer. *Br J Sports Med* 2005;39:212-6.
7 Becker A, Gaulrapp H, Hess H. Injuries in women soccer—results of a prospective study—in cooperation with the German Football Association (DFB). *Sportverletz Sportschaden* 2006;20:196-200.
8 Jacobson I, Tegner Y. Injuries among female football players—with special emphasis on regional differences. *Adv Physioth* 2006;8:66-74.
9 Jacobson I, Tegner Y. Injuries among Swedish female elite football players: a prospective population study. *Scand J Med Sci Sports* 2007;17:84-91.
10 Junge A, Dvorak J. Injuries in female football players in top-level international tournaments. *Br J Sports Med* 2007;41(suppl 1):i3-7.
11 Tegnander A, Olsen OE, Moholdt TT, Engebretsen L, Bahr R. Injuries in Norwegian female elite soccer: a prospective one-season cohort study. *Knee Surg Sports Traumatol Arthrosc* 2008;16:194-8.
12 Steffen K, Andersen TE, Bahr R. Risk of injury on artificial turf and natural grass in young female football players. *Br J Sports Med* 2007;41(suppl 1):i33-7.
13 Bjordal JM, Arnly F, Hannestad B, Strand T. Epidemiology of anterior cruciate ligament injuries in soccer. *Am J Sports Med* 1997;25:341-5.
14 Powell JW, Barber-Foss KD. Sex-related injury patterns among selected high school sports. *Am J Sports Med* 2000;28:385-91.
15 Lohmander LS, Östenberg A, Englund M, Roos H. High prevalence of knee osteoarthritis, pain, and functional limitations in female soccer players twelve years after anterior cruciate ligament injury. *Arthritis Rheum* 2004;50:3145-52.
16 Von Porat A, Roos EM, Roos H. High prevalence of osteoarthritis 14 years after an anterior cruciate ligament tear in male soccer players: a study of radiographic and patient relevant outcomes. *Ann Rheum Dis* 2004;63:269-73.
17 Myklebust G, Bahr R. Return to play guidelines after anterior cruciate ligament surgery. *Br J Sports Med* 2005;39:127-31.
18 Heidt RS Jr, Sweeterman LM, Carlonas RL, Traub JA, Tekulve FX. Avoidance of soccer injuries with preseason conditioning. *Am J Sports Med* 2000;28:659-62.
19 Söderman K, Werner S, Pietila T, Engström B, Alfredson H. Balance board training: prevention of traumatic injuries of the lower extremities in female soccer players? A prospective randomized intervention study. *Knee Surg Sports Traumatol Arthrosc* 2000;8:356-63.
20 Mandelbaum BR, Silvers HJ, Watanabe DS, Knarr JF, Thomas SD, Griffin LY, et al. Effectiveness of a neuromuscular and proprioceptive training program in preventing anterior cruciate ligament injuries in female athletes: 2-year follow-up. *Am J Sports Med* 2005;33:1003-10.
21 Dvorak J, Junge A. *Football medicine manual*. Zurich: F-MARC, 2005:81-93.
22 Steffen K, Myklebust G, Olsen OE, Holme I, Bahr R. Preventing injuries in female youth football—a cluster-randomized controlled trial. *Scand J Med Sci Sports* 2008 Jan 14 [epub ahead of print].
23 Olsen OE, Myklebust G, Engebretsen L, Bahr R. Injury pattern in youth team handball: a comparison of two prospective registration methods. *Scand J Med Sci Sports* 2006;16:426-32.
24 Fuller CW, Ekstrand J, Junge A, Andersen TE, Bahr R, Dvorak J, et al. Consensus statement on injury definitions and data collection procedures in studies of football (soccer) injuries. *Br J Sports Med* 2006;40:193-201.
25 Lin DY, Wei DJ. The robust inference for the Cox proportional hazards model. *J Am Stat Assoc* 1989;84:1074-8.
26 Bahr R, Holme I. Risk factors for sports injuries—a methodological approach. *Br J Sports Med* 2003;37:384-92.
27 Olsen OE, Myklebust G, Engebretsen L, Holme I, Bahr R. Exercises to prevent lower limb injuries in youth sports: cluster randomised controlled trial. *BMJ* 2005;330:449.
28 Caraffa A, Cerulli G, Projetti M, Aisa G, Rizzo A. Prevention of anterior cruciate ligament injuries in soccer. A prospective controlled study of proprioceptive training. *Knee Surg Sports Traumatol Arthrosc* 1996;4:19-21.
29 Myklebust G, Engebretsen L, Brækken IH, Skjølberg A, Olsen OE, Bahr R. Prevention of anterior cruciate ligament injuries in female team handball players: a prospective intervention study over three seasons. *Clin J Sport Med* 2003;13:71-8.
30 Emery CA, Cassidy JD, Klassen TP, Rosychuk RJ, Rowe BH. Effectiveness of a home-based balance-training program in reducing sports-related injuries among healthy adolescents: a cluster randomized controlled trial. *CMAJ* 2005;172:749-54.
31 Hewett TE, Lindenfeld TN, Riccobene JV, Noyes FR. The effect of neuromuscular training on the incidence of knee injury in female athletes. A prospective study. *Am J Sports Med* 1999;27:699-706.
32 Boden BP, Dean GS, Feagin JA Jr, Garrett WE Jr. Mechanisms of anterior cruciate ligament injury. *Orthopedics* 2000;23:573-8.
33 Ebstrup JF, Bojsen-Møller F. Anterior cruciate ligament injury in indoor ball games. *Scand J Med Sci Sports* 2000;10:114-6.
34 Olsen OE, Myklebust G, Engebretsen L, Bahr R. Injury mechanisms for anterior cruciate ligament injuries in team handball: a systematic video analysis. *Am J Sports Med* 2004;32:1002-12.
35 Hewett TE, Myer GD, Ford KR, Heidt RS Jr, Colosimo AJ, McLean SG, et al. Biomechanical measures of neuromuscular control and valgus loading of the knee predict anterior cruciate ligament injury risk in female athletes: a prospective study. *Am J Sports Med* 2005;33:492-501.
36 Krosshaug T, Nakamae A, Boden BP, Engebretsen L, Smith G, Slauterbeck JR, et al. Mechanisms of anterior cruciate ligament injury in basketball: video analysis of 39 cases. *Am J Sports Med* 2007;35:359-67.
37 Mjølsnes R, Arnason A, Østhagen T, Raastad T, Bahr R. A 10-week randomized trial comparing eccentric vs. concentric hamstring strength training in well-trained soccer players. *Scand J Med Sci Sports* 2004;14:311-7.
38 Arnason A, Andersen TE, Holme I, Engebretsen L, Bahr R. Prevention of hamstring strains in elite soccer: an intervention study. *Scand J Med Sci Sports* 2008;18:40-8.
39 Hewett TE, Stroupe AL, Nance TA, Noyes FR. Plyometric training in female athletes. Decreased impact forces and increased hamstring torques. *Am J Sports Med* 1996;24:765-73.
40 Chappell JD, Yu B, Kirkendall DT, Garrett WE. A comparison of knee kinetics between male and female recreational athletes in stop-jump tasks. *Am J Sports Med* 2002;30:261-7.
41 Fagenbaum R, Darling WG. Jump landing strategies in male and female college athletes and the implications of such strategies for anterior cruciate ligament injury. *Am J Sports Med* 2003;31:233-40.
42 Li G, Rudy TW, Sakane M, Kanamori A, Ma CB, Woo SL. The importance of quadriceps and hamstring muscle loading on knee kinematics and in-situ forces in the ACL. *J Biomech* 1999;32:395-400.
43 Beynnon BD, Fleming BC, Johnson RJ, Nichols CE, Renstrom PA, Pope MH. Anterior cruciate ligament strain behavior during rehabilitation exercises in vivo. *Am J Sports Med* 1995;23:24-34.
44 Withrow TJ, Huston LJ, Wojtys EM, Ashton-Miller JA. Effect of varying hamstring tension on anterior cruciate ligament strain during in vitro impulsive knee flexion and compression loading. *J Bone Joint Surg Am* 2008;90:815-23.
45 Bahr R, Lian O, Bahr IA. A twofold reduction in the incidence of acute ankle sprains in volleyball after the introduction of an injury prevention program: a prospective cohort study. *Scand J Med Sci Sports* 1997;7:172-7.
46 Junge A, Rosch D, Peterson L, Graf-Baumann T, Dvorak J. Prevention of soccer injuries: a prospective intervention study in youth amateur players. *Am J Sports Med* 2002;30:652-9.

Accepted: 26 September 2008
