# Supplementary material for: Comparative Effectiveness of Multi-Component, Exercise-Based Interventions for Preventing Soccer-Related Musculoskeletal Injuries: A Systematic Review and Meta-Analysis
Source: Healthcare (Basel). 2025 Mar 29;13(7):765. doi: 10.3390/healthcare13070765 (PMC11988859; doi:10.3390/healthcare13070765)
Supplement: Supplementary file 1 [file healthcare-13-00765-s001.zip › Additional material Included literature/Steffen 2008.pdf]

Scand J Med Sci Sports 2008: 18: 605–614
Printed in Singapore . All rights reserved
DOI: 10.1111/j.1600-0838.2007.00703.x

Copyright © 2007 The Authors
Journal compilation © 2007 Blackwell Munksgaard

SCANDINAVIAN JOURNAL OF
MEDICINE & SCIENCE
IN SPORTS

Preventing injuries in female youth football – a cluster-randomized controlled trial

K. Steffen, G. Myklebust, O. E. Olsen, I. Holme, R. Bahr

Department of Sports Medicine, Oslo Sports Trauma Research Center, Norwegian School of Sport Sciences, Oslo, Norway
Corresponding author: Kathrin Steffen, Department of Sports Medicine, Oslo Sports Trauma Research Center, Norwegian School of Sport Sciences, P.O. Box 4014 Ullevål Stadion, 0806 Oslo, Norway. Fax: 47 23 26 23 07, E-mail: kathrin.-steffen@nih.no

Accepted for publication 18 April 2007

A set of exercises – the "11" – have been selected to prevent football injuries. The purpose of this cluster-randomized controlled trial was to investigate the effect of the "11" on injury risk in female youth football. Teams were randomized to an intervention ($n = 59$ teams, 1091 players) or a control group ($n = 54$ teams, 1001 players). The intervention group was taught the "11," exercises for core stability, lower extremity strength, neuromuscular control and agility, to be used as a 15-min warm-up program for football training over an 8-month season. A total of 396 players (20%) sustained 483 injuries. No difference was observed in the overall injury rate between the intervention (3.6 injuries/1000 h, confidence interval (CI) 3.2–4.1) and control group (3.7, CI 3.2–4.1; RR = 1.0, CI 0.8–1.2; $P = 0.94$) nor in the incidence for any type of injury. During the first 4 months of the season, the training program was used during 60% of the football training sessions, but only 14 out of 58 intervention teams completed more than 20 prevention training sessions. In conclusion, we observed no effect of the injury prevention program on the injury rate, most likely because the compliance with the program was low.

The popularity of female football has increased worldwide during the last decades (Engström et al., 1991; Östenberg & Roos, 2000; Söderman et al., 2001; Emery et al., 2005; Faude et al., 2005), and in 2005 25% of the players in the Norwegian Football Association (NFF) were female (NFF, 2005). The injury incidence among elite and non-elite female players is reported to be similar to male football players, ranging from 12.6 to 24 injuries per 1000 match hours and from 1.2 to 7 per 1000 training hours (Engström et al., 1991; Östenberg & Roos, 2000; Faude et al., 2005; Giza et al., 2005). However, two recent cohort studies have reported somewhat lower figures among adolescent female football players: 8.9–9.1 and 1.5–2.6 per 1000 match and training hours, respectively (Söderman et al., 2001; Emery et al., 2005).

The most common injury locations in female football players are the knee (7–32%), ankle (9–31%) and thigh (6–22%) (Engström et al., 1991; Östenberg & Roos, 2000; Söderman et al., 2001; Junge et al., 2004a; Faude et al., 2005; Giza et al., 2005). The risk for serious knee injuries, such as anterior cruciate ligament (ACL) injuries, is a particular concern in female sports (Myklebust et al., 2003; Faude et al., 2005; Mandelbaum et al., 2005; Olsen et al., 2005; Silvers et al., 2005; Hewett et al., 2006), and after an ACL injury, there is a dramatic increase in the risk of early osteoarthrosis (von Porat et al., 2004; Myklebust & Bahr, 2005). In junior football, the ACL injury rate was reported to be five times higher for girls than for boys (Bjørdal et al., 1997; Powell & Barber-Foss, 2000), Thus, there is clearly a need to develop programs to prevent lower extremity injuries in football and implement these as early as possible. Studies from different sports have shown promising reductions in injury rates using training protocols incorporating one or more exercise component focusing on balance training, strength and/or agility (Caraffa et al., 1996; Heidt et al., 2000; Junge et al., 2002; Askling et al., 2003; Myklebust et al., 2003; Wedderkopp et al., 2003; Mandelbaum et al., 2005; Olsen et al., 2005). However, prospective randomized intervention studies are still needed to investigate the efficacy of training programs aimed to reduce injuries in football, especially among young female players. One such program – the "11" – has been developed recently by an expert group convened by Fédération Internationale de Football Association (FIFA) (F-MARC, 2005). The "11" was developed as a structured warm-up program targeting the most common injury types in football, i.e. ankle and knee sprains, groin and hamstring strains. The program was designed on the basis of previous research on injury

prevention (Junge et al., 2002; Myklebust et al., 2003; Mjølsnes et al., 2004; Mandelbaum et al., 2005; Olsen et al., 2005) and established principles for rehabilitation of such injuries (F-MARC, 2005).

The purpose of this cluster-randomized controlled trial was to examine the effect of the "11," used as a warm-up exercise program to prevent injuries among young female football players.

Material and methods

Study population

All teams in the southeast regions of Norway that registered to participate in the Under-17 league system during the 2005 season were invited to take part in the study. Of 157 available teams, 113 teams (72%) with a total of 2100 players agreed to participate in the investigation (Fig. 1). The competitive season lasted from the end of April until mid-October, interrupted by a 7-week summer break without regular league matches, only invitational tournaments. The teams were also followed for 2 months of the pre-season period (March–April).

After recruitment of all teams into the study, these were block-randomized with four teams in each block into an intervention group and a control group. To reduce potential confounding, the teams were matched by region. All teams from one club were in the same treatment arm, and there were seven clubs included with two teams each. The statistician (I. H.) who conducted the randomization was not involved in the intervention, and recruitment was completed before randomization. The teams in the intervention group were given information about the prevention program, while the teams in the control group were asked to continue their warm-up and training as usual during the season. They were informed that they would receive the same injury prevention program as the intervention group during the subsequent season, provided the program was shown to prevent injuries.

Before the start of the pre-season, the players received written and oral information about the study, and it was emphasized that participation was voluntary. The study was approved by the Regional Committee for Research Ethics, and written consent was obtained. A player was entered in the study if she was registered by the team as participating in the U17 league system, which means that she had to be 16 years or younger. However, teams competing in the U17 league could apply for an exemption to use older players, if they did not have enough eligible players. The players were screened for injuries using a self-constructed questionnaire, and they had to be uninjured at the start of the study to be included.

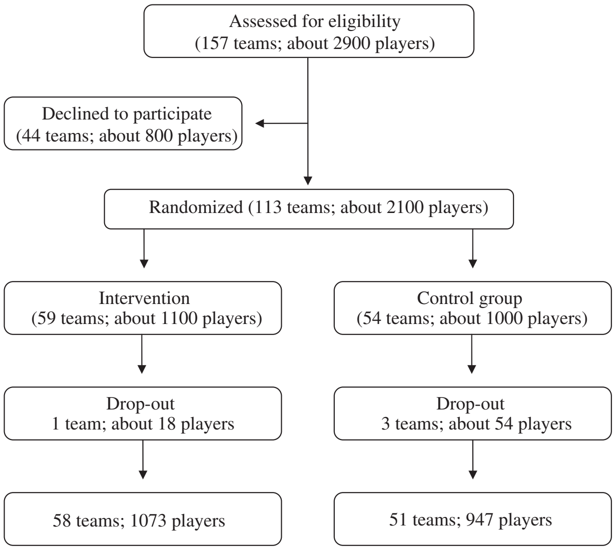

Fig. 1. Flow of teams and players through the study.

The intervention program

The intervention program – the "11" – includes 10 exercises focusing on core stability, balance, dynamic stabilization and eccentric hamstrings strength (F-MARC, 2005) (Table 1). The 11th component, fair play, was not included as part of the program tested in this study.

When introducing the program to the teams, the main focus was on performing the exercises properly. The players were encouraged to concentrate on the quality of their movements, and emphasis was placed on core stability, hip control and proper knee alignment to avoid excessive genu valgus in the static and dynamic balance exercises, as well as in landings from jumps. The coaches and players were instructed to watch each other closely during the training sessions and give continuous feed back.

The teams in the intervention group received 15 balance mats each (40 cm × 50 cm, 7 cm thick; Alusuisse Airex, Sins, Switzerland), which were not part of the original "11," Additionally, all coaches and players received a detailed brochure describing the intervention program, how the exercises should be performed properly, as well as common errors. After familiarization with the exercises, the program was planned to last about 20 min, including 5 min of jogging before starting the exercises. The coaches were asked to use the program every training session for 15 consecutive sessions and thereafter once a week during the rest of the season, replacing any warm-up routine normally used by the team.

The prevention program was introduced to the teams in the intervention group in the beginning of the pre-season, with guidance and surveillance by 26 instructors, mainly from the NFF, each of them responsible for two to three teams. The instructors had been introduced to the intervention program during a seminar, where they received theoretical and practical training in the program and were instructed in how to teach the exercises to the teams. The instructors visited the teams in the intervention group three times during the initial training period, with a booster visit immediately after the 7-week summer break to encourage the teams to continue using the training program. In addition, the first author (K. S.) was in regular contact with the coaches, i.e. by phone/mail and by site visits on the pitch. Implementation of the prevention program in the selected teams by the instructors took about $1\frac{1}{2}$ month.

At the first prevention training session, the coaches in the intervention group received a compliance form to record participation in the prevention program. Detailed information was requested on the duration of each session in minutes, and the number of attending players.

Injury and exposure registration

To monitor injuries and playing exposure, 18 physical therapists were recruited as injury recorders and assigned to the teams (typically five to seven teams each) during the period from March 1, through October 31, 2005. A seminar had been held for injury recorders to introduce them to the study aims and the injury registration system. All injuries were recorded that occurred after the first prevention training session had been completed by an intervention team, and from the same

Table 1. Exercises and repetitions of the "11" used as a structured warm-up program (F-MARC, 2005)

Exercises	Description	Repetitions
Core stability		
The bench (1)	Leaning on your elbows in the prone position, lift the upper body, hips and knees so that the body forms a straight line from the shoulder to the heels. Hold this position	15 s × 4 repetitions
Sideways bench (2)	Leaning on one elbow in the side position, lift top leg and hips until the shoulder, hip and top leg are in straight line and parallel to the ground. Hold this position	15 s × 2 repetitions on each side
Balance		
Cross-country skiing (3)	In single-leg stance, continuously bend and extend the knee of the supporting leg and swing the arms in rhythm	15 s × 2 repetitions on each leg
Chest pass in single-leg stance (4)	Partner exercise with both players in single-leg stance. Throw a ball back and forth	15 s × 3 repetitions on each leg
Forward bend in single-leg stance (5)	As (4). Before throwing back, touch the ball to the ground without putting weight on it	15 s × 3 repetitions on each leg
Figure-of-eights in single-leg stance (6)	As (4). Before throwing back, move the ball in a figure-eight through and around both legs	15 s × 3 repetitions on each leg
Plyometrics		
Line jumps (sideways, forwards-backwards) (7)	Two-leg jumps sideways over a line and forward-back as quickly as possible	15 jumps of each type
Zigzag shuffle (forwards and backwards) (8)	Shuffle sideways with a low center of mass to the first cone, turn so that the other shoulder points to the next cone and complete the zigzag course as fast as possible	2 repetitions in each direction (20 m)
Bounding (9)	Spring as high and far as possible off the supporting leg. Bring the knee of the trailing leg up as high as possible and the opposite arm in front of the body. Continuous bounding, switching legs on each take off	10–15 jumps × 3 repetitions (20 m)
Strength		
Nordic hamstrings (10)	Lower legs are held stable by a partner. Slowly lean forward keeping the upper body and hips straight while resisting the forward-falling motion by the hamstring muscles	5 repetitions

The single-leg balance exercises (4, 5 and 6) were done on a balance mat when the players were able to perform these exercises properly on stable ground.

date a team in the control group that was randomized to the same block. The coaches of each team were contacted by telephone and/or e-mail at least once a month to record all training and match activity, in addition to new injuries. Injured players were interviewed by the injury recorders to assess aspects of the injury based on a standardized injury questionnaire. The injury recorders were blinded to which group the teams and injured players belonged to. The information was registered using a web-based recording system.

In accordance with the consensus statement on injury definitions and data collection procedures (Fuller et al., 2006), an injury was registered if it caused the player to be unable to fully take part in the next match or training session ("time loss" injury). Acute injuries were defined as injuries with a sudden onset associated with a known trauma, whereas overuse injuries were those with a gradual onset without any known trauma. Recurrent injuries were defined as an injury of the same type and the same site as an index injury and that occurred after a player's return to full participation from the index injury. In addition, the injury circumstances (contact vs non-contact), the location and the type of injury were recorded. Injuries were classified into three severity categories according to the length of absence from training sessions and matches until the player was fully fit to take part in all types of organized football play as follows: minor (1–7 days), moderate (8–21 days) and major (>21 days) (van Mechelen et al., 1992). In almost all cases of moderate and major injuries, the players were seen in a medical center to diagnose the injury by clinical tests, imaging studies or surgery. In cases of minor injuries, the players were examined by a local physical therapist, the coach or not at all. None of the injured players was examined or treated by any of the authors or injury recorders involved in the study.

Data on match and training exposure were collected on a team basis. The overall match and training injury incidence was calculated as the number of injuries per 1000 player hours. To calculate match exposure, match playing time (in minutes) was multiplied by 11 and for training exposure, training time (in minutes) was multiplied by the monthly player attendance. A regular league match was played for 2 × 40 min, while a training session in most cases lasted for 90 min.

Additionally, information on any preventive training used in the control group, including their type and frequency, was obtained at the end of the season.

Outcome measures

The primary outcome variable was to compare the overall injury rate of the intervention with the control groups, after the first prevention training session in the intervention teams had been completed. Secondary effect variables were the

proportion of injured players, the incidences of ankle, knee, groin, hamstrings and other injuries in both groups during the same study period.

Sample size and statistics

Because data on injury incidence in female youth team sports are limited, the sample size was based on data from Swedish female youth football (Söderman et al., 2001) and Danish and Norwegian youth team handball (Wedderkopp et al., 2003; Olsen et al., 2006), which was more assumed to be similar to youth football. From these studies, we estimated that 12% of the players would injure their knee or ankle during one season.

Similar to Olsen et al. (2005), we used one-way analysis of variance to estimate the intracluster correlation coefficient (ICC) to obtain estimates of the inflation factor for comparison with planned sample size. An ICC of 0.05 will give us an estimated inflation factor for cluster effects of 1.8. Owing to randomization by teams, a total of 900 players in each group would provide a power of 0.73 at the 5% significant level to detect a 40% reduction in the number of players with a knee or ankle injury. Our model was based on 120 teams with 18 players per team and a drop-out rate of 15%, i.e. a total of 1800 players.

The proportional difference in the frequency of injured players in the intervention group and the control group was analyzed by a χ^2-test. We used a Z-test and found 95% confidence intervals (CI) based on the Poisson model to compare the rate ratio (RR) of number of injuries between these two groups.

In addition to the intention-to-treat analysis, data are shown separately for teams in the intervention group who better complied with the program and teams who did less (subgroup analysis). Teams were considered to be more compliant if they carried out the prevention program for at least 20 sessions from their first instruction day through June. Results are presented as means with a 95% CI, unless otherwise noted. All tests were two tailed, and results significant with P-values below 0.05.

Results

Inclusion of teams and players

Figure 1 shows the flow of teams and players through the study. After the randomization had been completed, four teams declined to participate in the trial: one in the intervention group (did not have a coach) and three in the control group (withdrew from participation in the league system). The players ($n = 72$) in these teams were excluded from the study. The remaining players in the two groups were similar in their age [15.4 ± 0.8 years (SD) in both groups], age distribution and drop-out rates. Thus, the final sample consisted of 58 teams (1073 players) in the intervention group and 54 teams (947 players) in the control group (Table 2).

Table 2. Characteristics of participants

	Control ($n = 947$ players)	Intervention ($n = 1073$ players)
Age (years)		
13	11 (1.2)	5 (0.5)
14	77 (8.1)	127 (11.8)
15	425 (44.9)	462 (43.0)
16	377 (39.8)	432 (40.3)
17	57 (6.0)	47 (4.4)
Drop-outs	21 (2.2)	27 (2.5)

Values are numbers (%) of participants.

Compliance with the prevention program

The 58 teams in the intervention group performed the injury prevention program a total of 23 ± 9 (SD) times (range 2–42) during the course of the season. In comparison, the teams completed a total of 44 ± 16 sessions of football training (19–90) during the same period. In other words, the injury prevention program was used at 52% of all training sessions (10–100). During the first half of the season (from March through June), the injury prevention program was used in 14 ± 5 sessions (2–25) and after the summer break (from July through October) in 9 ± 5 sessions (0–21). This represents 60% (15–100) of the total number of training sessions before and 44% (0–100) of the training sessions after the summer break. For the prevention training sessions, the average player attendance was 67 ± 10% (51–91). The average time spent per session was 20 ± 4 min (12–32).

Two teams in the intervention group withdrew from the study during the summer break.

None of the control group teams reported to have performed structured exercises comparable with the prevention program throughout the season.

Injury characteristics

During the 8-month season, including the 2-month pre-season and the summer break, 396 (20%) of 2020 players sustained at least one injury. Of these players, 57 (3%) incurred two injuries and 15 (1%) three injuries, leading to a total of 483 injuries. Of these 483 injuries, 98 (20%) were re-injuries, and nine injuries (1.9%) were recurrences of previous injuries during the same season. The proportion of re-injured players in the intervention and control groups was 16% ($n = 32$ out of 204) vs 22% ($n = 43$ out of 192), respectively ($P = 0.22$). Most injuries were acute ($n = 421$, 87%) and most were located in the lower extremities ($n = 413$, 86%). The most common overuse injuries ($n = 62$, 13%) were anterior lower leg pain (29% of all overuse injuries) and knee pain (19%). With a proportion of 28%, an ankle sprain was the most common acute injury type.

In 42% of the 421 acute injury cases, the injury occurred in a non-contact playing situation, while 58% resulted from player-to-player contact. The proportion of contact injuries was higher during matches (86%, $n = 209$) than training (14%, $n = 33$) ($P < 0.001$).

Effect of the prevention program

The total exposure, as well as the match and training exposure, were similar for both groups (Table 3). No difference was observed in the proportion of injured players between the intervention group (19.0%, $n = 204$) and the control group (20.3%, $n = 192$) ($P = 0.50$). The mean age of injured players was 15.4 ± 0.8 years in the intervention and the control group, the same as in the total study population.

The intention-to-treat analysis revealed no difference in the overall injury incidence, nor in the acute match or training incidence between the intervention group and the control group (Table 4). The RR for the intervention vs the control group was 1.0 (CI 0.8–1.2, $P = 0.94$) for all injuries, 1.1 (0.9–1.3, $P = 0.54$) for acute match injuries and 0.7 (0.5–1.1, $P = 0.12$) for acute training injuries.

There were no significant differences between the groups in the distribution of type, location or severity of injuries. During the 8-month study period, nine ACL injuries occurred (0.07 injuries/1000 h, 95% CI 0.02–0.11), four in the intervention group and five in the control group (RR 0.8, 0.2–2.9, $P = 0.73$). An ankle sprain was the most common re-injury type in both groups. However, there was no significant difference between the groups in the number of re-injuries, including the number of recurrent ankle sprains. The RR for the intervention vs the control group for re-injuries was 0.9 (0.6–1.4, $P = 0.79$) and 1.0 (0.5–1.7, $P = 0.86$) for ankle sprain re-injuries.

In a sub-group analysis to determine whether compliance with the intervention program could have influenced the risk for injuries throughout the study period, the intervention group was divided into two sub-groups: those who performed at least 20 prevention training sessions (compliant), and those who completed <20 sessions (non-compliant). However, the analysis revealed no difference in the injury incidence of overall and acute injuries between these two sub-groups, or between the compliant sub-group and the control group (Table 4).

Discussion

The main finding of this investigation on young female football players was that we could not detect any differences in injury rates between teams in the intervention group, who were asked to use a structured warm-up program, and teams in the control group, who were told to warm up as usual with jogging and ball-based exercises. The most likely explanation is that the compliance of the teams and players in the intervention group was insufficient to produce the necessary training effects to reduce injury risk. The average intervention team only participated in about 15 prevention training sessions during the first half of the season, which includes the pre-season and the first half of the competitive season.

Table 3. Exposure data (hours) for the teams

No of teams	Control ($n = 947$ players)	Intervention ($n = 1073$ players)			Total ($n = 2020$ players)
	51	All	Compliant	Non-compliant	109
		58	14	44	
Match	19 856	20 731	5371	15 360	40 587
Training	45 869	45 692	13 722	31 970	91 561
Total	65 725	66 423	19 093	47 330	132 148

The present findings cannot directly be compared with other injury prevention studies from football, which have shown (Caraffa et al., 1996; Heidt et al., 2000; Junge et al., 2002; Mandelbaum et al., 2005) or failed to detect (Söderman et al., 2000) a reduction in injury rates. However, these studies differ in several aspects from the present study. Some used ACL injuries alone as the outcome of interest (Caraffa et al., 1996; Mandelbaum et al., 2005), and some used all-male (Caraffa et al., 1996; Junge et al., 2002) or professional players (Caraffa et al., 1996; Söderman et al., 2000) as their study population.

Compliance with the prevention program

Considerable efforts were made to motivate the intervention teams to include the exercise program as a standard part of their training program. Instructors visited the teams three times during training at the start of the study and again after the summer break, and the teams received balance mats and a brochure detailing the intervention program. Despite this, the intervention teams included the injury prevention program in only 60% of their training sessions during the first half of the season, and only about one quarter of the teams had completed more than 20 prevention training sessions before the summer break. In addition, only two-thirds of the players on these teams participated in these sessions on the average, which means that the average player only completed approximately 15 prevention training sessions. As shown by the sub-group analysis, this amount of prevention training seems to be ineffective in reducing injury risk among young female football players.

The compliance was considerably lower than that reported by Olsen et al. (2005) in their study on youth team handball, where a similar exercise program developed for youth team handball led to an impressive reduction in lower extremity injury risk. In a non-randomized study in senior elite team

Table 4. Number and incidence of injuries in various categories

	Intention-to-treat analysis					Sub-group analysis					
	Control ($n = 51$ teams)		Intervention ($n = 58$ teams)			Compliant ($n = 14$ teams)			Non-compliant ($n = 44$ teams)		
	Injuries	Incidence	Injuries	Incidence	Rate ratio (INT vs CON)	Injuries	Incidence	Rate ratio (COM vs CON)	Injuries	Incidence	Rate ratio (COM vs N-COM)
All injuries	241	3.7 (3.2–4.1)	242	3.6 (3.2–4.1)	1.0 (0.8–1.2)	64	3.4 (2.5–4.2)	0.9 (0.7–1.2)	178	3.8 (3.2–4.3)	0.9 (0.7–1.2)
Overuse	31	0.5 (0.3–0.6)	31	0.5 (0.3–0.6)	0.9 (0.6–1.7)	11	0.6 (0.2–0.9)	1.2 (0.6–2.4)	20	0.4 (0.2–0.6)	1.4 (0.7–2.8)
All acute injuries	210	3.2 (2.8–3.6)	211	3.2 (2.7–3.6)	1.0 (0.8–1.2)	53	2.8 (2.0–3.5)	0.9 (0.6–1.2)	158	3.3 (2.8–3.9)	0.8 (0.6–1.1)
Match	151	7.6 (6.4–8.8)	169	8.2 (6.9–9.4)	1.1 (0.9–1.3)	42	7.8 (5.5–10.2)	1.0 (0.7–1.4)	127	8.3 (6.8–9.7)	0.9 (0.7–1.3)
Training	59	1.3 (1.0–1.6)	42	0.9 (0.6–1.2)	0.7 (0.5–1.1)	11	0.8 (0.3–1.3)	0.6 (0.3–1.2)	31	1.0 (0.6–1.3)	0.8 (0.4–1.6)
Body location											
Upper body	37	0.6 (0.4–0.7)	30	0.5 (0.3–0.6)	0.8 (0.5–1.3)	4	0.1 (0–0.2)	–*	26	0.5 (0.3–0.8)	–*
Lower body	173	2.6 (2.2–3.0)	181	2.7 (2.3–3.1)	1.0 (0.8–1.3)	49	2.6 (1.8–3.3)	1.0 (0.7–1.3)	132	2.8 (2.3–3.3)	0.9 (0.7–1.3)
Groin	14	0.2 (0.1–0.3)	6	0.1 (0–0.2)	0.4 (0.2–1.1)	0	–*	–*	6	0.1 (0–0.2)	–*
Thigh	28	0.4 (0.3–0.6)	35	0.5 (0.4–0.7)	1.2 (0.8–2.0)	13	0.7 (0.3–1.1)	1.6 (0.8–3.1)	23	0.5 (0.3–0.7)	1.4 (0.7–2.8)
Knee	30	0.5 (0.3–0.6)	37	0.6 (0.4–0.7)	1.2 (0.8–2.0)	5	0.3 (0–0.5)	0.6 (0.2–1.5)	32	0.7 (0.4–0.9)	0.4 (0.2–1.0)
Ankle	74	1.1 (0.9–1.4)	79	1.2 (0.9–1.5)	1.1 (0.8–1.5)	24	1.3 (0.8–1.8)	1.1 (0.7–1.7)	55	1.2 (0.9–1.5)	1.1 (0.7–1.7)
Injury type											
Contusion	54	0.8 (0.6–1.0)	54	0.8 (0.6–1.0)	1.0 (0.7–1.4)	15	0.8 (0.4–1.2)	1.0 (0.5–1.7)	39	0.8 (0.6–1.1)	1.0 (0.5–1.7)
Sprain	84	1.3 (1.0–1.6)	89	1.3 (1.1–1.6)	1.1 (0.8–1.4)	22	1.2 (0.7–1.6)	0.9 (0.6–1.4)	67	1.4 (1.1–1.8)	0.8 (0.5–1.3)
Strain	42	0.6 (0.4–0.8)	45	0.7 (0.5–0.9)	1.1 (0.7–1.6)	14	0.7 (0.3–1.1)	1.1 (0.6–2.1)	31	0.7 (0.4–0.9)	1.1 (0.6–2.1)
Other	29	0.4 (0.3–0.6)	23	0.4 (0.2–0.5)	0.8 (0.5–1.4)	2	0.1 (0–0.1)	–*	21	0.4 (0.3–0.6)	–*
Re-injuries	43	0.7 (0.5–0.8)	41	0.6 (0.4–0.8)	0.9 (0.6–1.4)	9	0.5 (0.2–0.8)	0.7 (0.4–1.5)	32	0.7 (0.4–0.9)	0.7 (0.3–1.5)
Ankle sprains	23	0.3 (0.2–0.5)	22	0.3 (0.2–0.5)	1.0 (0.5–1.7)	6	0.3 (0.1–0.6)	0.9 (0.4–2.2)	16	0.3 (0.2–0.5)	0.9 (0.4–2.4)
Contact	124	1.9 (1.6–2.2)	118	1.8 (1.5–2.1)	0.9 (0.7–1.2)	33	1.7 (1.1–2.3)	0.9 (0.6–1.3)	85	1.8 (1.4–2.2)	1.0 (0.6–1.4)
Non-contact	86	1.3 (1.0–1.5)	93	1.4 (1.1–1.7)	1.1 (0.8–1.5)	20	1.0 (0.6–1.5)	0.8 (0.5–1.3)	73	1.5 (1.2–1.9)	0.7 (0.4–1.1)
Time loss (days)											
1–7	82	1.3 (1.0–1.5)	102	1.5 (1.2–1.8)	1.2 (0.9–1.6)	28	1.5 (0.9–2.0)	1.2 (0.8–1.8)	74	1.6 (1.2–1.9)	0.9 (0.6–1.4)
8–21	69	1.1 (0.8–1.3)	61	0.9 (0.7–1.1)	0.9 (0.6–1.2)	15	0.8 (0.4–1.2)	0.7 (0.4–1.3)	46	1.0 (0.7–1.3)	0.8 (0.5–1.4)
> 21	59	0.9 (0.7–1.1)	48	0.7 (0.5–0.9)	0.8 (0.6–1.2)	10	0.5 (0.2–0.8)	0.6 (0.3–1.4)	38	0.8 (0.5–1.1)	0.7 (0.3–1.3)

*Owing to small numbers statistics were not computed.

The incidence is reported per 1000 h of exposure with 95% CI. Rate ratios are shown with 95% CI for intention-to-treat analyses between control (CON) and intervention (INT) teams, and for sub-group analyses between compliant (COM) teams and control teams, as well as between compliant and non-compliant (N-COM) teams.

CI, confidence interval.

handball, which examined the effect of a program developed to prevent ACL injuries, Myklebust et al. (2003) also observed poor compliance, as low as 28%. Similar to the present study, their study did not show any overall effect on injury rate, but in contrast to the present study a sub-group analysis revealed that the injury risk was significantly lower in players who had followed the prevention program. With the exception of these three studies, other intervention studies (Caraffa et al., 1996; Heidt et al., 2000; Junge et al., 2002; Askling et al., 2003; Mandelbaum et al., 2005) have not reported individual or team compliance in a way that makes it possible to compare between studies.

When attempting wide-scale implementation of preventive programs, it may be difficult to know how likely it is that the intervention will be adopted (Finch, 2006). The low compliance observed in the current study probably results from a number of factors. The pre-season preparation period for these teams was relatively short, as was the competitive season. Also, during midseason there was a 7-week summer break (school holidays), when few of the teams had organized training, but participated in tournaments. Also, matches were generally scheduled on weekdays, which further limited the time available for training, because the exercise program was generally not used to warm up for games. In the youth team handball study (Olsen et al., 2005), teams played their matches during the weekend, played a longer winter season with only a 2-week Christmas break midseason and used the injury prevention exercises up to three times a week during the season (Olsen OE, personal communication, 2006). In contrast, during the competitive season, many of the teams in the present investigation often trained only once or twice weekly. As a consequence of these factors, the ability to include preventive training sessions on a consistent basis may have been limited. It is possible that program implementation would be easier in teams who train more intensively, with more than three training sessions each week.

Previous authors have emphasized the importance of being focused and providing continuous feedback when training neuromuscular control (Myklebust et al., 2003; Hewett et al., 2005; Olsen et al., 2005), because balance exercises should be performed properly with a stable core and focus on the "knee-over-toe position" to achieve the desired training effects. Söderman et al. (2000) showed that home-based balance training did not have any effect on injury rates in female football players. In contrast, in the study by Myklebust et al. (2003) physical therapists were recruited to improve compliance and training quality by supervising training sessions regularly. Unfortunately, although proper skills and feedback by coaches and team mates were emphasized by the instructors during the implementation and follow-up visits, no quantifiable information could be obtained on the quality of exercise performance during the prevention sessions.

Contamination from the intervention to the control groups is a relevant issue in intervention trials such as this. However, our results show that there was no contamination in the control group. Instead, the challenge was in motivating the intervention teams to do the exercises as prescribed. At the end of the season, we made phone calls to all the coaches in the control group to ask them about their warm-up exercises and training routines. None of these teams performed structured exercises similar to the "11," and contamination is therefore not thought to have biased our results.

Content and structure of the prevention program

The exercises comprising the "11" represent evidence-based rehabilitation exercises for lower limb injuries (F-MARC, 2005) and key exercises from other effective injury prevention programs. However, in contrast to other prevention programs, the present exercise prescription did not provide the possibility for variation and progression. The ACL injury prevention program of Caraffa et al. (1996) and Myklebust et al. (2003) included a five-step progression from simple to more challenging within each of the balance and jump-landing exercises used. The lower limb injury prevention program of Olsen et al. (2005) consisted of four groups of varied exercises with progression guidelines within each category, structured jogging, technique, neuromuscular and strength training. Also, both the Myklebust program and the Olsen program included exercises where the athlete was perturbed during single-leg balance training, representing an additional challenge to the ability to maintain a stable core and proper alignment. In elite male football, hamstrings strength was trained three times weekly with a gradually increasing intensity over a 10-week period to increase strength successfully (Askling et al., 2003; Mjølsnes et al., 2004) and reduce strain injuries (Askling et al., 2003; Árnason et al., 2007). Thus, the current program contrasts with those used in these studies in that all of the 10 exercises were to be carried out during every 15-min training session, generally without progression or variation. This may have resulted in reduced motivation among coaches and players. Because training volume and intensity are key determinants for training outcome, the effectiveness of the current program might be improved by fewer exercises with more repetitions each training session to allow progression and higher intensities. In a separate study, we could not detect any effect on a range of performance tests in a group of adolescent female

football players who used the "11" as a structured warm-up program for a 10-week period (Steffen et al., 2007).

A final issue is whether the exercises used were appropriate. In both of the team handball injury prevention programs (Myklebust et al., 2003; Olsen et al., 2005), focus was placed on technique training in high-risk situations identified from video analysis of ACL injuries (Olsen et al., 2004). Although it may be reasonable to assume that female football players would also benefit from not allowing the knee to pivot medially during cut and plant movements and after landings, there is no direct evidence identifying the injury mechanisms in female youth football. It may be that, unlike team handball, knee and ankle injuries in football to a greater extent result from direct contact with the lower extremity, with less potential for intervention through balance or fitness training. More dynamic exercises resembling football play and injury-risk situations, like running with rapid changes of direction, dribbling, landing after heading and perturbations (Giza et al., 2003; Andersen et al., 2004; Árnason et al., 2004), may be some ways to adjust to the program.

Methodological issues

Lack of randomization (Caraffa et al., 1996; Heidt et al., 2000; Junge et al., 2002; Mandelbaum et al., 2005), low study power (Caraffa et al., 1996; Heidt et al., 2000; Söderman et al., 2000; Junge et al., 2002) and a high drop-out rate (Söderman et al., 2000; Junge et al., 2002) are some of the limitations in former investigations on injury prevention in football. The main strength of the present investigation is its design as a randomized-controlled trial with a large sample size. The intraclass correlation coefficient, which was calculated to 0.06, gave us an inflation factor of 1.8. However, even if the sample size estimate was accurate, study power was still limited. The final sample of 2020 players provided a statistical power of 0.76 to detect a group difference of 40% in the number of players with a knee or ankle injury. However, this means that we cannot rule out that there may have been beneficial effects on specific injury types. For example, on comparing the subgroup of teams who completed more than 20 training sessions with the control group, in absolute numbers the rate of severe injuries was 42% lower, the rate of moderate injuries 25% lower and the rate of re-injuries was 28% lower within the intervention teams than the control teams (see Table 4). However, the number of injuries in each of these groups is limited and the effect of the program on specific injury types therefore cannot be assessed reliably.

The same registration method as in the present study was successfully used by Olsen et al. (2005). The reliability and validity of the exposure and injury registration have been discussed in detail previously (Olsen et al., 2006), and found to be adequate. The proportion of injured players (20%) during one competitive season was considerably lower than reported from a similar cohort of young female Swedish players (41%) (Söderman et al., 2001), but their incidences of acute match (9.1 injuries per 1000 h) and training injuries (1.5 per 1000 h) were similar to the present injury rates. A higher playing exposure among the Swedish players explains the higher proportion of injured players. Therefore, the present injury rates are comparable with previous studies in female youth football (Söderman et al., 2001; Emery et al., 2005). Moreover, if injuries were missed, there is no reason to expect a difference between the intervention and control groups. Nevertheless, one limitation of the present registration method is that, in contrast to Söderman et al. (2000) and Myklebust et al. (2003), individual exposure data were not recorded, which means that it was not possible to perform a sub-group analysis on the individual level.

Perspectives

The benefits of prevention training on injury risk among young female football players have been documented in two trials (Heidt et al., 2000; Mandelbaum et al., 2005). Low compliance among the players, as seen in the current study, is a limitation, and a total of 15 prevention training sessions completed over a 3-month period are insufficient to reduce injury rates. Similar to what has been seen in young Swedish female players (Söderman et al., 2001), the injury pattern among young female players seems to differ from male professional (Hägglund et al., 2005; Waldén et al., 2005) and youth football (Junge et al., 2004b), where groin and hamstring injuries represent as much of a problem as knee and ankle sprains. Among female football players, knee and ankle injuries predominate (Heidt et al., 2000; Östenberg & Roos, 2000; Söderman et al., 2000, 2001; Faude et al., 2005; Giza et al., 2005; Mandelbaum et al., 2005). It therefore seems reasonable to suggest that injury prevention programs for this target group should emphasize lower extremity neuromuscular control, strength and balance training (Hewett et al., 2006). Based on previous research (Hewett et al., 1999; Heidt et al., 2000; Myklebust et al., 2003; Olsen et al., 2005), it appears that such prevention programs should include at least 15 training sessions during the first 6–8 weeks of training. However, further research is needed on how to develop and implement such programs to be as effective as possible in this age and gender group.

Conclusion

In conclusion, we observed no effect of the injury prevention program on the injury rate, most likely because the compliance with the program was low.

Key words: injuries, prevention, neuromuscular training, strength, compliance, soccer.

Acknowledgements

This study was supported by a grant from FIFA. In addition, financial support came from the Oslo Sports Trauma Research Center, which has been established at the Norwegian School of Sport Sciences through grants from the Eastern Norway Regional Health Authority, the Royal Norwegian Ministry of Culture and Church Affaires, the Norwegian Olympic Committee and Confederation of Sports and Norsk Tipping AS. The authors thank the instructors supporting the implementation of the "11," the physical therapists involved in the injury registration and the coaches and players for their cooperation.

References

Andersen TE, Floerenes TW, Árnason A, Bahr R. Video analysis of the mechanisms for ankle injuries in football. Am J Sports Med 2004: 32: 69S–79S.

Árnason A, Andersen TE, Holme I, Engebretsen L, Bahr R. Prevention of hamstring strains in elite soccer: an intervention study. Scand J Med Sci Sports. Published online on March 12, 2007, doi: 10.1111/j.1600-0838.2006.00634.x.

Árnason A, Tenga A, Engebretsen L, Bahr R. A prospective video-based analysis of injury situations in elite male football: football incident analysis. Am J Sports Med 2004: 32: 1459–1465.

Askling C, Karlsson J, Thorstensson A. Hamstring injury occurrence in elite soccer players after preseason strength training with eccentric overload. Scand J Med Sci Sports 2003: 13: 244–250.

Bjørdal JM, Arnly F, Hannestad B, Strand T. Epidemiology of anterior cruciate ligament injuries in soccer. Am J Sports Med 1997: 25: 341–345.

Caraffa A, Cerulli G, Projetti M, Aisa G, Rizzo A. Prevention of anterior cruciate ligament injuries in soccer. A prospective controlled study of proprioceptive training. Knee Surg Sports Traumatol Arthrosc 1996: 4: 19–21.

Emery CA, Meeuwisse WH, Hartmann SE. Evaluation of risk factors for injury in adolescent soccer: implementation and validation of an injury surveillance system. Am J Sports Med 2005: 33: 1882–1891.

Engström B, Johansson C, Törnkvist H. Soccer injuries among elite female players. Am J Sports Med 1991: 19: 372–375.

Faude O, Junge A, Kindermann W, Dvorak J. Injuries in female soccer players: a prospective study in the German national league. Am J Sports Med 2005: 33: 1694–1700.

Finch C. A new framework for research leading to sports injury prevention. J Sci Med Sport 2006: 9: 3–9.

F-MARC. Football medicine Manual. Zurich: F-MARC, 2005: 81–93.

Fuller CW, Ekstrand J, Junge A, Andersen TE, Bahr R, Dvorak J, Hägglund M, McCrory P, Meeuwisse WH. Consensus statement on injury definitions and data collection procedures in studies of football (soccer) injuries. Br J Sports Med 2006: 40: 193–201.

Giza E, Fuller C, Junge A, Dvorak J. Mechanisms of foot and ankle injuries in soccer. Am J Sports Med 2003: 31: 550–554.

Giza E, Mithofer K, Farrell L, Zarins B, Gill T. Injuries in women's professional soccer. Br J Sports Med 2005: 39: 212–216.

Hägglund M, Waldén M, Ekstrand J. Injury incidence and distribution in elite football-a prospective study of the Danish and the Swedish top divisions. Scand J Med Sci Sports 2005: 15: 21–28.

Heidt RS, Sweeterman LM, Carlonas RL, Traub JA, Tekulve FX. Avoidance of soccer injuries with preseason conditioning. Am J Sports Med 2000: 28: 659–662.

Hewett TE, Ford KR, Myer GD. Anterior cruciate ligament injuries in female athletes: part 2, a meta-analysis of neuromuscular interventions aimed at injury prevention. Am J Sports Med 2006: 34: 490–498.

Hewett TE, Lindenfeld TN, Riccobene JV, Noyes FR. The effect of neuromuscular training on the incidence of knee injury in female athletes. Am J Sports Med 1999: 27: 699–706.

Hewett TE, Myer GD, Ford KR. Reducing knee and anterior cruciate ligament injuries among female athletes: a systematic review of neuromuscular training interventions. J Knee Surg 2005: 18: 82–88.

Junge A, Cheung K, Edwards T, Dvorak J. Injuries in youth amateur soccer and rugby players-comparison of incidence and characteristics. Br J Sports Med 2004b: 38: 168–172.

Junge A, Dvorak J, Graf-Baumann T, Peterson L. Football injuries during FIFA tournaments and the Olympic Games, 1998–2001: development and implementation of an injury-reporting system. Am J Sports Med 2004a: 32: 80S–89S.

Junge A, Rösch D, Peterson L, Graf-Baumann T, Dvorak J. Prevention of soccer injuries: a prospective intervention study in youth amateur players. Am J Sports Med 2002: 30: 652–659.

Mandelbaum BR, Silvers HJ, Watanabe DS, Knarr JF, Thomas SD, Griffin LY, Kirkendall DT, Garett W, Jr. Effectiveness of a neuromuscular and proprioceptive training program in preventing anterior cruciate ligament injuries in female athletes: 2-year follow-up. Am J Sports Med 2005: 33: 1003–1010.

Mjølsnes R, Árnason A, Østhagen T, Raastad T, Bahr R. A 10-week randomized trial comparing eccentric vs. concentric hamstrings strength training in well-trained soccer players. Scand J Med Sci Sports 2004: 14: 1–7.

Myklebust G, Bahr R. Return to play guidelines after anterior cruciate ligament surgery. Br J Sports Med 2005: 39: 127–131.

Myklebust G, Engebretsen L, Braekken I, Skjølberg A, Olsen O, Bahr R. Prevention of anterior cruciate ligament injuries in female team handball players: a prospective intervention study over three seasons. Clin J Sports Med 2003: 13: 71–78.

Norwegian Football Association (NFF). http://www.fotball.no/t2.aspx?p = 749&x = 1&a = 123438 (accessed January 31, 2005)

Olsen OE, Myklebust G, Engebretsen L, Bahr R. Injury mechanisms for

anterior cruciate ligament injuries in team handball: a systematic video analysis. Am J Sports Med 2004: 32: 1002–1012.
Olsen OE, Myklebust G, Engebretsen L, Bahr R. Injury pattern in youth team handball: a comparison of two prospective registration methods. Scand J Med Sci Sports 2006: 16: 426–432.
Olsen OE, Myklebust G, Engebretsen L, Holme I, Bahr R. Exercises to prevent lower limb injuries in youth sports: cluster randomised controlled trial. Br Med J 2005: 330: 449–452.
Östenberg A, Roos H. Injury risk factors in female European football. A prospective study of 123 players during one season. Scand J Med Sci Sports 2000: 10: 279–285.
Powell JW, Barber-Foss KD. Sex-related injury patterns among selected high school sports. Am J Sports Med 2000: 28: 385–391.
Silvers HJ, Giza E, Mandelbaum BR. Anterior cruciate ligament tear prevention in the female athlete. Curr Sports Med Rep 2005: 4: 341–343.
Söderman K, Adolphson J, Lorentzon R, Alfredson H. Injuries in adolescent female players in European football: a prospective study over one outdoor soccer season. Scand J Med Sci Sports 2001: 11: 299–304.
Söderman K, Werner S, Pietila T, Engström B, Alfredson H. Balance board training: prevention of traumatic injuries of the lower extremities in female soccer players? A prospective randomized intervention study. Knee Surg Sports Traumatol Arthrosc 2000: 8: 356–363.
Steffen K, Bakka HM, Myklebust G, Bahr R. Performance aspects of an injury prevention program: a 10-week intervention in adolescent female football players. Scand J Med Sci Published online on May 10, 2007, doi: 10.1111/j.1600-0838.2007.00708.x.
van Mechelen W, Hlobil H, Kemper HC. Incidence, severity, aetiology and prevention of sports injuries. A review of concepts. Sports Med 1992: 14: 82–99.
von Porat A, Roos EM, Roos H. High prevalence of osteoarthritis 14 years after an anterior cruciate ligament tear in male soccer players: a study of radiographic and patient relevant outcomes. Ann Rheum Dis 2004: 63: 269–273.
Waldén M, Hägglund M, Ekstrand J. Injuries in Swedish elite football-a prospective study on injury definitions, risk for injury and injury pattern during 2001. Scand J Med Sci Sports 2005: 15: 118–125.
Wedderkopp N, Kaltoft M, Holm R, Froberg K. Comparison of two intervention programmes in young female players in European handball – with and without ancle disc. Scand J Med Sci Sports 2003: 13: 371–375.
