# Supplementary material for: Comparative Effectiveness of Multi-Component, Exercise-Based Interventions for Preventing Soccer-Related Musculoskeletal Injuries: A Systematic Review and Meta-Analysis
Source: Healthcare (Basel). 2025 Mar 29;13(7):765. doi: 10.3390/healthcare13070765 (PMC11988859; doi:10.3390/healthcare13070765)
Supplement: Supplementary file 1 [file healthcare-13-00765-s001.zip › Additional material Included literature/van de Hoef2018.pdf]

Accepted Article

MR SANDER VAN DE HOEF (Orcid ID : 0000-0001-5657-1417)

Article type : Original Article

Does a bounding exercise program prevent hamstring injuries in adult male soccer players? – A cluster –RCT

Corresponding author:
P.A. (Sander) van de Hoef, PT MSc
Utrecht University, University Medical Center Utrecht, Rudolf Magnus Institute of Neurosciences, Department Rehabilitation, Physical Therapy Science & Sports, Utrecht, The Netherlands.
Correspondence: P.O. Box 85500, 3508 GA Utrecht, the Netherlands.
E-mail: p.a.vandehoef@umcutrecht.nl

Co-authors:
M.S. (Michel) Brink, PhD
University of Groningen, University Medical Center Groningen, Center for Human Movement Sciences, Groningen, The Netherlands
Utrecht University, University Medical Center Utrecht, Rudolf Magnus Institute of Neurosciences, Department of Rehabilitation, Physical Therapy Science & Sports, Utrecht, the Netherlands

B.M.A. (Bionka) Huisstede, PT PhD
Utrecht University, University Medical Center Utrecht, Rudolf Magnus Institute of Neurosciences, Department of Rehabilitation, Physical Therapy Science & Sports, Utrecht, the Netherlands

M. (Maarten) van Smeden, PhD
Leiden University Medical Center, department of Clinical Epidemiology, Leiden, the Netherlands

N. (Niels) de Vries, MSc
FIFA Medical Center, Royal Netherlands Football Association, Zeist, the Netherlands
This article has been accepted for publication and undergone full peer review but has not been through the copyediting, typesetting, pagination and proofreading process, which may lead to differences between this version and the Version of Record. Please cite this article as doi: 10.1111/sms.13353

This article is protected by copyright. All rights reserved.

Accepted Article

E.A. (Edwin) Goedhart, MD

FIFA Medical Center, Royal Netherlands Football Association, Zeist, the Netherlands

V. (Vincent) Gouttebarge, PhD

Dutch Consumer Safety Institute, Amsterdam, the Netherlands

Amsterdam Collaboration for Health & Safety in Sports (ACHSS), AMC/VUmc IOC Research Center, Amsterdam, The Netherlands

World Players' Union (FIFPro), Hoofddorp, The Netherlands

Academic Center for Evidence based Sports medicine (ACES), Academic Medical Center, Amsterdam, The Netherlands

Division of Exercise Science and Sports Medicine (ESSM), University of Cape Town, Cape Town, South Africa

F.J.G. (Frank) Backx MD PhD

Utrecht University, University Medical Center Utrecht, Rudolf Magnus Institute of Neurosciences, Department of Rehabilitation, Physical Therapy Science & Sports, Utrecht, the Netherlands

Abstract

Background: Although the Nordic Hamstring Exercise (NHE) prevents hamstring injury in soccer players effectively, the annual incidence of these injuries still increases. This may be because of poor long-term compliance with the program. Furthermore, the timing and amplitude of gluteal and core muscle activation seem to play an important role in hamstring injury prevention, the NHE program was not designed to improve activation of these muscles. Therefore, we propose plyometric training as an alternative to reduce hamstring injuries soccer players.

Purpose: To determine the preventive effect of the Bounding Exercise Program (BEP) on hamstring injury incidence and severity in adult male amateur soccer players.

Study design: A cluster-Randomized Controlled Trial

Methods: Thirty-two soccer teams competing in the first-class amateur league were cluster-randomized into the intervention or control group. Both groups were instructed to perform their regular training program, and the intervention group additionally performed BEP. Information about player characteristics was gathered at baseline and exposure, hamstring injuries and BEP compliance were weekly registered during one season (2016–2017)

This article is protected by copyright. All rights reserved.

Results: The data of 400 players were analyzed. In total, 57 players sustained 65 hamstring injuries. The injury incidence was 1.12/1000 hours in the intervention group and 1.39/1000 hours in the control group. There were no statistically significant differences in hamstring injury incidence (OR=0.89, 95% CI 0.46-1.75) or severity between the groups (p>0.48).

Conclusion: In this large cluster-randomized controlled trial, no evidence was found for plyometric training in its current form to reduce hamstring injuries in amateur soccer players.

Keywords: Hamstring injuries, Injury prevention, Soccer, Plyometric training, Bounding Exercise.

Introduction:

Hamstring injuries are the most common muscle injuries in amateur soccer and account for 15% of all injuries in adult male soccer players(1,2). The high incidence rate (0.7/1000 soccer hours), together with a high recurrence rate (12–30%) and long rehabilitation (mean >28 days) make this injury a major problem in soccer(1,3,4). Hamstring injuries can be classified as sprint-type injuries and stretching-type injuries, with sprint-type hamstring injuries being the most common in soccer(1,5). The sprint-type hamstring injury occur mostly in the late swing phase, when the hamstring undergoes a stretch-shortening cycle(6,7). In this phase, the hamstring eccentrically contracts to decelerate hip flexion and knee extension. Subsequently, it keeps this position of the hip and knee isometrically and concentrically contracts to accelerate for the next foot step(6–11).

To prevent this hamstring injury, the Nordic hamstring exercise program is developed. Several studies indicated the preventive effect of this exercise program(4,12). The Nordic hamstring exercise, by itself or incorporated in an injury prevention program can reduce the hamstring injury rate when compliance is high(13). Although effective programs, like these, have been developed to prevent hamstring injuries, the incidence of hamstring injuries in professional soccer players competing in the UEFA is still increasing by 4% annually(14). As in professional soccer (UEFA), in amateur soccer poor long-term compliance probably limits the effectiveness of interventions such as the NHE(15,16). Reasons for not performing this effective program are poor knowledge of the (effectiveness of the) program and lack of motivation because the exercises are not specific to soccer (submitted data). Soccer coaches in particular do not consider the NHE as soccer-specific enough(17). This is a problem in compliance with injury prevention programs in team sports like soccer, since coaches are crucial implementation components (18)

This article is protected by copyright. All rights reserved.

Accepted Article

In addition to the low compliance with injury prevention programs, eccentric strength training might be less effective than plyometric training. Recent studies suggest that the timing of hamstring muscle activation and the timing and amplitude of gluteal and abdominal muscle activation are important for preventing hamstring injuries. Both can be improved by plyometric exercises(19,20). These exercises strengthen the elastic properties of connective tissue, increase motor unit activation, increase passive tension of the muscle-tendon complex, and improve cross-bridge mechanics(21,22). This improves eccentric strength, joint stiffness, and neuromuscular control, all variables associated with the occurrence of hamstring injuries(23–25).

Therefore, a new functional, soccer-specific program was developed to reduce hamstring injuries, the bounding exercise program (BEP)(26). The BEP aims to improve long-term compliance and increase both eccentric strength and neuromuscular control. The BEP consists of a gradual build-up from concentric, eccentric to plyometric exercises that can easily be incorporated in regular soccer training and which can be performed individually. The exercises are focused specifically on the late swing phase, during which most hamstring injuries occur, and accentuate the horizontal speed to cause optimal loading of the hamstring muscle(26). Plyometric exercises also increase functional performance in tasks common to soccer, such as sprinting and jumping. This might increase implementation of the program in regular training, thereby potentially increasing compliance(27–29). As little is known about the effectiveness of the BEP, the aim of this study was to assess the preventive effect of a functional, soccer-specific BEP on the incidence and severity of hamstring injuries in adult male amateur soccer players.

Methods

The design of this prospective cluster-randomized controlled trial has been described extensively in the research protocol(26).

Study setting

This study was carried out in close collaboration with the FIFA Medical Center, Royal Netherlands Football Association (KNVB). In this prospective, cluster-randomized controlled trial, the BEP was investigated in a real-world context among male amateur soccer players in the first class amateur league. On average, players have two training sessions and one match a

This article is protected by copyright. All rights reserved.

Accepted Article

week during the 39-week soccer season. This study design was approved by the Medical Ethics Committee of the University Medical Center Utrecht (16–332\C), registered in the Dutch Trial Registry (NTR6129) and was partly funded by The Netherlands Organization for Health Research and Development (ZonMw), and the KNVB.

Eligibility criteria

Male amateur soccer players aged 18–45 years and playing in a first class league soccer team were eligible to participate in this study. Players who were injured at the start of the study participated from the moment they returned to play. All players received a patient information letter and signed an informed consent before the start of the study. Players who joined the team after the start of the 2016–2017 season could not participate in the study.

Randomization procedure

Randomization was done by a cluster-randomization procedure. All teams were considered as clusters to avoid a risk of contamination between the players within a team(30). Teams were randomized independently by an online randomizer (https://www.randomizer.org/) and an equal number of teams were assigned to the intervention and control groups.

Intervention

The bounding exercise program (BEP) is a 12-week build-up program (concentric to eccentric to plyometric exercises) and a maintenance program that takes approximately 3–5 minutes to complete(26).

The intervention group performed the BEP (Table 1 and figure 1A,1B,1C) in addition to their regular soccer training. After randomization, all coaches and medical staff of the included soccer clubs attended a workshop in their area to practice how to instruct players to perform the exercises, in order to ensure high-quality performance of the BEP. The control group performed their usual soccer training. Two researchers visited all participating teams to answer questions and monitor the BEP in the intervention group.

This article is protected by copyright. All rights reserved.

Accepted Article

Data collection

Weekly, every player received four or five questions (control or intervention group, respectively) regarding the incidence of hamstring and other injuries, training and match exposure, and compliance with the program (intervention group only). All players could choose to receive the questions by email or short message service (SMS). If a time-loss hamstring injury(31) occurred, the player and medical staff received an additional questionnaire by email regarding the type, location, timing, and occurrence of injury.

Outcomes

The primary outcomes were the incidence of hamstring injuries per 1000 soccer hours and the severity of these injuries. The secondary outcome was compliance with the BEP, calculated as the meters performed divided by total number of meters they performed during regular competition times 100%. The compliance for BEP is measured per player.

Statistical analysis

All data were analyzed with the statistical language and software program 'R'(32). Because of a total average of around 50% of missing data points from the weekly self-reports, multilevel multivariate imputation by chained equations was performed to impute missing data for weekly match and training exposure, thereby accounting for the repeated measurement structure of the data, using the mice R-package(33). Multilevel logistic regression analysis (accounting for the cluster randomization) was performed on imputed data to evaluate and test differences in "any hamstring injury" occurring between the intervention and control groups during the soccer season. Compliance with the program was taken as a covariate in this model. In addition, differences in time-to-first injury between the intervention and control groups were evaluated using a Frailty Cox-regression model. Within the subgroup of players who had at least one hamstring injury during the soccer season, differences in days of absence of soccer between the control and intervention groups were tested using the Wilcoxon Rank test.

Accepted Article

This article is protected by copyright. All rights reserved.

Results

Population

A total of 80 teams were asked to participate in this RCT. Thirty-two teams, accounting for a total of 588 male soccer players, were included in the study. Sixteen teams (N=305 players) were randomly assigned to the intervention group and 16 teams (N= 283 players) to the control group. During the study, 188 players were lost to follow up (32%), because they did not complete the baseline questionnaire (N=140), they did not answer the weekly questions (N=27), they had a severe injury (N=7), or they stopped playing soccer or transferred to another club (N=14). Consequently, the data of 400 players were included in the final analysis, 229 in the intervention group and 171 in the control group (Figure 2 – Flowchart). Baseline characteristics are summarized in Table 2.

Exposure

During the 39-week 2016–2017 season, the total time at risk was 139 hours per player (97 hours training and 42 hours playing matches) in the intervention group and 127 hours per player (90 hours training and 37 hours playing matches) in the control group.

Hamstring injury

There were 65 hamstring injuries, of which 57 were primary hamstring injuries and 8 recurrent hamstring injuries. Thirty-one primary hamstring injuries occurred in the intervention group and 26 in the control group; 4 recurrent hamstring injuries occurred in each group (Table 3). Of the hamstring injuries, 35% were acute injuries and 54% were overuse injuries; the nature of the remaining 11% of the injuries was unknown. Hamstring muscle strains and partial ruptures were the most common types of injury and accounted for 59% of the injuries. Most hamstring injuries occurred during sprinting (39%), followed by jumping (10%) and cutting (6%). Twenty-nine hamstring injuries occurred during a match, 11 players reported the initial hamstring pain after match play, 2 occurred during the pre-match warming-up, and 15 occurred during training; the origin of 8 injuries was not known.

Accepted Article

This article is protected by copyright. All rights reserved.

Incidence and severity

The number of hamstring injuries divided by the exposure time resulted in an overall injury incidence of 1.12/1000 soccer hours for the intervention group and 1.39/1000 soccer hours for the control group. Intention-to-treat analysis showed no statistically significant difference between the intervention and control groups in any hamstring injury during the season (OR =0.89, 95% CI 0.46–1.75), and no significant difference in time-to-first hamstring injury (HR =0.90 ,95% CI = 0.48–1.70).

The mean number of days off play was 33.0 ± 42.7 in the intervention group and 21.35 ± 12.7 days in the control group. Wilcoxon Rank Testing for differences in injury severity between the intervention and control groups showed no statistically significant difference in injury severity (W=344, *p*=0.48).

Compliance

Overall compliance with the BEP was 71%. Figure 3 shows the average number of BEP meters per week. Total compliance was taken as a covariate in the multilevel logistic regression analysis, which showed no evidence of a preventive effect of the BEP on the incidence and severity of hamstring injuries.

Discussion

This cluster-randomized controlled trial evaluated the preventive effect of the BEP on hamstring injury incidence and severity in adult male amateur soccer players. However, BEP did not protect against hamstring injuries incidence or decrease hamstring injury severity.

In line with earlier studies, two out of three hamstring injuries occurred during matches and almost half of the hamstring injuries occurred during high-speed running or sprinting (6,7,11,34,35). The most common type of injury was hamstring muscle strains. In this study, the incidence of hamstring injuries was higher than in previous studies, namely 1.3 per 1000 player hours in comparison to 0.7 per 1000 player hours(4,12), and the severity of hamstring injuries was also different. In this study, 28% of the hamstring injuries resulted in fewer than 7 days off play compared with 17% in the NHE study; however, the incidence of severe injuries was in line with other studies(4,12). A possible explanation for this discrepancy is a difference in the injury registration system used. In previous studies, the coaches or medical staff registered the injuries and registration forms were sent by email. In our study, we used email and SMS services and

This article is protected by copyright. All rights reserved.

Accepted Article

players reported hamstring injuries and exposure, with the injuries being confirmed by the medical staff. However, this approach may have led to a higher number of hamstring injuries than reported in other injury prevention studies(36).

Despite the expected high compliance and effectiveness, the BEP did not reduce the risk or severity of injuries. Compliance is an important factor in reducing the incidence of injuries during, but more importantly after, medical trials(37). Even though the BEP was developed to be easily incorporated into soccer training, overall compliance was 71% and lower than compliance with the NHE(4). This might be because the BEP intervention lasted longer than the NHE intervention, 39 weeks (one whole season) compared with 12 weeks, respectively(4). During this study, the BEP meters decreased almost linearly with time (Figure 2), which suggests that the lower compliance was in part due to the longer intervention period. A longer intervention period was chosen to prevent the detraining effects seen in previous studies, which reported a significant decrease in muscle power output and an increase in sprint time over 10 m and 20 m after a 4- to 6-week detraining period.(38,39)

Another possible reason for the lack of effect of the BEP might be in the technique during the exercises. Although the BEP was designed in accordance with the prescribed training parameters of a plyometric program(24,40), and met the specificity criteria for focusing on the late swing phase(41), the quality of execution of the bounding exercise might influence its effect on the actual dose reaching the hamstring muscle. Unlike NHE, which is a mono-articular exercise and therefore relatively easy to perform, the bounding exercise has multiple degrees of freedom, is a dynamic exercise, and needs better neuromuscular control for good performance. The bounding exercise focuses on reaching a horizontal speed as high as possible, which is in line with an increasing load by a higher sprint speed(8–10,42). If a player fails to reach a speed as high as possible, it might reduce the effect on the hamstring muscle during the late swing phase(8,42).

A final explanation is that the Bounding Exercise Program as we developed it, does not sufficiently load the hamstring or the lumbo-pelvic region sufficiently to gain a preventive effect. In the first six weeks we included triplings, lunges and droplunges in order to prepare players for bounding. However, it is questionable if this already resulted in improvement of hamstring strength (43,44). In the following six weeks bounding was introduced. Although there is extensive evidence that plyometric training can increase eccentric strength, none of these studies specifically focus in the hamstrings. Therefore it remains unclear if the current program does lead to the positive adaptations that we expected.

This article is protected by copyright. All rights reserved.

The BEP was based on recent knowledge of plyometric training(21,22,24,27–29) and implemented in a nationwide trial. Hamstring injuries were prospectively recorded via an on-line registration method and were confirmed by medical staff. This method was highly accessible to the participants, but the burden of weekly registration for a full season may have resulted in missing data. This problem of missing data was resolved by using sophisticated statistical analyses, and the effectiveness of BEP was analyzed with advanced innovative statistical techniques, including multilevel multiple imputation for missing data and multilevel analysis models to account for clustering effects.

Although plyometric training was expected to contribute to hamstring injury prevention (e.g. increased eccentric strength, improved timing and amplitude of hamstring, gluteal, and core muscle activation, and increased passive stretch in muscle tendon complex), to date there is no evidence that the BEP reduces hamstring injuries. This, in combination with the lower compliance with the program, raises the question whether functional and sport-specific exercises are better than less functional and complex exercises. For now, the NHE seems to be the most effective hamstring injury prevention program in male amateur soccer players. Since functional exercises are usually harder to perform and technique is important for the good-quality performance of these exercises(45), a limitation of this large-scale intervention study is that we could not monitor how well individual players performed the BEP because of the large amount of participants. We used a top-down strategy to implement the program in the intervention group, with workshops being organized to teach staff members how to instruct, and if necessary correct, players in how to perform the BEP. All teams in the intervention group received an instruction video and hard-copy instructions. During the soccer season, the researchers visited all teams at least twice to monitor the intervention in the real-life setting. Ideally, the researchers should have visited all teams on a weekly basis to monitor performance of the BEP, but this was not logistically possible.

Future research could assess the quality of performance of the BEP and determine the load during bounding exercises. This could also provide insight into which technique results in optimal adaptation after a 12-week program. Although the BEP did not result in primary prevention, we do not know whether it reduced the rate of injury recurrence. This could be investigated by studying only those players with previous injuries.

This article is protected by copyright. All rights reserved.

Conclusion

This large cluster-randomized controlled trial found no evidence that a new functional injury prevention exercise program prevented hamstring injuries in adult male amateur soccer players.

Perspective

Effective hamstring injury prevention programs did not accomplish an annual decrease of hamstring injuries in male amateur soccer players. One of the main reasons is long term compliance for these programs. Besides the compliance, not only eccentric hamstring strength but also gluteal and core muscle activation patterns seem to be important factors in hamstring injury prevention. This large cluster-randomized controlled trial is the first large trial investigating the preventive effects of plyometric training on hamstring injury incidence and severity in adult male amateur soccer players. This study did not find evidence for a preventive effect of BEP in its current form. Reasons for this result can be found in a lower compliance than expected and quality of performance of the BEP. A lower compliance and poor quality of performance could both lead to undertraining which could explain the absence of preventive effect. Finally, it could also be argued that the load of the hamstring or lumbo-pelvic region was insufficient to gain protective adaptations, regardless of the quantity and quality of BEP.

Acknowledgements

The authors thank the Royal Dutch Football Association (KNVB) and players, coaches and medical staff members of the participating teams for their contribution in this study. We would also like to thank Studio Sterkenburg (Richard Sterkenburg) for taking the photos of the BEP and Sven Brake for being the model in these pictures. Furthermore we would like to thank, Jur Brauers, Esmee de Jong and Rick Nevels for their contributions in this trial.

Funding

This study was funded by ZonMw: The Netherlands Organisation for Health Research and Development.

This article is protected by copyright. All rights reserved.

Accepted Article

Accepted Article

Ethical approval and patient consent

This study is approved by the Medical Ethics Committee of the University Medical Center Utrecht (16-332\C). All participants included in this study have provided written informed informed consent.

References

1. Ekstrand J, Hägglund M, Waldén M. Epidemiology of muscle injuries in professional football (soccer). Am J Sports Med. 2011;

2. Ekstrand J, H??gglund M, Wald??n M. Injury incidence and injury patterns in professional football: The UEFA injury study. Br J Sports Med. 2011;

3. Van Beijsterveldt AMC, Van De Port IGL, Krist MR, Schmikli SL, Stubbe JH, Frederiks JE, et al. Effectiveness of an injury prevention programme for adult male amateur soccer players: A cluster-randomised controlled trial. Sport en Geneeskd. 2012;

4. van der Horst N, Smits D-W, Petersen J, Goedhart EA, Backx FJG. The Preventive Effect of the Nordic Hamstring Exercise on Hamstring Injuries in Amateur Soccer Players. Am J Sports Med [Internet]. 2015 Jun 20;43(6):1316–23. Available from: http://journals.sagepub.com/doi/10.1177/0363546515574057

5. Hägglund M, Waldén M, Ekstrand J. Risk factors for lower extremity muscle injury in professional soccer: The UEFA injury study. Am J Sports Med. 2013;

6. Askling C, Thorstensson A. Hamstring muscle strain in sprinters. New Studies in Athletics. 2008.

7. Heiderscheit BC, Sherry MA, Silder A, Chumanov ES, Thelen DG. Hamstring Strain Injuries: Recommendations for Diagnosis, Rehabilitation, and Injury Prevention. J Orthop Sport Phys Ther. 2010;

8. Chumanov ES, Heiderscheit BC, Thelen DG. The effect of speed and influence of individual muscles on hamstring mechanics during the swing phase of sprinting. J Biomech. 2007;

9. Schache AG, Dorn TW, Blanch PD, Brown NAT, Pandy MG. Mechanics of the human hamstring muscles during sprinting. Med Sci Sports Exerc. 2012;

10. Thelen DG, Chumanov ES, Best TM, Swanson SC, Heiderscheit BC. Simulation of biceps

This article is protected by copyright. All rights reserved.

Accepted Article

femoris musculotendon mechanics during the swing phase of sprinting. In: Medicine and Science in Sports and Exercise. 2005.

11. Woods C, Hawkins R, Maltby S, Hulse M, Thomas A, Hodson A. The Football Association Medical Research Programme: an audit of injuries in professional football--analysis of hamstring injuries. Br J Sports Med [Internet]. 2004 Feb 1;38(1):36–41. Available from: http://bjsm.bmj.com/cgi/doi/10.1136/bjsm.2002.002352

12. Petersen J, Thorborg K, Nielsen MB, Budtz-Jørgensen E, Hölmich P. Preventive effect of eccentric training on acute hamstring injuries in Men's soccer: A cluster-randomized controlled trial. Am J Sports Med. 2011;

13. Al Attar WSA, Soomro N, Sinclair PJ, Pappas E, Sanders RH. Effect of Injury Prevention Programs that Include the Nordic Hamstring Exercise on Hamstring Injury Rates in Soccer Players: A Systematic Review and Meta-Analysis. Sports Medicine. 2017.

14. Ekstrand J, Waldén M, Hägglund M. Hamstring injuries have increased by 4% annually in men's professional football, since 2001: A 13-year longitudinal analysis of the UEFA Elite Club injury study. Br J Sports Med. 2016;

15. Bahr R, Thorborg K, Ekstrand J. Evidence-based hamstring injury prevention is not adopted by the majority of Champions League or Norwegian Premier League football teams: The Nordic Hamstring survey. Br J Sports Med. 2015;

16. van Reijen M, Vriend I, van Mechelen W, Finch CF, Verhagen EA. Compliance with Sport Injury Prevention Interventions in Randomised Controlled Trials: A Systematic Review. Sports Medicine. 2016.

17. Gambetta, V. & Benton D. A systematic approach to hamstring prevention & rehabilitation. Sport Coach. 2008;28(4):1–6.

18. O'Brien J, Finch CF. A systematic review of core implementation components in team ball sport injury prevention trials. Injury prevention : journal of the International Society for Child and Adolescent Injury Prevention. 2014.

19. Panayi S. The need for lumbar-pelvic assessment in the resolution of chronic hamstring strain. J Bodyw Mov Ther. 2010;

20. Schuermans J, Danneels L, Van Tiggelen D, Palmans T, Witvrouw E. Proximal Neuromuscular Control Protects Against Hamstring Injuries in Male Soccer Players: A Prospective Study with Electromyography Time-Series Analysis during Maximal

This article is protected by copyright. All rights reserved.

Accepted Article

Sprinting. Am J Sports Med. 2017;

21. Malisoux L. Stretch-shortening cycle exercises: an effective training paradigm to enhance power output of human single muscle fibers. J Appl Physiol. 2005;

22. Ramírez-Campillo R, Burgos CH, Henríquez-Olguín C, Andrade DC, Martínez C, Álvarez C, et al. Effect of unilateral, bilateral, and combined plyometric training on explosive and endurance performance of young soccer players. J Strength Cond Res. 2015;

23. Komi P V. Stretch-shortening cycle. Strength Power Sport. 1992;

24. Ramirez-Campillo R, Henriquez-Olguin C, BURGOS C, ANDRADE DC, Zapata D, Martinez C, et al. Effect of Progressive Volume-Based Overload During Plyometric Training on Explosive and Endurance Performance in Young Soccer Players. J Strength Cond Res. 2015;

25. Toumi H, Best TM, Martin A, Poumarat G. Muscle plasticity after weight and combined (weight + jump) training. Med Sci Sports Exerc. 2004;

26. Van De Hoef S, Huisstede BMA, Brink MS, De Vries N, Goedhart EA, Backx FJG. The preventive effect of the bounding exercise programme on hamstring injuries in amateur soccer players: The design of a randomized controlled trial. BMC Musculoskelet Disord. 2017;18(1).

27. Bedoya AA, Miltenberger MR, Lopez RM. Plyometric Training Effects on Athletic Performance in Youth Soccer Athletes: A Systematic Review. Journal of Strength and Conditioning Research. 2015.

28. Markovic G, Mikulic P. Neuro-musculoskeletal and performance adaptations to lower-extremity plyometric training. Sport Med. 2010;

29. Slimani M, Chamari K, Miarka B, Del Vecchio FB, Chéour F. Effects of Plyometric Training on Physical Fitness in Team Sport Athletes: A Systematic Review. Journal of Human Kinetics. 2016.

30. Emery CA. Considering cluster analysis in sport medicine and injury prevention research. Clinical Journal of Sport Medicine. 2007.

31. Fuller CW, Molloy MG, Bagate C, Bahr R, Brooks JHM, Donson H, et al. Consensus statement on injury definitions and data collection procedures for studies of injuries in rugby union. Clin J Sport Med. 2007;

This article is protected by copyright. All rights reserved.

32. R Core Team. R Core Team (2014). R: A language and environment for statistical computing. R Found Stat Comput Vienna, Austria URL http//wwwR-project.org/. 2014;

33. Buuren S van, Groothuis-Oudshoorn K. **mice** : Multivariate Imputation by Chained Equations in *R*. J Stat Softw. 2011;

34. Árnason Á, Gudmundsson Á, Dahl HA, Jóhannsson E. Soccer injuries in Iceland. Scand J Med Sci Sports. 2007;

35. Freckleton G, Pizzari T. Risk factors for hamstring muscle strain injury in sport: a systematic review and meta-analysis. Br J Sports Med [Internet]. 2013 Apr;47(6):351–8. Available from: http://bjsm.bmj.com/lookup/doi/10.1136/bjsports-2011-090664

36. Nilstad A, Bahr R, Andersen T. Text messaging as a new method for injury registration in sports: A methodological study in elite female football. Scand J Med Sci Sport. 2014;

37. Hanson D, Allegrante JP, Sleet DA, Finch CF. Research alone is not sufficient to prevent sports injury. British Journal of Sports Medicine. 2014.

38. Izquierdo M, Ibañez J, González-Badillo JJ, Ratamess NA, Kraemer WJ, Häkkinen K, et al. Detraining and tapering effects on hormonal responses and strength performance. J Strength Cond Res. 2007;

39. Koundourakis NE, Androulakis NE, Malliaraki N, Tsatsanis C, Venihaki M, Margioris AN. Discrepancy between exercise performance, body composition, and sex steroid response after a six-week detraining period in professional soccer players. PLoS One. 2014;

40. De Villarreal ESS, Kellis E, Kraemer WJ, Izquierdo M. Determining variables of plyometric training for improving vertical jump height performance: A meta-analysis. J Strength Cond Res. 2009;

41. Sale DG. Neural adaptation to resistance training. Med Sci Sports Exerc. 1988;

42. Chumanov ES, Wille CM, Michalski MP, Heiderscheit BC. Changes in muscle activation patterns when running step rate is increased. Gait Posture. 2012;

43. Bourne MN, Williams MD, Opar DA, Al Najjar A, Kerr GK, Shield AJ. Impact of exercise selection on hamstring muscle activation. Br J Sports Med. 2017;

44. Brughelli M, Nosaka K, Cronin J. Application of eccentric exercise on an Australian Rules football player with recurrent hamstring injuries. Phys Ther Sport. 2009;

Accepted Article

This article is protected by copyright. All rights reserved.

Accepted Article

45. Marshall BM, Moran KA. Which drop jump technique is most effective at enhancing countermovement jump ability, “countermovement” drop jump or “bounce” drop jump? J Sports Sci. 2013;

This article is protected by copyright. All rights reserved.

Accepted Article

Table 1. Bounding exercise program

Week	Program
1	2x30m walking lunges (2x10)
2	3x30m walking lunges (3x10)
3	3x30m walking lunges + 1x30m triplings + droplunges)
4	2x30m triplings + droplunges (2x10)
5	3x30m triplings + droplunges (3x10)
6	3x30m triplings + droplunges + 1x 30m bounding
7	2x20m bounding (+/- 7 jumps)
8	3x20m bounding (+/- 7 jumps)
9	4x20m bounding (+/- 7 jumps)
10	3x30m bounding (+/-10 jumps)
11	4x30m bounding (+/- 10 jumps)
12	4x30m bounding (in the fewest possible jumps)
13 until end of the soccer season	3x30m bounding (in the fewest possible jumps)

This article is protected by copyright. All rights reserved.

Table 2. Baseline characteristics

		Intervention Group (N=229)	Control group (N=171)
Age, yrs		23.8 ± 6.4	22.2 ± 3.1
Height, cm		183.0 ± 8.6	182.6 ± 6.6
Weight, kg		78.88 ± 8.6	76.15 ± 7.4
Dutch nationality % (N)		98.3% (225)	98.2% (168)
Soccer experience, yrs		16.4 ± 4.6	16.3 ± 4.0
Leg dominance			
	Right	70% (160)	69% (118)
	Left	18% (41)	20% (34)
	Two-legged	12% (28)	11% (19)
Field position			
	Forwarder	24% (54)	21% (36)
	Midfielder	32% (74)	35% (59)
	Defender	32% (74)	36% (62)
	Goalkeeper	12% (27)	7% (12)
Number of hamstring injuries in previous competition	(2015-2016)		
	1	15% (35)	20% (34)
	2	5% (12)	3% (5)
	3	1% (3)	4% (7)
ADL			
	Work	54% (123)	57% (98)
	Study	21% (49)	23% (39)
	Both	25% (57)	20% (34)

Accepted Article

This article is protected by copyright. All rights reserved.

Table 3. Comparison between intervention and control group

		Intervention Group (N=229)	Control group (N=171)
Exposure (hours)			
	Training	97.1	90.3
	Match	42.2	36.7
	Total	139.3	127.0
Hamstring injuries (N=57)		31	26
	acute	20	15
Recurrent injuries (N=8)		4	4
Injury severity (days of soccer absence)		36 ± 67	22 ± 12
Injuries by severity (N)			
	Slight (0 days)	1	3
	Minimal (1-3 days)	3	0
	Mild (4-7 days)	6	3
	Moderate (8-28 days)	14	15
	Severe (>28 days)	7	5
Recurrent injuries (N)	Slight (0 days)	1	0
	Minimal (1-3 days)	0	0
	Mild (4-7 days)	0	0
	Moderate (8-28 days)	1	3
	Severe (>28 days)	2	1
Type of injury (N)			
	Strain	22	10
	(partial) rupture)	4	3
	Tendon injury	1	1
	Muscle cramps	4	3
	DOMS	5	9
	Overuse injury	8	9

Accepted Article

This article is protected by copyright. All rights reserved.

Accepted Article

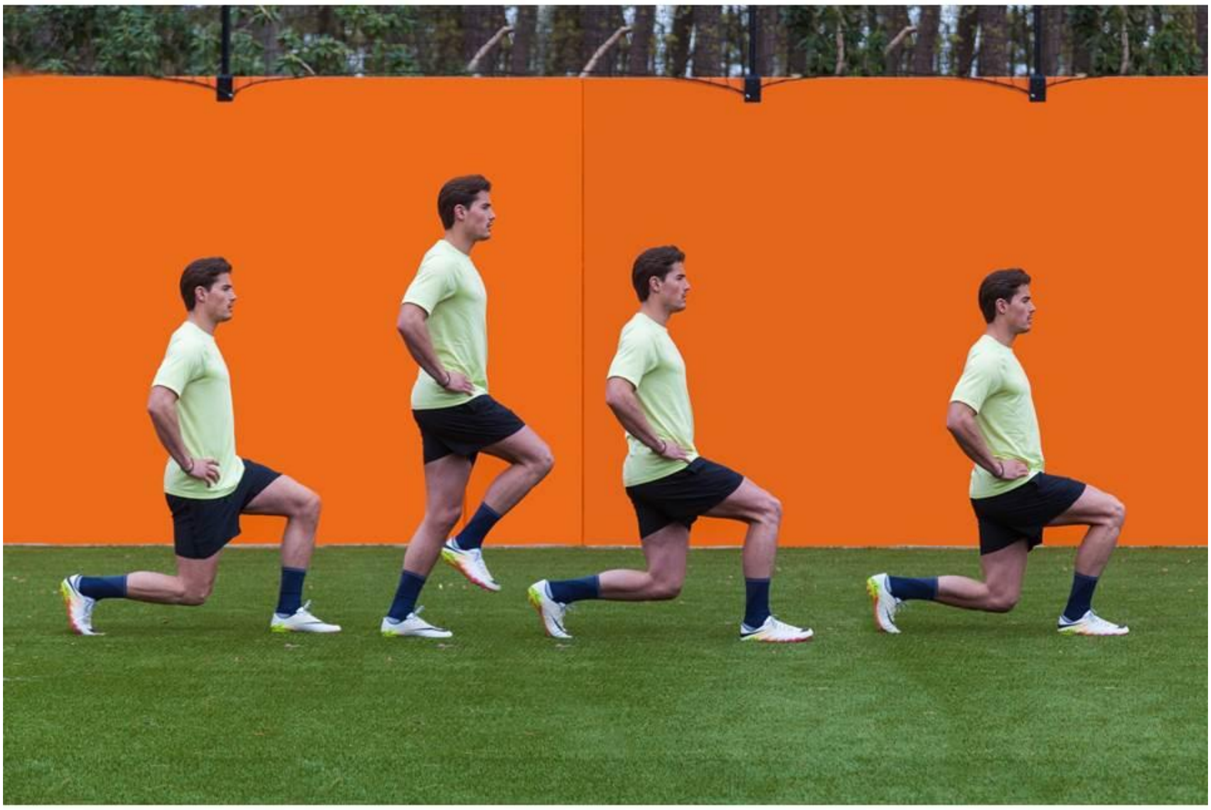

Figure 1A. Walking lunges

This article is protected by copyright. All rights reserved.

Accepted Article

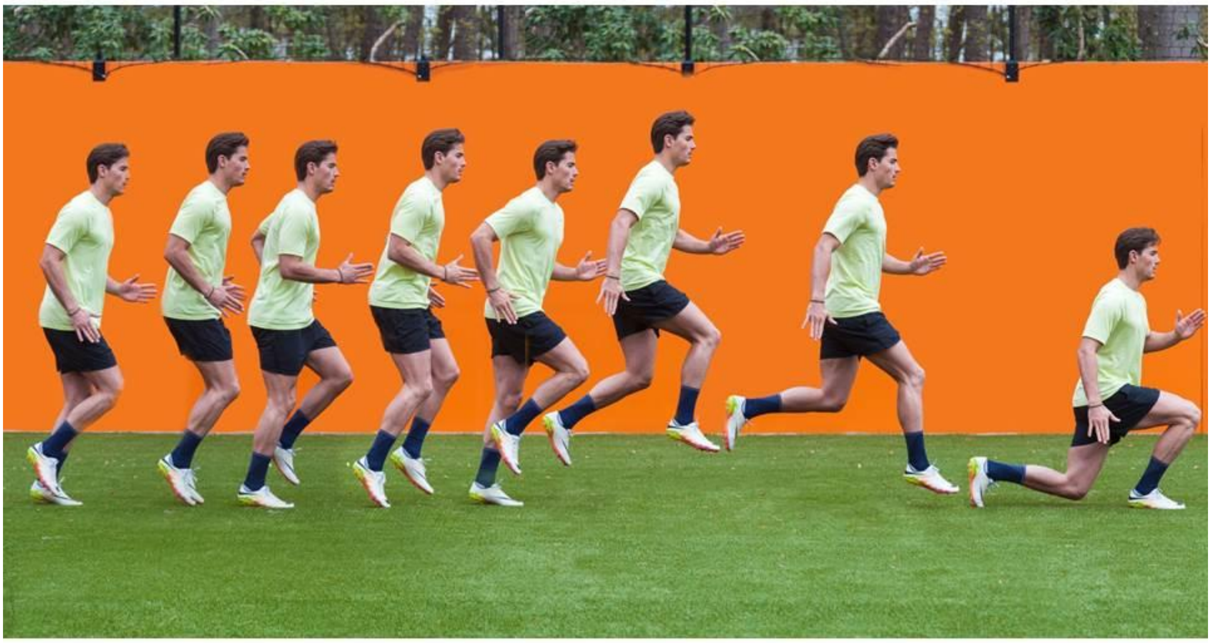

Figure 1B. Triplings and droplunges

This article is protected by copyright. All rights reserved.

Accepted Article

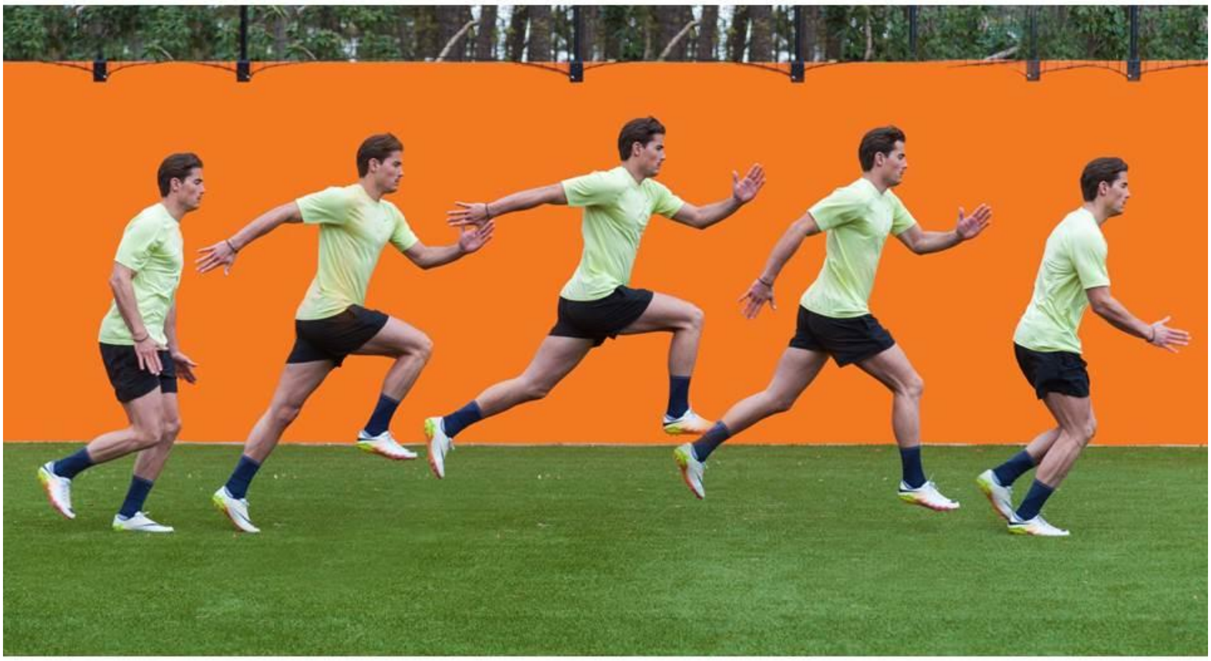
Figure 1C Bounding

This article is protected by copyright. All rights reserved.

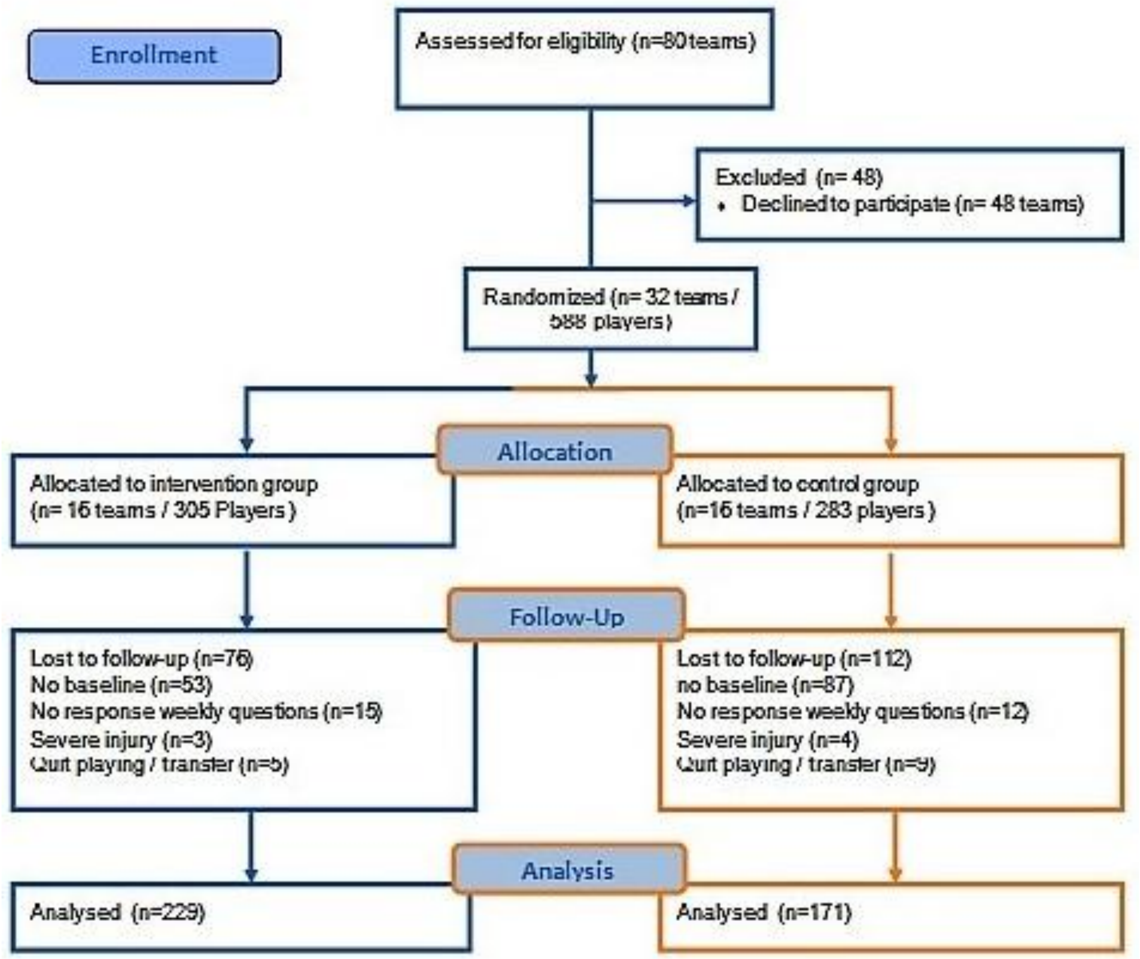

Figure 2. Consort flowchart

Accepted Article

This article is protected by copyright. All rights reserved.

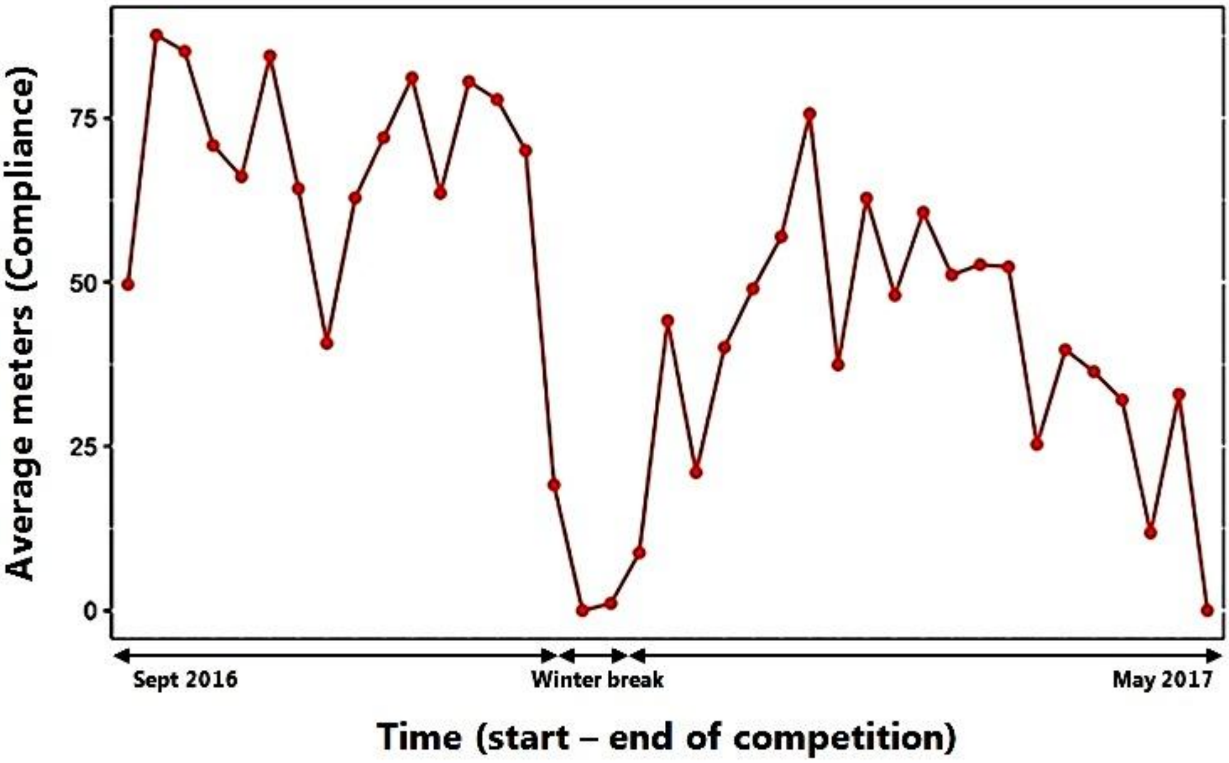

Figure 3. Compliance during the soccer season 2016-2017

This article is protected by copyright. All rights reserved.
