# Supplementary material for: Comparative Effectiveness of Multi-Component, Exercise-Based Interventions for Preventing Soccer-Related Musculoskeletal Injuries: A Systematic Review and Meta-Analysis
Source: Healthcare (Basel). 2025 Mar 29;13(7):765. doi: 10.3390/healthcare13070765 (PMC11988859; doi:10.3390/healthcare13070765)
Supplement: Supplementary file 1 [file healthcare-13-00765-s001.zip › Additional material Included literature/van der Horst2015.pdf]

Effective But Not Adhered to: How Can We Improve Adherence to Evidence-Based Hamstring Injury Prevention in Amateur Football?

Nick van der Horst, PT, MSc,* Sander van de Hoef, PT, MSc,* Paul van Otterloo, PT, MSc,* Milan Klein, PT, MSc,* Michel Brink, PhD,*† and Frank Backx, MD, PhD*

Abstract

Objectives: To investigate adherence to a Nordic hamstring exercise (NHE) program in a real-world context of male amateur football, and the perceptions of end users (players) and intervention deliverers (coaches and medical staff) about adherence to this proven effective program. **Design:** Retrospective cohort study. **Setting:** Dutch amateur football. **Participants:** Two hundred sixty-four players, 23 coaches, and 29 medical staff from Dutch amateur football teams that participated in a national randomized controlled trial 2 years earlier. **Independent Variables:** Nordic hamstring exercise program. **Main Outcome Measures:** Nordic hamstring exercise program adherence during 2014 and 2015. Intervention or control group allocation during the trial, transfers, and personal perception about adherence to the program were also examined. **Results:** Of all players, 69% reported never, 16% sometimes, 6% frequently, 5% often, and 4% always performing exercises of the NHE program. Adherence to the NHE program was higher among players who had been in the NHE arm of the previous trial and among players who had not been transferred to another club compared with players who had been transferred. Key factors in stimulating players to adhere to the NHE program were knowledge of the NHE and personal motivation. Coaches and medical staff members also mentioned personal motivation and consensus with team staff as key factors to encourage NHE adherence. **Conclusions:** Among high-level male amateur football players, adherence to an evidence-based hamstring injury-prevention program was very low. It is essential to recognize factors that stimulate or limit adherence to injury-prevention programs for effective programs to actually lead to a reduction in hamstring injuries in a real-world context.

Key Words: hamstring injury, prevention, football, soccer, adherence

(*Clin J Sport Med* 2018;00:1–7)

INTRODUCTION

Hamstring injuries are a primary target for injury prevention in football because they are the most common muscle injury and have a high recurrence rate (12%-33%).[1,2] On average, a team squad (usually 25 players) suffers about 5 to 6 hamstring injuries each season, losing a total of ~80 days from football (including match and training) activities. These injuries not only have severe personal, medical, and financial consequences, but are also related to decreased performance caused by the unavailability of players for matches.[3] Since 2001, the rate of hamstring injury in football has increased by 4% annually.[1]

The Nordic hamstring exercise (NHE) program was specifically developed to prevent hamstring injuries (Figure 1, NHE). The NHE can easily be incorporated into regular training sessions and increases eccentric hamstring strength.[4–6] Large-scale randomized controlled trials (RCTs) have shown that when the NHE program is incorporated in regular training, the rate of first-time hamstring injuries is reduced by more than 60%,[4,6] and recurrent injury rates are reduced by 85%.[4]

Unfortunately, injury-prevention programs that have proven to be effective in trials do not necessarily reduce injury incidence in a real-world setting.[7] There is still a gap between compliance in a research setting (eg, an individual following professional recommendations regarding prescribed dosage, timing, and frequency of an intervention) and adherence in a real-world setting (where the process is influenced by environment, social context, personal knowledge, motivation, skills, and resources).[8,9] Sports injury-prevention programs can only benefit the health of athletes if they are adopted by the intended end users (eg, the athletes themselves).[10,11]

To better understand health-promoting behavior, social psychologists developed the health-belief model (HBM)[12,13] in the 1950s, which is still widely used in health behavior research. This model suggests that people's beliefs about health problems, perceived benefits of action and barriers to action, and self-efficacy explain (lack of) engagement in health-promoting behavior.[12–14] In this model, the perceived susceptibility (ie, beliefs about the risk of sustaining an injury) and the perceived seriousness (ie, beliefs about the consequences of the injury for health and sport activities) lead to the perceived threat. The perceived threat, along with the

Submitted for publication May 9, 2018; accepted October 30, 2018.

*From the *Department of Rehabilitation, Physical Therapy Science and Sports, Rudolf Magnus Institute of Neurosciences, University Medical Center Utrecht, Utrecht, the Netherlands; and †Center for Human Movement Sciences, University Medical Center Groningen, University of Groningen, Groningen, the Netherlands.*

The authors report no conflicts of interest.

Corresponding Author: Nick van der Horst, PT, MSc, Department of Rehabilitation, Physical Therapy Science and Sports, Rudolf Magnus Institute of Neurosciences, University Medical Center Utrecht, P.O. Box 85500, 3508 GA Utrecht, the Netherlands (n.vanderhorst-3@umcutrecht.nl).

Copyright © 2018 Wolters Kluwer Health, Inc. All rights reserved.

http://dx.doi.org/10.1097/JSM.0000000000000710

Copyright © 2018 Wolters Kluwer Health, Inc. Unauthorized reproduction of this article is prohibited.

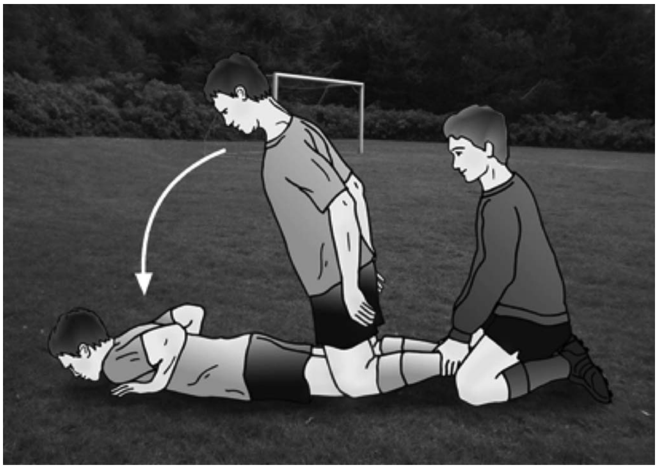

Figure 1. The Nordic hamstring exercise.

perceived benefits (ie, beliefs about the effectiveness of injury prevention) and perceived barriers (ie, beliefs about the negative aspects of adopting injury preventive measures), self-efficacy (ie, the player's belief how well he can execute preventive exercises) and cues to action (ie, stimuli to motivate players to take action to prevent injuries) ultimately lead to the likelihood of engaging in health-promoting behavior. Consequently, some studies have shown that adoption of preventive measures can be stimulated, and inhibited, by players' motivation,[15] staff support,[16,17] and knowledge about injuries and injury prevention.[18–20]

Recently, a study showed that the NHE program, for which there is compelling evidence of effectiveness, has not been adopted by the majority of football players in Champions League teams and Norwegian Premier League football teams (where the pioneer research was conducted).[21] However, the arguments for nonadherence to NHEs were not investigated, and factors that could improve NHE adherence were not addressed either. Furthermore, amateur footballers are the largest subgroup of football players, and there are significant differences between professional and amateur football in terms of medical staff, level of play, financial considerations, and perceptions about injury prevention, which could all influence adherence to injury-prevention programs.

Therefore, the aim of this study was 2-fold: (1) to determine adherence to the NHE program in a real-life context of male amateur football players, and (2) to investigate the perceptions of targeted end users (eg, players) and program deliverers (eg, coaches and medical staff) regarding adherence to the evidence-based NHE program for the prevention of hamstring injuries.

MATERIALS AND METHODS

This study was conducted as a follow-up of a large cluster RCT, with data collection during 2013, of the preventive effect of the NHE program on hamstring injuries in male amateur football players.[6] The RCT was designed and performed in close collaboration with the Royal Netherlands Football Association (KNVB).[22] Following excellent compliance (91% of players performed all sessions) and high effectiveness [odds ratio 0.282; 95% confidence interval (CI), 0.110-0.721, $P = 0.005$] in the RCT, a dissemination strategy was conducted to share results with the Dutch (amateur and professional) football medicine community. Study findings were presented in the media, in courses, at conferences, and (scientific) in journals and by organizing a meeting for all participating coaches and medical staff (both intervention and control groups) to share and discuss the results of the study as well as the future implementation of the NHE program. The Medical Ethics Committee of the University Medical Center Utrecht approved the follow-up study (No. 15/661), and ethical guidelines were followed. The follow-up data in this study entail adherence data of 2014 and 2015, which is the 2-year period immediately after the RCT was conducted and when there was no monitoring by the research team anymore. Before the study, participants were informed of the study aims and procedures. Informed consent was obtained from all players, coaches, and medical staff [eg, (sports) physiotherapists and/or sports masseurs].

All first-class amateur football players (n = 579), coaches (n = 38), and medical staff (n = 47) involved in the RCT were invited to participate in the follow-up study through email and/or phone if there was no response to the email. Each potential participant was contacted with a minimum of 3 attempts through phone and/or email. Participants were excluded if they did not provide informed consent or if they had not been active at top amateur playing level (eg, professional or more than one level below the level of the original RCT) for 1 year or longer during follow-up.

A questionnaire addressing adherence to the NHE program was developed specifically for players, coaches, and medical staff. The first part of the questionnaire focused on measuring NHE adherence by the intended end users (eg, players). The second part of the questionnaire focused on potential factors that could contribute to adherence, based on elements of the HBM.[12–14] These factors, such as knowledge, (personal) motivation, effectiveness, and environment, were extracted from the scientific literature on compliance and/or adherence to exercise programs in sports.[15–20] To provide insights on injury perceptions, self-efficacy, and cues to action and ultimately their likelihood of adhering to the NHE program, players, coaches, and medical staff were asked to indicate which factors contributed to their adherence to the NHE program.

During an independent peer-review procedure, the questionnaire was pilot tested by 4 experts in the field of sports injury research to ensure clarity of the questionnaire, suitability to the participants, reader friendliness, and time consumption. Subsequently, a standardized interview was developed, which was pilot tested before data collection to ensure uniformity and standardization of the data collection procedure. Two independent researchers (P.O. and M.K.) administered the questionnaires through structured telephone interviews from November 2015 to January 2016. Answers were entered on a standardized registration form.

The data were analyzed using SPSS version 23.0 (IBM Corp, New York, NY). Statistical significance was set at 0.05 for all tests. Descriptive statistics (mean values and SDs) were used to describe baseline characteristics and adherence data.

The full NHE program consisted of 25 sessions in 13 weeks.[6] Performance of the program was classified on a Likert scale in 5 categories as never (0 sessions), sometimes (1-8 sessions), regular (9-16 sessions), often (17-24 session), and always (25 sessions or more). In addition, *t* tests were performed to analyze potential group differences regarding adherence between (1) intervention versus control players, (2) players with and without a history of hamstring injury,

Copyright © 2018 Wolters Kluwer Health, Inc. Unauthorized reproduction of this article is prohibited.

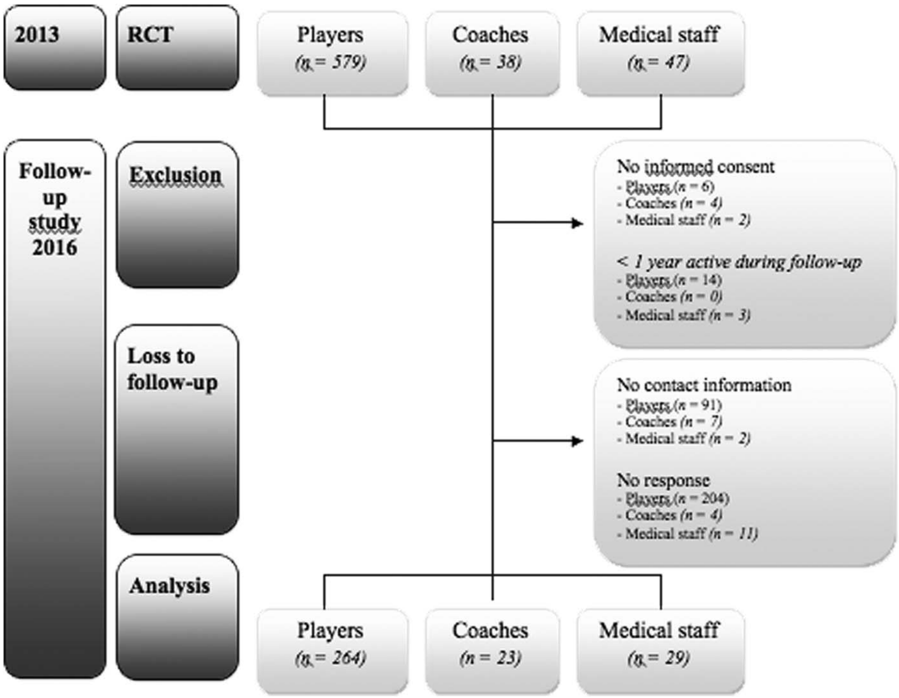

Figure 2. Flowchart of the study population.

and (3) players who had been transferred to another club or remained at the club during the follow-up. Factors that could influence adherence were scored on a 3-point Likert scale (less important, neutral, and important). An item was considered important if 75% or more of the respondents considered it important.[23]

RESULTS

A total of 664 participants from the original RCT were invited to participate in this follow-up study. After exclusion and loss to follow-up, 264 players, 23 coaches, and 29 medical staff were included (Figure 2, flowchart). Of the 264 included players, 135 (51.1%) had been in the intervention group in the original RCT and 129 (49%) had been in the control group. Baseline characteristics of all players, coaches, and medical staff members are summarized in Table 1.

After approximately 2 years, 69% (n = 180) of the participants reported never performing the NHE program and 14% (n = 38) reported sometimes performing the exercises (1-8 sessions). The NHE program was performed on a regular basis (9-16 sessions) by 10% (n = 27), often (17-

TABLE 1. Baseline Characteristics of the Study Participants

**Player characteristics (n = 264)**	**Mean (SD)/%**
Age (yrs)	26.3 (±3.7)
Football experience (yrs)	19.9 (±4.2)
Field position*	
Forward	29.2% (n = 76)
Midfielder	33.5% (n = 87)
Defender	34.6% (n = 90)
Goalkeeper	12.3% (n = 32)
Hamstring injury in previous year	20% (n = 52)
Other football injuries in previous year	59.2% (n = 154)
**Coach characteristics (n = 23)**	**Mean (SD)**
Age (yrs)	47.4 (±9.7)
Coaching experience (yrs)	20 (±10)
**Medical staff characteristics (n = 29)**	**Mean (SD)**
Age (yrs)	45.2 (±11.9)
Medical staff experience (yrs)	18.1 (±10.8)

Some players had multiple field positions.

Copyright © 2018 Wolters Kluwer Health, Inc. Unauthorized reproduction of this article is prohibited.

24 session) by 4% (n = 10), and always by 3% (n = 8) of the participants (Figure 3).

Adherence to the NHE program was significantly higher in those players who were in the intervention group in the previous trial than in those who were in the control group ($t = -4.460$; 95% CI: −0.851 to −0.329; $P < 0.001$) and in players who remained at their club compared with players who transferred to other clubs ($t = 2.572$; 95% CI: 0.081-0.612; $P = 0.011$). There was no statistically significant difference in adherence ($t = 0.608$; 95% CI: 0.231-0.437; $P = 0.544$) between players with and without a history of hamstring injury during the follow-up period.

Participants' perceptions of factors that contributed to maintenance or increased adherence to the NHE program for the prevention of hamstring injuries are provided in Table 2. Some factors considered by players, coaches, and medical staff members to contribute to continued or increased adherence to the NHE program overlapped (Table 2), although there were some role-specific differences.

Players stated that knowledge of the NHE program and personal motivation were the primary reasons to perform the exercise program (Table 2). Coaches stated that effectiveness and knowledge of the NHE program, and personal motivation were the primary reasons to use the program. Medical staff stated that effectiveness and knowledge of the NHE program, consensus with the team staff (primarily coach), and personal motivation were the main reasons to use the NHE program.

DISCUSSION

This study investigated adherence to an evidence-based hamstring injury-prevention program in a real-world context of male amateur football players, and factors perceived by targeted end users (eg, players, coaches, and medical staff) that influence the adherence to the program. The results showed that the majority of the players (69%) did not adhere to the NHE program after a 2-year follow-up, and adherence was higher among players who already had experienced the NHE program in the RCT. Players stated that "personal motivation" and "knowledge of the NHE program" were important factors for adherence. Coaches additionally stated that

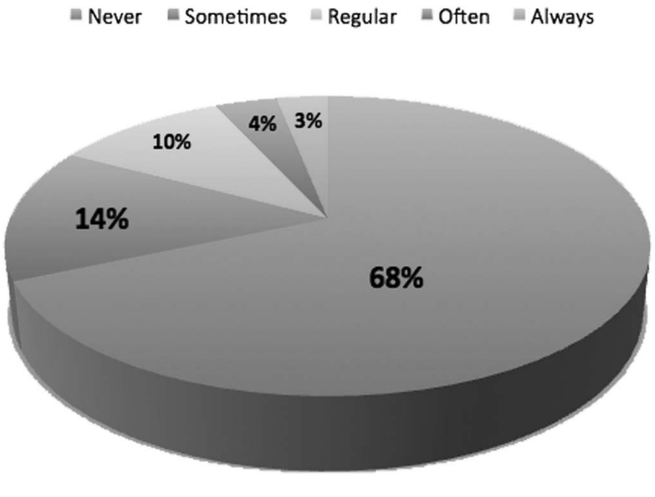

Figure 3. Adherence to the NHE program.

effectiveness of the NHE program was important, and medical staff considered "consensus with staff" and "motivation of players" as important factors.

Bahr et al[21] also studied adherence to an NHE program, but in a professional football environment, where adherence to injury preventive measures would be expected to be higher. They investigated the medical staff of 50 professional football teams from Champions League teams and Norwegian premier league teams, where pioneer research on NHEs was performed.[4,5] However, even in a professional environment, adoption of the NHE program was low–only 10.7% of all clubs were adherent with the NHE program.[21] Interestingly, a study on adherence to neuromuscular training aimed at reducing anterior cruciate ligament injury rates in female adolescent football revealed similar results.[25] Despite high effectiveness and adoption, the neuromuscular training program was only used sporadically, revealing low fidelity to the program protocol.[24] Other studies of adherence or compliance to other preventive measures have also reported poor adherence,[25–27] although it is recognized that the effectiveness of injury-prevention strategies is dependent on adherence.[15,18,28–31] While proven effectiveness strengthens athletes' willingness to perform exercise programs,[32] other factors must be important as well because adherence to the NHE program was poor although there is compelling evidence of its effectiveness.[4–6] We found that while overall adherence was low, it was higher among players who already had performed the NHE program during the original RCT.

This is in line with the HBM because positive player experiences with the NHE program could have contributed to their perceived benefits of the NHE program. From a practical perspective, this suggests that the supervised introduction of the NHE program could familiarize players with the program and improve adherence. In addition, the role of the team staff (eg, coach and/or medical staff) as the program deliverers and important cue to action for players must be considered. Team staff members are responsible for physical training and if they include injury prevention in their program, players are likely to follow the instructions of the team staff. Therefore, factors that promote team staff to implement the program are also very important if we aim for maximal adherence to NHE, or potentially any other, prevention programs. In addition, it needs to be emphasized that there is a responsibility as well to ensure the proper knowledge transfer and instruction follow-up from coaches to the athletes.

Key factors for all stakeholders to consider when aiming to promote or improve adherence are knowledge of the NHE and personal motivation. However, each stakeholder also mentioned other factors as well. These factors to promote adherence to injury-prevention strategies among targeted end users and those who deliver the program need to be considered if we want evidence-based hamstring injury prevention to truly work in the real world. So far, no studies have investigated the relationship between adherence and effectiveness of hamstring injury-prevention programs. In a study of team adherence to FIFA 11+ exercises and injury risk in Canadian female youth football players,[33] the injury rate among players with high adherence was 57% lower than the injury rate among players with low adherence. However, after adjustment for team, age group, level of play, and injury history, this between-group difference was not statistically significant. Greater understanding is required regarding the relationship

Copyright © 2018 Wolters Kluwer Health, Inc. Unauthorized reproduction of this article is prohibited.

TABLE 2. Factors That Contributed to Continued or Increased Adherence to the NHE Program For the Prevention of Hamstring Injuries

	Important % (n)	Neutral % (n)	Less Important % (n)
Male football players (n = 264)			
Knowledge of NHE	**85** (225)	11 (29)	4 (10)
Personal motivation	**81** (215)	8 (22)	10 (27)
Effectiveness of NHE	74 (194)	21 (55)	6 (15)
Stimulus from coach	54 (143)	19 (51)	27 (70)
Support from board of directors	52 (138)	16 (41)	32 (85)
Match schedule	50 (132)	20 (53)	30 (79)
Other preventive exercises	37 (97)	41 (107)	23 (60)
Time consumption	33 (86)	25 (66)	42 (112)
Football coaches (n = 23)			
Effectiveness of NHE	**87** (20)	4 (1)	9 (2)
Personal motivation	**87** (20)	0 (0)	13 (3)
Knowledge of NHE	**83** (19)	17 (4)	0 (0)
Support from board of directors	61 (14)	13 (3)	26 (6)
Match schedule	52 (12)	17 (4)	30 (7)
Time consumption	48 (11)	17 (4)	35 (8)
Motivation of players	44 (10)	9 (2)	48 (11)
Correspondence to training	39 (9)	13 (3)	48 (11)
Other preventive exercises	22 (5)	44 (10)	35 (8)
Medical staff members of football clubs (n = 29)			
Personal motivation	**100** (29)	0 (0)	0 (0)
Effectiveness of NHE	**93** (27)	7 (2)	0 (0)
Knowledge of NHE	**86** (25)	14 (4)	0 (0)
Consensus with staff (eg, coach)	**86** (25)	3 (1)	10 (3)
Motivation of players	**79** (23)	14 (4)	7 (2)
Other preventive exercises	59 (17)	17 (5)	24 (7)
Time consumption	55 (16)	7 (2)	38 (11)
Match schedule	48 (14)	10 (3)	41 (12)
Support from board of directors	45 (13)	14 (4)	41 (12)

Bold items indicate a score ≥75%.

between adherence to, and the effectiveness of, injury-prevention programs to reduce injuries in a real-world setting.

A strong point of this study is the large number of participants (=264). Moreover, recall bias was limited because standardized and easy-to-remember questionnaires that also focused on the recent past were performed. A dissemination procedure based on the results from the original RCT[6] was used to promote the NHE program. This ensured that participants from the trial, as well as the Dutch football medicine community, were updated on the results of the original RCT. However, there was no additional implementation strategy to ensure adoption in a real-world setting.[6,22] This could have limited adoption and adherence of the NHE program. A weak point of the study was the low response rate (45%), but this is similar to the average response rate (53.7 ± 20.4%) to questionnaires.[34] The study team made intensive efforts to contact all players of the original RCT (n = 579) through email, telephone, football club, and social media. However, mostly due to the fact that participating clubs did not have up-to-date contact data of players involved, the research team was unable to contact 295 (51%) of these players. The majority of these players had transferred to other teams; so, contact details were lost. Although low response rates can effect generalizability of study results, this study was still able to reach over 300 stakeholders involved in hamstring injury prevention in amateur football, which is a large sample of participants.[35] Therefore, we do not feel that a higher response would have affected our results and conclusions because the results of this study were derived from a large sample of intended end users, and professional football data show similar results.[21]

The practical implication of our findings is that factors such as personal motivation, experience and knowledge need to be considered when stimulating adherence to hamstring injury-prevention programs. Nordic hamstring exercise adherence was higher among players who were familiar with the program. Thus, it is important to take the time to familiarize players and coaches with the NHE program. Players need knowledge of the NHE program and motivation, and coaches additionally required proof of the effectiveness of the program.

Future studies–specifically on injury prevention in a football setting–should aim to investigate how the key factors for adherence reported by the intended end users and program

Copyright © 2018 Wolters Kluwer Health, Inc. Unauthorized reproduction of this article is prohibited.

deliverers can be translated into strategies to support the successful implementation of injury-prevention programs.

CONCLUSIONS

Adherence among high-level male amateur football players to the NHE program was very low. Adherence in the intervention group was better than in the control group, suggesting that experience with the program is relevant. Personal motivation of each stakeholder (eg, player, coach, medical staff), knowledge of the NHE program, effectiveness of the NHE program, and consensus between staff members need to be stimulated to improve adherence. Only if these conditions are met, will the NHE program be effective in preventing acute hamstring injuries in a real-world setting.

Perspective

This study encompasses some clear messages for clinical practice: (1) adherence among high-level male amateur football players to the NHE program is very low, too low to expect an effect on hamstring injury prevention in a real-world setting; (2) practical experience with the NHE program is relevant to enhance adherence; and (3) personal motivation of each stakeholder (eg, player, coach, and medical staff), knowledge of the NHE program, effectiveness of the NHE program, and consensus between staff members need to be stimulated to improve adherence. Only then can effective hamstring injury prevention lead to an actual hamstring injury reduction in the real-world amateur football setting. Furthermore, great attention is given in the scientific community to hamstring injury research, and many studies on effective preventive measures (such as Nordics) have gained global interest. However, there is much debate about adherence to these exercises, and our findings contribute to this gap in the scientific landscape, aiming to contribute to a framework where evidence-based hamstring injury prevention will be adopted in a real-world setting and actually lead to a hamstring injury reduction.

References

1. Ekstrand J, Waldén M, Hägglund M. Hamstring injuries have increased by 4% annually in men's professional football, since 2001: a 13-year longitudinal analysis of the UEFA Elite Club injury study. *Br J Sports Med.* 2016;50:731–737.
2. Ekstrand J, Hägglund M, Waldén M. Epidemiology of muscle injuries in professional football (soccer). *Am J Sports Med.* 2011;39:1226–1232.
3. Hägglund M, Waldén M, Magnusson H, et al. Injuries affect team performance negatively in professional football: an 11-year follow-up of the UEFA Champions League injury study. *Br J Sports Med.* 2013;47:738–742.
4. Petersen J, Thorborg K, Nielsen MB, et al. Preventive effect of eccentric training on acute hamstring injuries in men's soccer: a cluster-randomized controlled trial. *Am J Sports Med.* 2011;39:2296–2303.
5. Mjølsnes R, Arnason A, Østhagen T, et al. A 10-week randomized trial comparing eccentric vs concentric hamstring strength training in well-trained soccer players. *Scand J Sci Med Sports.* 2004;14:311–317.
6. van der Horst N, Smits DW, Petersen J, et al. The preventive effect of the nordic hamstring exercise on hamstring injuries in amateur soccer players: a randomized controlled trial. *Am J Sports Med.* 2015;43:1316–1323.
7. Finch C. A new framework for research leading to sports injury prevention. *J Sci Med Sport.* 2006;9:3–10.
8. McKay CD, Verhagen E. "Compliance" versus "adherence" in sport injury prevention: why definition matters. *Br J Sports Med.* 2016;50:382–383.
9. Hay MC, Weisner TS, Subramanian S, et al. Harnessing experience: exploring the gap between evidence-based medicine and clinical practice. *J Eval Clin Pract.* 2008;14:707–713.
10. Finch CF. No longer lost in translation: the art and science of sports injury prevention implementation research. *Br J Sports Med.* 2011;45:1253–1257.
11. McKay CD, Steffen K, Romiti M, et al. The effect of coach and player injury knowledge, attitudes and beliefs on adherence to the FIFA 11+ programme in female youth soccer. *Br J Sports Med.* 2014;48:1281–1286.
12. Janz NK, Becker MH. The health belief model: a decade later. *Health Educ Q.* 1984;11:1–47.
13. Rosenstock I. Historical origins of the health belief model. *Health Edu Behav.* 1974;2:328–335.
14. Carpenter CJ. A meta-analysis of the effectiveness of health belief model variables in predicting behavior. *Health Commun.* 2010;25:661–669.
15. Emery CA, Roy TO, Whittaker JL, et al. Neuromuscular training injury prevention strategies in youth sport: a systematic review and meta-analysis. *Br J Sports Med.* 2015;49:865–870.
16. Soligard T, Nilstad A, Steffen K, et al. Compliance with a comprehensive warm-up programme to prevent injuries in youth football. *Br J Sports Med.* 2010;44:787–793.
17. Niven A. Rehabilitation adherence in sport injury: sport physiotherapists' perceptions. *J Sport Rehabil.* 2007;16:93–110.
18. Steffen K, Meeuwisse WH, Romiti M, et al. Evaluation of how different implementation strategies of an injury prevention programme (FIFA 11+) impact team adherence and injury risk in Canadian female youth football players: a cluster-randomised trial. *Br J Sports Med.* 2013;47:480–487.
19. Orr B, Brown C, Hemsing J, et al. Female soccer knee injury: observed knowledge gaps in injury prevention among players/parents/coaches & current evidence (the KNOW study). *Scan J Sport Med.* 2013;23:271–280.
20. Twomey D, Finch CF, Roediger E, et al. Preventing lower limb injuries: is the latest evidence being translated into the football field? *J Sci Med Sport.* 2008;12:452–456.
21. Bahr R, Thorborg K, Ekstrand J. Evidence-based hamstring injury prevention is not adopted by the majority of Champions League or Norwegian Premier League football teams: the Nordic Hamstring survey. *Br J Sports Med.* 2015;49:1466–1471.
22. van der Horst N, Smits DW, Petersen J, et al. The preventive effect of the Nordic hamstring exercise on hamstring injuries in amateur soccer players: study protocol for a randomised controlled trial. *Inj Prev.* 2014;20:e8.
23. Hasson F, Keeney S, McKenna H. Research guidelines for the Delphi survey technique. *J Adv Nurs.* 2000;32:1008–1015.
24. Fortington LV, Donaldson A, Lathlean T, et al. When "just doing it" is not enough: assessing the fidelity of player performance of an injury prevention exercise program. *J Sci Med Sport.* 2015;18:272–277.
25. Lindblom H, Waldén M, Carlfjord S, et al. Implementation of a neuromuscular training programme in female adolescent football: 3-year follow-up study after a randomised controlled trial. *Br J Sports Med.* 2014;48:1425–1430.
26. Lindblom H, Carlfjord S, Hagglund M. Adoption and use of an injury prevention exercise program in female football: a qualitative study among coaches. *Scand J Med Sci Sports.* 2018;28:1295–1303.
27. O'Brien J, Young W, Finch CF. The delivery of injury prevention exercise programmes in professional youth soccer: comparison to the FIFA 11+. *J Sci Med Sport.* 2017;20:26–31.
28. Boffano P, Boffano M, Gallesio C, et al. Rugby athletes' awareness and compliance in the use of mouthguards in the North West of Italy. *Dent Traumatol.* 2012;28:210–213.
29. Verhagen EA, Hupperets MD, Finch CF, et al. The impact of adherence on sports injury prevention effect estimates in randomised controlled trials: looking beyond the CONSORT statement. *J Sci Med Sport.* 2011;14:287–292.
30. van Reijen M, Vriend I, van Mechelen W, et al. Compliance with sport injury prevention interventions in randomised controlled trials: a systematic review. *Sports Med.* 2016;46:1125–1139.
31. Goode AP, Reiman MP, Harris L, et al. Eccentric training for prevention of hamstring injuries may depend on intervention compliance: a systematic review and meta-analysis. *Br J Sports Med.* 2015;49:349–356.
32. McCall A, Dupont G, Ekstrand J. Injury prevention strategies, coach compliance and player adherence of 33 of the UEFA Elite Club Injury

Copyright © 2018 Wolters Kluwer Health, Inc. Unauthorized reproduction of this article is prohibited.

Study teams: a survey of teams' head medical officers. *Br J Sports Med.* 2016;50:725–730.
33. Steffen K, Emery CA, Romiti M, et al. High adherence to a neuromuscular injury prevention programme (FIFA 11+) improves functional balance and reduces injury risk in Canadian youth female football players: a cluster randomised trial. *Br J Sports Med.* 2013;47:794–802.
34. Baruch Y, Holtom BC. Survey response rate levels and trends in organizational research. *Hum Rel.* 2008;61:1139–1160.
35. Gerbase MW, Germond M, Cerutti B, et al. How many responses do we need? Using generalizability analysis to estimate minimum necessary response rates for online student evaluations. *Teach Learn Med.* 2015;27: 395–403.

Copyright © 2018 Wolters Kluwer Health, Inc. Unauthorized reproduction of this article is prohibited.
