# Supplementary material for: Comparative Effectiveness of Multi-Component, Exercise-Based Interventions for Preventing Soccer-Related Musculoskeletal Injuries: A Systematic Review and Meta-Analysis
Source: Healthcare (Basel). 2025 Mar 29;13(7):765. doi: 10.3390/healthcare13070765 (PMC11988859; doi:10.3390/healthcare13070765)
Supplement: Supplementary file 1 [file healthcare-13-00765-s001.zip › Additional material Included literature/Zarei2019.pdf]

Journal Pre-proof

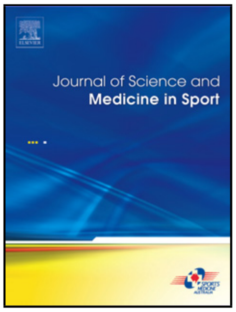

The 11+ Kids warm-up programme to prevent injuries in young Iranian male high-level football (soccer) players: A cluster-randomised controlled trial

Mostafa Zarei, Hamed Abbasi, Parisa Namazi, Mojtaba Asgari, Nikki Rommers, Roland Rössler

PII: S1440-2440(19)30140-9

DOI: https://doi.org/10.1016/j.jsams.2019.12.001

Reference: JSAMS 2210

To appear in: *Journal of Science and Medicine in Sport*

Received Date: 7 February 2019

Revised Date: 27 November 2019

Accepted Date: 1 December 2019

Please cite this article as: Zarei M, Abbasi H, Namazi P, Asgari M, Rommers N, Rössler R, The 11+ Kids warm-up programme to prevent injuries in young Iranian male high-level football (soccer) players: A cluster-randomised controlled trial, *Journal of Science and Medicine in Sport* (2019), doi: https://doi.org/10.1016/j.jsams.2019.12.001

This is a PDF file of an article that has undergone enhancements after acceptance, such as the addition of a cover page and metadata, and formatting for readability, but it is not yet the definitive version of record. This version will undergo additional copyediting, typesetting and review before it is published in its final form, but we are providing this version to give early visibility of the article. Please note that, during the production process, errors may be discovered which could affect the content, and all legal disclaimers that apply to the journal pertain.

© 2019 Published by Elsevier.

Journal Pre-proof

The 11+ Kids warm-up programme to prevent injuries in young Iranian male high-level football (soccer) players: a cluster-randomised controlled trial

Mostafa Zarei[a], Hamed Abbasi[b], Parisa Namazi[a], Mojtaba Asgari[c], Nikki Rommers[d,e,f,g*], Roland Rössler[g,h*]

[*]the authors contributed equally

[a] *Department of Physical Education and Sports Sciences, University of Shahid Beheshti, Tehran, Iran. (Velenjak Square, Evin, Tehran, Iran. 1983963113)*

[b] *Department of Sport Injuries and Corrective Exercises, Sport Sciences Research Institute, Tehran, Iran. (No. 3, 5th Alley, Miremad Street, Motahhari Street, Tehran, Iran. 1587958711)*

[c] *Department of Physical Education and Sports Sciences, University of Tehran, (Faculty of Physical Education and Sport Sciences, between 15th and 16th St., North Kargar st., Tehran, Iran. 1439813117)*

[d] *Department of Movement and Sports Sciences, Vrije Universiteit Brussel, Brussels, Belgium. (Pleinlaan 2, 1050 Brussels, Belgium)*

[e] *Department of Movement and Sports Sciences, Ghent University, Ghent, Belgium. (St. Pietersnieuwstraat 33, 9000 Ghent, Belgium)*

[f] *Research Foundation Flanders (FWO), Belgium (Egmontstraat 5, 1000 Brussels, Belgium)*

[g] *Amsterdam Collaboration on Health & Safety in Sports & Department of Public and Occupational Health, Amsterdam Movement Sciences, VU University Medical Center, Amsterdam, Netherlands (Van der Boechorststraat 7, 1081 BT Amsterdam)*

Journal Pre-proof

[h] *Department of Sport, Exercise, and Health, University of Basel, Basel, Switzerland (Birsstrasse 320 B, CH - 4052 Basel)*

Corresponding author:

Dr. Roland Rössler

Birsstrasse 320 B

4052 Basel, Switzerland

Mail: roland.roessler@unibas.ch

Abstract

Objective: To assess the effectiveness of the 11+ Kids warm-up programme regarding injury reduction in male high-level children's football players.

Design: Cluster-randomised controlled trial

Method: Male youth football teams of Iran's high-level football schools were invited to participate. Inclusion criteria were: teams are competing in the highest league of their province; players are between 7 and 14 years old; regular training takes place at least twice per week. Teams were excluded if they used an injury prevention measure.

Participating clubs were randomised to an intervention (INT, N = 20 teams) and a control group (CON, N = 22 teams), stratified by the number of teams and the age group. The groups were blinded against each other. The follow-up period was one season (9 months). INT replaced their warm-up by 11+ Kids. CON performed a standard warm-up programme. The primary outcome was the injury incidence density (injuries per 1000h of football exposure), compared between groups by incidence rate ratios (RR).

Journal Pre-proof

Journal Pre-proof

Results: In total, 64,047 hours of football exposure of 962 players (INT = 443 players, 31,934 hours of football, CON = 519 players, 32,113 hours of football) were recorded. During the study, 90 (INT = 30; CON = 60) injuries occurred. The overall injury incidence density in INT was reduced by 50% compared to CON (RR 0.50; 95%-CI 0.32, 0.78). No injuries occurred during the execution of the intervention exercises.

Conclusions: The 11+ Kids reduces injuries in high-level children's football players, thus supporting player health and potentially performance and player development.

Funding: [insert after review].

Trial registration: [insert after review]

Keywords: primary prevention; FIFA; efficacy; athletic injures, child, adolescent, warmup

Journal Pre-proof

Journal Pre-proof

Introduction

Football academies, incorporating athlete development programmes, provide optimal training conditions to develop talented young players towards professional athletes.[1] These athlete development programmes are characterised by high pressure, football-specific high-intensity training sessions and a large number of games.[2] The high load leads to a substantial injury risk in football players from a young age.[2, 3] To ensure a safe and successful development, the prevention of football-related injuries is key from a young age onwards.[4, 5]

The football-specific 11+ injury prevention programme, has shown to reduce the risk of injuries in youth players from 14 years of age.[6-8] Recently, the 11+ Kids was developed as a specific injury prevention programme for football players up to 13 years old. This programme is found to improve motor performance,[9] substantially reduce injuries in grassroots-level players,[5, 10] as well as reduce the health-care costs related to those injuries.[11] Because of the simultaneous performance improvement and injury reduction, the 11+ Kids programme could be very beneficial for young high-level players as well. So far, the effects of the 11+ Kids have only been studied in grassroots players. So far there is no evidence whether the positive effects found in previous studies are transferable to players of higher levels of competition, who have clearly defined physical development programmes and train in a more structured way than grassroots level players. Therefore, the aim of the current study was to test the effectiveness of the 11+ Kids regarding the reduction of football related injuries in a large sample of players, playing at the highest level of the country. The organisational structure of the sport asked for a clustered study design. We hypothesised that players of the intervention group would be at lower risk of injury and that the overall injury incidence density in teams of the intervention group would be reduced compared to the control group.

Journal Pre-proof

Journal Pre-proof

Methods

The study was a two-armed cluster-randomised controlled trial, conducted according to the CONSORT statement guideline.[12] All Iranian youth clubs (i.e. football schools) from five provinces of Iran, offering a structured player development programme to a selected group of male players, were invited to participate in the study. Inclusion criteria were: (1) teams are competing in the highest league of their province; (2) players are between 7 and 14 years old at the start of the study, and (3) regular training takes place at least twice per week. Teams were excluded from the study if they already used an exercise-based injury prevention measure.

The clubs who agreed to participate in the study were randomised by [insert initials after review] into the intervention or control group using an automated randomisation procedure. [insert initials after review] had no direct contact with the clubs or team officials and was not involved in the intervention. The number of participating teams per club and the age group served as strata for the randomisation. Teams of the same club were allocated to the same group to minimise the risk of contamination.

The players and their parents received a printed information letter prior to the start of the study. Participation was voluntary. Passive informed consent from the parents was acquired to include children in the statistical analysis. In case children or parents declined participation, parents informed the researchers via e-mail or telephone. All parents of injured players gave their active consent to use the injury data of their child for analysis. The study was approved by the local ethics committee [insert after review].

The observation period comprised one football season from February to November 2017, including a pre-round (6 months) and the final round of the top 20 teams (2 months). Prior to the start of the study, information meetings were conducted to inform coaches about the aims and procedures of the study and, for intervention group teams only, to give detailed instructions on the practical application on the 11+ Kids injury prevention warm-up programme.

Journal Pre-proof

The 11+ Kids programme consists of seven exercises and takes about 20 min to be performed. The exercises focus on unilateral, dynamic stability of the lower extremities, on trunk strength and stability, and on falling technique. Five levels of difficulty allow accounting for performance differences and for general differences in motor skills between individuals (for a detailed description including the manual, please see: https://edoc.unibas.ch/54955/). According to the description in the manual, all teams started with the first level of each exercise and only proceeded to the next level when the coach decided that all players were able to perform the exercise correctly.

Prior to the start of the study, study assistants were thoroughly instructed at the [insert after review] University. The instruction course covered theoretical and practical sessions to educate study assistants. During the first weeks of the season, coaches of the intervention group were instructed by the study assistants on how to use the programme correctly. Coaches received a detailed, printed manual of the 11+ Kids programme in Persian language. The intervention group was asked to use the 11+ Kids programme at least twice a week at the beginning of the training session, replacing their usual warm-up. The control group was stimulated to use a warm-up that represented a "standard warm-up programme" generally used by the participating teams. The warm-up consisted of aerobic activities, dynamic stretching exercises and football-specific movements. The intervention and control group were blinded against each other. Teams of both groups were visited at least every two weeks by our study assistants to monitor whether the intervention teams used the programme and if the control teams did not use (parts of) a structured injury prevention programme but a "regular warm-up".

Football exposure and injury characteristics were assessed following the methods of previous studies on injuries in children's football.[13, 14] Football exposure of teams and (for the intervention group only) information about 11+ Kids usage was entered by the coaches after every training using electronic forms.[15] Coaches sent these forms to the study assistants on a weekly basis. In case no data were received within a period of two weeks, study assistants

Journal Pre-proof

contacted the coach to ask for the data. Data recording of exposure time and injuries in the intervention teams started after the instruction session. Body height and weight of the children were measured and recorded by our study assistants at the start of the study.

An injury was defined as any physical complaint sustained by a player during a scheduled training session or match play leading to the absence from a subsequent training session or match.[15] Injuries were recorded by medical staff (club physiotherapist or club physician). In case of an injury, study assistants contacted the coach, the player, and the parents via telephone and/or e-mail to collect injury data, based on a standardised form to gather additional information on the injury mechanism. In case the player was treated by the club physiotherapist or club physician, the medical staff directly forwarded the respective information to the study assistants. If a player was medically treated elsewhere, parents were instructed to obtain the diagnosis from the treating physician. To ensure an objective injury classification, injury data were checked independently regarding plausibility by two co-authors [insert initials after review], who were blinded to group allocation.

We estimated the sample size for overall injuries based on the assumption that 10% of the players in the control group would sustain an injury.[13] We assumed a preventive effect of 46% (i.e. 5.4% injured players in the intervention group).[16] Based on an alpha level of 0.05 and a power of 80%, 526 players per group were needed.

Anthropometric data of players are described using descriptive statistics (mean and standard deviation). Independent samples t-test was used to compare means between the intervention and control group. Injury incidence densities were calculated as the number of injuries per 1000 hours of exposure. We analyzed the injury burden (lay-off days per 1000 hours) in each group and compared the injury burden between the intervention and control group.

We performed per-protocol analyses. We report injury characteristics descriptively as absolute numbers and percentages for overall, match, and training injuries, as well as for knee, ankle, lower extremity and severe injuries (i.e. leading to an absence longer than 28 days) separately.

Journal Pre-proof

The injury incidence densities (i.e. number of injuries per 1000h of football exposure) are presented with 95% confidence intervals.[17] Rate ratios (RRs) of injury incidence densities between groups were calculated using Stata 14 (StataCorp LP).[17] In addition, to provide a more conservative analysis of our data, we performed an intention-to-treat analysis for overall injuries (incorporating all dropped out players and assuming the injury incidence density of the control group for these players). For the intention-to-treat analysis we followed the same method as for the per-protocol analyses.

To analyze the influence of compliance, the intervention group was split into tertiles according to their 11+ Kids completion rate.[18] “Completion” was defined as the full utilisation of the 11+ Kids warm-up programme (with all of its seven exercises as described in the manual) at the beginning of a training session, based on self-reported data from the coaches.[5] The three subgroups groups (HIGH/MID/LOW completion rate) of the intervention group were compared against each other as well as against CON by calculating rate ratios with 95% confidence intervals.

Journal Pre-proof

Results

Of all 92 Iranian football teams (from 72 clubs) competing at the highest level in the age categories studied, 42 teams (from 35 clubs) decided to participate in this study. Ten teams dropped out after randomisation due to time constraints and were excluded from the analysis. Within the included teams, a total of 12 players in the intervention group and 23 in the control group did not give their consent. Consequently, these players were excluded from the analysis. Please see Figure 1 for the flow of participants.

In total, 64,047 hours of football exposure of 962 players (CON: 519 players, INT: 443 players) were recorded. The mean age of players was 12.2 (SD 1.7) years. The intervention and control group players did not significantly differ at baseline with regards to age, body mass, and height. Further baseline data are presented in Table 1. All players in the intervention group reached difficulty level 5 of the 11+ Kids exercises. No injuries occurred during the execution of the intervention exercises.

**** Please insert Table 1 here ****

**** Please insert Table 2 here ****

The per-protocol analysis showed a reduction of the overall injury incidence density in the intervention group by 50% compared to the control group (Table 2). The total number of days lost due to injury was lower in the intervention group compared to the control group (Table 1). Injury burden (lay-off days per 1000 hours) was 58% lower in the intervention group compared to the control group (RR 0.42 95%-CI 0.37, 0.48). Lower extremity (55% reduction), training (45% reduction) and specifically knee injuries (66% reduction) were reduced in the intervention group compared to the control group. The results regarding match, ankle and severe injuries were not as clear. The according point estimates of the rate ratios do indicate a beneficial effect of the 11+ Kids intervention. However, the respective confidence intervals were too large to draw firm conclusions for the latter injury-subcategories (Table 2). The additional intention-

Journal Pre-proof

Journal Pre-proof

to-treat analysis revealed a reduction in the overall injury incidence density by 39% (RR 0.61 95%-CI 0.42, 0.88).

The compliance analysis showed increasing effectiveness with increasing compliance to the 11+ Kids programme. The risk of injury in the high compliance group was 72% lower compared to the control group and 61% lower compared to low compliance group. Further, the mid compliance group showed a 58% lower injury risk compared to the control group (Table 3).

Discussion

This study is the first to investigate the application of the 11+ Kids warm-up and injury prevention programme in Iranian high-level male youth football players. The application of the 11+ Kids programme reduced football injuries in 7- to 13-year-old high-level, male players in Iran's top (school) competition by half compared to the control group. This is important information because it is known that the injury incidence in high-level youth football is particularly high.[3] Specifically large protective benefits were found for knee injuries (66% reduction) and lower extremity injuries (55% reduction). The total number of days lost to injury and the injury burden (lay-off days per 1000 hours) were clearly lower in the intervention group compared to the control group. Importantly, the compliance analysis showed larger protective effects (i.e. lower injury rates) with higher 11+ Kids completion rates.

The observed reductions of overall injuries, as well as lower extremity injuries in the current study, are comparable to the findings of a recent study on children's football players of lower levels of play [5] and to studies in older football players.[6, 16] The protective effect of knee injuries was slightly higher compared to the effect reported earlier (53% reduction).[5] The observed effect of compliance (i.e. dose-response-relationship) is comparable to a previous study in children's football.[5] Since compliance is an important factor in the effectiveness of an injury prevention programme, future research should focus on the intrinsic motivation of coaches to use these programmes and factors attributing to compliance.

Journal Pre-proof

Neuromuscular injury prevention programmes, such as 11+ Kids, generally focus on improving strength, proprioception, fundamental movement skills and landing strategies.[8, 16, 19] Practitioners in the field of elite level youth football indicated these factors as being related to injury risk.[20] When a player is stronger, he does not only have a better ability to withstand in contact situations but generally also a higher load tolerance, potentially reducing the risk of lower extremity and especially knee injuries.[21] Previous research found positive effects of the 11+ Kids on motor performance and isokinetic strength in a sample of high level players.[22] In combination with the positive effects in injury reduction in the study at hand, the 11+ Kids exercises seem to be of relevance for high-level players as well as grassroots players.

We contacted all clubs playing at the highest level in Iran, but only about 46% agreed to participate in this study, limiting the generalizability of the results. It should be noted that the highest level of play in Iran is not comparable to elite level youth football in Europe. Future studies could investigate the effects of the 11+ Kids in European high-level football.

It has been discussed earlier that blinding of participants regarding group allocation is nearly impossible in studies on injury prevention.[5] Coaches, parents and players reported injury-related information to the researchers, which is a commonly used approach in this type of study.[18, 23, 24] To improve the quality of reporting, all participating coaches were thoroughly instructed regarding the data collection. Our study assistants regularly visited all teams of both groups to provide support and to control the data collection process throughout the study period.

The applied statistical analysis methods do not account for the clustered data structure and/or potential covariates. In a previous study on injury prevention in children's football, it has been shown that the respective adjustment slightly influenced the magnitude of the outcome (i.e. unadjusted Rate Ratio versus an adjusted Hazard Ratio, derived from a mixed effects Cox model).[17] Importantly, the direction of the effect and the conclusion drawn would not have changed in that study.[5, 17] Unfortunately, our sample was slightly smaller than what we aimed for based on the a priori sample size estimation. Nevertheless, the outcomes show clear

(enough) effects. The study was not powered for the analysis of injury subgroups or the influence of compliance. It has to be noted, that despite not initially powered, some effects appear to be interpretable. Results that are not directly interpretable might be included in future meta-analyses. We had a total drop out of about 22% after randomisation (equal in INT and CON). We performed an intention-to-treat analysis, incorporating all dropped out players while assuming the average injury incidence density of the control group for the dropped-out players. The results of this conservative way to analyse the data did show a smaller beneficial effect of the intervention (i.e. 39% versus 50% injury reduction), while still clearly favouring the intervention group.

The observed injury reduction is of relevance from a health and especially from a performance perspective. The number of days lost to injury was cut by more than half, leading to less interruptions of training and absence of match play of intervention group players. Reductions in training load can lead to a reduction in players' performance.[25] Time-loss injuries, per definition, lead to a reduction in training/competition participation and can therefore reduce players' performance. Exercise-based injury prevention programmes (including the 11+ Kids) have shown to directly improve athletes' performance compared to the use of a regular warm-up programme.[9, 26, 27] Therefore, such injury prevention programmes might satisfy the needs of high-level sport, where the training generally aims at maximising performance.

An injury refers to a physical tissue damage [15] which can directly lead to a reduction in performance through physical, physiological and/or biomechanical reactions. For example, after a hamstring strain, the range of motion of the knee is decreased resulting in non-optimal movement patterns, reducing overall performance of the player.[28] Depending on the severity of the injury, the player might need to reduce the current training intensity or even rest completely until recovered sufficiently. Furthermore, injuries are one of the most relevant reasons to drop out from sport participation.[29]

Previously, it has been highlighted that injury prevention should particularly focus on severe injuries.[16, 30] The study was not powered to specifically investigate severe injuries. However,

Journal Pre-proof

the point estimates indicate a clear reduction of severe injuries. Next to a detailed analysis regarding severe injuries, subsequent studies might investigate the effectiveness of 11+ Kids in the field setting and the long-term-effect regarding injury risk and performance. In older athletes, the football-specific injury prevention programme 11+ has successfully been adopted by basketball players.[31] As such, it could be worth investigating whether 11+ Kids might be used and/or adapted for other sports.

Conclusion

The 11+ Kids injury prevention programme is effective in reducing injuries in Iranian high-level football players aged 7-13 years. Overall injuries were reduced by half. Importantly, the preventive effect on knee injuries was particularly large (66% reduction). Next to the beneficial effects on player's health, the 11+ Kids allows players to train and compete more often as the programme clearly reduces the number of days lost to injury. This can positively influence players' and teams' overall performance and might support the athlete development in the long term. These findings highlight the importance and necessity of injury prevention in young athletes, especially on a high-performance level.

Practical implications:

- The warm-up and injury prevention programme 11+ Kids does not require specific equipment and can reduce injuries in high-level football players aged 7-13 years by half.
- The 11+ Kids is relevant for the prevention of lower extremity injuries and particularly knee injuries.
- Investing time into injury prevention should not be regarded as a loss of training time. The 11+ Kids allows players to train and compete more often as the programme clearly reduces the number of days lost to injury, supporting the performance development of talented football players.

Journal Pre-proof

Acknowledgments

We would like to thank the Iranian Physical Education Research Center and the Iranian Football Federation for their support which allowed the realisation of this project. We would like to thank all clubs, coaches, and players for their participation and our study assistants for their valuable support during data collection.

Funding

This research project was kindly supported by the Sport Science Research Institute of Iran. The funding source had no involvement in the conduct and reporting of the study.

Declaration of interest: none

Journal Pre-proof

Journal Pre-proof

References

1. Williams, A.M. and T. Reilly, *Talent identification and development in soccer.* J Sports Sci, 2000. **18**(9): p. 657-67.
2. Read, P.J., et al., *The scientific foundations and associated injury risks of early soccer specialisation.* J Sports Sci, 2016. **34**(24): p. 2295-2302.
3. Read, P.J., et al., *An audit of injuries in six English professional soccer academies.* J Sports Sci, 2017: p. 1-7.
4. Johnson, A., P.J. Doherty, and A. Freemont, *Investigation of growth, development, and factors associated with injury in elite schoolboy footballers: prospective study.* BMJ, 2009. **338**: p. b490.
5. Rössler, R., et al., *A multinational cluster randomised controlled trial to assess the efficacy of '11+ kids': a warm-up programme to prevent injuries in children's football.* Sports Med, 2018. **48**(6): p. 1493-1504.
6. Al Attar, W.S.A., et al., *How effective are F-MARC injury prevention programs for soccer players? A systematic review and meta-analysis.* Sports Med, 2016. **46**(2): p. 205-217.
7. Barengo, N.C., et al., *The impact of the FIFA 11+ training program on injury prevention in football players: a systematic review.* Int J Environ Res Public Health, 2014. **11**(11): p. 11986-2000.
8. Mayo, M., R. Seijas, and P. Alvarez, *Structured neuromuscular warm-up for injury prevention in young elite football players.* Rev Esp Cir Ortop Traumatol, 2014. **58**(6): p. 336-42.
9. Rössler, R., et al., *A new injury prevention programme for children's football - FIFA 11+ Kids - can improve motor performance: a cluster-randomised controlled trial.* J Sports Sci, 2016. **34**(6): p. 549-56.

Journal Pre-proof

Journal Pre-proof

10. Beaudouin, F., et al., *Effects of the '11+ Kids' injury prevention programme on severe injuries in children's football: a secondary analysis of data from a multicentre cluster-randomised controlled trial.* Br J Sports Med, 2018.
11. Rössler, R., et al., *Comparison of the '11+ Kids' injury prevention programme and a regular warmup in children's football (soccer): a cost effectiveness analysis.* Br J Sports Med, 2019. **53**(5): p. 309-314.
12. Campbell, M.K., et al., *Consort 2010 statement: extension to cluster randomised trials.* BMJ, 2012. **345**: p. e5661.
13. Rössler, R., et al., *Soccer injuries in players aged 7 to 12 years: a descriptive epidemiological study over 2 seasons.* Am J Sports Med, 2016. **44**(2): p. 309-17.
14. Rössler, R., et al., *Risk factors for football injuries in young players aged 7 to 12 years.* Scand J Med Sci Sports, 2018. **28**(3): p. 1176-1182.
15. Fuller, C.W., et al., *Consensus statement on injury definitions and data collection procedures in studies of football (soccer) injuries.* Scand J Med Sci Sports, 2006. **16**(2): p. 83-92.
16. Rössler, R., et al., *Exercise-based injury prevention in child and adolescent sport: a systematic review and meta-analysis.* Sports Med, 2014. **44**(12): p. 1733-48.
17. Delfino Barboza, S., R. Rössler, and E. Verhagen, *Considerations and interpretation of sports injury prevention studies.* Clin Sports Med, 2018. **37**(3): p. 413-425.
18. Silvers-Granelli, H., et al., *Efficacy of the FIFA 11+ injury prevention program in the collegiate male soccer player.* Am J Sports Med, 2015. **43**(11): p. 2628-37.
19. Emery, C.A., et al., *Neuromuscular training injury prevention strategies in youth sport: a systematic review and meta-analysis.* Br J Sports Med, 2015. **49**(13): p. 865-70.
20. Read, P.J., et al., *Injury prevention in male youth soccer: Current practices and perceptions of practitioners working at elite English academies.* J Sports Sci, 2018. **36**(12): p. 1423-1431.
21. Frisch, A., et al., *Injuries, risk factors and prevention initiatives in youth sport.* Br Med Bull, 2009. **92**: p. 95-121.

Journal Pre-proof

22. Zarei, M., et al., *The effect of the "11+ Kids" on the isokinetic strength of young football players.* Int J Sports Physiol Perform, 2019: p. 1-19.

23. Faude, O., et al., *Head injuries in children's football-results from two prospective cohort studies in four European countries.* Scand J Med Sci Sports, 2017. **27**(12): p. 1986-1992.

24. Soligard, T., et al., *Comprehensive warm-up programme to prevent injuries in young female footballers: cluster randomised controlled trial.* BMJ, 2008. **337**: p. a2469.

25. Gabbett, T.J., et al., *The relationship between workloads, physical performance, injury and illness in adolescent male football players.* Sports Med, 2014. **44**(7): p. 989-1003.

26. Faude, O., et al., *Neuromuscular adaptations to multimodal injury prevention programs in youth sports: a systematic review with meta-analysis of randomized controlled trials.* Front Physiol, 2017. **8**: p. 791.

27. Zarei, M., et al., *Long-term effects of the 11+ warm-up injury prevention programme on physical performance in adolescent male football players: a cluster-randomised controlled trial.* J Sports Sci, 2018. **36**(21): p. 2447-2454.

28. Opar, D.A., M.D. Williams, and A.J. Shield, *Hamstring strain injuries: factors that lead to injury and re-injury.* Sports Med, 2012. **42**(3): p. 209-26.

29. Crane, J. and V. Temple, *A systematic review of dropout from organized sport among children and youth.* Eur Phys Educ Rev, 2015. **21**(1): p. 114-131.

30. Chalmers, D.J., *Injury prevention in sport: not yet part of the game?* Inj Prev, 2002. **8 Suppl 4**: p. IV22-5.

31. Longo, U.G., et al., *The FIFA 11+ program is effective in preventing injuries in elite male basketball players: a cluster randomized controlled trial.* Am J Sports Med, 2012. **40**(5): p. 996-1005.

Figure 1. Flow of study participants.

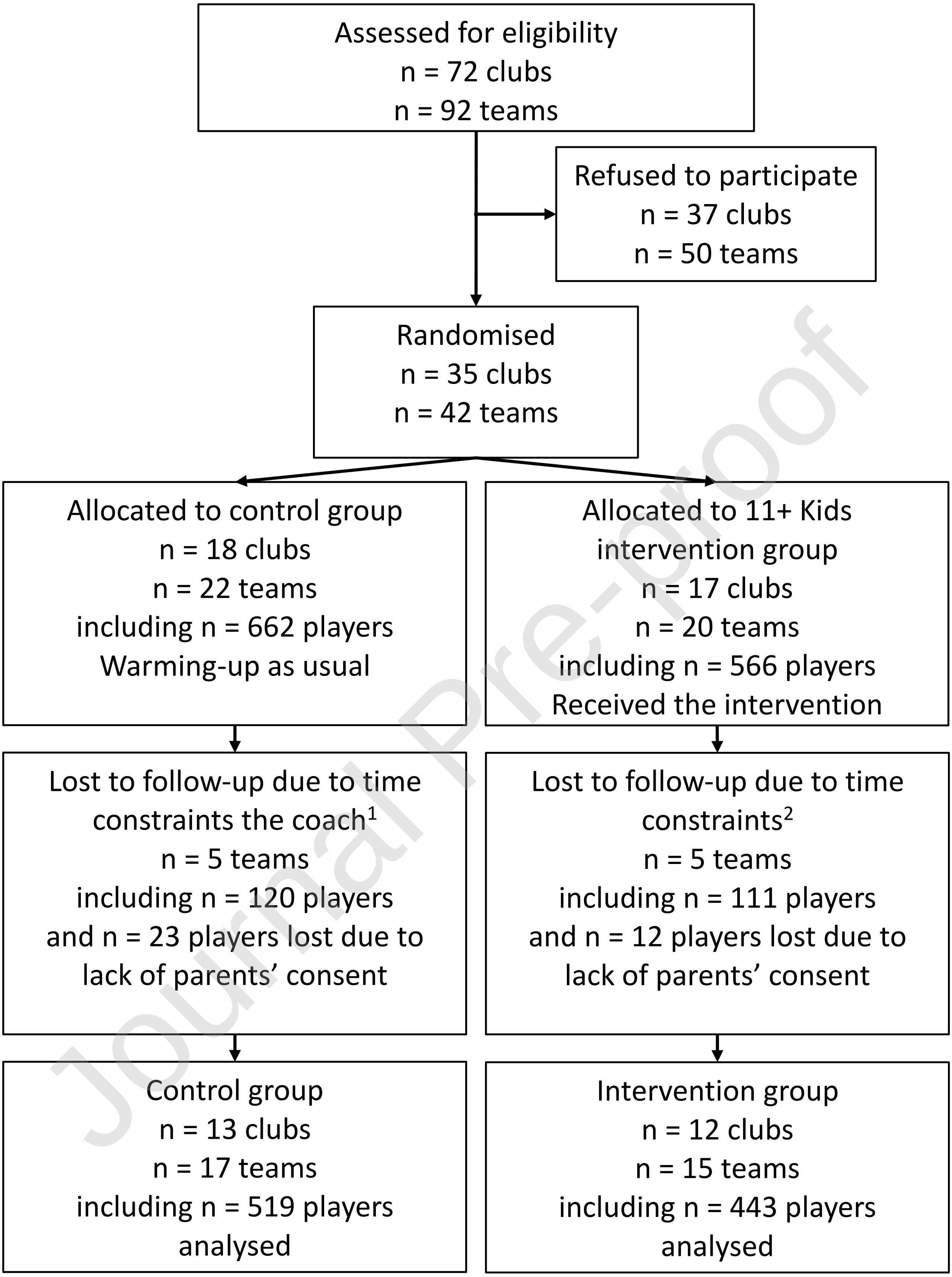

Journal Pre-proof

Journal Pre-proof

1 Coaches did not have the time to take up the extra workload to deliver accurate exposure/injury data needed for the analyses.

2 Three teams dropped out as the coaches did not have the time to take up the extra workload to deliver accurate exposure/injury data needed for our analyses; two further teams withdrew after several weeks of training, expressing that they did not want to continue implementing the program due to a “loss of training time” when using the 11+ Kids.

Journal Pre-proof

Journal Pre-proof

Table 1: Player and injury characteristics of the control (CON) and 11+ Kids intervention (INT) group

	CON	INT
**Number of teams [N]**	17	15
**Number of players [N]**	519	443
**Age [y]**	12.2 (1.7)	12.1 (1.8)
**Body height [cm]**	150 (16)	149 (15)
**Body mass [kg]**	44.8 (13.0)	43.3 (12.8)
**Total exposure [h]**	32113	31934
**Match exposure [h]**	2397	1908
**Training exposure [h]**	29716	30026
**Number of injuries during the study period by time loss**		
**Total number of injuries [N] (%)**	60	30
**1-3 d [N] (%)**	16 (26.7)	6 (20.0)
**4-7 d [N] (%)**	21 (35.0)	12 (40.0)
**8-28 d [N] (%)**	18 (30.0)	11 (36.7)
**> 28 d [N] (%)**	5 (8.3)	1 (3.3)
**Sum of days lost to injury [d]**	664	278
**Mean lay-off time [d] (95%-CI)**	11.1 (6.9, 15.2)	9.3 (6.3, 12.2)
**Injury burden [d/1000h] (95%-CI)**	20.7 (19.2, 22.3)	8.7 (7.7, 9.8)

y = years, cm = centimetre, kg = kilogram, h = hour, d = days, 95%-CI = 95% confidence interval

Journal Pre-proof

Journal Pre-proof

Table 2: Number and percentage of injuries in the control (CON) and 11+ Kids intervention (INT) group. Injury incidence density (IID) in CON and INT and rate ratio (RR) between CON and INT.

	CON (N = 60 injuries)			INT (N = 30 injuries)			
**Injury**	**N**	**%**	**IID (95%-CI)**	**N**	**%**	**IID (95%-CI)**	**RR (95%-CI)**
Overall	60	100	1.87 (1.45; 2.41)	30	100	0.94 (0.66; 1.34)	0.50 (0.32; 0.78)
Match	24	40	10.01 (6.71; 14.94)	10	33	5.24 (2.82; 9.74)	0.52 (0.25; 1.09)
Training	36	60	1.21 (0.87; 1.68)	20	67	0.67 (0.43; 1.03)	0.55 (0.32; 0.95)
Knee	18	30	0.56 (0.35; 0.89)	6	20	0.19 (0.08; 0.42)	0.34 (0.13; 0.84)
Ankle	16	27	0.50 (0.31; 0.81)	9	30	0.28 (0.15; 0.54)	0.57 (0.25; 1.28)
Lower extremity	54	90	1.68 (1.29; 2.20)	24	80	0.75 (0.50; 1.12)	0.45 (0.28; 0.72)
Severe	5	8	0.16 (0.06; 0.37)	1	3	0.03 (0.00; 0.22)	0.20 (0.02; 1.72)

IID = injury incidence density, 95%-CI = 95% confidence interval, RR = rate ratio

Table 3: Results of the compliance analysis comparing different compliance groups (team-based tertile split according to 11+ Kids sessions per week: LOW, MID, HIGH) and the control group (CON).

	CON	LOW	MID	HIGH
**N players**	519	146	155	142
**N injuries**	60	15	9	6
**Total exposure [h]**	32113	10192	10983	10759
**IID per 1000h (95%-CI)**	1.87 (1.45; 2.41)	1.44 (0.87; 2.39)	0.84 (0.43; 1.61)	0.56 (0.25; 1.24)
**11+ Kids sessions per week [N] with SD**	-	1.1 (0.3)	1.9 (0.2)	2.8 (0.3)
**Comparison [RR with (95%-CI)]**				
**LOW**	0.73 (0.42; 1.29)	-		
**MID**	0.42 (0.21; 0.85)	0.58 (0.25; 1.32)	-	
**HIGH**	0.28 (0.12; 0.65)	0.39 (0.15; 0.99)	0.67 (0.24; 1.88)	-

IID = injury incidence density, 95%-CI = 95% confidence interval, RR = rate ratio

Journal Pre-proof
